# Divergent Reactions of Coumarin- and 7-Azacoumarin-3-Carboxylic Acids with Azomethine Ylides: Spirocyclization and Ring Expansion

**DOI:** 10.3390/ijms27219600

**Published:** 2026-10-27

**Authors:** Alexey V. Trifonov, Nurbol O. Appazov, Lyudmila K. Kibardina, Almir S. Gazizov, Alexandra D. Voloshina, Anna P. Luybina, Alexey B. Dobrynin, Igor A. Litvinov, Aiym E. Tolegen, Alexander R. Burilov, Michail A. Pudovik, Rakhymzhan A. Turmanov, Nurgali I. Akylbekov, Igor V. Alabugin, Elena A. Chugunova

**Affiliations:** 1 Arbuzov Institute of Organic and Physical Chemistry, FRC Kazan Scientific Center, Russian Academy of Science, Arbuzova Str., 8, Kazan 420088, Russia; kibardina@iopc.ru (L.K.K.); agazizov@iopc.ru (A.S.G.); micrbi@iopc.ru (A.D.V.); luybina@iopc.ru (A.P.L.); aldo@iopc.ru (A.B.D.); litvinov@iopc.ru (I.A.L.); burilov@iopc.ru (A.R.B.); pudovik@iopc.ru (M.A.P.); chugunova.e.a@gmail.com (E.A.C.); 2 Laboratory of Engineering Profile “Physical and Chemical Methods of Analysis”, Korkyt Ata Kyzylorda University, Aiteke Bi Street, 29A, Kyzylorda 120014, Kazakhstan; rakowrakha@gmail.com (R.A.T.); nurgaliakylbekov@gmail.com (N.I.A.); 3 Institute for Radio-Electronics and Telecommunications, Department for Design and Techniques of Electronic Tools Manufacturing, Kazan National Research Technical University Named After A. N. Tupolev-KAI, Karl Marx Str., 10, Kazan 420111, Russia; 4 Institute Of Petroleum, Chemistry And Nanotechnologies, Department of General Organic and Petrochemical Synthesis Technology Named After Prof. G. Kamai, Kazan National Research Technological University, Karl Marx Str., 68, Kazan 420015, Russia; aiym-tolegen@mail.ru; 5 Department of Chemistry and Biochemistry, Florida State University, 102 Varsity Way, Tallahassee, FL 32306-4390, USA; alabugin@chem.fsu.edu

**Keywords:** azacoumarin, coumarin, 1,3-dipolar cycloaddition, cytotoxicity, anti-tumor, X-ray structure

## Abstract

A series of new spiro-fused polycyclic systems has been synthesized via cascade reactions of 7-azacoumarin-3-carboxylic acid and coumarin-3-carboxylic acid derivatives with azomethine ylides generated in situ from L-proline or sarcosine and various carbonyl components (ninhydrin, aceanthrenequinone, isatin, and substituted isatins). For the conventional cycloaddition products (derived from isatin with L-proline or sarcosine), the cascade reactions contain a 1,3-dipolar cycloaddition with a decarboxylation step, affording a single detectable diastereomer in each case. For those products for which single-crystal X-ray structures were obtained, the crystals belong to centrosymmetric space groups, so the compounds are racemic. The reaction of 7-azacoumarin-3-carboxylic acids with the ylides derived from L-proline and ninhydrin or aceanthrenequinone provides the first example of a pyrrolidine-to-piperidine ring expansion (5→6); this transformation is currently demonstrated only for a limited set of substrates and should be regarded as a proof-of-concept rather than a general method. Transformation differs mechanistically from the conventional cycloaddition–decarboxylation; the proposed mechanism is presented as a working hypothesis. The effect of the solvent on the structure of the final products of reaction of 7-azacoumarin-3-carboxylic acid with ylides based on sarcosine and isatins is shown: in methanol, ring-opened esters are formed, whereas in acetonitrile the coumarin fragment is retained. The structures of all products were established by comprehensive NMR spectroscopy, IR, and mass spectrometry. Single-crystal X-ray diffraction was performed for representative compounds **6a**, **10**, **12a**, **17a**, and **20a**. The synthesized compounds were evaluated for their in vitro cytotoxic activity against M-HeLa, HuTu80, and A549 tumour cell lines, as well as against the normal Wi-38 fibroblast line. For compounds **10** and **17d**, the IC_50_ values against M-HeLa cells were numerically similar to those of the reference drug 5-fluorouracil, while compound **6b** gave an IC_50_ against HuTu80 cells numerically similar to that of 5-fluorouracil. Compound **10** gave a numerically lower IC_50_ value than 5-fluorouracil against A549 lung carcinoma cells. These results should be regarded as a preliminary cell-viability screening rather than evidence of therapeutic potential; the spirocyclic scaffolds provide a starting point for further structure–activity investigation.

## 1. Introduction

The development of new drugs and functional materials remains one of the most active frontiers in organic chemistry. Particularly attractive targets are compounds built around nitrogen-containing heterocycles such as pyrrolidine, pyrroline, and piperidine—core motifs of numerous natural alkaloids and pharmacologically active substances [1,2,3].

**Figure 1:**
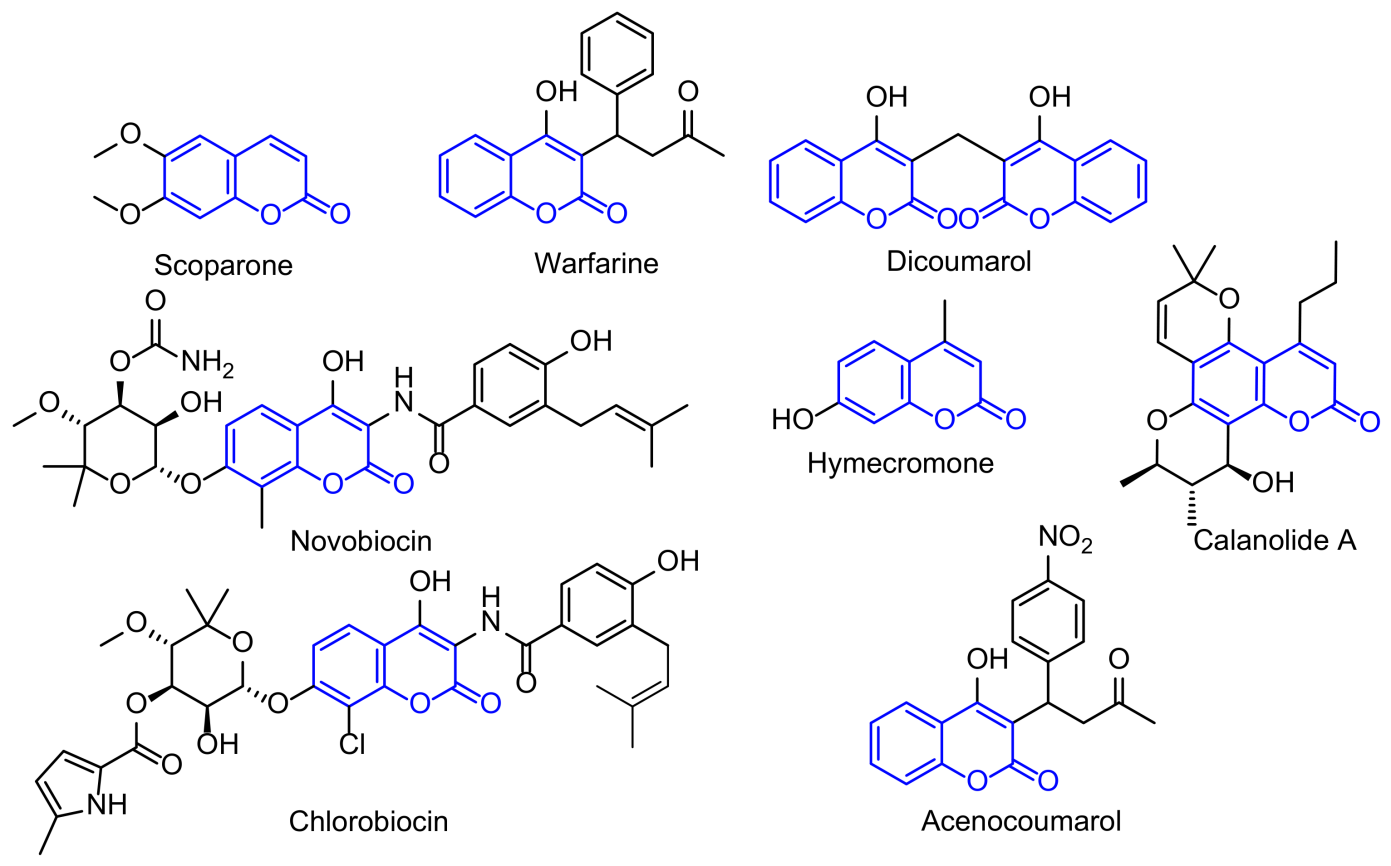
Commercially available biologically active coumarins.

Coumarin (2*H*-chromen-2-one) and its aza-analogues are privileged structural motifs in medicinal chemistry, widely represented in natural products and pharmaceuticals (Figure 1). Well-known examples include the immunosuppressive agent Scoparone [4], the anticoagulants Warfarin [5] and Dicoumarol [6], the antibiotics Clorobiocin and Novobiocin [7], the choleretic drug Hymecromone [8], and the anti-HIV agent Calanolide A [9,10].

Coumarin derivatives exhibit a wide range of biological activities: anti-HIV [11,12], anticancer [13,14,15,16,17], antimicrobial [18,19], antioxidant [20,21,22], anti-Alzheimer [23,24], anti-tuberculosis [25,26], anti-platelet activity [27], COX inhibitors [28], anti-inflammatory [29,30], anti-asthmatic [31,32], anti-viral [33,34] and DNA gyrase inhibitors [35,36]. In addition, fluorescent dyes based on coumarin structures are used as fluorescent probes in various biological systems [37,38].

Coumarin-3-carboxylic acids have emerged as a versatile platform for the construction of polycyclic frameworks. Chithanna et al. exploited them to prepare 3,4-dihydropyridones and 2-piperidinones via reactions with enaminones [39]. Song et al. reported an organocatalytic Diels–Alder reaction of coumarin-3-carboxylic acids with aldehydes, where the carboxyl group both enhanced dienophile reactivity and enabled catalyst release, delivering products in high yield and enantioselectivity [40]. Shankar and Swamy demonstrated an oxidative (4 + 2) cyclization of coumarin/chromene-3-carboxylic acids with alkynes or propargyl alcohols using a Ru(II) catalyst, while Hayashi et al. described a rhodium-catalysed (4 + 2) annulation with alkynes via C4–H bond cleavage [41].

1,3-dipolar cycloaddition is widely used in organic synthesis and chemical biology [42,43]. These reactions are based on the interaction of two molecules: a dipole, which is represented by azomethine ylides formed in situ, and a dipolarophile, such as compounds containing a double or triple bond [44,45,46,47]. The α,β-unsaturated lactone system of coumarins makes them ideal dipolarophiles, opening a direct route to complex polycyclic scaffolds with potential biological activity.

Coumarin-3-carboxylic acids can participate in 1,3-dipolar cycloaddition reactions as the alkene component [48]. Diastereoselective cycloaddition of azomethine ylides, generated in situ from heterocyclic carbonyl compounds such as indenoquinoxaline and isatin, to coumarin derivatives gives access to spiro-fused polyheterocyclic scaffolds [49]. The carboxyl group at C3 plays a dual role: it activates the coumarin ring toward cycloaddition and serves as a handle for subsequent decarboxylative transformations, making C3-carboxylated coumarins particularly attractive substrates compared to their unsubstituted counterparts.

**Scheme 1:**
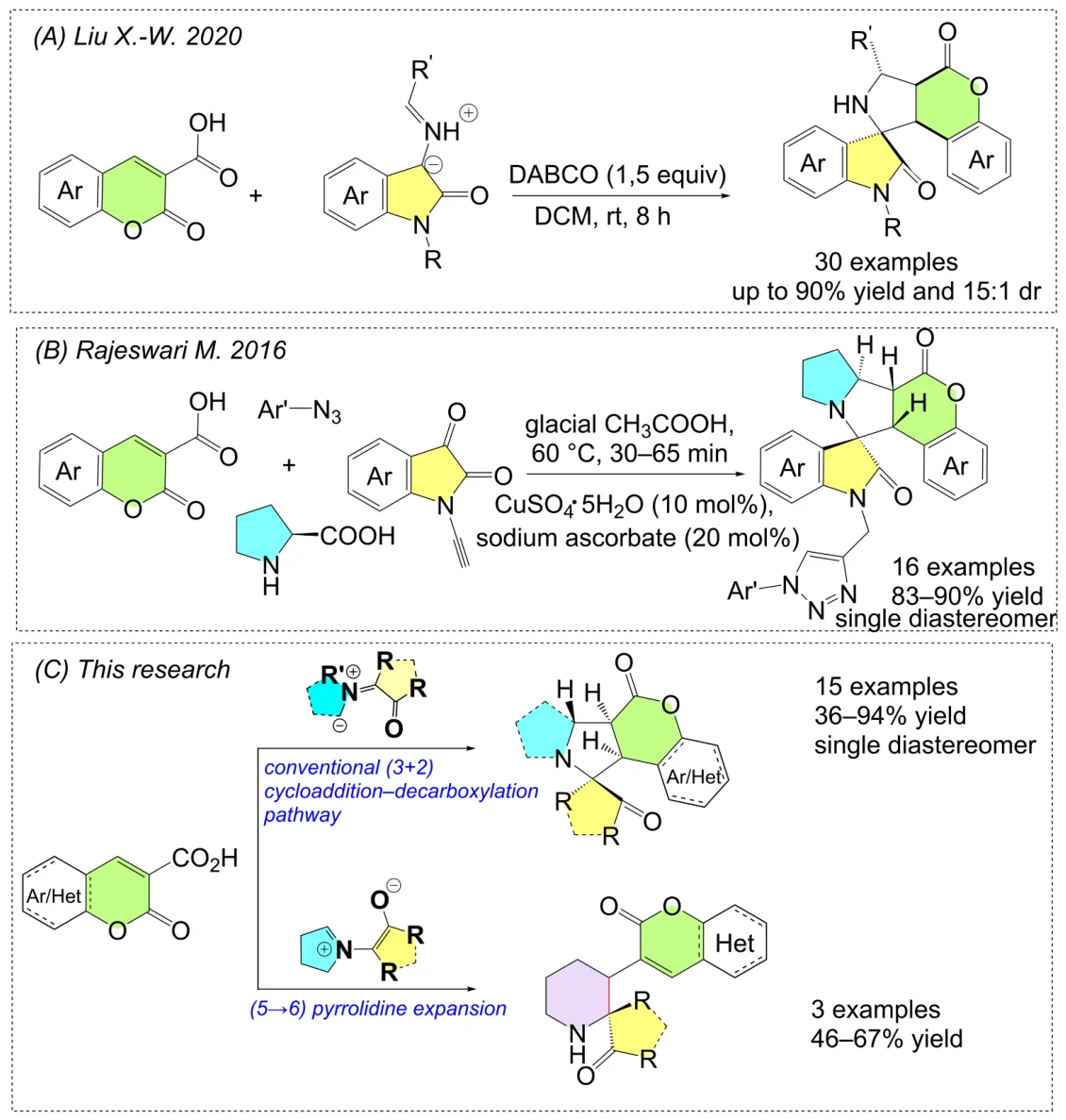
Known synthesis, [50] (**A**), known synthesis, [51] (**B**), and reactivity of azacoumarins disclosed in this work (**C**).

Only isolated examples of 1,3-dipolar cycloaddition involving coumarin-3-carboxylic acids have been reported. Liu et al. [50] described a decarboxylative, regioselective cascade cycloaddition of 3-aminooxindole-derived azomethine ylides with coumarin-3-carboxylic acids (Scheme 1A), producing spiro[oxindole-pyrrolidine-dihydrocoumarin] derivatives bearing four contiguous stereocenters, including a spiroquaternary centre, in up to 90% yield and 15:1 diastereoselectivity.

Rajeswari, Kumari and Khurana reported a one-pot four-component cycloaddition using coumarin-3-carboxylic acid, N-propargylated isatin, L-proline or sarcosine, and aryl azides in the presence of Cu(I) in glacial acetic acid at 60 °C, affording 1,2,3-triazole-conjugated spirooxoindole-pyrrolizines [51] (Scheme 1B).

Together, these precedents establish coumarin-3-carboxylic acids as versatile dipolarophiles whose carboxyl group tunes reactivity and enables downstream decarboxylative ring-forming steps. In the present work, we investigated the reactions of 7-azacoumarin-3-carboxylic and coumarin-3-carboxylic acids, each bearing a reactive endocyclic double bond, with a panel of azomethine ylides derived from L-proline or sarcosine combined with ninhydrin, aceanthrenequinone, isatin, or substituted isatin derivatives (Scheme 1C). The choice of the carbonyl component was found to be critical in determining whether the reaction follows the conventional (3 + 2) cycloaddition–decarboxylation pathway or the ring-expansion pathway. The factors governing this divergence are discussed in Section 2.1. The resulting complex polycyclic frameworks, combining the well-established biological profile of coumarins with newly introduced heterocyclic fragments, are expected to exhibit interesting pharmacological properties.

## 2. Results and Discussion

### 2.1. Chemistry

We began by exploring 1,3-dipolar cycloaddition reactions of 7-azacoumarin-3-carboxylic acid, which carries a reactive endocyclic double bond, with a series of azomethine ylides—to the best of our knowledge, the first application of azacoumarins as dipolarophiles in such reactions. Starting materials **1a**,**b** were prepared by our previously reported method [52].

**Scheme 2:**
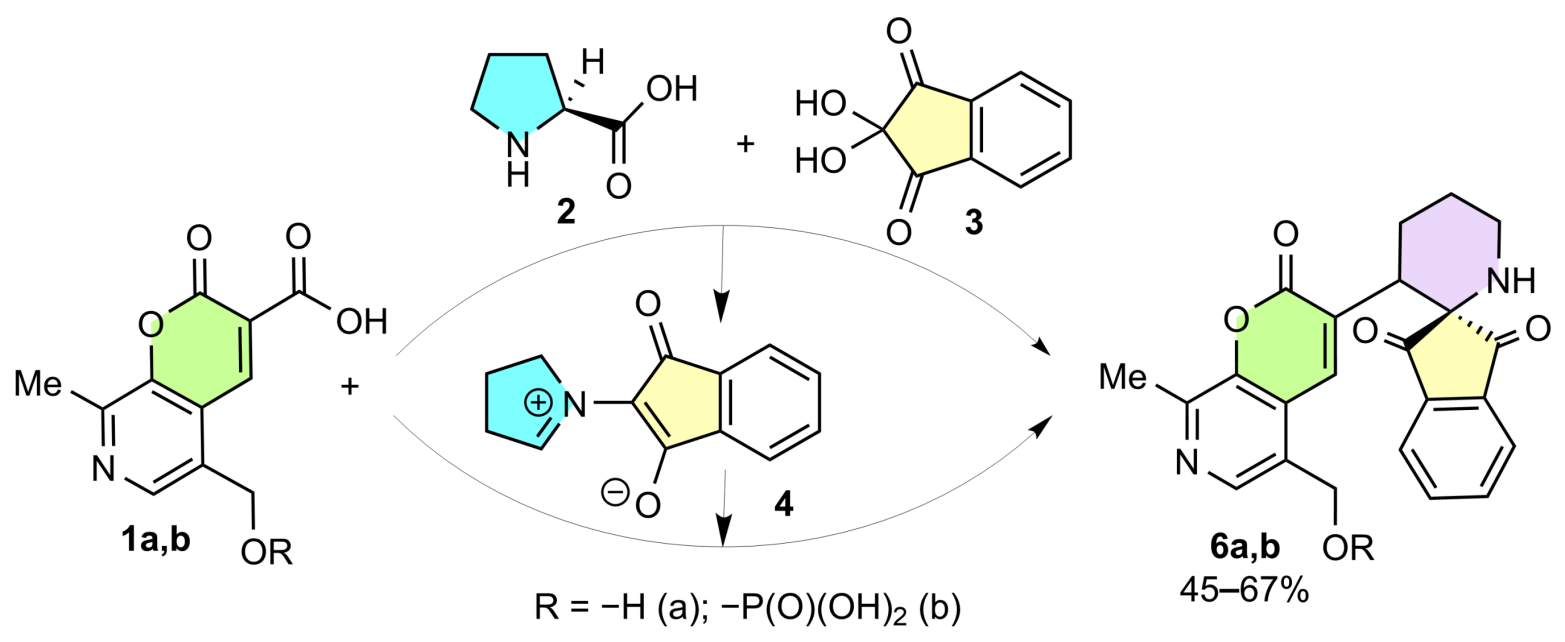
Reaction of 7-azacoumarin-3-carboxylic acids **1a**,**b** with L-proline **2** and ninhydrin **3**/ or azomethine ylide **4**. Conditions: EtOH (abs.), reflux, 1 h (**6a**); MeOH, reflux, 8 h (**6b**). Isolated yields: **6a**, 45% (in situ, solid reagents), 59% (in situ, ethanolic solution) and 67% (preformed **4**); **6b**, 46% (preformed **4**).

Reaction of 7-azacoumarin-3-carboxylic acid **1a** with the azomethine ylide generated in situ from L-proline **2** and ninhydrin **3** was performed in refluxing absolute ethanol. The solvent employed is standard for 1,3-dipolar cycloadditions of this type [53,54]. Compound **6a** was initially obtained in 45% yield by adding 20% excess L-proline and ninhydrin in solid form to a boiling solution of acid **1a**. To determine the most effective preparative protocol for the formation of **6a**, we compared three protocols differing in the mode of reagent addition and the use of preformed azomethine ylide **4** (Table S1, Supplementary Material). Adding a warm ethanolic solution of L-proline and ninhydrin to a refluxing solution of **1a** increased the yield from 45% to 59% compared to the addition of solid reagents. The use of preformed ylide **4** afforded the highest yield (67%). Based on these results, we identified the protocol giving the highest isolated yield for obtaining compound **6a** as the addition of preformed ylide **4** to acid **1a,** refluxing in absolute ethanol, followed by refluxing the reaction mixture for 1 h. Purity was confirmed by ^1^H NMR analysis of the crude mixture and filtrate, which showed only one set of product signals alongside minor unidentified by-product peaks. Conversion was essentially quantitative; the yield loss arose solely from the purification step. Compound **6a** is a ring-expansion product (pyrrolidine-to-piperidine, 5 → 6), not the conventional (3 + 2) cycloadducts described later. The same product **6a** (67% yield) was obtained using pre-formed azomethine ylide **4** (Scheme 2).

To further probe the scope of this cascade, the phosphate-substituted 7-azacoumarin-3-carboxylic acid derivative **1b** was reacted with the pre-formed azomethine ylide **4**. The reaction was carried out in methanol at reflux for 8 h, which differs from the conditions used for **6a** (absolute ethanol, reflux, 1 h). Under these conditions, the spiro-fused product **6b** was isolated in 46% yield (Scheme 2). The phosphate group at the 5-position of the azacoumarin core does not alter the course of the cascade; the product **6b** is structurally analogous to **6a**, as confirmed by ^1^H, ^13^C, and ^31^P NMR spectroscopy (Figures S8–S12, Supplementary Materials). The lower yield of **6b** compared to **6a** (46% vs. 67%) may be attributed to the increased steric bulk and electron-withdrawing nature of the phosphate substituent, which could affect the solubility of the substrate and the efficiency of the final rearrangement step. In addition, the use of methanol instead of ethanol was necessary to achieve complete conversion due to better solubility of the phosphate-containing substrate in the more polar protic solvent. These results demonstrate that the ring-expansion cascade is tolerant of a phosphate substituent on the azacoumarin core, although the reaction conditions require adjustment.

**Figure 2:**
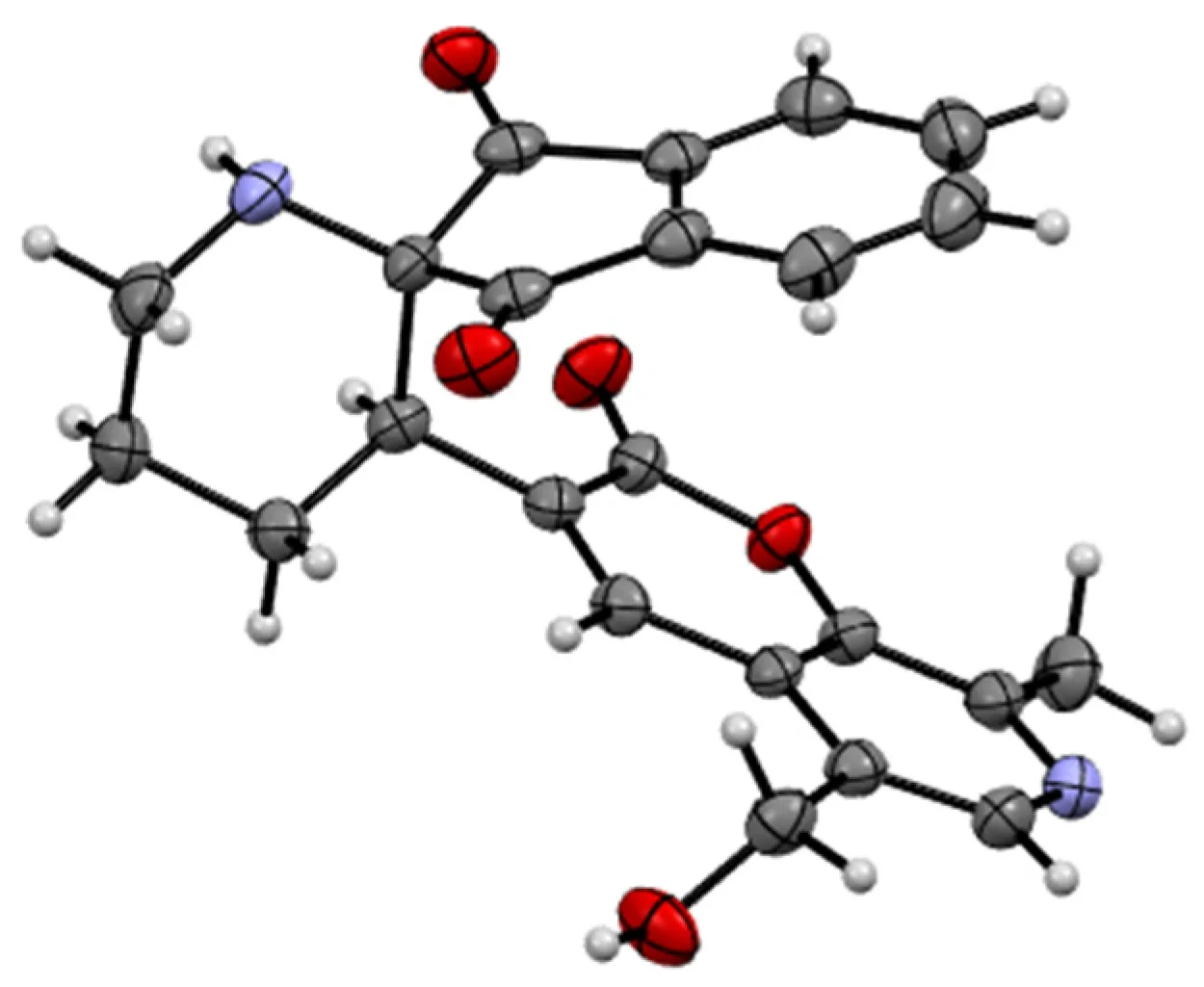
Molecular structure of compound **6a**. Colour code: blue—nitrogen, red—oxygen, dark gray—carbon, light gray—hydrogen.

Single crystals of **6a** suitable for X-ray diffraction were obtained, allowing unambiguous determination of the relative configuration (Figure 2, Figure S1, Tables S2 and S3, Supplementary Materials). The compound crystallizes in the monoclinic space group *P*2_1_/n with two independent molecules in the asymmetric unit. In both molecules, the heterocycle adopts a half-chair conformation along the C13–C18 bond. The two independent molecules differ in configuration at C12: C12A is R and C12B is S.

The complete assignment of signals in ^1^H and ^13^C spectra for compound **6a** was accomplished by using the 2D NMR techniques (COSY, ^1^H-^13^C HSQC and ^1^H-^13^C HMBC). 2D NMR data for compound **6a** are provided in the Supplementary Materials (Figures S45–S47).

**Scheme 3:**
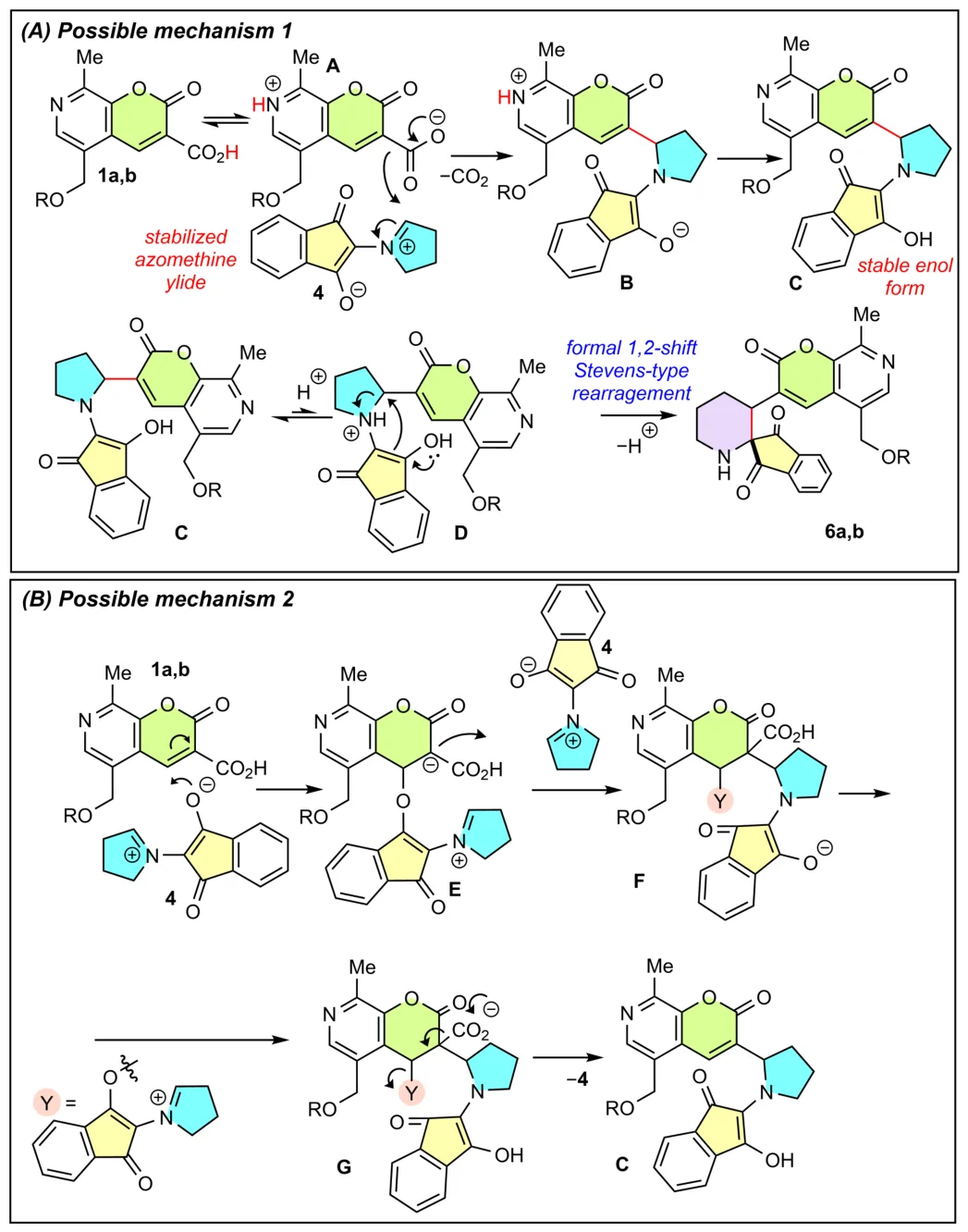

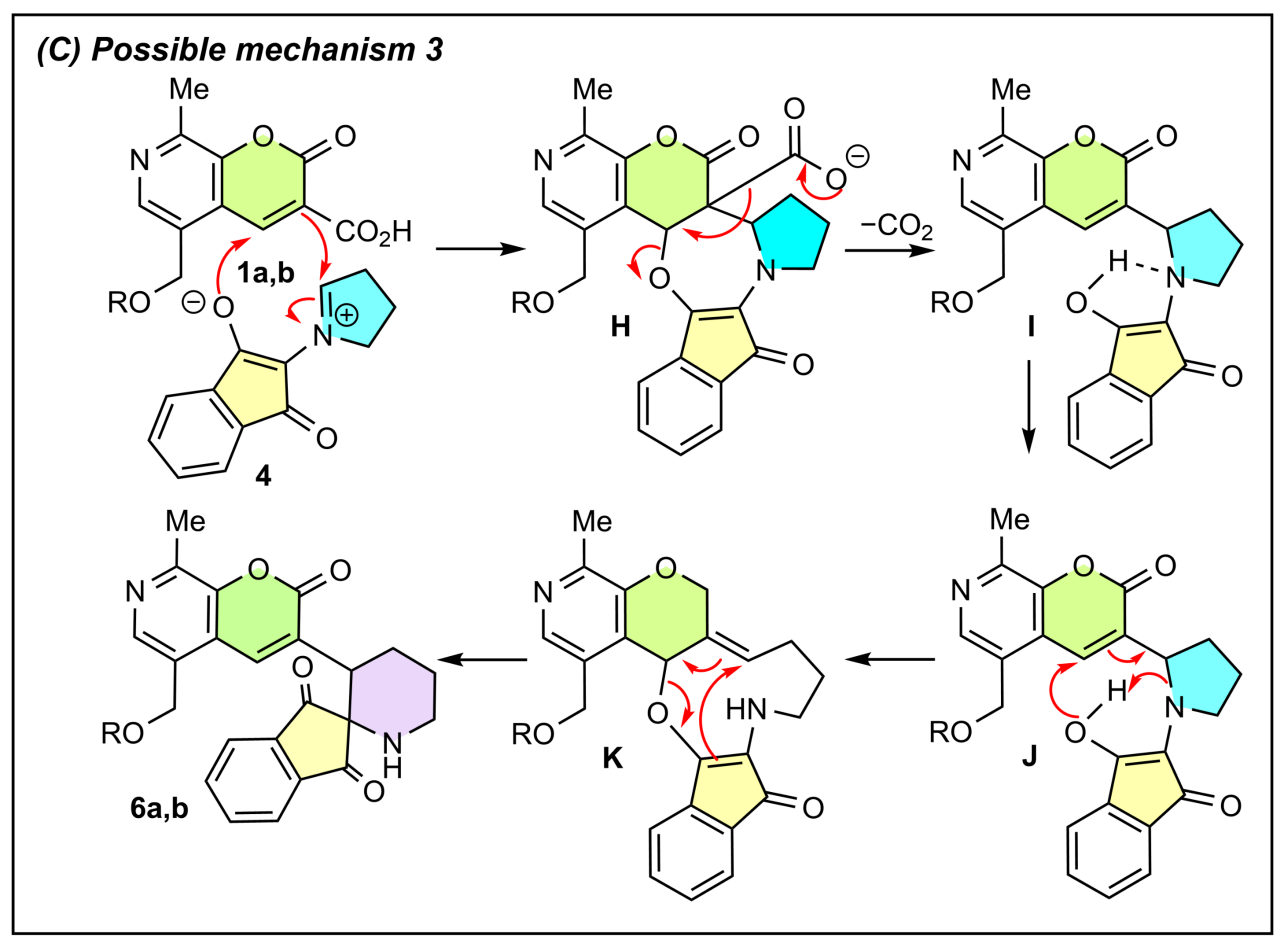
Possible mechanisms for the cascade forming compounds **6a**,**b**.

A plausible mechanism for piperidine ring formation is outlined in Scheme 3.

We speculated that the reaction begins with deprotonation of 7-azacoumarin-3-carboxylic acid to give the corresponding betaine **A**. Given that the structurally similar isonicotinic acid exists, at least partially, as a zwitterion [55,56], this assumption is reasonable. The next step is the electrophilic *ipso*-substitution of the carboxylate group by the stabilized [57,58] azomethine ylide **4**, furnishing intermediate **B**. Mechanistically, this step may proceed via a stepwise decarboxylation–alkylation sequence or a concerted pathway, similar to the known *ipso*-nitration [59,60], *ipso*-arylation [61] and protodecarboxylation [62] of aromatic carboxylates. Although this would lead to high-energy aryl anionic intermediates, such processes are not impossible [63]. Thus, formed charge-separated intermediate **B** then isomerizes to the neutral enol **C**. Notably, the enol **C** may undergo subsequent isomerization to the corresponding keto form. However, given that the equilibrium mixture of many 2-substituted 1,3-indandione derivatives contains a considerable amount of the enol form [64,65] especially in protic solvents, its existence in the present case is plausible. Subsequent steps may involve the Stevens-type rearrangement, resulting in ring expansion and formation of product **6**. Overall, the feasibility of this reaction pathway is determined by (a) deprotonation of the carboxylic acid, which facilitates its decarboxylation; (b) the presence of a persistent azomethine ylide in the reaction mixture; and (c) stabilization of the enol form of intermediate **C**, which enables the ring expansion. The proposed mechanism for the ring expansion is tentative and has not been experimentally demonstrated.

Alternatively, the formation of intermediate **C** may be rationalized by conjugate addition of the enolate to the electron-deficient double bond of the azacoumarin (Scheme 3B). The resulting enolate **E** may then react with a second molecule of ylide **4** to furnish intermediate **G**. Subsequent decarboxylation and β-elimination restore the double bond and regenerate ylide **4**. Intermediate **C** then undergoes the transformations depicted in Scheme 3A to afford the final product.

A third, purely polar pathway, suggested by one of the reviewers, is shown in Scheme 3C. Here the cascade proceeds through conjugate addition, decarboxylative elimination, and C–N fragmentation followed by intramolecular re-closure (intermediates **H**–**K**), without the involvement of an ammonium ylide. For the pathways in Scheme 3A,B, a radical-pair 1,2-shift is not unreasonable, because both fragments would be strongly stabilized: a captodative α-amino radical flanked by the two indandione carbonyl groups, and a migrating carbon conjugated with the azacoumarin π-system. The open question for these pathways is the formation of a genuine ammonium ylide under protic conditions, which the pathway in Scheme 3C avoids. Distinguishing between these alternatives will require further mechanistic study.

**Scheme 4:**
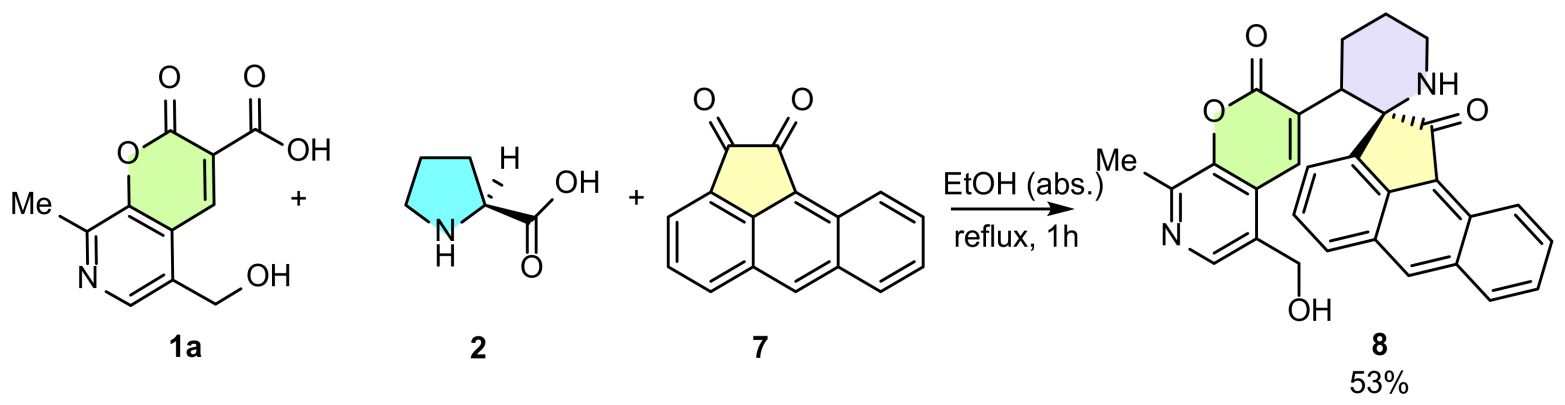
Reaction of azacoumarin-3-carboxylic acid **1a** with L-proline **2** and aceanthrenequinone **7**.

To broaden the scope of this cascade, we examined the reaction of **1a** with the ylide derived from L-proline **2** and aceanthrenequinone **7** in refluxing absolute ethanol. Compound **8** was isolated in 53% yield (Scheme 4). The NMR spectral data of **8** closely parallel those of **6a** and are provided in the Supplementary Materials (Figures S8 and S9 for compound **6a** and Figures S13 and S14 for compound **8**). To confirm the structure of the obtained product **8**, correlation 1D/2D NMR spectroscopy (provided in the Supplementary Materials, Figures S48–S50) was carried out.

**Scheme 5:**
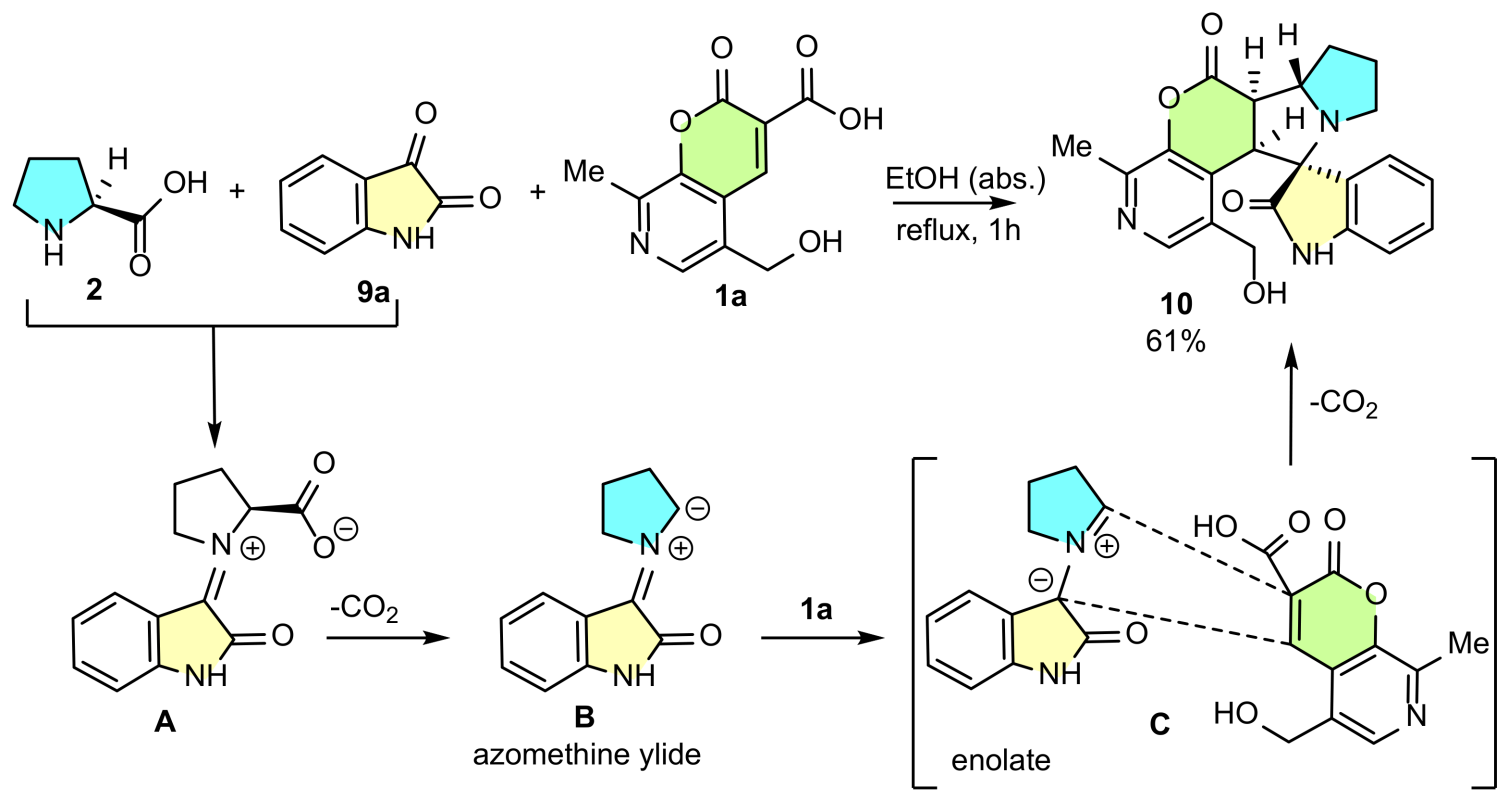
Reaction of azacoumarin-3-carboxylic acid **1a** with L-proline **2** and isatin **9a**. Compound **10** was obtained as a single diastereomer (racemic).

**Figure 3:**
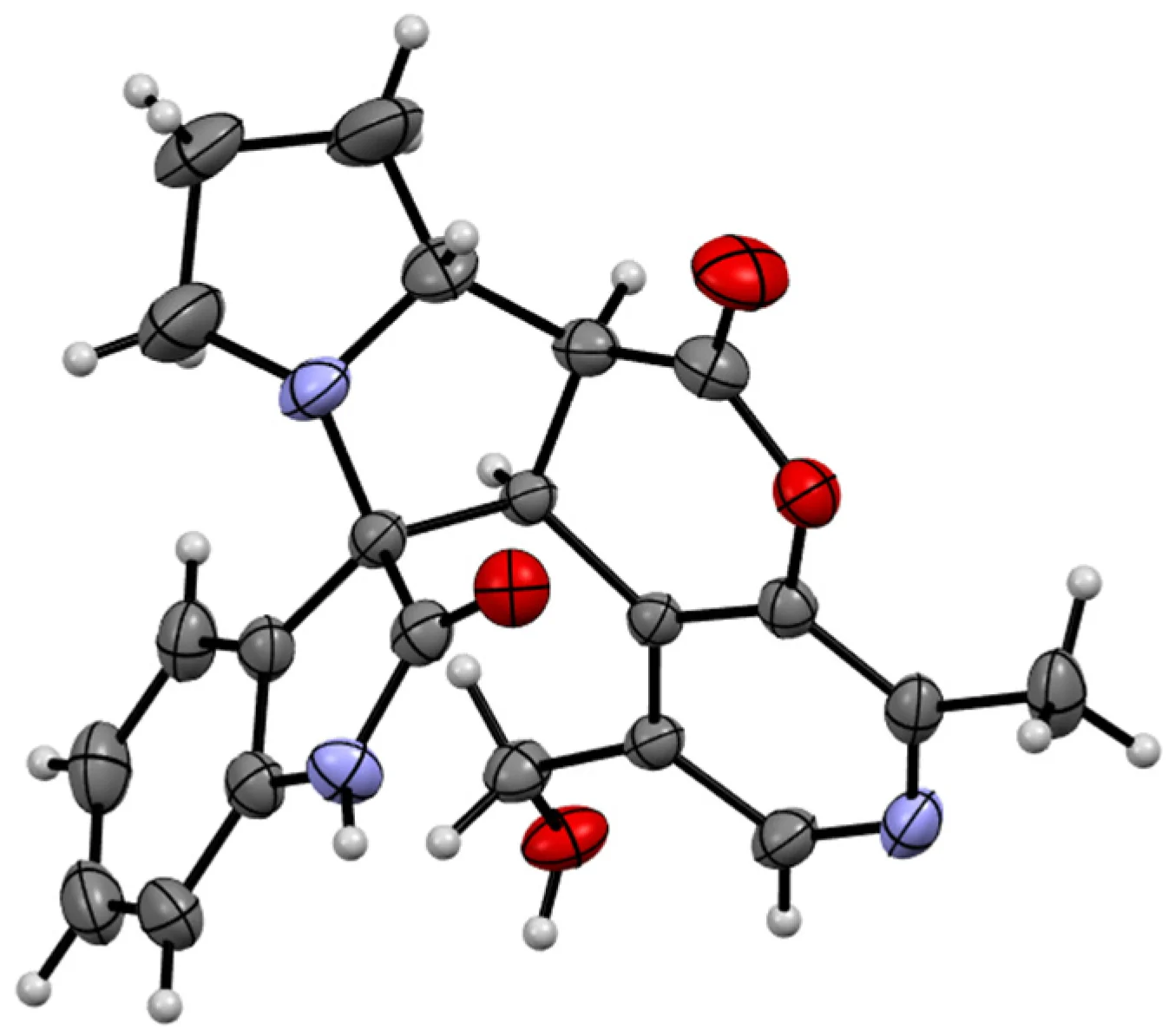
Molecular structure of compound **10**. Colour code: blue–nitrogen, red—oxygen, dark gray—carbon, light gray—hydrogen.

Switching the carbonyl component to isatin **9a** gave a different outcome. The reaction of **1a** with the L-proline/isatin ylide proceeded via a cascade (3 + 2) cycloaddition–decarboxylation sequence, but delivered spiro-fused compound **10** bearing four stereocenters as a single diastereomer (Scheme 5), as confirmed by X-ray analysis (Figure 3, Figure S2, Tables S2 and S3, Supplementary Materials) and the NMR spectral data. The ^1^H and ^13^C spectra for compound **10** are provided in the Supplementary Materials (Figures S15 and S16). Compound **10** crystallizes in the monoclinic space group *P*2_1_/n. The five-membered heterocycle N16–C15–C7–C8–C17 adopts a C7-envelope conformation, with the N16–C15–C8–C17 fragment planar and C7 deviating by 0.475 Å. The six-membered ring O10–C6–C5–C7–C8–C9 adopts a twist-boat conformation, with C7 and C8 displaced to the same side at unequal distances. The configurations C7 R, C8 S, C15 R, C17 R refer to the arbitrarily selected enantiomer shown in Figure 3.

**Scheme 6:**
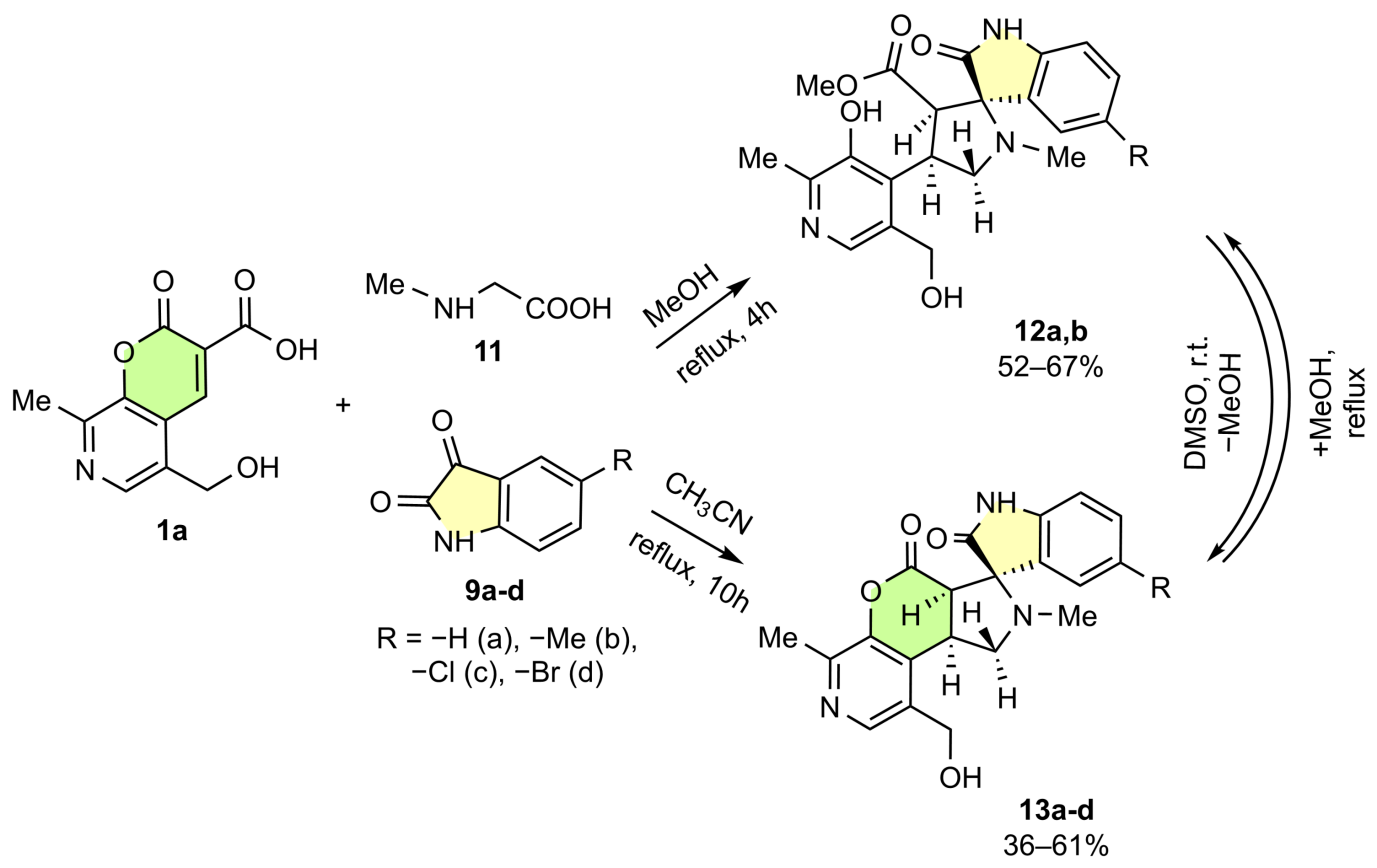
Reaction of azacoumarin-3-carboxylic acid **1a** with sarcosine **11** and isatins **9a**–**d**. Isolated yields: **12a**, 52%; **12b**, 67%; **13a**–**d**, 36–61%. All products were obtained as single detectable diastereomers (racemic).

**Figure 4:**
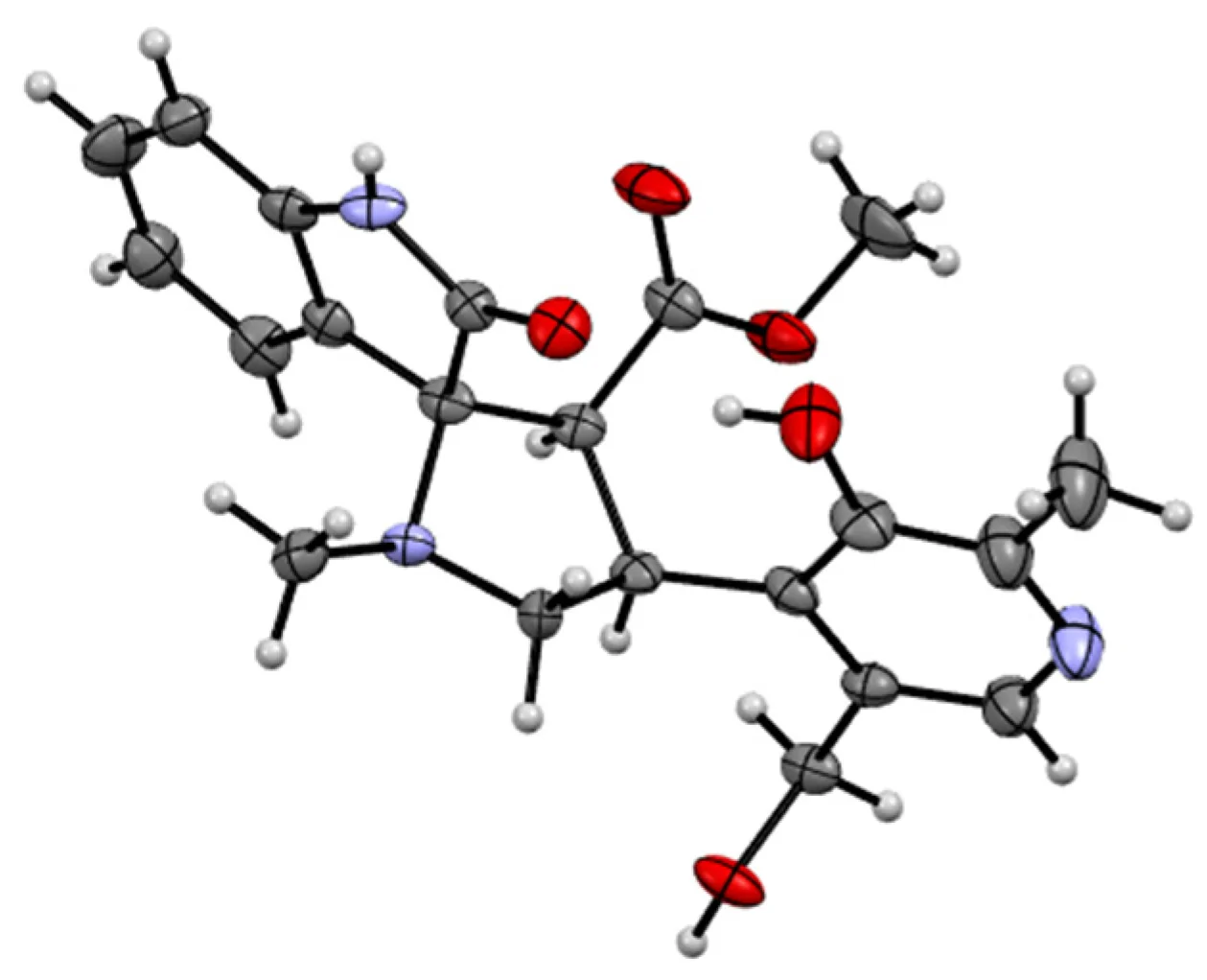
Molecular structure of compound **12a**. Colour code: blue—nitrogen, red—oxygen, dark gray—carbon, light gray—hydrogen.

The reaction of **1a** with ylides derived from sarcosine **11** and isatins **9a**–**d** is notable because the outcome proved to be solvent-dependent. When the reaction is carried out under reflux in methanol, the azacoumarin ring opens to give ring-opened esters **12a**,**b**. To prevent the formation of an ether fragment, we carried out the reaction in an acetonitrile medium, spiro compounds **13a**–**d** are formed (Scheme 6). The interconversion between the two forms was confirmed by dedicated experiments: heating **13a** in methanol caused complete conversion to **12a**, whereas **12a** spontaneously cyclised back to **13a** in DMSO-*d*_6_ at room temperature, reaching completion after seven days (partial conversion already evident after 6 h), as monitored by ^1^H NMR (^1^H NMR spectra of a solution of compound **12a** recorded after standing for 6 h at room temperature in DMSO-*d*_6_ are provided in the Supplementary Materials, Figure S54). The structures of **12a**,**b** and **13a**–**d** were established by ^1^H and ^13^C NMR (provided in the Supplementary Materials, Figures S17–S28), IR spectroscopy, and MALDI-TOF mass spectrometry; the structure of **12a** was additionally confirmed by X-ray crystallography (Figure 4, Figure S3, Tables S2 and S3, Supplementary Materials). Compound **12a** crystallizes in the monoclinic space group *P*2_1_/c; the five-membered heterocycle N1–C2–C3–C4–C5 adopts an N1-envelope conformation (N1 displaced by 0.624 Å, all other rings planar). The configurations C3 R, C4 S, C5 S refer to the arbitrarily selected enantiomer shown in Figure 4. All products **12a**,**b** and **13a**–**d** were obtained as single detectable diastereomers (isolated as racemates).

To confirm the structure of the obtained product **13a**, correlation 1D/2D NMR spectroscopy (provided in the Supplementary Materials, Figures S51–S53) was performed.

**Scheme 7:**
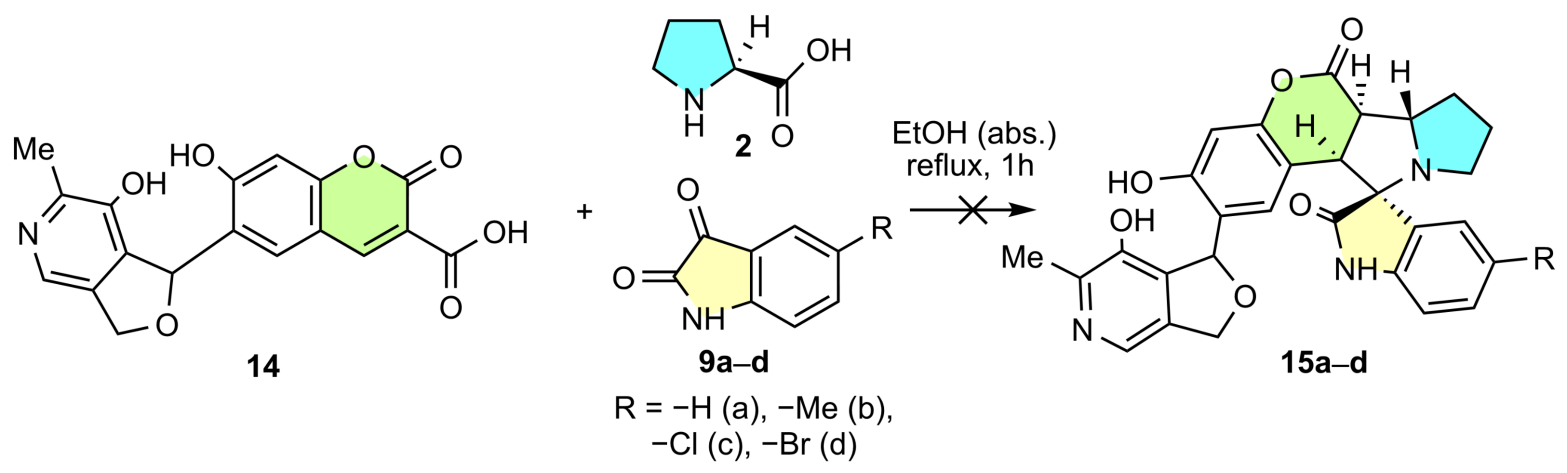
Reaction of coumarin-3-carboxylic acid **14** with L-proline **2** and isatins **9a**–**d**.

In the next stage, we examined the reaction of coumarin-3-carboxylic acid **14**, bearing a bulky vitamin-B_6_-derived furopyridine substituent at C6 (reported previously by us [66]), with azomethine ylides from L-proline **2** and isatins **9a**–**d**. Unexpectedly, acid **14** did not undergo cycloaddition under these conditions (Scheme 7) and returned virtually quantitatively in unchanged form as precipitate. Thus, the issue is not the decomposition of the substrate, but rather its low reactivity under the given conditions.

**Scheme 8:**
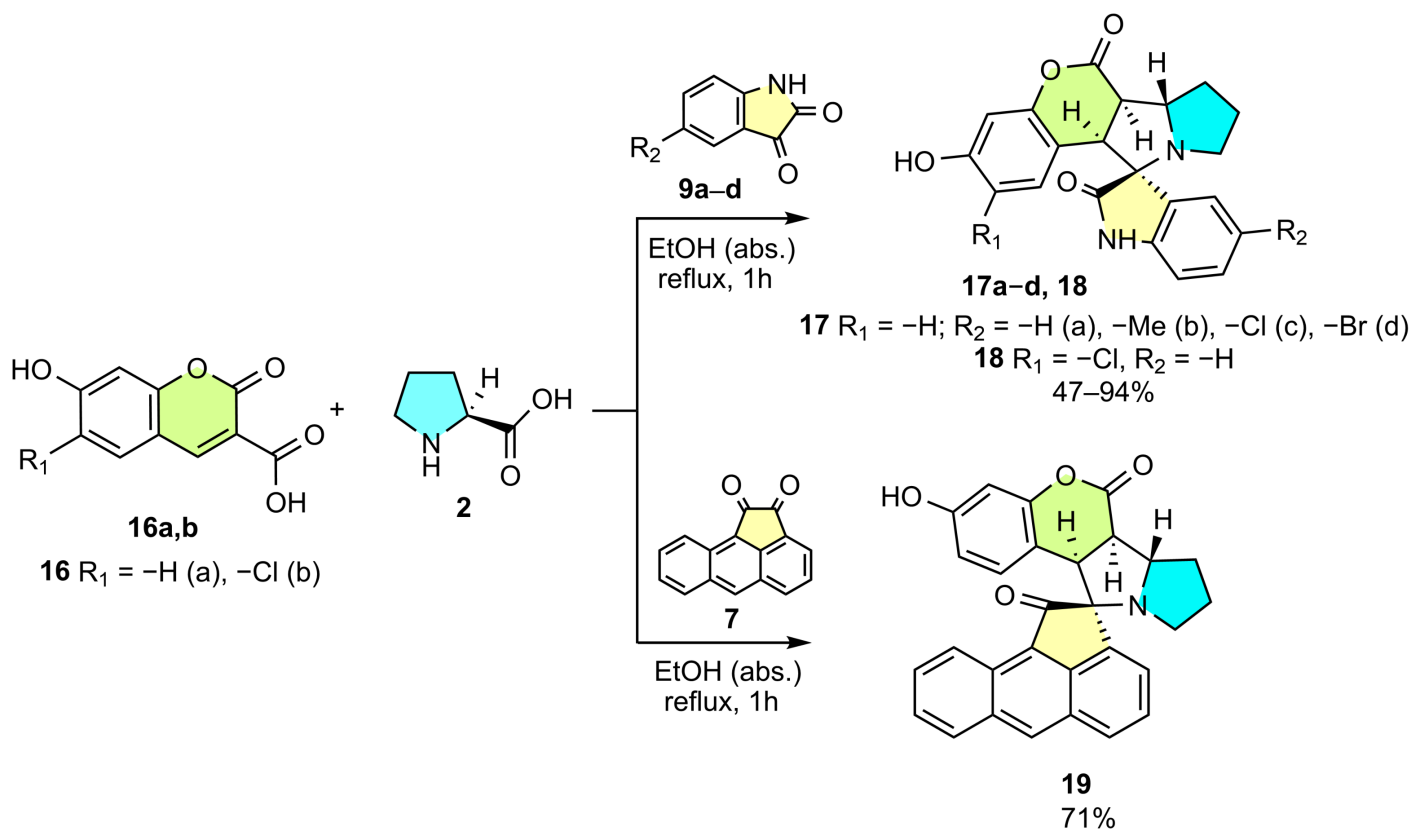
Reaction of coumarin-3-carboxylic acids **16a**,**b** with L-proline **2** and isatins **9a**–**d/** or aceanthrenequinone **7**. All products were obtained as single detectable diastereomers (racemic).

As a comparison, we examined coumarin-3-carboxylic acids **16a**,**b**, which lack the furopyridine fragment, to assess their reactivity under the same conditions. These compounds **16a**,**b** are known in the literature and were obtained using a similar method to compound **1a** (by reacting the corresponding resorcylic aldehyde with Meldrum’s acid in an aqueous medium [52]). They reacted smoothly with azomethine ylides from L-proline **2** and isatins **9a**–**d**, mirroring the behavior of acid **1a** and giving single detectable diastereomers of pyrrolidine-containing spiro compounds **17a**–**d** and **18**. Neither the presence of an electron-withdrawing substituent nor its absence at the 6-position of the aromatic ring affected the outcome. Reaction of **16a** with L-proline **2** and aceanthrenequinone **7** gave product **19** without pyrrolidine ring expansion, in contrast to acid **1a** (Scheme 8). Because **16a** differs from **14** only by the absence of the furopyridine fragment, this comparison directly supports the conclusion that the furopyridine substituent is responsible for the lack of reactivity of **14**. The additional experiment with **16b** (R_1_ = Cl) showed that an electron-withdrawing substituent at C6 does not prevent the reaction, further confirming that the effect is specific to the presence of furopyridine group rather than to substitution at C6 in general. However, exactly how the furopyridine substituent in **14** influences the reactivity-whether through steric shielding, conformational restriction, or electronic effects-remains difficult to establish and requires further investigation.

**Figure 5:**
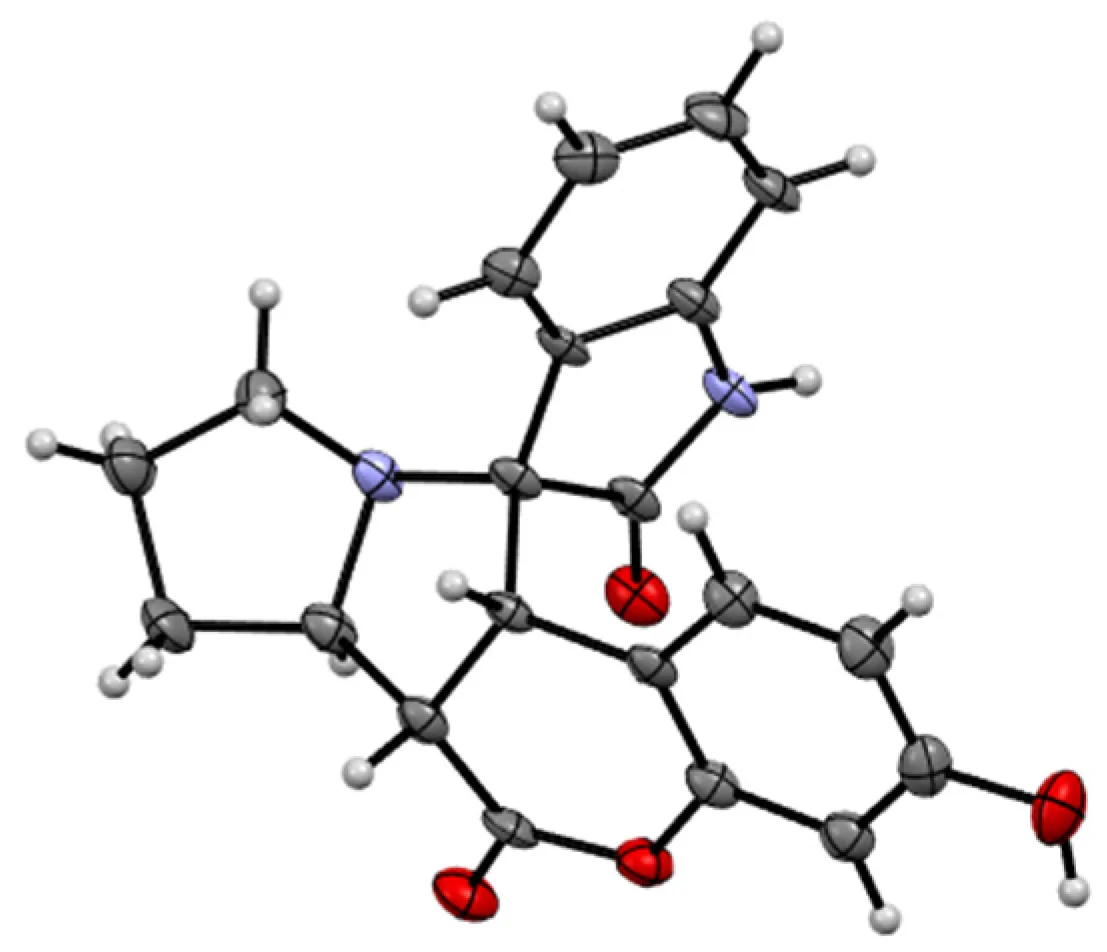
Molecular structure of compound **17a**. Colour code: blue—nitrogen, red—oxygen, dark gray—carbon, light gray—hydrogen.

Primarily the structures of **17a**--**d**,**18**,**19** were established by ^1^H and ^13^C NMR (provided in the Supplementary Materials, Figures S29–S40). Additionally, the structure of **17a** was confirmed by X-ray crystallography (Figure 5, Figure S4, Tables S2 and S3, Supplementary Materials). The compound crystallizes as a DMSO solvate in the monoclinic space group *P*2_1_/n. Both five-membered heterocycles—N5–C4–C3–C7–C6 and N5–C4–C26–C27–C23—adopt half-chair conformations along the C7–C6 and C27–C23 bonds, respectively. The configurations C3 S, C4 R, C6 R, C7 S refer to the arbitrarily selected enantiomer shown in Figure 5.

**Scheme 9:**
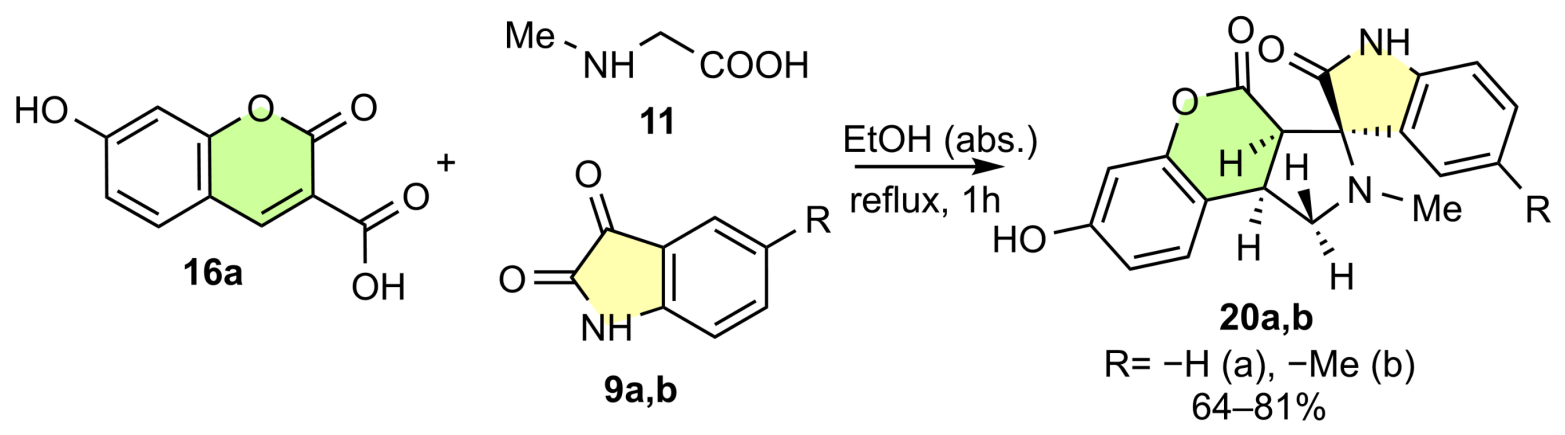
Reaction of coumarin-3-carboxylic acid **16a** with sarcosine **11** and isatins **9a**,**b**. Both products were obtained as single detectable diastereomers (racemic).

**Figure 6:**
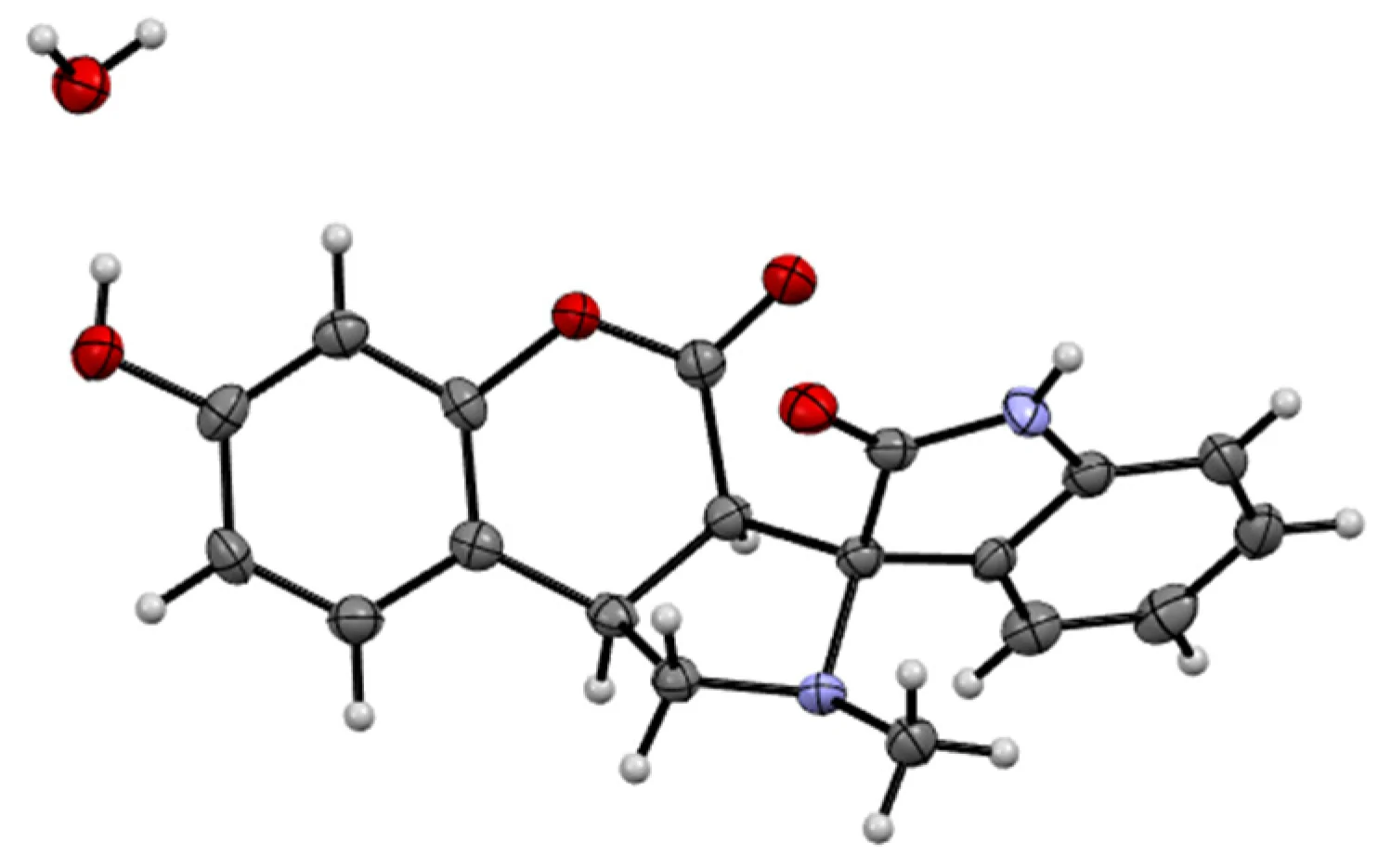
Molecular structure of compound **20a**. Colour code: blue—nitrogen, red—oxygen, dark gray—carbon, light gray—hydrogen.

Reaction of **16a** with sarcosine-derived ylides from isatins **9a**,**b** similarly furnished single detectable diastereomers of compounds **20a**,**b** in 81% and 64% yield, respectively (Scheme 9). The ^1^H and ^13^C spectra for compound **20a**,**b** are provided in the Supplementary Materials (Figures S41–S44). X-ray analysis of **20a** (Figure 6, Figure S5, Tables S2 and S3, Supplementary Materials) confirmed crystallization in the monoclinic space group *P*2_1_/n with 1 molecule of water as solvent; the five-membered heterocycle N5–C4–C3–C7–C6 adopts a half-chair along the C6–N5 bond. The configurations C3 R, C4 R, C7 R refer to the arbitrarily selected enantiomer shown in Figure 6.

For all conventional cycloaddition products (**10**, **12a**,**b**, **13a**–**d**, **17a**–**d**, **18**, **19**, **20a**,**b**), the X-ray structures (where available) reveal a consistent pattern: the newly formed stereocenters adopt a single relative configuration, which is rationalized by a concerted (3 + 2) cycloaddition via an endo-transition state. In this transition state, the dipole approaches the dipolarophile from the less hindered face, with the carbonyl group of the isatin moiety oriented endo relative to the coumarin/azacoumarin framework. This rationale is in full agreement with literature precedents for related azomethine ylide cycloadditions [49,50,51]. For the ring-expansion products **6a**,**b** and **8**, the stereochemical assignment is based solely on the X-ray structure of **6a**; the analogous stereochemical relationships in **6b** and **8** are assumed by analogy, as their NMR data are consistent with the same relative configuration.

The formation of ring-expansion products **6a**,**b** and **8** is mechanistically distinct from the conventional (3 + 2) cycloadducts obtained with isatin-derived ylides. While the latter proceed via a concerted cycloaddition–decarboxylation sequence, the ring expansion involves a more complex pathway that likely proceeds through a stepwise decarboxylative substitution, tautomerization, and Stevens-type rearrangement.

It is important to emphasize that the ring-expansion transformation is currently demonstrated only for a limited set of substrates—specifically, 7-azacoumarin-3-carboxylic acid (**1a**,**b**) in combination with the ylides derived from L-proline and either ninhydrin or aceanthrenequinone. It should therefore be regarded as a proof-of-concept rather than a general method. The factors that determine whether the reaction follows the conventional cycloaddition pathway or the ring-expansion pathway appear to be governed primarily by the nature of the carbonyl component, although the heterocyclic core also contributes: the aceanthrenequinone-derived ylide gives the ring-expanded product **8** with 7-azacoumarin-3-carboxylic acid **1a**, but the conventional cycloadduct **19** with coumarin-3-carboxylic acid **16a**. Specifically, ninhydrin and aceanthrenequinone are stronger electrophiles and more rigid carbonyl components than isatin. Their vicinal tricarbonyl and quinone motifs, respectively, stabilize the enol form of the intermediate C (Scheme 3), which is a prerequisite for the Stevens-type rearrangement and subsequent ring expansion. In contrast, isatin and its substituted derivatives possess an amide NH group capable of participating in hydrogen bonding, and their less electrophilic carbonyl group directs the reaction toward the conventional (3 + 2) cycloaddition–decarboxylation pathway. The ring expansion also requires the presence of a persistent azomethine ylide in the reaction mixture and the stabilization of the enol form of intermediate C in the protic solvent used (absolute ethanol). Steric factors also play a role: the more compact and rigid structures of ninhydrin and aceanthrenequinone may favor the conformational arrangement required for the rearrangement, whereas the more flexible isatin-derived intermediates preferentially undergo the concerted cycloaddition.

Further systematic variation in the carbonyl component would be required to establish the generality of the ring-expansion transformation. At present, the limited substrate scope precludes any claim of broad applicability. The proposed mechanism for the ring expansion is tentative and has not been experimentally demonstrated; it is presented as a working hypothesis.

### 2.2. Preliminary Cell-Viability Screening

With a library of spiro-fused polycyclic compounds in hand, we evaluated their preliminary cytotoxic activity against the normal human fibroblast line Wi-38 and three tumor cell lines—M-HeLa, HuTu80, and A549—at concentrations of 1–100 μM using the MTT assay (Table 1).

Among the compounds tested, **10** and **17d** showed IC_50_ values against M-HeLa cells numerically similar to those of 5-fluorouracil (IC_50_ 59.2 ± 5.6 and 66.9 ± 3.2 μM, respectively, vs. 62.0 ± 4.7μM for the reference drug). Compound **6b** showed moderate activity against HuTu80 cells (IC_50_ 60.3 ± 5.8μM) and showed no measurable cytotoxicity toward the normal Wi-38 line at the highest concentration tested. Compound **10** gave a numerically lower IC_50_ value than 5-fluorouracil against A549 lung carcinoma cells (84.7 ± 2.0 vs. >100 μM); both values are in the high-micromolar range, and no formal statistical comparison between compounds was performed. For comparison, the widely used anticancer agent doxorubicin, included in Table 1 as an additional reference, exhibited IC_50_ values of 3.5 ± 0.2 μM against M-HeLa, 3.2 ± 0.2 μM against HuTu80 cells, 2.0 ± 0.2 μM against A549 cells, and 2.7 ± 0.2 μM against the normal Wi-38 line, confirming its potent but non-selective cytotoxicity. The newly synthesized compounds are considerably less active than doxorubicin. Compound **10** was less cytotoxic to the normal Wi-38 line than to M-HeLa cells under these conditions, but no formal statistical comparison was made and the difference is small relative to the experimental uncertainty; it should therefore be treated as a preliminary observation rather than as evidence of a favourable safety margin. The cell lines used here constitute a standard panel for initial cytotoxicity testing and were not selected for sensitivity to coumarin derivatives. Confirmation in an orthogonal viability assay and in cell lines relevant to coumarin pharmacology would be required before any therapeutic potential could be inferred.

## 3. Materials and Methods

### 3.1. Chemicals

#### 3.1.1. General Methods

^1^H and ^13^C NMR spectra were recorded on a Bruker Avance 400 (Bruker, Billerica, MA, USA) spectrometer (at the frequencies of 400.05, 100.61 MHz, respectively), Bruker Avance III 500 (Bruker Corporation, Rheinstetten, Germany) spectrometer (at the frequencies of 500, 126 MHz, respectively) and on a Bruker Avance 600 spectrometer (at the frequencies of 600.1, 150.9 MHz, respectively and 242.94 MHz for ^31^P NMR). Values of the chemical shifts for the ^1^H and ^13^C nuclei are reported relative to the residual signals of the solvent (DMSO-d_6_). IR spectra were recorded in KBr pellets on a Bruker 3/5 E2_76513 Tensor-27 spectrometer in the range of 400–3600 cm^–1^. Mass spectra were recorded on an Ultraflex III TOF/TOF Bruker instrument (*p*-nitroaniline as the matrix) and an AmaZon X Bruker instrument. Elemental analysis was performed using a Carlo Erba EA 1108 (Carlo Erba, Cornaredo, Italy) instrument.

#### 3.1.2. Single-Crystal X-Ray Diffraction

Data sets for the single crystals **6a**, **10**, **12a**, **17a** and **20a** were collected on a Bruker SMART APEX CCD area detector using MoKα (0.71073 Å) radiation at a temperature of 110 K for **6a**, 150 K for **10** and **12a**, 105 K for **17a** and 293(2) K for **20a**. Data collection and indexing, determination, and refinement of unit cell parameters were carried out using the *APEX*4 software package (Bruker AXS, Madison, WI, USA). Numerical absorption correction based on the crystal shape, additional spherical absorption correction, and systematic error correction were performed using the *SADABS* software [67]. Using *Olex*2 [68], the structures were solved by the intrinsic phasing approach using *SHELXT* [69] and refined by the full-matrix least squares on *F*^2^ using *SHELXL* [70]. Nonhydrogen atoms were refined anisotropically. The positions of hydrogen atoms of methyl groups were found using rotating group refinement with idealized tetrahedral angles. The positions of the H(O) hydrogen atoms were found from Fourier maps, and these atoms were refined isotropically. The other hydrogen atoms were inserted at the calculated positions and refined as riding atoms. Images of the crystal structures were obtained using the software program *Mercury* [71]. Some reflections were omitted to improve the quality of the structure. In the crystal of compound **17a**, one of the DMSO solvent molecules is disordered over two positions with an occupancy ratio of 0.72 to 0.28. High R-factor values arise due to the crystal’s weak reflective capabilities. Structure **17a** contains a solvent-accessible volume of 519 Å^3^; we applied a solvent mask to calculate the position of an undefined solvent. The crystallographic data for structures **6a**, **10**, **12a**, **17a** and **20a** reported in this article have been deposited with the Cambridge Crystallographic Data Center and are freely available on request on the website www.ccdc.cam.ac.uk/structures/ (accessed on 19 October 2026). The crystallographic data for structures **6a**, **10**, **12a**, **17a** and **20a** are listed in Tables S2 and S3.

#### 3.1.3. Procedures for the Synthesis of Compounds

**2-(3,4-dihydro-2*H*-pyrrol-1-ium-1-yl)-1-oxo-1*H*-inden-3-olate 4**

A mixture of L-proline **2** (0.35 g, 3.0 mmol) and ninhydrin **3** (0.53 g, 3.0 mmol) in absolute ethanol (5 mL) was placed in a 25-mL round-bottom flask equipped with a reflux condenser. The reaction mixture was heated under reflux for 1 h. After cooling to room temperature, the precipitated solid was collected by vacuum filtration, washed with absolute ethanol (2 × 5 mL) and diethyl ether (2 × 5 mL), and dried in vacuo to afford the azomethine ylide **4** (0.52 g, 81%) as a yellow solid. The product was sufficiently pure for subsequent transformations and was used without further purification. M.p. 215–218 °C; IR (KBr), ν, cm^−1^: 520, 722, 791, 893, 970, 1039, 1076, 1152, 1215, 1292, 1364, 1408, 1442, 1578, 1623, 1662, 1709, 3090, 3449. ^1^H NMR (400 MHz, DMSO-*d*_6_), δ ppm: 2.13 (2H, p, *J* = 7.9 Hz, CH_2_); 3.03 (2H, tq, *J* = 7.8, 2.5 Hz, CH_2_), 4.72 (2H, td, *J* = 7.8, 2.2 Hz, CH_2_), 7.36 (2H, dd, *J* = 5.2, 3.1 Hz, Ph), 7.46 (2H, dd, *J* = 5.2, 3.0 Hz, Ph), 9.13 (1H, s, CH). ^13^C NMR (101 MHz, DMSO-*d*_6_), δ, ppm: 19.02, 33.52, 59.06, 119.70, 132.02, 137.41, 155.78, 182.29. Found, %: C 73.34; H 5.11; N 6.59. C_13_H_11_NO_2_. Calculated, %: C 73.23; H 5.20; N 6.57.

**3′-(5-(hydroxymethyl)-8-methyl-2-oxo-2*H*-pyrano [2,3-*c*]pyridin-3-yl)spiro[indene-2,2′-piperidine]-1,3-dione 6a**

(1) A 25-mL round-bottom flask equipped with a reflux condenser was charged with 7-azacoumarin-3-carboxylic acid **1a** (0.30 g, 1.28 mmol) and absolute ethanol (5 mL). The mixture was heated to reflux with stirring, whereupon L-proline **2** (0.18 g, 1.53 mmol, 20 mol% excess) and ninhydrin **3** (0.27 g, 1.53 mmol, 20 mol% excess) were added in one portion as solids. The reaction mixture was refluxed with stirring for 1 h. After cooling to room temperature, the solvent was removed under reduced pressure. The product 0.23 g (45%) was isolated by recrystallization of the residue from absolute ethanol (3 mL).
(2) A 25-mL round-bottom flask equipped with a reflux condenser was charged with 7-azacoumarin-3-carboxylic acid **1a** (0.30 g, 1.27 mmol) and absolute ethanol (3 mL). The mixture was heated to reflux with stirring. Separately, a warm solution of L-proline **2** (0.18 g, 1.53 mmol) and ninhydrin **3** (0.27 g, 1.53 mmol) in absolute ethanol (2 mL) was prepared and added dropwise to the boiling reaction mixture. The reaction mixture was refluxed with stirring for 1 h. After cooling to room temperature, the precipitated solid was collected by vacuum filtration, washed with absolute ethanol (2 × 3 mL) and diethyl ether (2 × 3 mL), and dried in vacuo to afford compound **6a** (0.30 g, 59%) as a brown solid.
(3) A 25-mL round-bottom flask equipped with a reflux condenser was charged with 7-azacoumarin-3-carboxylic acid **1a** (0.30 g, 1.27 mmol) and absolute ethanol (5 mL). The mixture was heated to reflux with stirring, and pre-formed azomethine ylide **4** (0.27 g, 1.27 mmol) was added in one portion. The reaction mixture was refluxed with stirring for 1 h. After cooling to room temperature, the precipitate that formed was filtered off and discarded. The filtrate was concentrated under reduced pressure, and the residue was recrystallized from absolute ethanol (3 mL) to afford compound **6a** (0.34 g, 67%) as a brown solid.

M.p. 228–230 °C; IR (KBr), ν, cm^−1^: 699, 721, 753, 782, 874, 896, 936, 1005, 1032, 1048, 1063, 1076, 1091, 1142, 1184, 1251, 1268, 1338, 1417, 1439, 1594, 1706, 1724, 1744, 2874, 2946, 3162, 3293, 3431.^1^H NMR (400 MHz, DMSO-*d*_6_), δ ppm: 1.65–1.72 (1H, m, CH), 1.84 (2H, d, *J* = 11.4 Hz, CH_2_), 2.33–2.42 (4H, m, CH_3_ + CH), 2.91 (1H, d, *J* = 12.6 Hz, CH), 3.28–3.31 (1H, m, CH), 3.48 (1H, dd, *J* = 12.6, 3.2 Hz, CH), 4.49–4.57 (2H, m, CH_2_OH), 5.39 (1H, t, *J* = 5.1 Hz, CH_2_OH), 7.78–7.81 (5H, m, Ph + CH_arom_), 8.17 (1H, S, CH). ^13^C NMR (101 MHz, DMSO-*d*_6_), δ, ppm: 18.65; 25.87; 26.19; 40.79; 40.99; 58.49; 66.33; 121.87; 123.25; 123.71; 130.60; 133.05; 135.88; 136.73; 136.09; 140.12; 141.20; 142.84; 146.10; 146.17; 159.22 199.50. MS (ESI-TOF), m/z: 405.1 [M + H]^+^. Found, %: C 68.37; H 5.06; N 7.02. C_23_H_20_N_2_O_5_. Calculated, %: C 68.31; H 4.98; N 6.93.

**(3-(1,3-dioxo-1,3-dihydrospiro[indene-2,2′-piperidin]-3′-yl)-8-methyl-2-oxo-2*H*-pyrano [2,3-*c*]pyridin-5-yl)methyl dihydrogen phosphate 6b**

A 25-mL round-bottom flask equipped with a reflux condenser was charged with phosphate-substituted 7-azacoumarin-3-carboxylic acid **1b** (0.24 g, 0.762 mmol) and absolute methanol (5 mL). The mixture was heated to reflux with stirring, and pre-formed azomethine ylide **4** (0.16 g, 0.762 mmol) was added in one portion. The reaction mixture was refluxed with stirring for 8 h. After cooling to room temperature, the precipitated solid was collected by vacuum filtration, washed with absolute methanol (2 × 5 mL) and diethyl ether (2 × 5 mL), and dried in vacuo to afford compound **6b** (0.17 g, 46%) as a brown solid. Subsequent purification of the product was not required. M.p. > 300 °C; IR (KBr), ν, cm^−1^: 496, 555, 611, 716, 780, 826, 928, 1049,1083, 1147, 1210, 1255, 1375, 1419, 1440, 1595, 1616, 1736, 2930, 3431. ^1^H NMR (600 MHz, DMSO-*d*_6_), δ ppm: 1.78–1.93 (3H, m, CH), 2.35 (3H, s, CH_3_), 2.43 (1H, qd, *J* = 12.6, 12.2, 2.8 Hz, CH), 3.11 (1H, d, *J* = 11.9 Hz, CH), 3.49 (1H, d, *J* = 12.0 Hz, CH), 3.53–3.56 (1H, m, CH), 4.96 (2H, q, *J* = 9.4, 6.3 Hz, CH_2_OH), 7.75–7.86 (5H, m, Ph + CH_arom_), 8.22 (1H, s, CH). ^13^C NMR (151 MHz, DMSO-*d*_6_), δ, ppm: 18.20; 23.75; 24.50; 40.55; 40.64; 60.97; 65.79; 121.39; 123.14; 123.35; 126.30; 131.98; 135.77; 136.45; 136.93; 139.34; 140.81; 143.41; 145.52; 146.67; 158.52; 197.10; 198.21. ^31^P NMR (243 MHz, DMSO-*d*_6_), δ, ppm: -0.7. MS (MALDI-TOF), m/z: 485.1 [M + H]^+^. Found, %: C 56.96; H 4.40; N 5.51; P 6.34. C_23_H_21_N_2_O_8_P. Calculated, %: C 57.03; H 4.37; N 5.78; P 6.39.

**5-(hydroxymethyl)-8-methyl-3-(1-oxo-1*H*-spiro[aceanthrylene-2,2′-piperidin]-3′-yl)-2*H*-pyrano [2,3-*c*]pyridin-2-one 8**

A 25-mL round-bottom flask equipped with a reflux condenser was charged with 7-azacoumarin-3-carboxylic acid **1a** (0.15 g, 0.638 mmol) and absolute ethanol (2.5 mL). The mixture was heated to reflux with stirring, and L-proline **2** (0.08 g, 0.696 mmol) and aceanthrenequinone **7** (0.16 g, 0.696 mmol) were added in one portion as solids. The reaction mixture was refluxed with stirring for 1 h. After cooling to room temperature, the precipitated solid was collected by vacuum filtration, washed with absolute ethanol (2 × 3 mL) and diethyl ether (2 × 3 mL), and dried in vacuo to afford compound **8** (0.16 g, 53%) as a black solid. The product was sufficiently pure and was used without further purification. M.p. 239–240 °C. IR(KBr), ν, cm^−1^: 491, 506, 530, 572, 624, 686, 748, 759, 776, 813, 857, 912, 983, 1017, 1033, 1089, 1103, 1139, 1175, 1190, 1121, 1266, 1284, 1337, 1362, 1422, 1460, 1576, 1596, 1624, 1701, 1725, 2863, 2935, 3263, 3288. ^1^H NMR (600 MHz, DMSO-*d_6_*), δ ppm: 1.83–1.89 (1H, m, CH), 2.00 (2H, d, *J* = 12.7 Hz, CH), 2.14 (3H, s, CH_3_), 2.44 (1H, dd, *J* = 24.9, 12.3 Hz, CH), 2.71 (1H, br. s, CH), 3.12 (1H, d, *J* = 13.5 Hz, CH), 3.74 (1H, dd, *J* = 13.1, 3.2 Hz, CH), 4.03–4.09 (2H, m, CH_2_OH), 5.15 (1H, t, *J* = 5.6 Hz, CH_2_OH), 7.12 (1H, s, Ph), 7.36 (1 H, t, *J* = 7.7 Hz, Ph), 7.74 (1H, t, *J* = 7.7 Hz, Ph), 7.79 (1H, t, *J* = 7.7 Hz, Ph), 7.96 (1 H, s, CH_arom_), 8.04 (1 H, d, *J* = 8.6 Hz, Ph), 8.18 (1 H, d, *J* = 8.5 Hz, Ph), 8.23 (1 H, d, *J* = 6.7 Hz, Ph), 8.79 (1H, s, CH), 8.89 (1 H, d, *J* = 8.6 Hz, Ph). ^13^C NMR (151 MHz, DMSO-*d*_6_), δ, ppm: 18.43, 26.70, 27.39, 41.99, 42.33, 57.94, 69.77, 121.23, 123.55, 124.11, 124.47, 125.61, 127.05, 127.65, 128.12, 128.28, 129.98, 130.08, 130.15, 133.10, 133.49, 133.62, 139.65, 142.21, 143.28, 145.61, 145.68, 159.29, 202.86. MS (MALDI-TOF), m/z: 477.2 [M + H]^+^. Found, %: C 75.51; H 5.16; N 6.00. C_30_H_24_N_2_O_4_. Calculated, %: C 75.62; H 5.08; N 5.88.

**1′-(hydroxymethyl)-4′-methyl-6a′,6b′,7′,8′,9′,11a′-hexahydro-6′*H*-spiro[indoline-3,11′-pyrido [4′,3′:5,6]pyrano [3,4-*a*]pyrrolizine]-2,6′-dione 10**

A 25-mL round-bottom flask equipped with a reflux condenser was charged with 7-azacoumarin-3-carboxylic acid **1a** (0.30 g, 1.277 mmol) and absolute ethanol (5 mL). The mixture was heated to reflux with stirring, and L-proline **2** (0.18 g, 1.530 mmol) and isatin **9a** (0.23 g, 1.530 mmol) were added in one portion as solids. The reaction mixture was refluxed with stirring for 1 h. After cooling to room temperature, the solvent was removed under reduced pressure. The residue was recrystallized from DMF (5 mL) to afford compound **10** (0.31 g, 61%) as a white solid. M.p. 211–213 °C. IR(KBr), ν, cm^−1^: 558, 592, 614, 667, 701, 742, 759, 1011, 1047, 1020, 1203, 1238, 1266, 1340, 1385, 1443, 1475, 1598, 1621, 1715, 1737, 2927, 2954, 3450. ^1^H NMR (600 MHz, DMSO-*d*_6_), δ ppm: 1.65–1.70 (1H, m, CH_2_), 1.77–1.84 (1H, m, CH_2_), 1.90–1.94 (1H, m, CH_2_), 2.32 (1H, dd, *J* = 18.9, 7.4 Hz, CH), 2.37 (3H, s, CH_3_), 2.68 (1H, t, *J* = 8.0 Hz, CH), 3.11 (1H, dd, *J* = 14.4, 4.8 Hz, CH), 3.15–3.22 (1H, m, CH), 3.38 (1H, d, *J* = 10.4 Hz, CH), 4.02 (1H, dd, *J* = 13.0, 4.5 Hz, CH_2_OH), 4.35 (1H, t, *J* = 7.8 Hz, CH), 4.39 (1H, d, *J* = 10.8 Hz, CH_2_OH), 5.04 (1H, br.s, CH_2_OH), 6.70 (1H, d, *J* =7.7 Hz, Ph), 7.09 (1H, t, *J* = 7.5 Hz, Ph), 7.31 (1H, t, *J* = 7.8 Hz, Ph), 7.70 (1H, d, *J* = 7.5 Hz, Ph), 8.04 (1H, s, CH_arom_), 10.11 (1H, s, NH). ^13^C NMR (151 MHz, DMSO-*d*_6_), δ, ppm: 19.40, 26.53, 32.82, 41.05, 46.65, 50.79, 58.37, 67.99, 77.07, 110.32, 121.85, 123.87, 125.82, 128.41, 130.63, 134.98, 143.13, 143.45, 145.01, 146.12, 167.56, 177.57. MS (MALDI-TOF), m/z: 392.1 [M + H]^+^. Found, %: C 67.64; H 5.52; N 10.87. C_22_H_21_N_3_O_4_. Calculated, %: C 67.51; H 5.41; N 10.74.

**Methyl 4′-(3-hydroxy-5-(hydroxymethyl)-2-methylpyridin-4-yl)-1′-methyl-2-oxospiro[indoline-3,2′-pyrrolidine]-3′-carboxylate 12a**

A 25-mL round-bottom flask equipped with a reflux condenser was charged with 7-azacoumarin-3-carboxylic acid **1a** (0.30 g, 1.277 mmol), sarcosine **11** (0.14 g, 1.573 mmol), isatin **9a** (0.23 g, 1.573 mmol), and absolute methanol (3 mL). The reaction mixture was heated under reflux with stirring for 4 h. After cooling to room temperature, the precipitated solid was collected by vacuum filtration, washed with absolute methanol (2 × 5 mL) and diethyl ether (2 × 5 mL), and dried in vacuo to afford compound **12a** (0.24 g, 52%) as a light green solid. The product was sufficiently pure and was used without further purification. M.p. 216 °C. IR(KBr), ν, cm^−1^: 552, 598, 633, 670, 701, 746, 783, 894, 933, 965, 991, 1023, 1066, 1103, 1126, 1189, 1218, 1275, 1294, 1336, 1359, 1434, 1474, 1552, 1603, 1625, 1697, 1734, 2742, 2877, 2919, 2949, 3090. ^1^H NMR (600 MHz, DMSO-*d*_6_), δ ppm: 2.00 (3H, s, CH_3_), 2.30 (3H, s, CH_3_), 3.15 (3H, s, OCH_3_), 3.32 (1H, dd, *J* = 9.9, 7.7 Hz, CH_2_), 3.47 (1H, dd, *J* = 9.9, 7.6 Hz, CH_2_), 4.23 (1H, d, *J* = 12.1 Hz, CH), 4.43–4.51 (2H, m, CH_2_OH + CH), 4.63 (1H, dd, *J* = 12.6, 6.3 Hz, CH_2_OH), 5.10 (1H, dd, *J* = 6.2, 4.7 Hz, CH_2_OH), 6.89 (1H, dd, *J* = 7.7, 0.9 Hz, Ph), 7.07 (1H, t, *J* =7.5, 1.0 Hz, Ph), 7.29 (1H, td, *J* = 7.7, 1.2 Hz, Ph), 7.48 (1H, d, *J* = 7.4 Hz, Ph), 7.81 (1H, s, CH_arom_), 10.31 (1H, s, NH), 10.87 (1H, s, OH). ^13^C NMR (151 MHz, DMSO-*d*_6_), δ, ppm: 20.23, 34.18, 36.32, 51.57, 55.37, 56.39, 60.73, 73.25, 110.56, 122.84, 125.14, 129.34, 130.11, 130.35, 135.43, 139.28, 142.99, 147.57, 150.49, 169.64, 179.46. MS (MALDI-TOF), m/z: 398.1 [M + H]^+^. Found, %: C 63.60; H 5.94; N 10.49. C_21_H_23_N_3_O_5_. Calculated, %: C 63.47; H 5.83; N 10.57.

**Methyl 4′-(3-hydroxy-5-(hydroxymethyl)-2-methylpyridin-4-yl)-1′,5-dimethyl-2-oxospiro[indoline-3,2′-pyrrolidine]-3′-carboxylate 12b**

A 25-mL round-bottom flask equipped with a reflux condenser was charged with 7-azacoumarin-3-carboxylic acid **1a** (0.30 g, 1.277 mmol), sarcosine **11** (0.14 g, 1.573 mmol), 5-methylisatin **9b** (0.25 g, 1.573 mmol), and absolute methanol (3 mL). The reaction mixture was heated under reflux with stirring for 4 h. After cooling to room temperature, the precipitated solid was collected by vacuum filtration, washed with absolute methanol (2 × 5 mL) and diethyl ether (2 × 5 mL), and dried in vacuo to afford compound **12b** (0.35 g, 67%) as a light yellow solid. The product was sufficiently pure and was used without further purification. M.p. 200–203 °C. IR(KBr), ν, cm^−1^: 552, 598, 613, 677, 739, 771, 821, 898, 935, 991, 1025, 1075, 1114, 1153, 1196, 1220, 1288, 1336, 1353, 1435, 1495, 1627, 1698, 1733, 2750, 2868, 2919, 2950, 3139, 3433. ^1^H NMR (400 MHz, DMSO-*d*_6_), δ ppm: 2.00 (3H, s, CH_3_), 2.29 (3H, s, CH_3_), 2.30 (3H, s, CH_3_), 3.14 (3H, s, OCH_3_), 3.28–3.32 (1H, m, CH_2_), 3.48 (1H, dd, *J* = 9.7, 7.7 Hz, CH), 4.19 (1H, d, *J* = 12.1 Hz, CH), 4.44–4.51 (2H, m, CH_2_OH + CH), 4.62 (1H, dd, *J* = 12.6, 6.0 Hz, CH_2_OH), 5.06 (1H, dd, *J* = 6.1, 4.7 Hz, CH_2_OH), 6.78 (1H, d, *J* = 7.8 Hz, Ph), 7.09 (1H, d, *J* = 7.6 Hz, Ph), 7.32 (1H, d, *J* = 1.7 Hz, Ph), 7.81 (1H, s, CH_arom_), 10.28 (1H, s, NH), 10.74 (1H, s, OH). ^13^C NMR (101 MHz, DMSO-*d*_6_), δ, ppm: 20.21, 21.20, 34.19, 36.30, 51.52, 55.40, 56.31, 60.75, 73.34, 110.27, 125.75, 129.43, 130.29, 130.33, 131.83, 135.39, 139.28, 140.53, 147.58, 150.49, 169.63, 179.46. MS (MALDI-TOF), m/z: 412.1 [M + H]^+^. Found, %: C 64.41; H 6.30; N 10.17. C_22_H_25_N_3_O_5_. Calculated, %: C 64.22; H 6.12; N 10.21.

**9′-(hydroxymethyl)-2′,6′-dimethyl-1′,2′,3a′,9b′-tetrahydro-4′*H*-spiro[indoline-3,3′-pyrrolo [3′,4′:4,5]pyrano [2,3-*c*]pyridine]-2,4′-dione 13a**

A 25-mL round-bottom flask equipped with a reflux condenser was charged with 7-azacoumarin-3-carboxylic acid **1a** (0.30 g, 1.277 mmol), sarcosine (0.12 g, 1.348 mmol), isatin **9a** (0.22 g, 1.348 mmol), and acetonitrile (5 mL). The reaction mixture was heated under reflux with stirring for 10 h. After cooling to room temperature, the solvent was removed under reduced pressure. The residue was purified by column chromatography on silica gel (eluent: hexane/acetone, 1:1) to afford compound **13a** (0.28 g, 61%) as a light yellow solid. M.p. 145–148 °C. IR(KBr), ν, cm^−1^: 472, 491, 537, 619, 679, 753, 912, 961, 1017, 1082, 1109, 1178, 1203, 1281, 1330, 1357, 1405, 1471, 1621, 1715, 2850, 2945, 3395. ^1^H NMR (600 MHz, DMSO-*d*_6_), δ ppm: 1.99 (3H, s, CH_3_), 2.39 (3H, s, CH_3_), 3.17 (1H, t, *J* = 9.1 Hz, CH_2_), 3.70 (1H, t, *J* = 8.3 Hz, CH_2_), 4.09 (1H, d, *J* = 12.2 Hz, CH), 4.16 (1H, ddd, *J* = 12.2, 9.4, 7.8 Hz, CH), 4.52 (1H, dd, *J* = 13.5, 5.5 Hz, CH_2_OH), 4.63 (1H, dd, *J* = 13.5, 5.4 Hz, CH_2_OH), 5.36 (1H, t, *J* = 5.4 Hz, CH_2_OH), 6.83 (1H, d, *J* = 7.7 Hz, Ph), 7.06 (1H, td, *J* = 7.5, 1.0 Hz, Ph), 7.27 (1H, td, *J* =7.7, 1.1 Hz, Ph), 7.36 (1H, d, *J* = 7.3 Hz, Ph), 8.23 (1H, s, CH_arom_), 10.41 (1H, s, NH). ^13^C NMR (151 MHz, DMSO-*d*_6_), δ, ppm: 18.87, 30.63, 33.81, 33.92, 48.87, 58.66, 58.75, 74.77, 109.65, 122.15, 124.14, 127.40, 128.88, 129.46, 133.12, 143.24, 143.48, 144.33, 144.92, 165.44, 177.16. MS (MALDI-TOF), m/z: 366.2 [M + H]^+^. Found, %: C 65.59; H 5.11; N 11.47. C_20_H_19_N_3_O_4_. Calculated, %: C 65.74; H 5.24; N 11.50.

**9′-(hydroxymethyl)-2′,5,6′-trimethyl-1′,2′,3a′,9b′-tetrahydro-4′*H*-spiro[indoline-3,3′-pyrrolo [3′,4′:4,5]pyrano [2,3-*c*]pyridine]-2,4′-dione 13b**

Similar to the previous one, from **1a** (0.30 g, 1.277 mmol), sarcosine **11** (0.12 g, 1.348 mmol) and 5-methylisatin **9b** (0.22 g, 1.348 mmol). Light yellow solid, yield 0.25 g (52%). M.p. 170–173 °C. IR(KBr), ν, cm^−1^: 470, 539, 596, 627, 689, 749, 914, 946, 1016, 1082, 1196, 1282, 1330, 1405, 1445, 1473, 1495, 1627, 1709, 1760, 2852, 2922, 3256, 3388. ^1^H NMR (600 MHz, DMSO-*d*_6_), δ ppm: 1.99 (3H, s, CH_3_), 2.30 (3H, s, CH_3_), 2.39 (3H, s, CH_3_), 3.16 (1H, t, *J* = 9.1 Hz, CH_2_), 3.68 (1H, t, *J* = 8.3 Hz, CH_2_), 4.06 (1H, d, *J* = 12.2 Hz, CH), 4.15 (1H, ddd, *J* = 12.2, 9.5, 7.9 Hz, CH), 4.52 (1H, dd, *J* = 13.5, 5.4 Hz, CH_2_OH), 4.62 (1H, dd, *J* = 13.5, 5.4 Hz, CH_2_OH), 5.36 (1H, t, *J* = 5.4 Hz, CH_2_OH), 6.72 (1H, d, *J* =7.9 Hz, Ph), 7.07–7.08 (1H, m, Ph), 7.18 (1H, s, Ph), 8.23 (1H, s, CH_arom_), 10.31 (1H, s, NH). ^13^C NMR (151 MHz, DMSO-*d*_6_), δ, ppm: 18.86, 20.69, 30.63, 33.83, 33.96, 48.86, 58.71, 58.76, 74.86, 109.39, 124.73, 127.43, 128.93, 129.69, 131.00, 133.11, 140.78, 143.46, 144.34, 144.91, 165.43, 177.11. MS (MALDI-TOF), m/z: 380.0 [M + H]^+^. Found, %: C 66.54; H 5.70; N 11.13. C_21_H_21_N_3_O_4_. Calculated, %: C 66.48; H 5.58; N 11.08.

**5-chloro-9′-(hydroxymethyl)-2′,6′-dimethyl-1′,2′,3a′,9b′-tetrahydro-4′*H*-spiro[indoline-3,3′-pyrrolo [3′,4′:4,5]pyrano [2,3-*c*]pyridine]-2,4′-dione 13c**

Similar to the previous one, from **1a** (0.30 g, 1.277 mmol), sarcosine **11** (0.12 g, 1.348 mmol) and 5-chloroisatin **9c** (0.25 g, 1.348 mmol). Light yellow solid, yield 0.23 g (45%). M.p. 183–186 °C. IR(KBr), ν, cm^−1^: 470, 559, 628, 683, 706, 745, 793, 819, 890, 975, 1016, 1081, 1120, 1176, 1204, 1270, 1331, 1357, 1405, 1440, 1479, 1621, 1716, 2849, 2952, 3223. ^1^H NMR (600 MHz, DMSO-*d*_6_), δ ppm: 2.01 (3H, s, CH_3_), 2.39 (3H, s, CH_3_), 3.14–3.17 (1H, m, CH_2_); 3.69–3.72 (1H, m, CH_2_), 4.16–4.20 (2H, m, 2CH), 4.52 (1H, dd, *J* = 13.5, 5.4 Hz, CH_2_OH), 4.62 (1H, dd, *J* =13.5, 5.4 Hz, CH_2_OH), 5.37 (1H, t, *J* = 5.4 Hz, CH_2_OH), 6.86 (1H, d, *J* = 8.3 Hz, Ph), 7.33 (1H, dd, *J* = 8.3, 2.2 Hz, Ph), 7.46 (1H, d, *J* = 2.2 Hz, Ph), 8.24 (1H, s, CH_arom_), 10.58 (1H, s, NH). ^13^C NMR (151 MHz, DMSO-*d*_6_), δ, ppm: 18.87, 30.62, 33.72, 33.98, 48.87, 58.78, 74.84, 111.17, 124.50, 126.24, 127.26, 129.41, 131.01, 133.12, 142.12, 143.59, 144.29, 145.01, 165.44, 176.93. MS (MALDI-TOF), m/z: 401.1 [M + H]^+^. Found, %: C 60.17; H 4.68; Cl 8.81; N 10.47. C_20_H_18_ClN_3_O_4_. Calculated, %: C 60.08; H 4.54; Cl 8.87; N 10.51.

**5-bromo-9′-(hydroxymethyl)-2′,6′-dimethyl-1′,2′,3a′,9b′-tetrahydro-4′*H*-spiro[indoline-3,3′-pyrrolo [3′,4′:4,5]pyrano [2,3-*c*]pyridine]-2,4′-dione 13d**

Similar to the previous one, from **1a** (0.30 g, 1.277 mmol), sarcosine **11** (0.12 g, 1.348 mmol) and 5-bromoisatin **9d** (0.30 g, 1.348 mmol). Light yellow solid, yield 0.20 g (36%). M.p. 164–166 °C. IR(KBr), ν, cm^−1^: 471, 537, 626, 691, 748, 817, 888, 913, 972, 1017, 1081, 1121, 1175, 1203, 1331, 1358, 1405, 1438, 1473, 1618, 1717, 2851, 2926, 3245, 3395. ^1^H NMR (600 MHz, DMSO-*d*_6_), δ ppm: 2.01 (3H, s, CH_3_), 2.39 (3H, s, CH_3_), 3.15 (1H, t, *J* = 8.8 Hz, CH_2_), 3.69–3.72 (1H, m, CH_2_), 4.16–4.20 (2H, m, 2CH), 4.52 (1H, dd, *J* = 13.6, 5.3 Hz, CH_2_OH), 4.62 (1H, dd, *J* =13.6, 5.3 Hz, CH_2_OH), 5.36 (1H, t, *J* = 5.4 Hz, CH_2_OH), 6.81 (1H, d, *J* = 8.2 Hz, Ph), 7.46 (1H, ddd, *J* = 8.4, 2.2, 0.9 Hz, Ph), 7.57 (1H, d, *J* = 2.1 Hz, Ph), 8.24 (1H, s, CH_arom_), 10.58 (1H, s, NH). ^13^C NMR (151 MHz, DMSO-*d*_6_), δ, ppm: 18.86, 29.58, 33.74, 33.99, 48.90, 58.79, 58.81, 74.81, 111.69, 113.92, 127.24, 131.38, 132.24, 133.11, 142.54, 143.61, 144.29, 145.04, 165.45, 176.81. MS (MALDI-TOF), m/z: 445.0 [M + H]^+^. Found, %: C 54.21; H 4.13; N 9.51. C_20_H_18_BrN_3_O_4_. Calculated, %: C 54.07; H 4.08; N 9.46.

**3-hydroxy-6a,6b,7,8,9,11a-hexahydro-6*H*-spiro[chromeno [3,4-*a*]pyrrolizine-11,3′-indoline]-2′,6-dione 17a**

A 25-mL round-bottom flask equipped with a reflux condenser was charged with coumarin-3-carboxylic acid **16a** (0.25 g, 1.214 mmol) and absolute ethanol (5 mL). The mixture was heated to reflux with stirring, and L-proline **2** (0.14 g, 1.217 mmol) and isatin **9a** (0.18 g, 1.214 mmol) were added in one portion as solids. The reaction mixture was refluxed with stirring for 1 h. After cooling to room temperature, the precipitated solid was collected by vacuum filtration, washed with absolute ethanol (2 × 5 mL) and diethyl ether (2 × 5 mL), and dried in vacuo to afford compound **17a** (0.36 g, 82%) as a grey solid. The product was sufficiently pure and was used without further purification. M.p. 237 °C. IR (KBr), ν, cm^−1^: 431, 451, 466, 500, 545, 557, 574, 616, 643, 665, 729, 756, 812, 826, 854, 909, 928, 980, 1025, 1048, 1120, 1157, 1181, 1207, 1299, 1358, 1461, 1511, 1620, 1702, 1757, 2416, 2664, 2690, 2811, 2877, 2979, 3059, 3128, 3367.^1^H NMR (400 MHz, DMSO-*d*_6_), δ ppm: 1.59–1.87 (3H, m, CH), 2.31 (1H, dt, *J* = 12.6, 7.6 Hz, CH), 2.58 (1H, t, *J* = 7.1 Hz, CH), 3.09–3.15 (1H, m, CH), 3.26 (1H, dd, *J* = 11.2, 3.3 Hz, CH), 3.97 (1H, d, *J* = 11.3 Hz, CH), 4.34 (1H, ddd, *J* = 8.1, 6.4, 3.2 Hz, CH), 6.06 (1H, d, *J* = 8.4 Hz, Ph), 6.17 (1H, dd, *J* = 8.4, 2.4 Hz, Ph), 6.31 (1H, d, *J* = 2.4 Hz, Ph), 6.70 (1H, d, *J* = 7.7 Hz, Ph), 7.07 (1H, t, *J* = 7.5 Hz, Ph), 7.27 (1H, t, *J* = 7.6 Hz, Ph), 7.67 (1H, d, *J* = 7.5 Hz, Ph), 9.58 (1H, s, OH), 10.00 (1H, s, NH). ^13^C NMR (101 MHz, DMSO-*d*_6_), δ, ppm: 27.01, 33.80, 43.00, 48.06, 51.30, 67.90, 77.15, 103.96, 108.89, 110.64, 112.00, 122.49, 127.34, 127.52, 128.97, 130.73, 144.38, 152.53, 158.55, 168.72, 178.67. MS (MALDI-TOF), m/z: 363.1 [M + H]^+^. Found, %: C 69.80; H 5.12; N 7.52. C_21_H_18_N_2_O_4_. Calculated, %: C 69.60; H 5.01; N 7.73.

**3-hydroxy-5′-methyl-6a,6b,7,8,9,11a-hexahydro-6*H*-spiro[chromeno [3,4-*a*]pyrrolizine-11,3′-indoline]-2′,6-dione 17b**

Similar to the previous one, from **16a** (0.25 g, 1.214 mmol), L-proline **2** (0.14 g, 1.217 mmol) and 5-methylisatin **9b** (0.195 g, 1.214 mmol). Grey solid, yield 0.38 g (83%). M.p. 238 °C. IR (KBr), ν, cm^−1^: 430, 445, 469, 505, 545, 564, 584, 601, 631, 666, 679, 705, 752, 820, 848, 926, 983, 1005, 1045, 1076, 1119, 1156, 1176, 1209, 1300, 1349, 1493, 1510, 1624, 1698, 1750, 2341, 2415, 2693, 2811, 2876, 2979, 3054, 3349. ^1^H NMR (400 MHz, DMSO-*d*_6_), δ ppm: 1.58–1.68 (1H, m, CH), 1.74–1.89 (2H, m, CH), 2.27–2.31 (1H, m, CH), 2.33 (3H, s, CH_3_), 2.58 (1H, t, *J* = 7.2 Hz, CH), 3.12 (1H, ddd, *J* = 10.7, 8.5, 5.1 Hz, CH), 3.25 (1H, dd, *J* = 11.2, 3.3 Hz, CH), 3.94 (1H, d, *J* = 11.3 Hz, CH), 4.33 (1H, ddd, *J* = 7.8, 6.4, 3.2 Hz, CH), 6.08 (1H, d, *J* = 8.4 Hz, Ph), 6.18 (1H, dd, *J* = 8.4, 2.4 Hz, Ph), 6.30 (1H, d, *J* = 2.4 Hz, Ph), 6.59 (1H, d, *J* = 7.9 Hz, Ph), 7.08 (1H, dd, *J* = 8.0, 1.6 Hz, Ph), 7.50 (1H, s, Ph), 9.56 (1H, s, OH), 9.88 (1H, s, NH). ^13^C NMR (101 MHz, DMSO-*d*_6_), δ, ppm: 21.89, 26.99, 33.80, 43.03, 48.04, 51.30, 67.91, 77.26, 103.92, 108.98, 110.32, 112.01, 127.41, 128.09, 129.02, 130.94, 131.31, 141.91, 152.50, 158.49, 168.74, 178.65. MS (MALDI-TOF), m/z: 377.1 [M + H]^+^. Found, %: C 70.15; H 5.40; N 7.41. C_22_H_20_N_2_O_4_. Calculated, %: C 70.20; H 5.36; N 7.44.

**5′-chloro-3-hydroxy-6a,6b,7,8,9,11a-hexahydro-6*H*-spiro[chromeno [3,4-*a*]pyrrolizine-11,3′-indoline]-2′,6-dione 17c**

Similar to the previous one, from **16a** (0.21 g, 1.019 mmol), L-proline **2** (0.12 g, 1.043 mmol) and 5-chloroisatin **9c** (0.19 g, 1.043 mmol). Light yellow solid, yield 0.27 g (52%). M.p. 239 °C. IR (KBr), ν, cm^−1^: 431, 454, 469, 505, 564, 626, 680, 714, 736, 820, 860, 927, 985, 1050, 1076, 1119, 1158, 1174, 1206, 1300, 1349, 1463, 1512, 1620, 1733, 1749, 2804, 2973, 3087, 3373. ^1^H NMR (500 MHz, DMSO-*d*_6_), δ ppm: 1.60–1.70 (1H, m, CH), 1.76–1.83 (1H, m, CH), 1.87–1.92 (1H, m, CH), 2.34 (1H, dt, *J* = 13.9, 7.9 Hz, CH), 2.60 (1H, t, *J* = 6.9 Hz, CH), 3.15 (1H, ddd, *J* = 10.4, 8.3, 5.4 Hz, CH), 3.29 (1H, dd, *J* = 11.2, 3.1 Hz, CH), 4.08 (1H, d, *J* = 6.9 Hz, CH), 4.36 (1H, td, *J* = 7.4, 3.1 Hz, CH), 6.16 (1H, d, *J* = 8.4 Hz, Ph), 6.26 (1H, dd, *J* = 8.4, 2.3 Hz, Ph), 6.35 (1H, d, *J* = 2.3 Hz, Ph), 6.74 (1H, d, *J* = 8.3 Hz, Ph), 7.36 (1H, dd, *J* = 8.3, 1.9 Hz, Ph), 7.85 (1H, d, *J* = 1.8 Hz, Ph), 9.62 (1H, s, OH), 10.18 (1H, s, NH). ^13^C NMR (126 MHz, DMSO-d6), δ, ppm: 27.04, 33.79, 42.95, 47.94, 51.22, 68.03, 77.34, 104.06, 108.65, 112.04, 112.18, 126.62, 127.73, 128.98, 129.38, 130.70, 143.32, 152.54, 158.66, 168.62, 178.42. MS (MALDI-TOF), m/z: 419.0 [M + Na]^+^. Found, %: C 63.37; H 4.37; Cl 8.87; N 6.98. C_21_H_17_N_2_O_4_Cl. Calculated, %: C 63.56; H 4.32; Cl 8.93, N 7.06.

**5′-bromo-3-hydroxy-6a,6b,7,8,9,11a-hexahydro-6*H*-spiro[chromeno [3,4-*a*]pyrrolizine-11,3′-indoline]-2′,6-dione 17d**

Similar to the previous one, from **16a** (0.21 g, 1.019 mmol), L-proline **2** (0.12 g, 1.043 mmol) and 5-bromoisatin **9d** (0.23 g, 1.043 mmol). Grey solid, yield 0.21 g (47%). M.p. 233 °C. IR (KBr), ν, cm^−1^: 505, 543, 582, 603, 624, 665, 679, 707, 757, 817, 862, 984, 1049, 1077, 1119, 1157, 1207, 1299, 1348 1463, 1511, 1620, 1703, 1750, 2972, 3042, 3087, 3373. ^1^H NMR (500 MHz, DMSO-*d*_6_), δ ppm: 1.61–1.69 (1H, m, CH), 1.76–1.83 (1H, m, CH), 1.86–1.93 (1H, m, CH), 2.34 (1H, dt, *J* = 13.9, 8.0 Hz, CH), 2.60 (1H, t, *J* = 6.6 Hz, CH), 3.15 (1H, br.s, CH), 3.27–3.30 (1H, m, CH), 4.09 (1H, d, *J* = 11.1 Hz, CH), 4.35 (1H, t, *J* = 8.5 Hz, CH), 6.16 (1H, d, *J* = 8.4 Hz, Ph), 6.26 (1H, d, *J* = 8.3 Hz, Ph), 6.34 (1H, s, Ph), 6.69 (1H, d, *J* = 8.2 Hz, Ph), 7.49 (1H, d, *J* = 8.3 Hz, Ph), 7.97 (1H, s, Ph), 9.62 (1H, s, OH), 10.19 (1H, s, NH). ^13^C NMR (126 MHz, DMSO-*d*_6_), δ, ppm: 27.02, 33.77, 42.94, 47.90, 51.21, 68.02, 77.29, 104.05, 108.65, 112.16, 112.54, 114.23, 128.94, 129.76, 130.39, 133.54, 143.71, 152.52, 158.64, 168.59, 178.29. MS (MALDI-TOF), m/z: 465.0 [M + Na]^+^. Found, %: C 57.16; H 4.02; N 6.17; Br 18.38. C_21_H_17_N_2_O_4_Br. Calculated, %: C 57.14; H 3.85; N 6.35; Br 18.14.

**2-chloro-3-hydroxy-6a,6b,7,8,9,11a-hexahydro-6*H*-spiro[chromeno [3,4-*a*]pyrrolizine-11,3′-indoline]-2′,6-dione 18**

A 25-mL round-bottom flask equipped with a reflux condenser was charged with **16b** (0.11 g, 0.457 mmol), L-proline **2** (0.052 g, 0.457 mmol), isatin **9a** (0.067 g, 0.457 mmol), and absolute ethanol (5 mL). The mixture was refluxed with stirring for 1 h. After cooling, the solvent was removed under reduced pressure, and the residue was recrystallized from ethanol (3 mL) to afford compound **18** (0.17 g, 94%) as a white solid. M.p. 175 °C. IR (KBr), ν, cm^−1^: 492, 572, 620, 652, 676, 757, 834, 884, 931, 1027, 1155, 1216, 1329, 1357, 1431, 1472, 1512, 1620, 1718, 2874, 2971, 3216.^1^H NMR (500 MHz, DMSO-*d*_6_), δ ppm: 1.62–1.68 (1H, m, CH), 1.77–1.83 (1H, m, CH), 1.86–1.90 (1H, m, CH), 2.31–2.37 (1H, m, CH), 2.61 (1H, t, *J* = 7.3 Hz, CH), 3.13 (1H, ddd, *J* = 10.7, 8.4, 5.1 Hz, CH), 3.30 (1H, dd, *J* = 11.2, 3.3 Hz, CH), 4.02 (1H, d, *J* = 11.3 Hz, CH), 4.36 (1H, ddd, *J* = 8.0, 6.5, 3.3 Hz, CH), 6.13 (1H, s, Ph), 6.55 (1H, s, Ph), 6.75 (1H, dd, *J* = 7.8, 1.0 Hz, Ph), 7.12 (1H, td, *J* = 7.6, 1.1 Hz, Ph), 7.33 (1H, td, *J* = 7.7, 1.2 Hz, Ph), 7.71 (1H, d, *J* = 7.4 Hz, Ph), 10.07 (1H, s, NH). ^13^C NMR (126 MHz, DMSO-*d*_6_), δ, ppm: 26.45, 33.22, 47.17, 50.74, 67.52, 76.69, 104.71, 109.91, 110.22, 114.83, 122.11, 126.43, 127.07, 128.20, 130.40, 143.80, 150.51, 153.42, 167.74, 177.99. MS (MALDI-TOF), m/z: 398.1 [M + H]^+^. Found, %: C 63.74; H 4.15; Cl 8.72; N 6.83. C_21_H_17_ClN_2_O_4_. Calculated, %: C 63.56; H 4.32; Cl 8.93; N 7.06.

**3′-hydroxy-6a′,6b′,7′,8′,9′,11a′-hexahydro-1*H*,6′*H*-spiro[aceanthrylene-2,11′-chromeno [3,4-*a*]pyrrolizine]-1,6′-dione 19**

A 25-mL round-bottom flask equipped with a reflux condenser was charged with coumarin-3-carboxylic acid **16a** (0.25 g, 1.214 mmol) and absolute ethanol (5 mL). The mixture was heated to reflux with stirring, and L-proline **2** (0.14 g, 1.217 mmol) and aceanthrenequinone **7** (0.28 g, 1.217 mmol) were added in one portion as solids. The reaction mixture was refluxed with stirring for 1 h. After cooling to room temperature, the precipitated solid was collected by vacuum filtration, washed with absolute ethanol (2 × 5 mL) and diethyl ether (2 × 5 mL), and dried in vacuo to afford compound **19** (0.39 g, 71%) as a dark brown solid. The product was sufficiently pure and was used without further purification. M.p. 169 °C. IR (KBr), ν, cm^−1^: 427, 457, 469, 485, 502, 526, 559, 617, 637, 666, 695, 712, 744, 775, 818, 853, 897, 927, 979, 1045, 1073, 1092, 1118, 1157, 1179, 1206, 1232, 1280, 1306, 1357, 1381, 1396, 1427, 1460, 1512, 1532, 1576. 1600, 1625, 1696, 1753, 1922, 2610, 2703, 2807, 2872, 2964, 3048. ^1^H NMR (400 MHz, DMSO-*d*_6_), δ ppm: 1.59–1.65 (1H, m, CH), 1.85–1.93 (2H, m, CH), 2.39 (1H, dt, *J* = 11.3, 7.6 Hz, CH), 2.50–2.52 (1H, m, CH), 3.21–3.26 (1H, m, CH), 3.44–3.47 (1H, m, CH), 4.31 (1H, br.s, CH), 4.36 (1H, d, *J* = 11.4 Hz, CH), 5.90 (1H, dd, *J* = 8.4, 2.4 Hz, Ph), 6.16 (1H, d, *J* = 8.4 Hz, Ph), 6.25 (1H, d, *J* = 2.4 Hz, Ph), 7.67 (2H, dt, *J* = 34.5, 7.7 Hz, Ph), 7.82 (1H, dd, *J* = 8.4, 7.0 Hz, Ph), 8.10 (1H, d, *J* = 6.7 Hz, Ph), 8.18 (1H, d, *J* = 8.7 Hz, Ph), 8.25 (1H, d, *J* = 8.5 Hz, Ph), 8.69 (1H, d, *J* = 8.5 Hz, Ph), 8.93 (1H, s, Ph), 9.39 (1H, s, OH). ^13^C NMR (101 MHz, DMSO-*d*_6_), δ, ppm: 26.58, 33.46, 41.68, 48.09, 51.64, 67.89, 80.01, 103.46, 108.47, 111.48, 123.52, 123.79, 124.29, 126.52, 127.10, 127.80, 128.27, 128.30, 130.11, 130.17, 133.32, 133.42, 136.54, 144.85, 151.91, 157.86, 168.39, 203.08. MS (MALDI-TOF), m/z: 448.1 [M + H]^+^. Found, %: C 77.98; H 4.85; N 3.24. C_29_H_21_NO_4_. Calculated, %: C 77.84; H 4.73; N 3.13.

**7-hydroxy-2-methyl-1,2,3a,9b-tetrahydro-4*H*-spiro[chromeno [3,4-*c*]pyrrole-3,3′-indoline]-2′,4-dione 20a**

A 25-mL round-bottom flask equipped with a reflux condenser was charged with coumarin-3-carboxylic acid **16a** (0.25 g, 1.214 mmol) and absolute ethanol (5 mL). The mixture was heated to reflux with stirring, and sarcosine **11** (0.11 g, 1.236 mmol) and isatin **9a** (0.18 g, 1.224 mmol) were added in one portion as solids. The reaction mixture was refluxed with stirring for 1 h. After cooling to room temperature, the solvent was removed under reduced pressure. The solid residue was recrystallized from a mixture of ethanol and diethyl ether (1:1, 3 mL) to afford compound **20a** (0.33 g, 81%) as a white solid. M.p. 252 °C. IR (KBr), ν, cm^−1^: 433, 460, 490, 514, 562, 606, 621, 648, 681, 716, 742, 753, 816, 869, 945, 969, 1017, 1109, 1169, 1212, 1264, 1326, 1361, 1384, 1444, 1469, 1522, 1597, 1620, 1719, 2508, 2623, 2703, 2803, 2867, 2945, 3063, 3410, 3511. ^1^H NMR (400 MHz, DMSO-*d*_6_), δ ppm: 1.97 (3H, s, CH_3_), 3.17 (1H, t, *J* = 8.8 Hz, CH), 3.55 (1H, t, *J* = 8.1 Hz, CH), 3.89 (1H, dt, *J* = 12.4, 8.4 Hz, CH), 4.05 (1H, d, *J* = 12.3 Hz, CH), 6.38 (1H, d, *J* = 2.4 Hz, CH), 6.60 (1H, dd, *J* = 8.3, 2.4 Hz, Ph), 6.81 (1H, d, *J* = 7.1 Hz, Ph), 7.04 (1H, td, *J* = 7.5, 1.0 Hz, Ph), 7.09 (1H, d, *J* = 8.4 Hz, Ph), 7.25 (1H, td, *J* = 7.7, 1.3 Hz, Ph), 7.33 (1H, d, *J* = 7.0 Hz, Ph), 10.37 (1H, s, NH). ^13^C NMR (101 MHz, DMSO-*d*_6_), δ, ppm: 35.02, 35.90, 50.06, 61.54, 75.86, 103.76, 110.52, 113.13, 113.23, 123.02 125.15, 129.95, 130.29, 130.35, 144.18, 150.81, 158.07, 167.39, 178.29. MS (MALDI-TOF), m/z: 359.0 [M + Na]^+^. Found, %: C 67.89; H 4.75; N 8.27. C_19_H_16_N_2_O_4_. Calculated, %: C 67.85; H 4.80; N 8.33.

**7-hydroxy-2,5′-dimethyl-1,2,3a,9b-tetrahydro-4*H*-spiro[chromeno [3,4-*c*]pyrrole-3,3′-indoline]-2′,4-dione 20b**

A 25-mL round-bottom flask equipped with a reflux condenser was charged with coumarin-3-carboxylic acid **16a** (0.21 g, 1.019 mmol) and absolute ethanol (3 mL). The mixture was heated to reflux with stirring, and sarcosine **11** (0.095 g, 1.067 mmol) and 5-methylisatin **9b** (0.168 g, 1.043 mmol) were added in one portion as solids. The reaction mixture was refluxed with stirring for 1 h. After cooling to room temperature, the precipitated solid was collected by vacuum filtration, washed with absolute ethanol (2 × 5 mL) and diethyl ether (2 × 5 mL), and dried in vacuo to afford compound **20b** (0.23 g, 64%) as a white solid. The product was sufficiently pure and was used without further purification. M.p. 255 °C. IR (KBr), ν, cm^−1^: 431,457, 523, 563, 618, 662, 724, 818, 845, 961, 1014, 1082, 1112, 1168, 1188, 1232, 1254, 1331, 1438, 1454, 1472, 1495, 1517, 1588, 1629, 1722, 2957, 3032, 3335. ^1^H NMR (600 MHz, DMSO-*d*_6_), δ ppm: 1.97 (3H, s, CH_3_), 2.29 (3H, s, CH_3_), 3.16 (1H, t, *J* = 8.8 Hz, CH), 3.54 (1H, t, *J* = 8.1 Hz, CH), 3.87 (1H, dt, *J* = 12.4, 8.5 Hz, CH), 4.02 (1H, d, *J* = 12.3 Hz, CH), 6.37 (1H, d, *J* = 2.4 Hz, CH), 6.60 (1H, dd, *J* = 8.3, 2.4 Hz, Ph), 6.70 (1H, d, *J* = 7.8 Hz, Ph), 7.05 (1H, d, *J* = 7.8 Hz, Ph), 7.08 (1H, d, *J* = 8.4 Hz, Ph), 7.15 (1H, s, Ph), 9.66 (1H, br.s, OH), 10.25 (1H, s, NH). ^13^C NMR (151 MHz, DMSO-*d*_6_), δ, ppm: 21.21, 34.56, 35.41, 49.54, 61.09, 75.47, 103.26, 109.76, 112.69, 112.71, 125.24, 129.43, 129.88, 130.01, 131.34, 141.22, 150.31, 157.55, 166.85, 177.74. MS (MALDI-TOF), m/z: 351.0 [M + H]^+^. Found, %: C 68.32; H 5.09; N 7.87. C_20_H_18_N_2_O_4_. Calculated, %: C 68.56; H 5.18; N 8.00.

### 3.2. Biology

The purity of all final compounds was rigorously evaluated using a comprehensive set of physicochemical methods, namely: elemental analysis (C, H, N), MALDI-TOF mass spectrometry, and ^1^H and ^13^C NMR spectroscopy. The purity of the most biologically active compounds **6b**, **10**, and **17d** was further confirmed by quantitative 1D ^1^H NMR (qNMR) spectroscopy and was found to be ≥97%. The data of qNMR experiments provided in the Supplementary Materials (Table S4).

#### 3.2.1. Cell Lines and Materials

The following tumor cell cultures were used in the experiments: M-HeLa clone 11 (epithelioid cervical carcinoma, a subclone of HeLa designated M-HeLa); HuTu 80 (human duodenal adenocarcinoma); A 549 (human lung carcinoma) and the normal human embryonic lung fibroblast cell line Wi-38. All cell lines were obtained from the “Vertebrate Cell Culture Collection” of the General Research Center at the Institute of Cytology, Russian Academy of Sciences. Cells were cultured in Eagle’s MEM standard growth medium (EMEM) containing sodium pyruvate (without HEPES), supplied by Wuhan Servicebio Co., Ltd., Wuhan, China, and supplemented with 10% fetal bovine serum and 1% essential amino acids.

#### 3.2.2. Cytotoxicity Assay

The cytotoxic effect on cells was assessed using a colorimetric cell proliferation assay (MTT test). Cells were seeded into 96-well Nunc plates at a density of 5 × 10^3^ cells per well in 100 μL of culture medium and incubated in a CO_2_ incubator at 37 °C for 24 h. Following incubation, the culture medium was removed, and solutions of the test compounds, prepared directly in fresh nutrient medium, were added to the wells. To enhance solubility, DMSO was included at a final concentration not exceeding 1% in the experimental wells. Control samples (1% DMSO) were run in parallel with the experimental groups. Cell viability in these wells was 98–100%, indicating the correctness of the culture conditions and the reliability of the results obtained. To rule out compound interference with the MTT readout, compound-only controls (without cells) were included; no significant absorbance above background was observed. After 24 h of incubation with the compounds, the medium was aspirated, and 100 μL of serum-free medium containing MTT (0.5 mg/mL) was added to each well. The plates were then incubated for an additional 4 h at 37 °C. To dissolve the formazan crystals, 100 μL of DMSO was added to each well. Absorbance was measured at 540 nm using an Invitrologic microplate reader (Russia). All experiments were performed in triplicate for each compound. The IC_50_ values were determined by means of the online calculator MLA-Quest Graph™ IC50 Calculator (AAT Bioquest, Inc., Sunnyvale, CA, USA, 2026) https://www.aatbio.com/tools/ic50-calculator, accessed on 19 October 2026. Full dose–response curves are provided in the Supplementary Materials (Figures S55–S72). The fitting procedure used a four-parameter logistic (sigmoidal) dose–response model.

## 4. Conclusions

A series of new spiro-fused polycyclic compounds were prepared via cascade reactions of 7-azacoumarin-3-carboxylic acid and coumarin-3-carboxylic acid derivatives with azomethine ylides. For the conventional cycloaddition products (from 7-azacoumarin-3-carboxylic acid derivatives with sarcosine or L-proline and isatins; from coumarin-3-carboxylic acid derivatives with sarcosine or L-proline and isatins or aceanthrenequinone), the cascade reactions contain a 1,3-dipolar cycloaddition with a decarboxylation step, affording a single diastereomer in each case. Single-crystal X-ray diffraction was performed for the representative compounds **6a**, **10**, **12a**, **17a**, and **20a**: these products crystallize in centrosymmetric space groups and are therefore racemic. The reaction of 7-azacoumarin-3-carboxylic acids with the ylides derived from L-proline and ninhydrin or aceanthrenequinone provides the first example of a pyrrolidine-to-piperidine ring expansion (5→6); this transformation is currently demonstrated only for a limited set of substrates and should be regarded as a proof-of-concept rather than a general method. The factors governing the divergence between the conventional cycloaddition and ring-expansion pathways appear to be determined primarily by the electrophilicity and rigidity of the carbonyl component, as well as by the ability of the intermediate to stabilize the enol form required for the Stevens-type rearrangement. Transformation differs mechanistically from the conventional cycloaddition–decarboxylation; the proposed mechanism is presented as a working hypothesis. All reactions of the conventional cycloaddition type proceeded with high diastereoselectivity, affording a single detectable diastereomer in each case irrespective of the number of stereocenters formed.

The cytotoxic activity of selected compounds was evaluated in vitro. For compounds **10** and **17d**, the IC_50_ values against M-HeLa cells were numerically similar to that of the reference drug 5-fluorouracil, while compound **6b** gave an IC_50_ against HuTu80 cells numerically similar to that of 5-fluorouracil, and no measurable cytotoxicity toward the normal Wi-38 line at the highest concentration tested. Compound **10** gave a numerically lower IC_50_ value than 5-fluorouracil against A549 lung carcinoma cells, warranting further investigation within this series; however, these results should be interpreted as preliminary screening data and do not constitute evidence of therapeutic potential.

## Figures and Tables

**Table 1:** Cytotoxic effects of synthesized polycyclic compounds on cancer and normal human cell lines ^1,2^.

Compounds	IC_50_ (μM)			
	Cancer Cell Lines		Normal Cell Line	
	M-HeLa	HuTu80	A549	Wi-38
**6a**	>100	>100	-	59.2 ± 3.7
**6b**	>100	60.3 ± 5.8	>100	>100
**8**	>100	>100	>100	98.1 ± 1.2
**10**	59.2 ± 5.6	>100	84.7 ± 2.0	>100
**13a**	>100	>100	>100	>100
**13b**	>100	>100	>100	>100
**13c**	>100	>100	90 ± 9.8	>100
**13d**	>100	>100	>100	>100
**17a**	>100	>100	>100	>100
**17b**	>100	>100	>100	>100
**17c**	87.0 ± 7.3	>100	>100	>100
**17d**	66.9 ± 3.2	98.8 ± 10	99.6 ± 3.9	95.1 ± 1.5
**18**	>100	>100	>100	>100
**19**	>100	>100	>100	>100
**20a**	>100	>100	>100	>100
**20b**	>100	>100	>100	>100
5-fluorouracil	62.0 ± 4.7	65.2 ± 5.4	>100	61.9 ± 6.3
doxorubicin	3.5 ± 0.2	3.2 ± 0.2	2.0 ± 0.2	2.7 ± 0.2

^1^ Three independent experiments were carried out. ^2^ Compounds **12a**,**b** were not included in the cytotoxicity screening because, in DMSO solution, they convert to compounds **13a,b**, which precludes testing the pure ester forms.

## Data Availability

The data presented in this study are contained within the article or in Supplementary Materials or are available upon request from the corresponding author Alexey V. Trifonov.

## Supplementary Materials

The following supporting information can be downloaded at: https://www.mdpi.com/article/10.3390/ijms27219600/s1.
