# Supplementary material for: Divergent Reactions of Coumarin- and 7-Azacoumarin-3-Carboxylic Acids with Azomethine Ylides: Spirocyclization and Ring Expansion
Source: Int J Mol Sci. 2026 Oct 27;27(21):9600. doi: 10.3390/ijms27219600 (PMC13762739; doi:10.3390/ijms27219600)

Supplementary Materials

Divergent Reactions of Coumarin- and 7-Azacoumarin-3-Carboxylic Acids with Azomethine Ylides: Spirocyclization and Ring Expansion

Alexey V. Trifonov[1*], Nurbol O. Appazov[2*], Lyudmila K. Kibardina[1], Almir S. Gazizov[1], Alexandra D. Voloshina[1], Anna P. Luybina[1], Alexey B. Dobrynin[1,3], Igor A. Litvinov[1], Aiym E. Tolegen[4], Alexander R. Burilov[1], Michail A. Pudovik[1], Rakhymzhan A. Turmanov[2], Nurgali I. Akylbekov[2], Igor V. Alabugin[5] and Elena A. Chugunova[1]

1 Arbuzov Institute of Organic and Physical Chemistry, FRC Kazan Scientific Center, Russian Academy of Science, Arbuzova Str., 8, Kazan 420088, Russia; kibardina@iopc.ru (L.K.K.); agazizov@iopc.ru (A.S.G.); micrbi@iopc.ru (A.D.V.); luybina@iopc.ru (A.P.L.); aldo@iopc.ru (A.B.D.); litvinov@iopc.ru (I.A.L.); burilov@iopc.ru (A.R.B.); pudovik@iopc.ru (M.A.P.); chugunova.e.a@gmail.com (E.A.C.)

2 Laboratory of Engineering Profile "Physical and Chemical Methods of Analysis", Korkyt Ata Kyzylorda University, Aiteke Bi Street, 29A, Kyzylorda 120014, Kazakhstan; rakowrakha@gmail.com (R.A.T.); nurgaliakylbekov@gmail.com (N.I.A.)

3 Institute for Radio-Electronics and Telecommunications, Department for Design and Techniques of Electronic Tools Manufacturing, Kazan National Research Technical University Named After A. N. Tupolev-KAI, Karl Marx Str., 10, Kazan 420111, Russia

4 Institute Of Petroleum, Chemistry And Nanotechnologies, Department of General Organic and Petrochemical Synthesis Technology named after prof. G. Kamai, Kazan National Research Technological University, Karl Marx Str., 68, Kazan 420015, Russia; aiym-tolegen@mail.ru

5 Department of Chemistry and Biochemistry, Florida State University, 102 Varsity Way, Tallahassee, Fl 32306-4390, USA; alabugin@chem.fsu.edu

* Correspondence: xahter91@gmail.com (A.V.T.); nurasar.82@korkyt.kz (N.O.A.)

Contents

Comparison of preparative protocols for the synthesis of compound **6a** S2

The X-ray diffraction data S3

NMR spectra of compounds S9

Data of correlation 1D/2D NMR spectroscopy S48

IC_{50} Charts of the compounds for cell lines S55

Data of Quantitative 1D 1H NMR spectroscopy (qNMR) S61

CheckCIF/PLATON report S65

Table S1. Comparison of preparative protocols for the synthesis of compound **6a**

Entry	Addition mode	Yield of **6a** (%)	Notes
1	L-Proline (0.18 g, 1.53 mmol) and ninhydrin (0.27 g, 1.53 mmol) added in solid form to a boiling suspension of **1a** (0.3 g, 1.28 mmol)	45	Product isolated by recrystallization from absolute ethanol
2	Warm solution of L-proline (0.18 g, 1.53 mmol) and ninhydrin (0.27 g, 1.53 mmol) in absolute ethanol (2 mL) added dropwise to a boiling solution of **1a** (0.3 g, 1.27 mmol) in absolute ethanol (3 mL)	59	Precipitate filtered and dried *in vacuo*
3	Pre-formed azomethine ylide **4** (0.27 g, 1.27 mmol) added to a boiling solution of **1a** (0.3 g, 1.27 mmol) in absolute ethanol (5 mL)	67	Precipitate filtered; filtrate evaporated and residue recrystallized from absolute ethanol

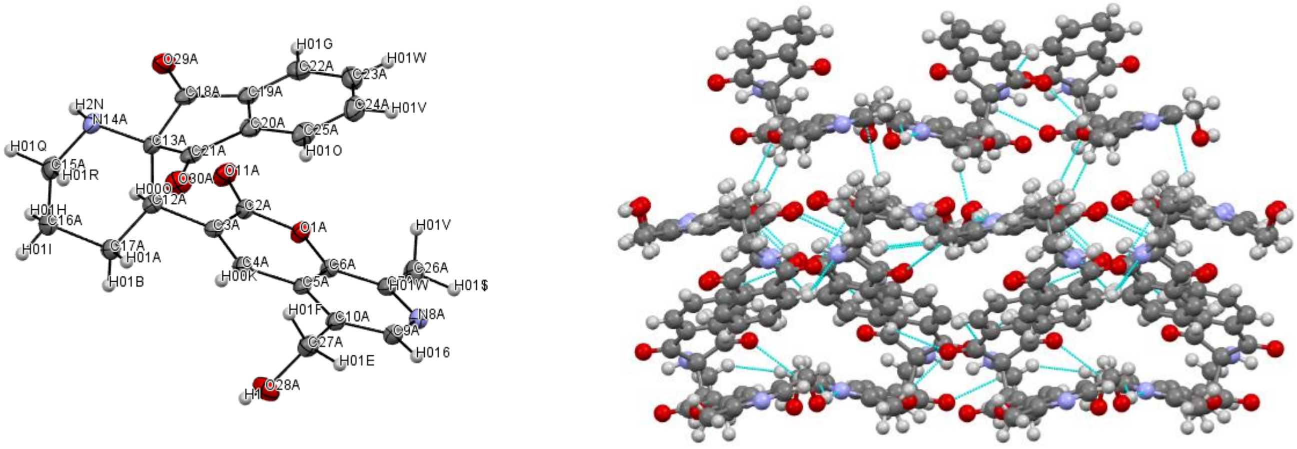

Figure S1. Molecular structure and fragments of the crystal packing of compound **6a**. Colour code: blue – nitrogen, red – oxygen, dark gray – carbon, light gray – hydrogen

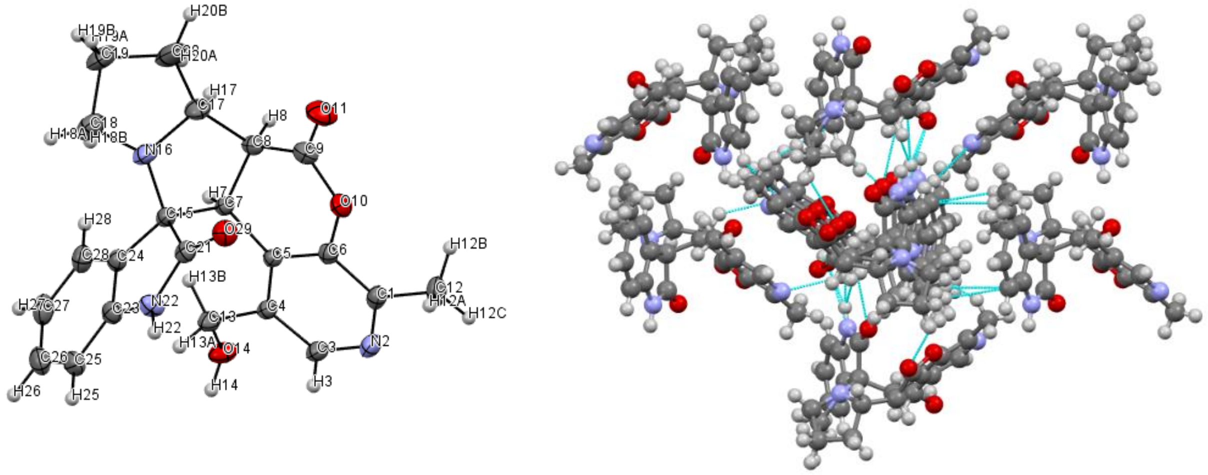

Figure S2. Molecular structure and fragments of the crystal packing of compound **10**. Colour code: blue – nitrogen, red – oxygen, dark gray – carbon, light gray – hydrogen

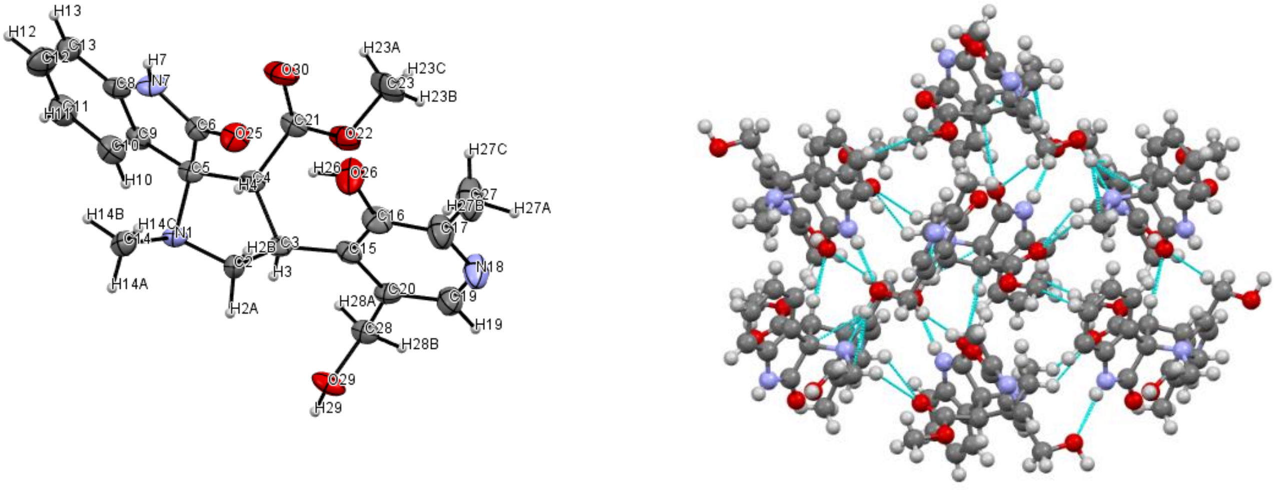

Figure S3. Molecular structure and fragments of the crystal packing of compound **12a**. Colour code: blue – nitrogen, red – oxygen, dark gray – carbon, light gray – hydrogen

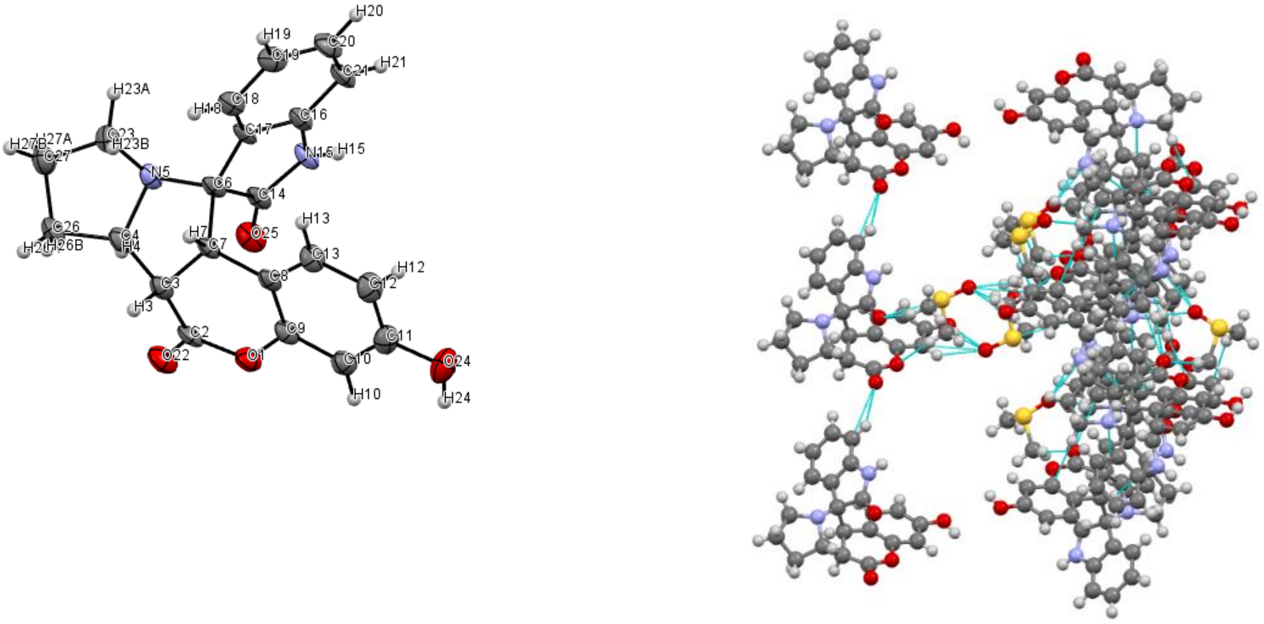

Figure S4. Molecular structure and fragments of the crystal packing of compound **17a**. Colour code: blue – nitrogen, red – oxygen, dark gray – carbon, light gray – hydrogen, yellow - sulfur

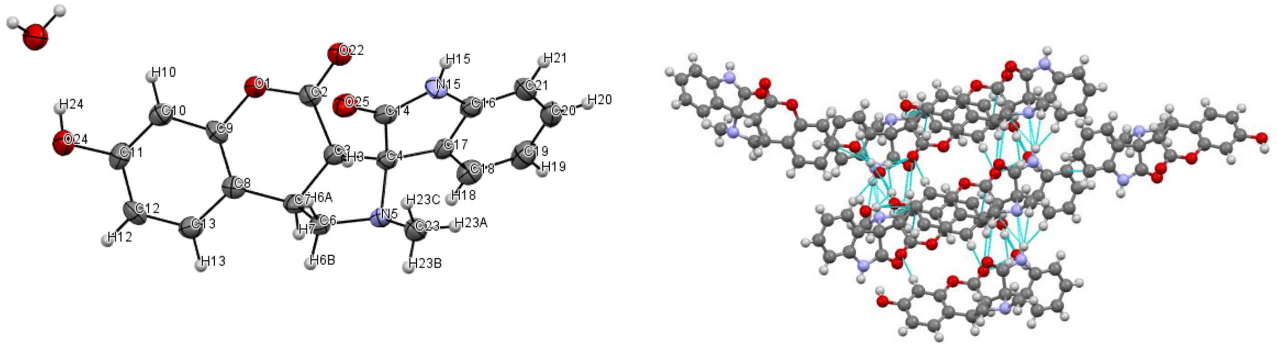

Figure S5. Molecular structure and fragments of the crystal packing of compound **20a**. Colour code: blue – nitrogen, red – oxygen, dark gray – carbon, light gray – hydrogen

Table S2. Data collection and selected refinement parameters for the obtained compounds.

	**6a**	**10**	**12a**
CCDC number	2573637	2573638	2573639
Empirical formula	$C_{23}H_{20}N_2O_5$	$C_{22}H_{21}N_3O_4$	$C_{21}H_{23}N_3O_5$
Formula weight	404.41	391.42	397.42
Temperature (K)	110.00	150.00	150.00
Crystal system	monoclinic	monoclinic	monoclinic
Space group	$P2_1/n$	$P2_1/n$	$P2_1/c$
a, *b*, c (Å)	12.7434(10)<br>13.0155(10)<br>22.6919(18)	9.4629(9)<br>15.8129(16)<br>12.6006(12)	14.1911(15)<br>13.6496(15)<br>12.3614(13)
β(°)	94.697(3)	108.043(3)	100.268(4)
V (Å^3)	3751.1(5)	1792.8(3)	2356.1(4)
Z	8	4	4
ρ_{calc}(mg/mm^3)	1.432	1.450	1.120
μ (mm^{-1})	0.102	0.101	0.081
F(000)	1696.0	824.0	840.0
Crystal size (mm)	0.15 × 0.15 × 0.12	0.4 × 0.22 × 0.16	0.12 × 0.08 × 0.05
Radiation	MoKα (λ = 0.71073)	MoKα (λ = 0.71073)	MoKα (λ = 0.71073)
2Θ range for data collection/°	3.804 to 50.606	4.746 to 69.232	2.916 to 61.092
Index ranges	-15 ≤ h ≤ 15, -15 ≤ k ≤ 15, -27 ≤ l ≤ 27	-15 ≤ h ≤ 15, -25 ≤ k ≤ 25, -20 ≤ l ≤ 20	-19 ≤ h ≤ 20, -19 ≤ k ≤ 19, -17 ≤ l ≤ 17
Reflections collected	105027	66845	41400
Independent reflections	6806 [R_{int} = 0.1037, R_{sigma} = 0.0480]	7662 [R_{int} = 0.0527, R_{sigma} = 0.0295]	7205 [R_{int} = 0.1424, R_{sigma} = 0.1198]
Data/restraints/parameters	6806/0/553	7662/0/277	7205/0/277
Goodness-of-fit on F^2	1.059	1.070	1.029
Final R indexes [I>=2σ (I)]	R_1 = 0.0417, wR_2 = 0.1078	R_1 = 0.0533, wR_2 = 0.1421	R_1 = 0.0916, wR_2 = 0.2341
Final R indexes [all data]	R_1 = 0.0610, wR_2 = 0.1146	R_1 = 0.0627, wR_2 = 0.1475	R_1 = 0.1406, wR_2 = 0.2545
Largest diff. peak/hole / e Å^{-3}	0.28/-0.23	0.79/-0.52	0.48/-0.36

	**17a**	**20a**
CCDC number	2573640	2573641
Empirical formula	$C_{21}H_{18}N_2O_4$ × 2(C_2H_6OS)	$C_{19}H_{16}N_2O_4$ × H_2O
Formula weight	518.63	354.35
Temperature (K)	105.00	293(2)
Crystal system	monoclinic	monoclinic
Space group	$P2_1/n$	$P2_1/n$
a, *b*, c (Å)	10.3836(12)<br>29.164(4)<br>10.4383(11)	15.8032(15)<br>6.2603(6)<br>16.5254(16)
β (°)	114.315(4)	96.234(3)
V (Å^3)	2880.6(6)	1625.2(3)
Z	4	4
ρ_{calc}(mg/mm^3)	1.196	1.448
μ (mm^{-1})	0.223	0.106
F(000)	1096.0	744.0
Crystal size (mm)	0.336 × 0.292 × 0.246	0.355 × 0.052 × 0.032
Radiation	MoKα (λ = 0.71073)	MoKα (λ = 0.71073)
2Θ range for data collection/°	4.504 to 61.126	3.776 to 55.988
Index ranges	-14 ≤ h ≤ 14, -41 ≤ k ≤ 41, -14 ≤ l ≤ 14	-20 ≤ h ≤ 20, -8 ≤ k ≤ 8, -21 ≤ l ≤ 21
Reflections collected	89386	41566
Independent reflections	8716 [R_{int} = 0.0651, R_{sigma} = 0.0299]	3922 [R_{int} = 0.1800, R_{sigma} = 0.1026]
Data/restraints/parameters	8712/0/351	3922/0/246
Goodness-of-fit on F^2	1.060	1.023
Final R indexes [I>=2σ (I)]	R_1 = 0.1072, wR_2 = 0.3299	R_1 = 0.0569, wR_2 = 0.1139
Final R indexes [all data]	R_1 = 0.1113, wR_2 = 0.3324	R_1 = 0.1119, wR_2 = 0.1317
Largest diff. peak/hole / e Å^{-3}	1.24/-0.96	0.32/-0.25

Table S3. Parameters of intermolecular contacts in the crystal of compounds **6**, **10**, **12a**, **17a**, **20a**.

D—H···A	D—H, Å	H…A, Å	D…A, Å	DHA, deg.	Symmetry operations
**6a**					
N14A--H14A..O29A	0.95(2)	2.48(2)	2.8153(19)	100.6(14)	.
N14B--H14B..O11A	0.96(2)	2.69(2)	3.396(2)	130.8(16)	-1+x,-1+y,z
O28A--H28A..N8B	0.8400	1.9300	2.7507(19)	165.00	.
O28B--H28B..N8A	0.8400	1.9600	2.7933(19)	168.00	-1+x,y,z
C12A--H12A..O11A	1.0000	2.4600	2.861(2)	103.00	.
C12B--H12B..O11B	1.0000	2.4300	2.866(2)	106.00	.
C15B--H15C..O30B	0.9900	2.5700	3.163(2)	118.00	.
C22B--H22B..N14A	0.9500	2.6000	3.386(2)	140.00	x,-1+y,z
C26A--H26B..O29B	0.9800	2.3400	3.311(2)	172.00	1+x,1+y,z
C26A--H26C..O1A	0.9800	2.5800	3.477(2)	152.00	2-x,2-y,1-z
C26B--H26F..O29A	0.9800	2.4800	3.441(2)	165.00	x,-1+y,z
**10**					
O14--H14..O11	0.92(2)	1.85(2)	2.7562(12)	169(2)	1+x,y,z
N22--H22..O14	0.8800	1.9900	2.8511(11)	165.00	-1/2+x,3/2-y,-1/2+z
C3--H3..O14	0.9500	2.4800	2.8176(13)	101.00	.
C8--H8..N2	1.0000	2.5800	3.3679(13)	135.00	1-x,1-y,1-z
C17--H17..O11	1.0000	2.4300	2.8833(14)	107.00	.
C25--H25..N2	0.9500	2.5800	3.4126(14)	147.00	3/2-x,1/2+y,1/2-z
C28--H28..O29	0.9500	2.5800	3.3726(13)	140.00	1/2+x,3/2-y,1/2+z
**12a**					
N7--H7..O29	0.68(5)	2.17(5)	2.845(3)	169(5)	1-x,1/2+y,1/2-z
O26--H26..O25	0.79(4)	1.84(4)	2.599(3)	161(4)	.
O29--H29..N1	1.04(6)	1.76(6)	2.789(3)	172(5)	1-x,1-y,1-z
C2--H2A..O30	0.9900	2.6000	3.231(3)	122.00	1-x,-1/2+y,1/2-z
C2--H2B..O25	0.9900	2.4900	3.082(3)	118.00	.
C3--H3..O29	1.0000	2.5900	3.266(3)	125.00	.

C4--H4..O25	1.0000	2.3000	3.156(3)	143.00	x,3/2-y,1/2+z
C28--H28A..O25	0.9900	2.5700	3.553(3)	173.00	x,3/2-y,1/2+z
**17a**					
N15--H15..O35	0.8800	1.9200	2.744(5)	155.00	x,y,1+z
O24--H24..O31	0.8400	1.9200	2.665(5)	148.00	.
C4--H4..O22	1.0000	2.4800	2.904(5)	105.00	.
C10--H10..O31	0.9500	2.5100	3.191(5)	128.00	.
C21--H21..O22	0.9500	2.5000	3.183(5)	129.00	x,y,1+z
C23--H23B..O35	0.9900	2.5600	3.431(6)	146.00	-1/2+x,3/2-y,1/2+z
C32--H32B..O25	0.9800	2.5700	3.292(7)	130.00	1-x,1-y,1-z
C36--H36C..O22	0.9800	2.4700	3.426(15)	166.00	.
**20a**					
N15--H15..N5	0.8600	2.2800	3.080(3)	154.00	x,1+y,z
O24--H24..O30	0.8200	1.8000	2.605(3)	167.00	.
O30--H30A..O24	0.96(3)	1.81(3)	2.756(3)	168(3)	1/2-x,1/2+y,1/2-z
O30--H30B..O22	0.86(3)	1.94(3)	2.762(3)	158(3)	1-x,2-y,1-z
C3--H3..O24	0.9800	2.5600	3.431(3)	147.00	1-x,1-y,1-z
C6--H6A..O25	0.9700	2.4700	3.083(3)	121.00	.
C6--H6B..O25	0.9700	2.4100	3.178(3)	136.00	x,-1+y,z
C7--H7..O22	0.9800	2.5600	3.505(3)	161.00	x,-1+y,z
C10--H10..O22	0.9300	2.5400	3.453(3)	166.00	1-x,2-y,1-z

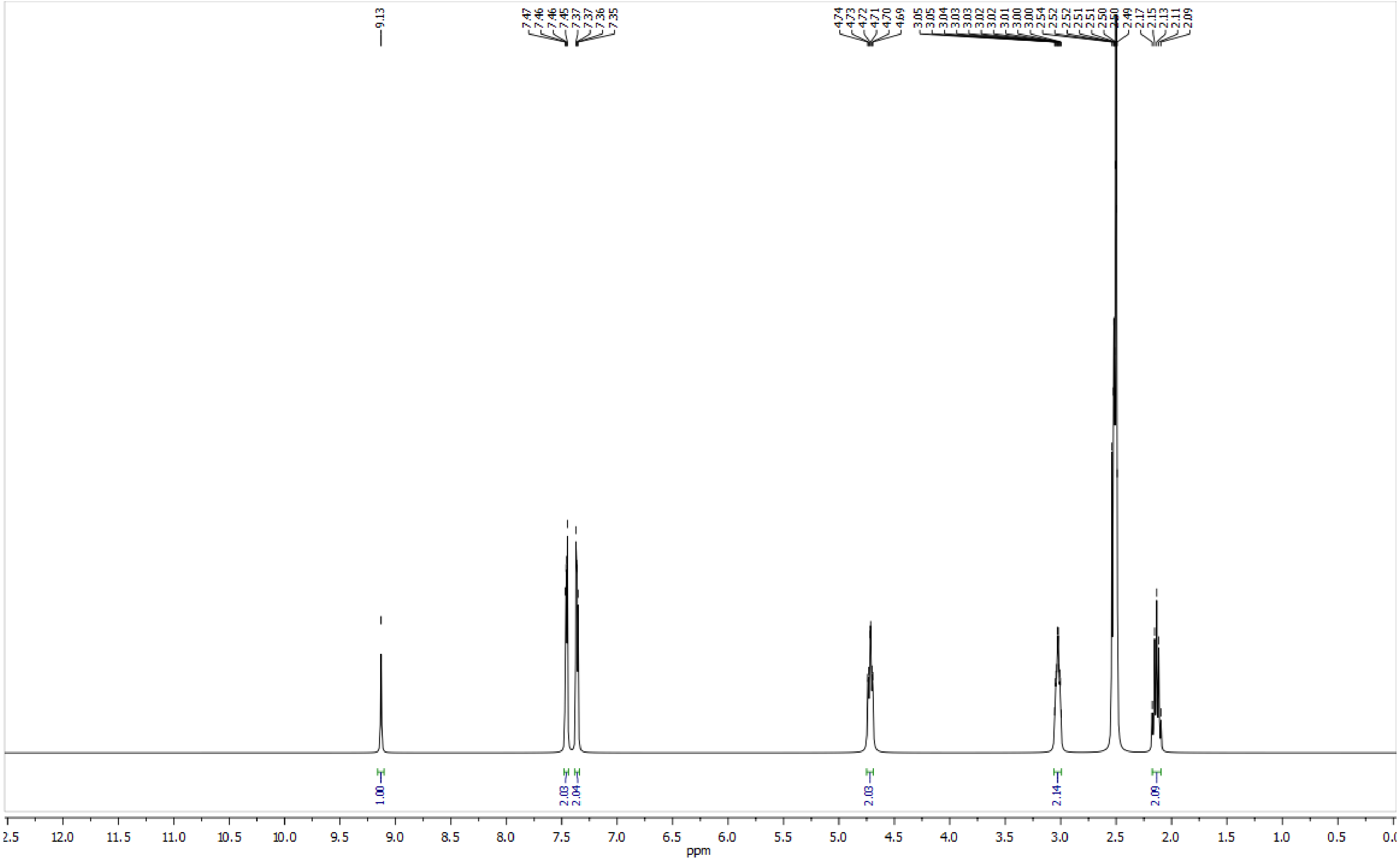

^{1}H NMR (400 MHz, DMSO-d_6) δ 7.46 (dd, J = 5.2, 3.0 Hz, 1H), 7.36 (dd, J = 5.2, 3.1 Hz, 1H), 4.72 (td, J = 7.8, 2.2 Hz, 1H), 3.03 (tq, J = 7.8, 2.5 Hz, 1H), 2.13 (p, J = 7.9 Hz, 1H).

Figure S6. ^{1}H NMR spectra of the compound **4** (DMSO-d_6)

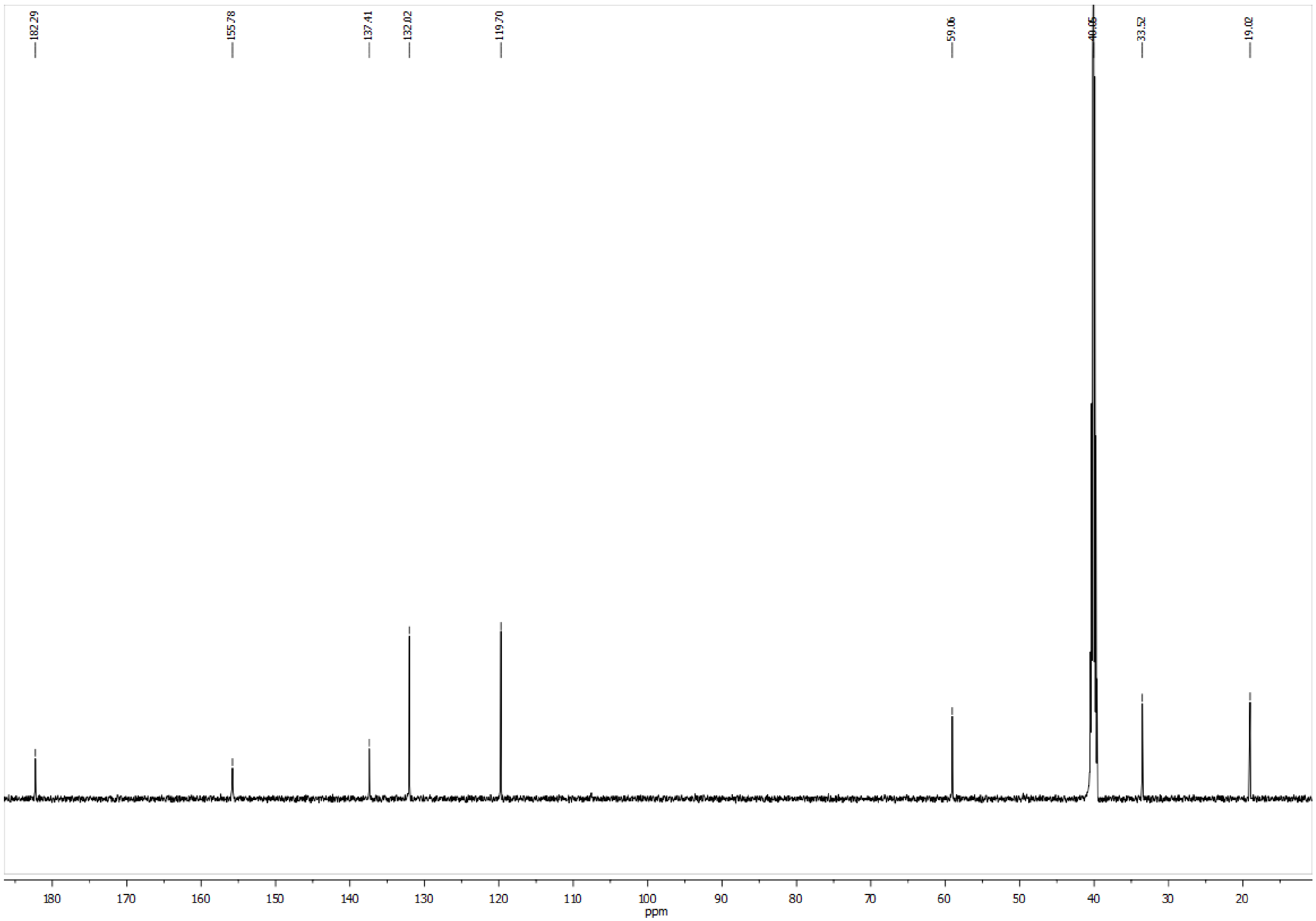

Figure S7. ^{13}C NMR spectra of the compound **4** (DMSO-d_6)

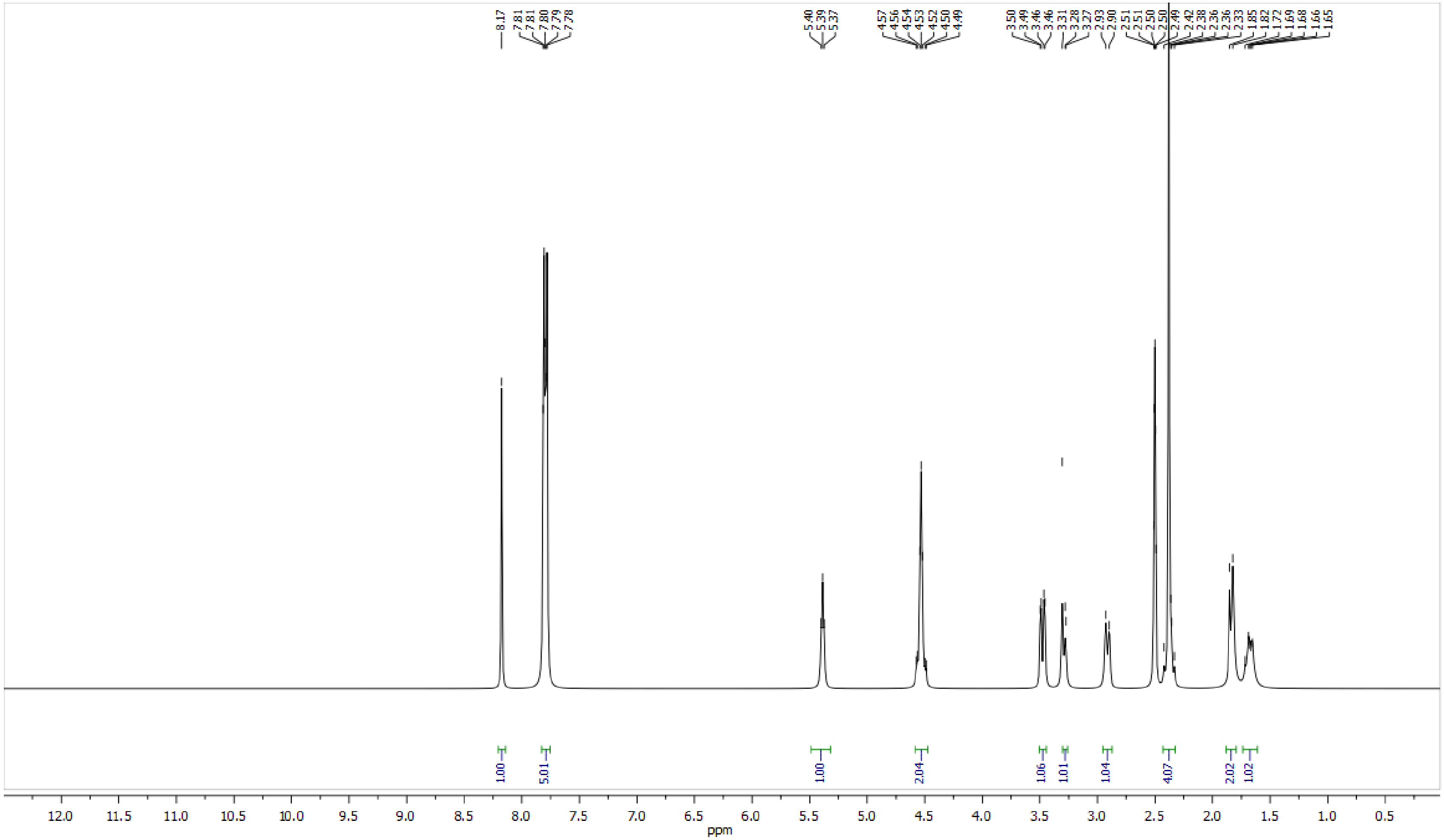

^{1}H NMR (400 MHz, DMSO-d_6) δ 5.39 (t, J = 5.1 Hz, 0H), 3.48 (dd, J = 12.6, 3.2 Hz, 1H), 2.91 (d, J = 12.6 Hz, 0H), 1.84 (d, J = 11.4 Hz, 1H).

Figure S8. ^{1}H NMR spectra of the compound **6a** (DMSO-d_6)

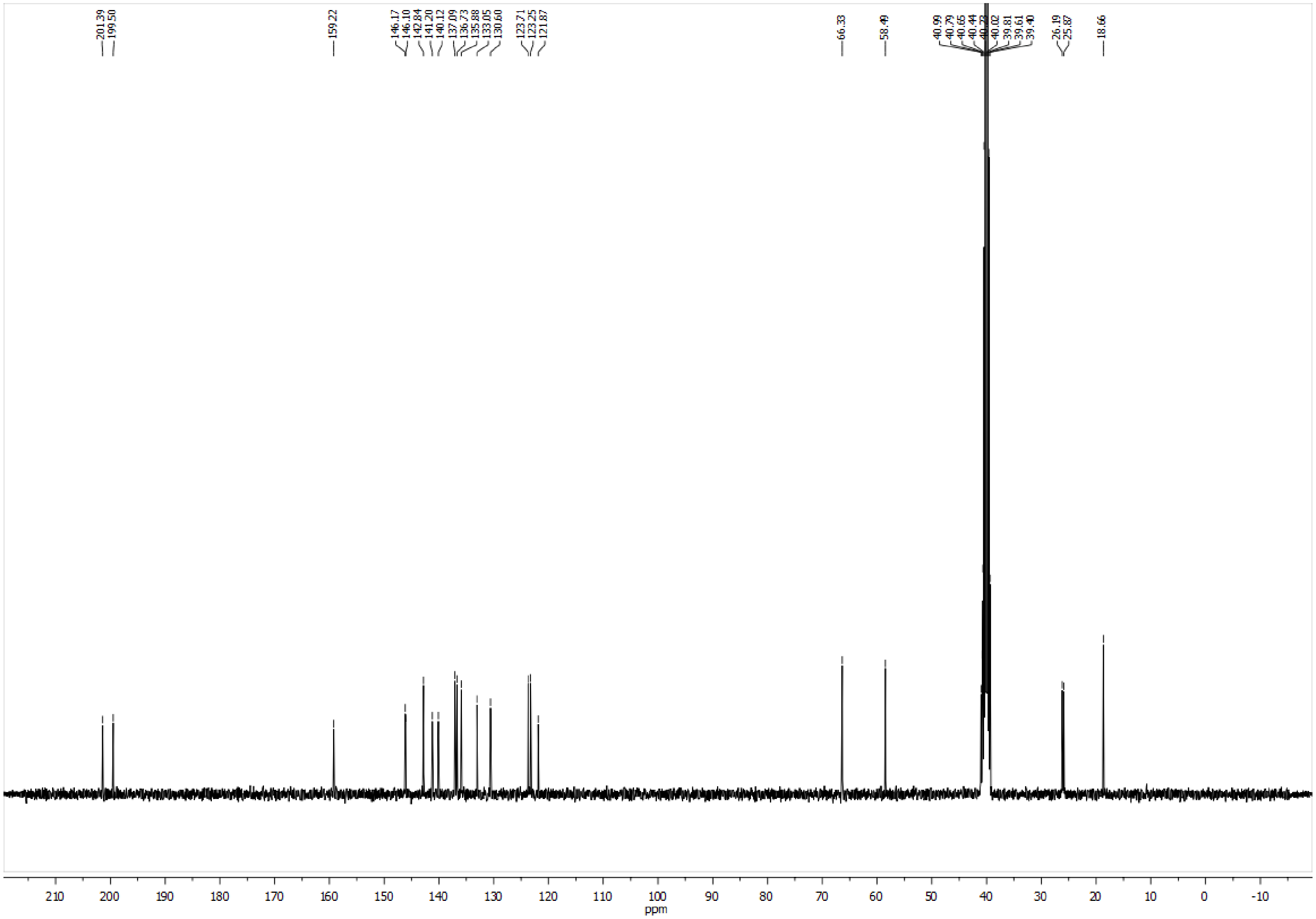

Figure S9. ^{13}C NMR spectra of the compound **6a** (DMSO-d_6)

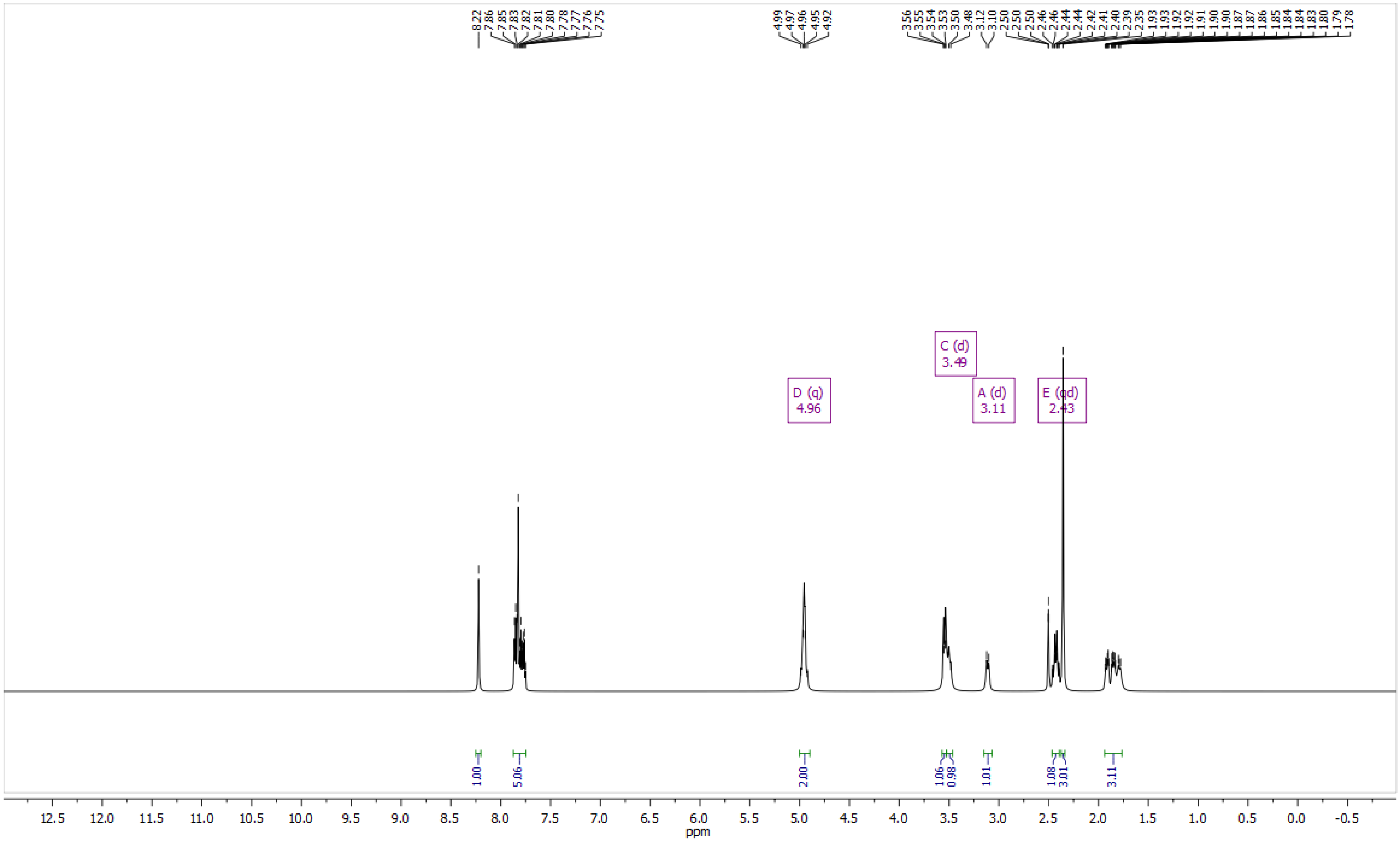

^{1}H NMR (600 MHz, DMSO-d_6) δ 4.96 (q, J = 9.4, 6.3 Hz, 2H), 3.49 (d, J = 12.0 Hz, 1H), 3.11 (d, J = 12.4 Hz, 1H), 2.43 (qd, J = 12.7, 2.5 Hz, 1H).

Figure S10. ^{1}H NMR spectra of the compound **6b** (DMSO-d_6)

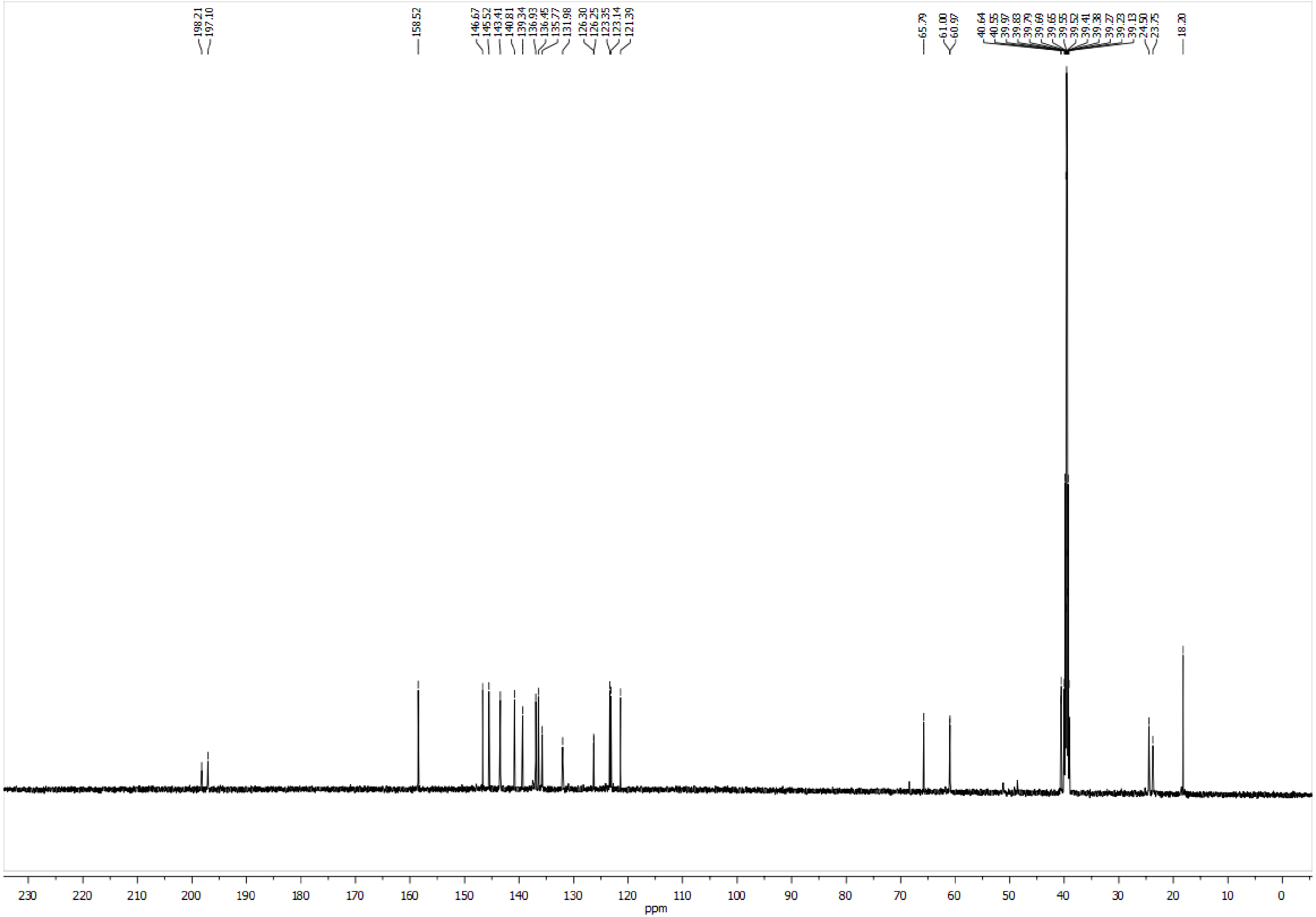

Figure S11. ^{13}C NMR spectra of the compound **6b** (DMSO-d_6)

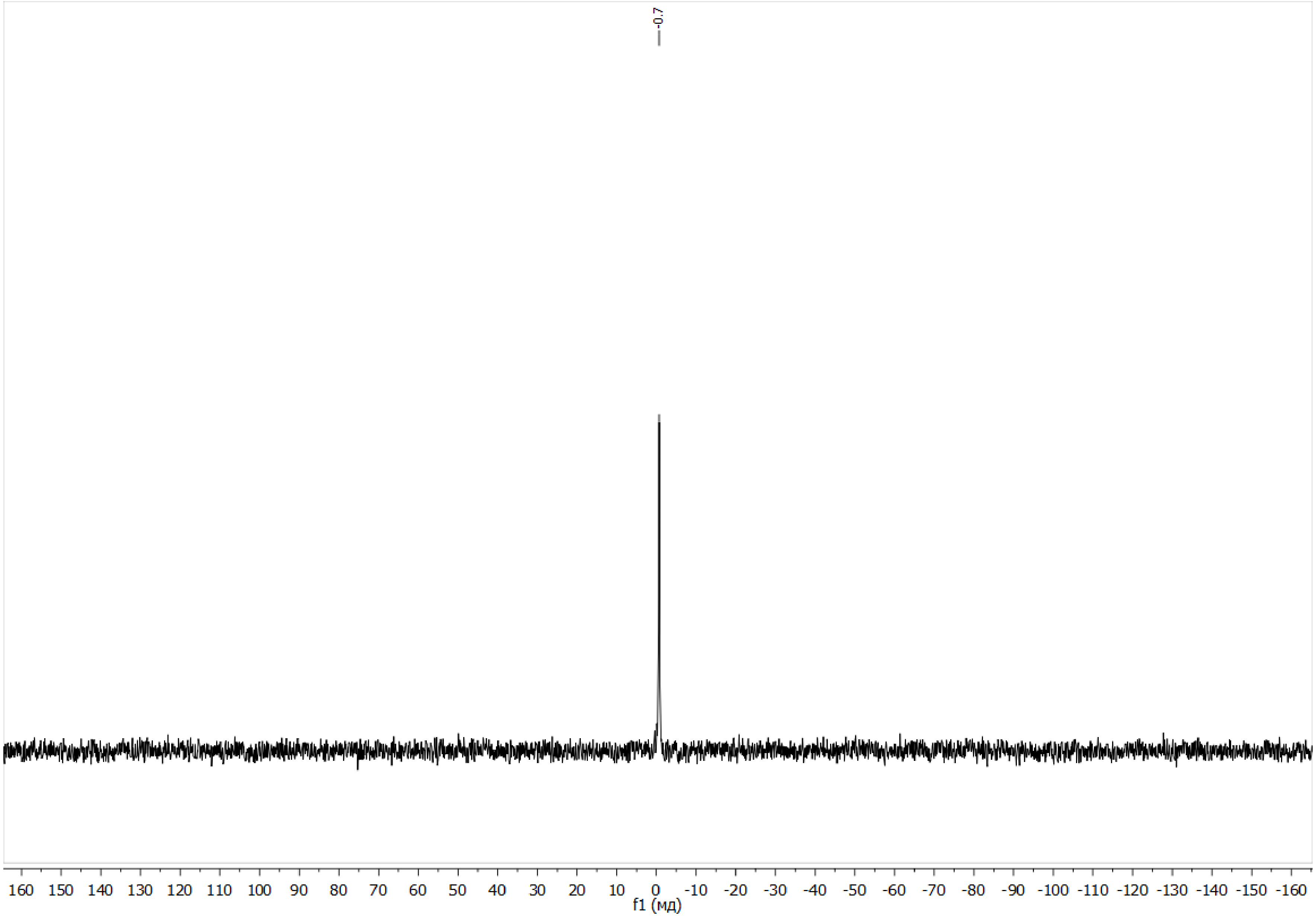

Figure S12. ^{31}P NMR spectra of the compound **6b** (DMSO-d_6)

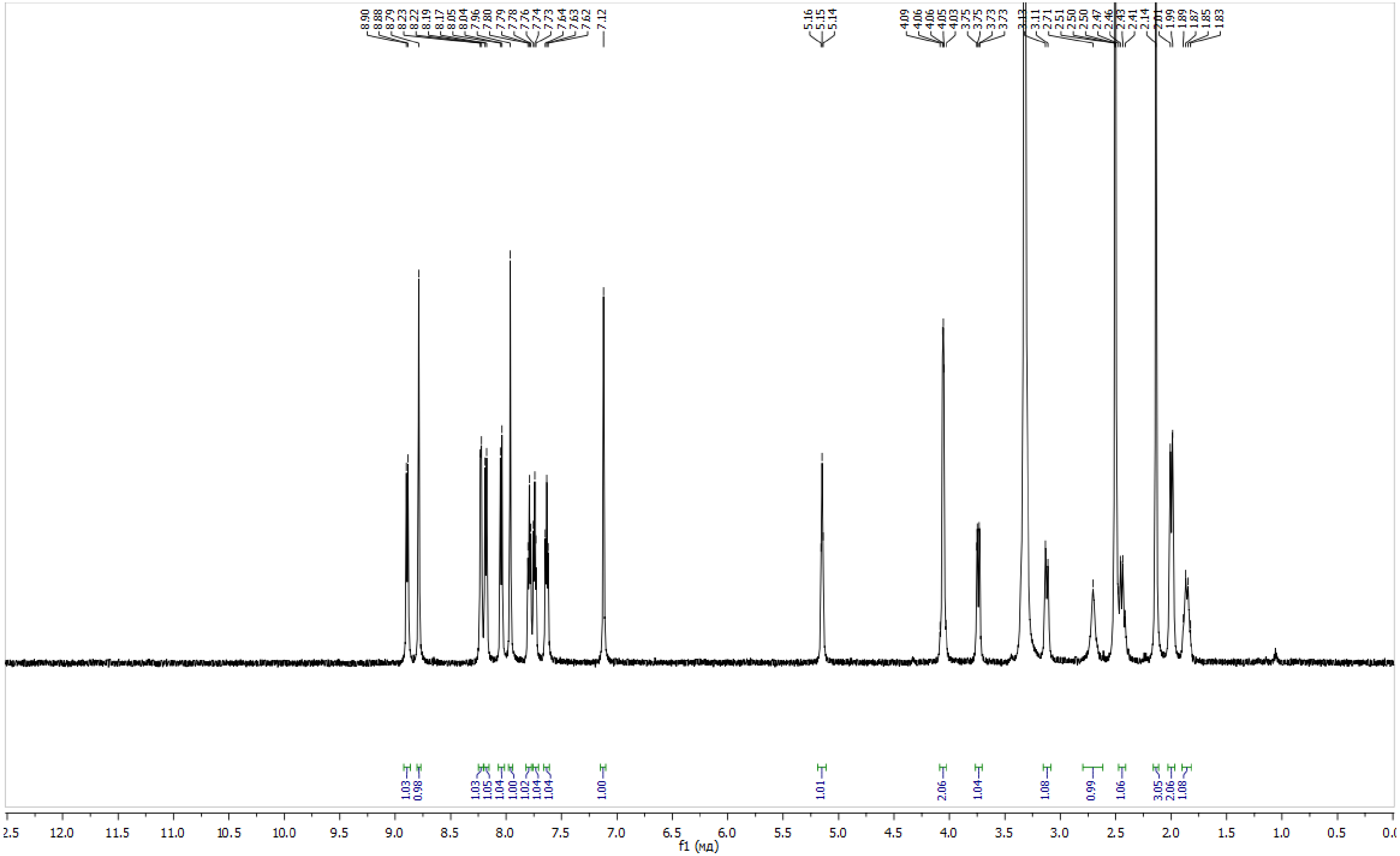

^{1}H NMR (600 MHz, DMSO-d_6) δ 8.89 (d, J = 8.6 Hz, 1H), 8.23 (d, J = 6.7 Hz, 1H), 8.18 (d, J = 8.5 Hz, 1H), 8.04 (d, J = 8.6 Hz, 1H), 7.79 (t, J = 7.6 Hz, 1H), 7.74 (t, J = 7.7 Hz, 1H), 7.63 (t, J = 7.7 Hz, 1H), 5.15 (t, J = 5.6 Hz, 1H), 3.74 (dd, J = 13.1, 3.2 Hz, 1H), 3.12 (d, J = 13.5 Hz, 1H), 2.44 (dd, J = 24.9, 12.3 Hz, 1H), 2.00 (d, J = 12.7 Hz, 2H).

Figure S13. ^{1}H NMR spectra of the compound **8** (DMSO-d_6)

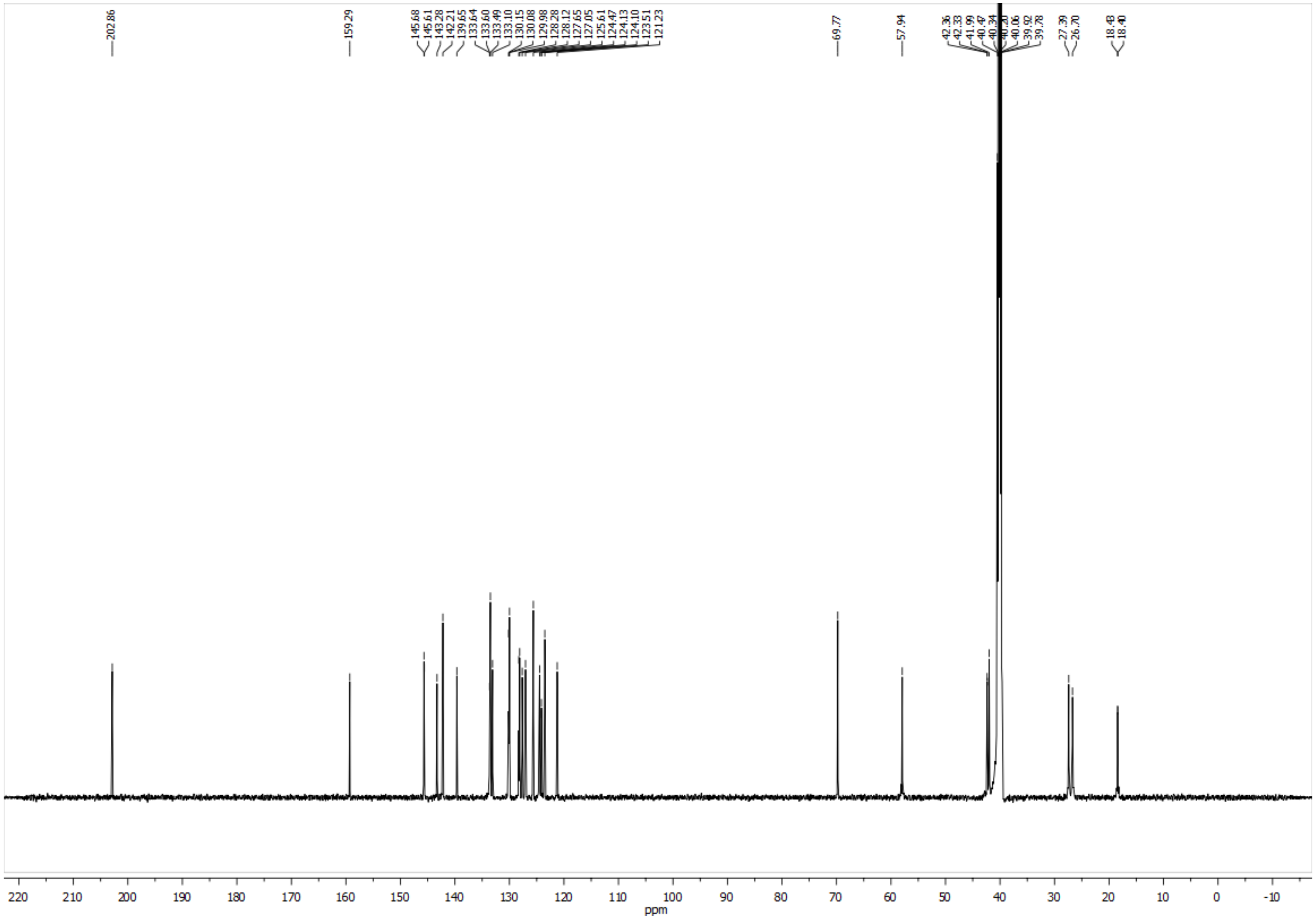

Figure S14. ^{13}C NMR spectra of the compound **8** (DMSO-d_6)

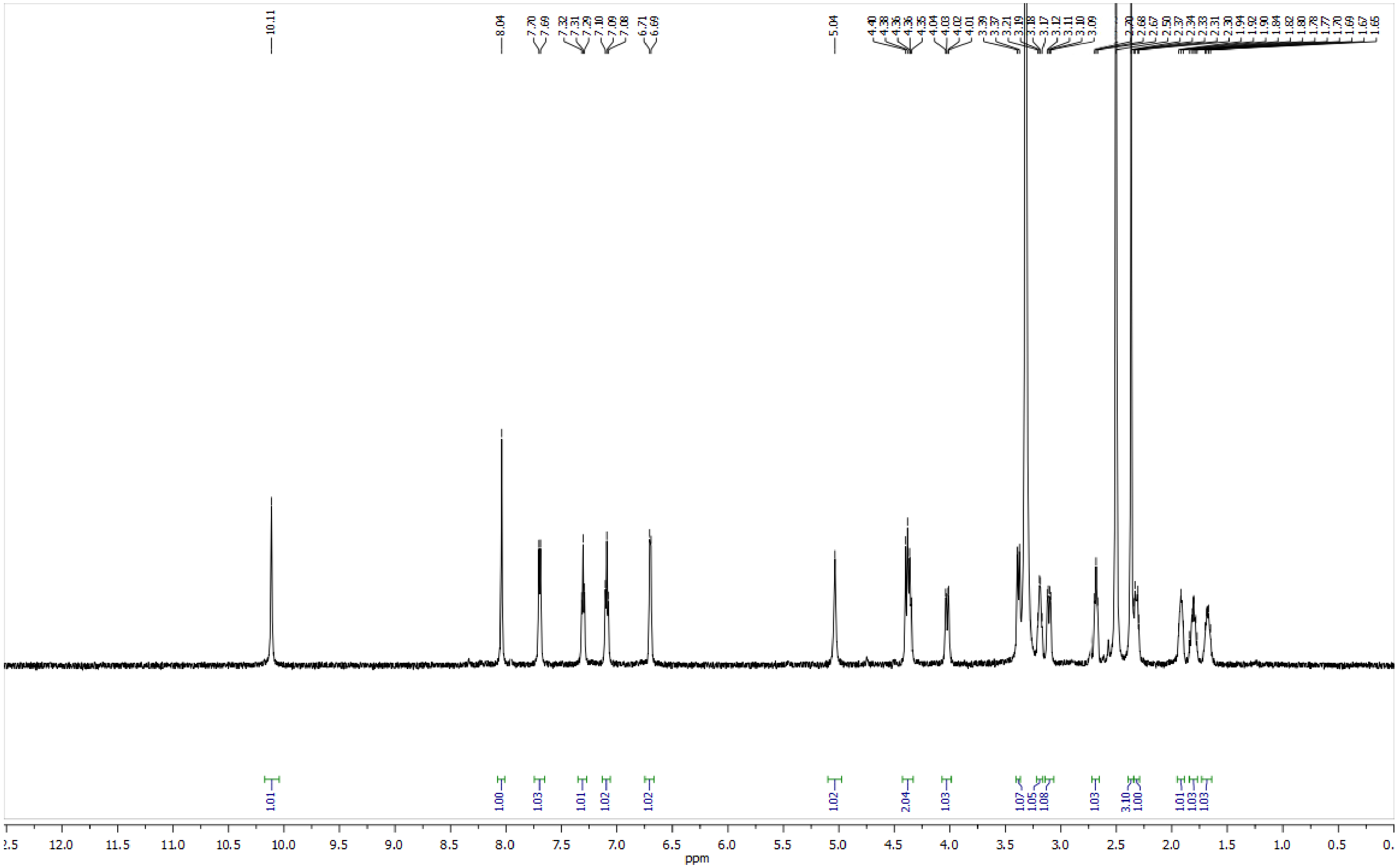

^{1}H NMR (600 MHz, DMSO-d_6) δ 7.70 (d, J = 7.5 Hz, 1H), 7.31 (t, J = 7.8 Hz, 1H), 7.09 (t, J = 7.5 Hz, 1H), 6.70 (d, J = 7.7 Hz, 1H), 4.02 (dd, J = 13.0, 4.5 Hz, 1H), 3.38 (d, J = 10.4 Hz, 1H), 3.22 – 3.15 (m, 0H), 3.11 (dd, J = 14.4, 4.8 Hz, 0H), 2.68 (t, J = 8.0 Hz, 1H), 2.32 (dd, J = 18.9, 7.4 Hz, 1H).

Figure S15. ^{1}H NMR spectra of the compound **10** (DMSO-d_6)

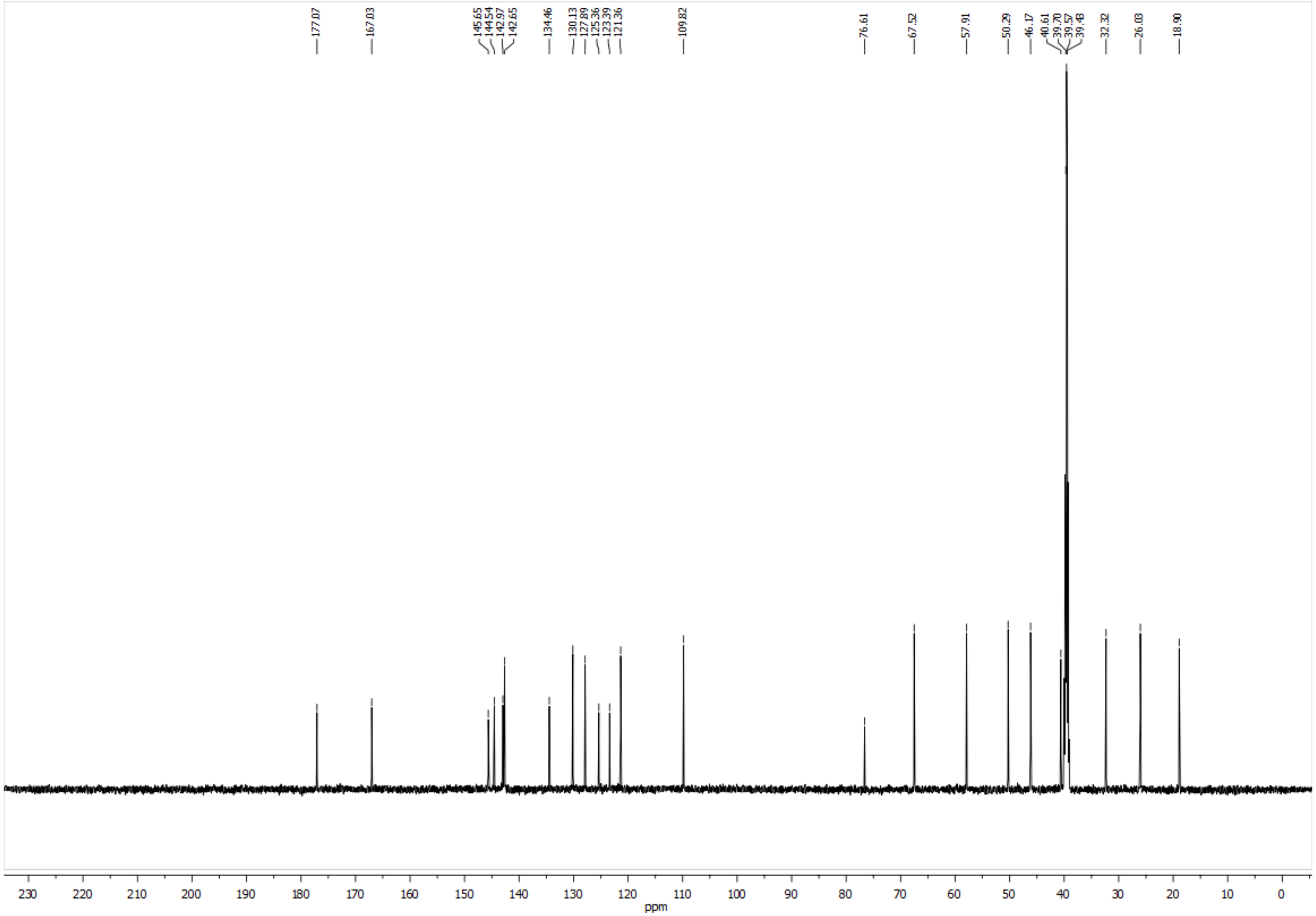

Figure S16. ^{13}C NMR spectra of the compound **10** (DMSO-d_6)

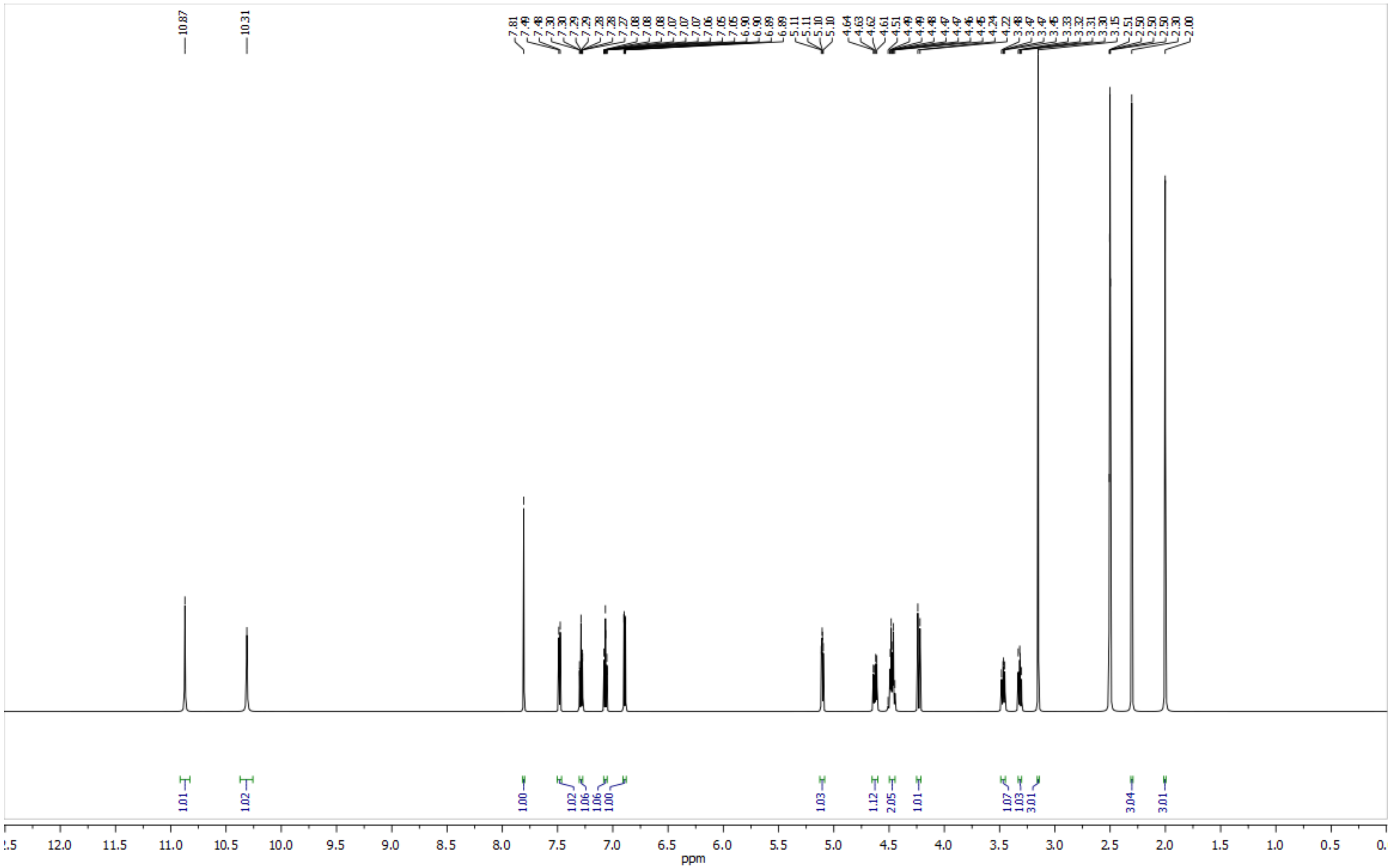

^{1}H NMR (600 MHz, DMSO-d_6) δ 7.48 (d, J = 7.4 Hz, 1H), 7.29 (td, J = 7.7, 1.2 Hz, 1H), 7.07 (td, J = 7.5, 1.0 Hz, 1H), 6.89 (dd, J = 7.7, 0.9 Hz, 1H), 5.10 (dd, J = 6.2, 4.7 Hz, 1H), 4.63 (dd, J = 12.6, 6.3 Hz, 1H), 4.51 – 4.43 (m, 2H), 4.23 (d, J = 12.1 Hz, 1H), 3.47 (dd, J = 9.9, 7.6 Hz, 1H), 3.32 (dd, J = 9.9, 7.7 Hz, 1H).

Figure S17. ^{1}H NMR spectra of the compound **12a** (DMSO-d_6)

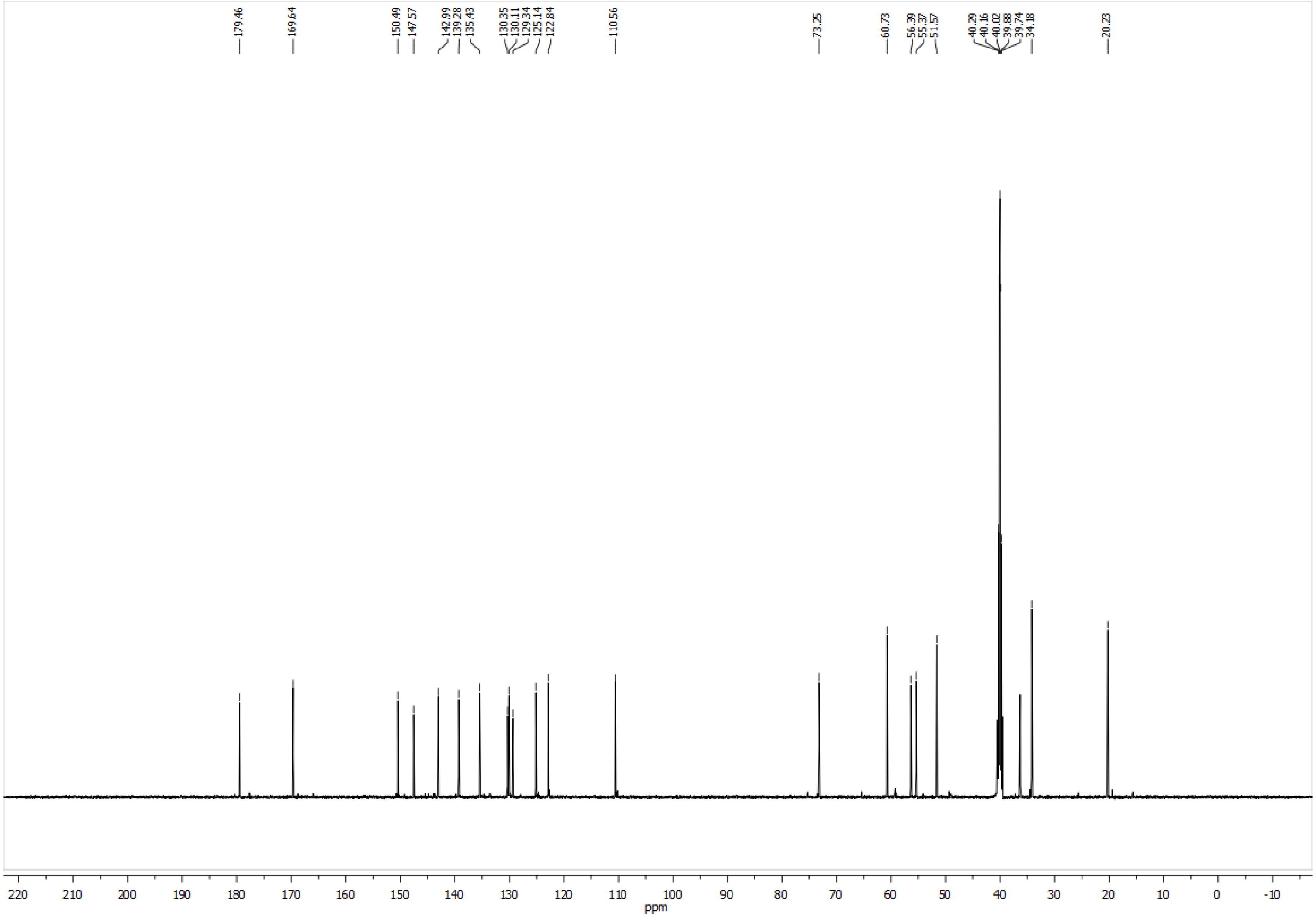

Figure S18. ^{13}C NMR spectra of the compound **12a** (DMSO-d_6)

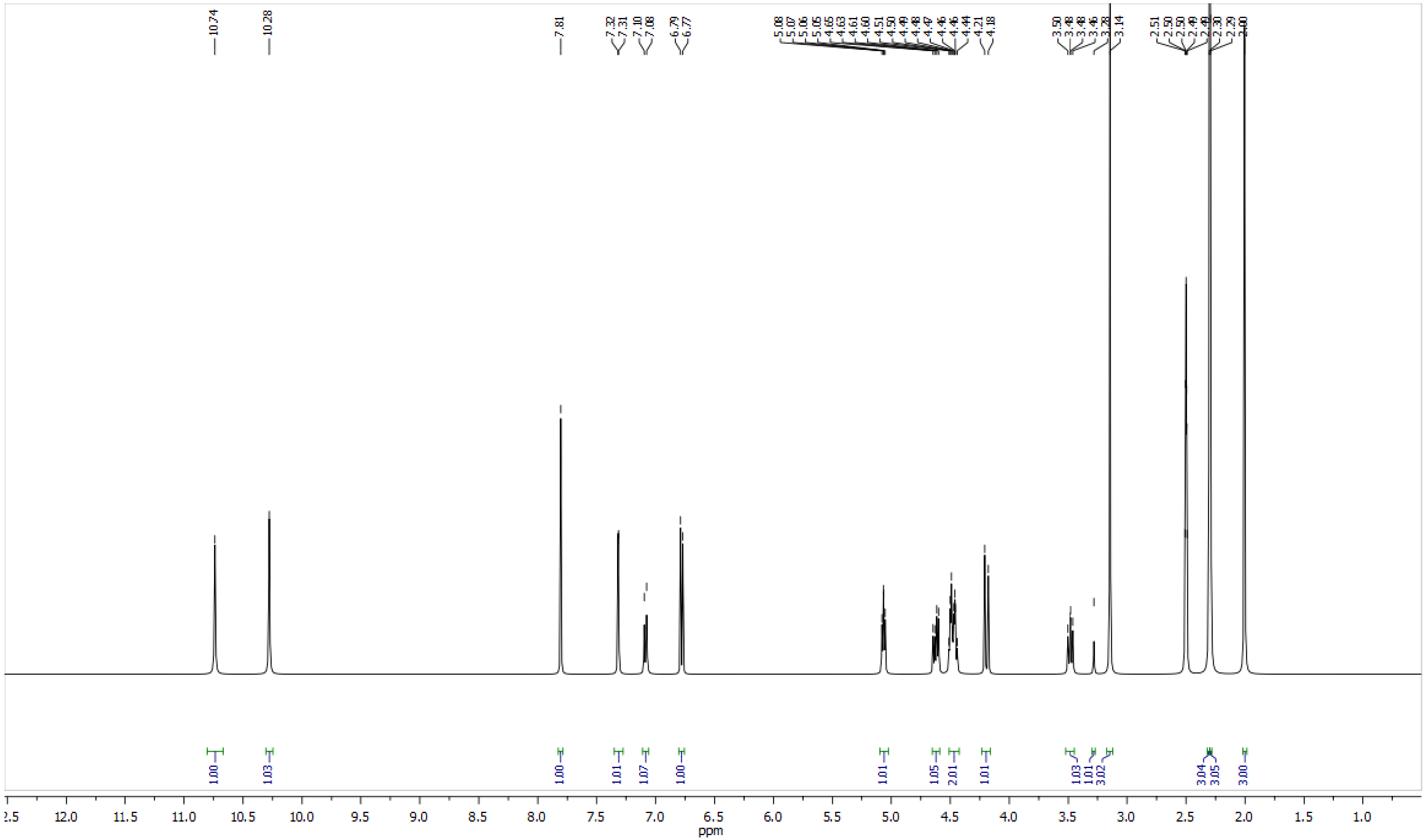

^{1}H NMR (400 MHz, DMSO-d_6) δ 7.32 (d, J = 1.7 Hz, 1H), 7.09 (d, J = 7.9 Hz, 1H), 6.78 (d, J = 7.8 Hz, 1H), 5.06 (dd, J = 6.1, 4.7 Hz, 1H), 4.62 (dd, J = 12.6, 6.0 Hz, 1H), 4.54 – 4.39 (m, 2H), 4.19 (d, J = 12.1 Hz, 1H), 3.48 (dd, J = 9.7, 7.7 Hz, 1H).

Figure S19. ^{1}H NMR spectra of the compound **12b** (DMSO-d_6)

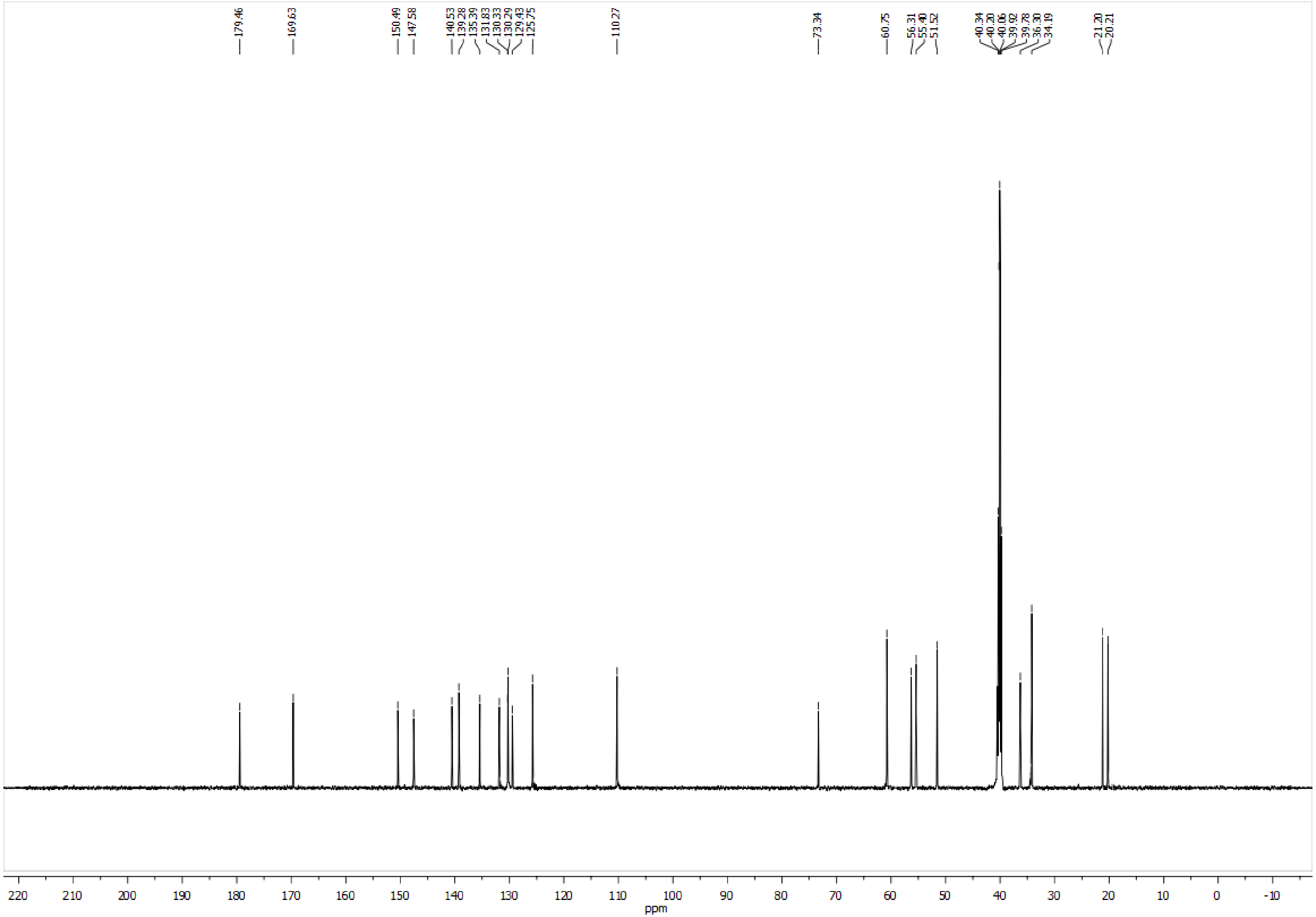

Figure S20. ^{13}C NMR spectra of the compound **12b** (DMSO-d_6)

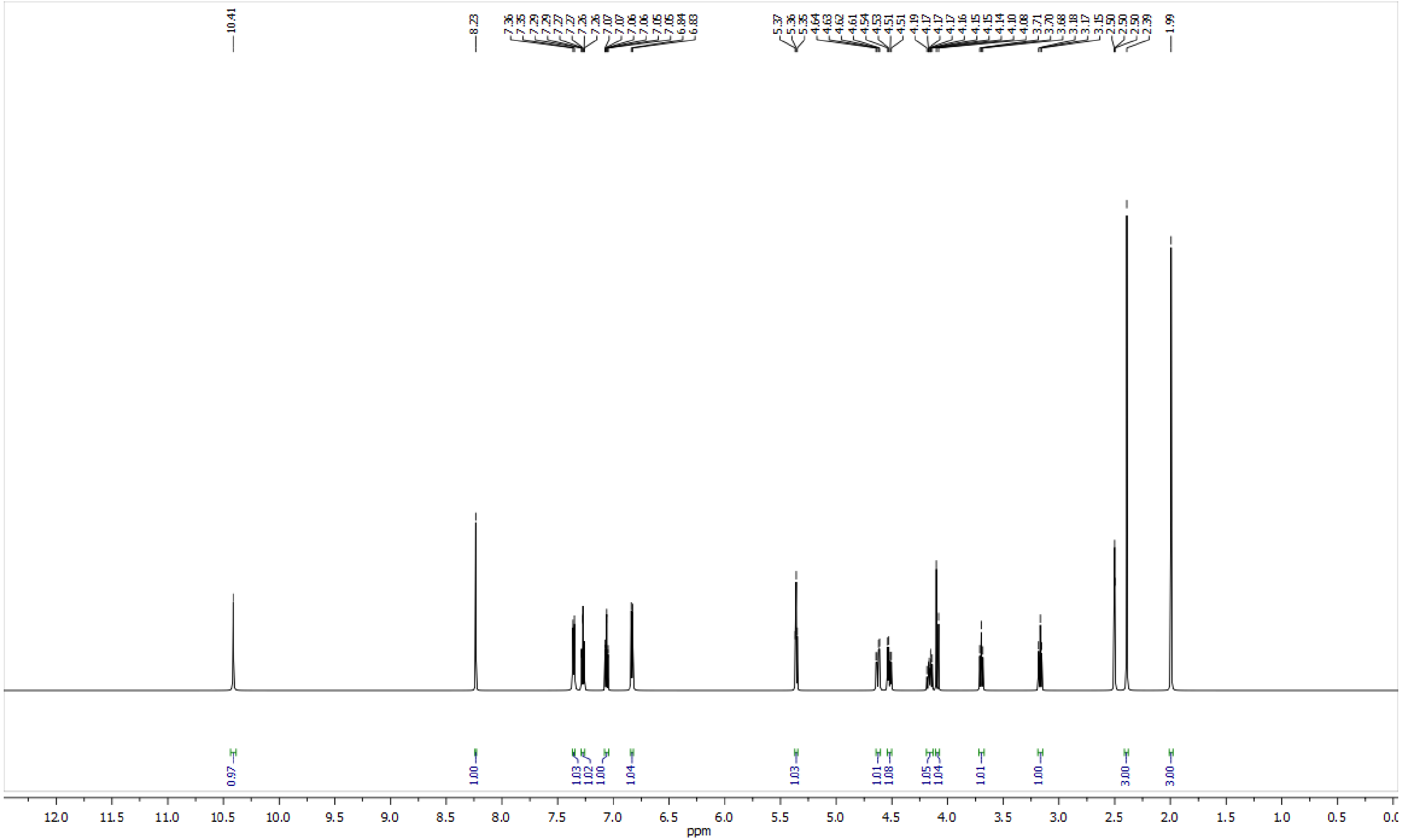

^{1}H NMR (600 MHz, DMSO-d_6) δ 7.36 (d, J = 7.3 Hz, 1H), 7.27 (td, J = 7.7, 1.1 Hz, 1H), 7.06 (td, J = 7.5, 1.0 Hz, 1H), 6.83 (d, J = 7.7 Hz, 1H), 5.36 (t, J = 5.4 Hz, 1H), 4.63 (dd, J = 13.5, 5.4 Hz, 1H), 4.52 (dd, J = 13.5, 5.5 Hz, 1H), 4.16 (ddd, J = 12.2, 9.4, 7.8 Hz, 1H), 4.09 (d, J = 12.2 Hz, 1H), 3.70 (t, J = 8.3 Hz, 1H), 3.17 (t, J = 9.1 Hz, 1H).

Figure S21. ^{1}H NMR spectra of the compound **13a** (DMSO-d_6)

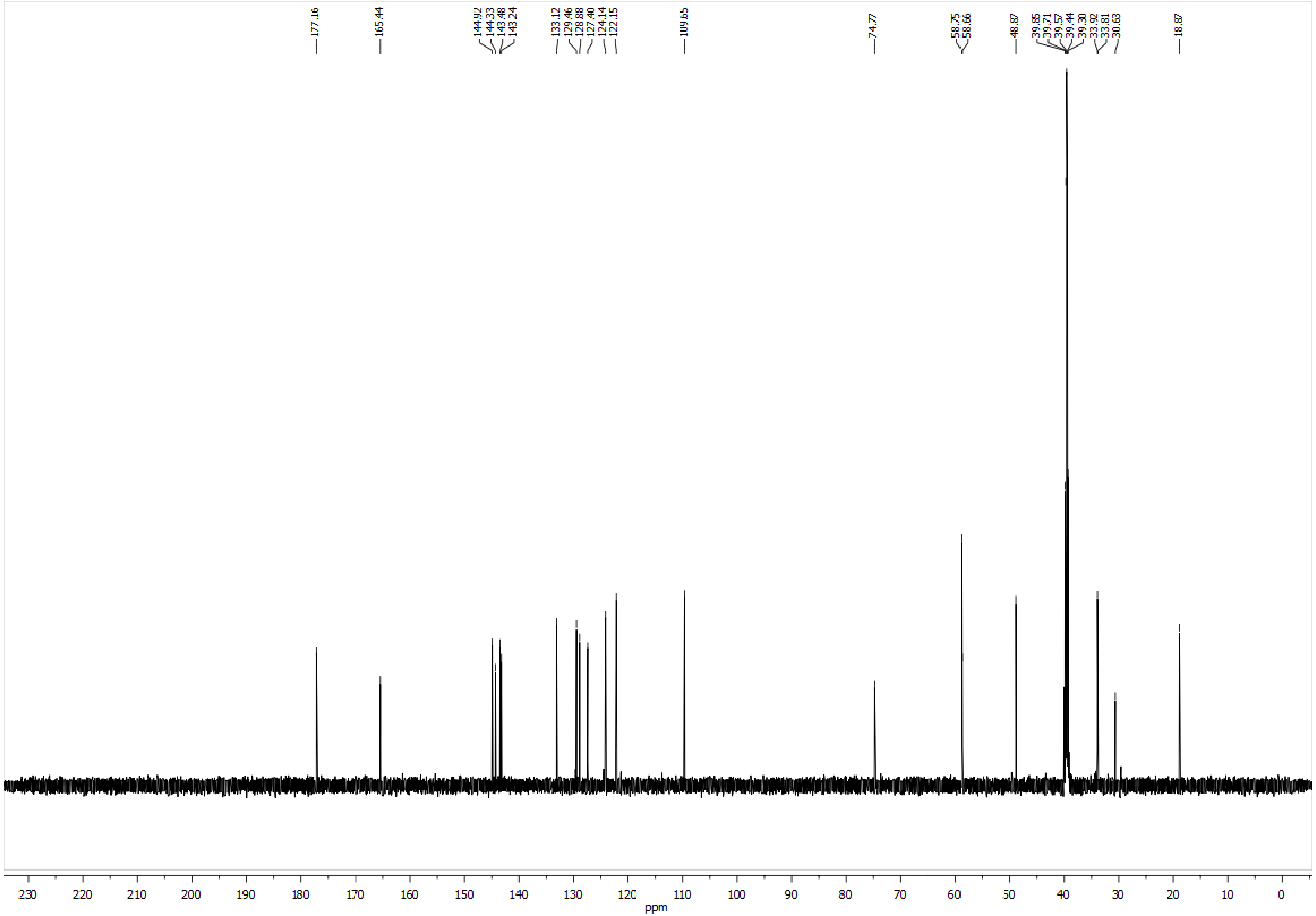

Figure S22. ^{13}C NMR spectra of the compound **13a** (DMSO-d_6)

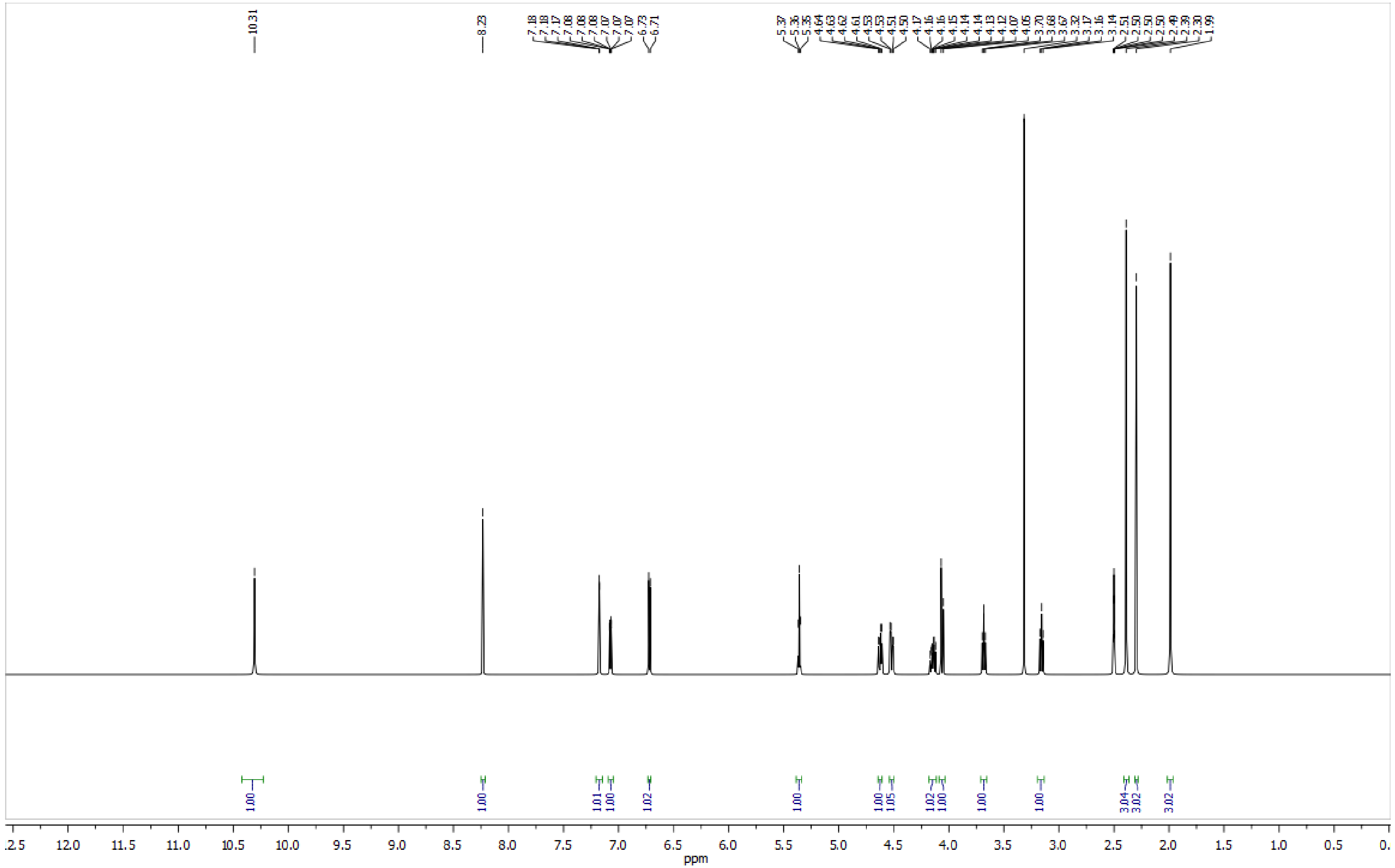

^{1}H NMR (600 MHz, DMSO-d_6) δ 7.14 – 7.05 (m, 1H), 6.72 (d, J = 7.9 Hz, 1H), 4.62 (dd, J = 13.5, 5.4 Hz, 1H), 4.52 (dd, J = 13.5, 5.4 Hz, 1H), 4.15 (ddd, J = 12.2, 9.5, 7.9 Hz, 1H), 4.06 (d, J = 12.2 Hz, 1H), 3.68 (t, J = 8.3 Hz, 1H), 3.16 (t, J = 9.1 Hz, 1H).

Figure S23. ^{1}H NMR spectra of the compound **13b** (DMSO-d_6)

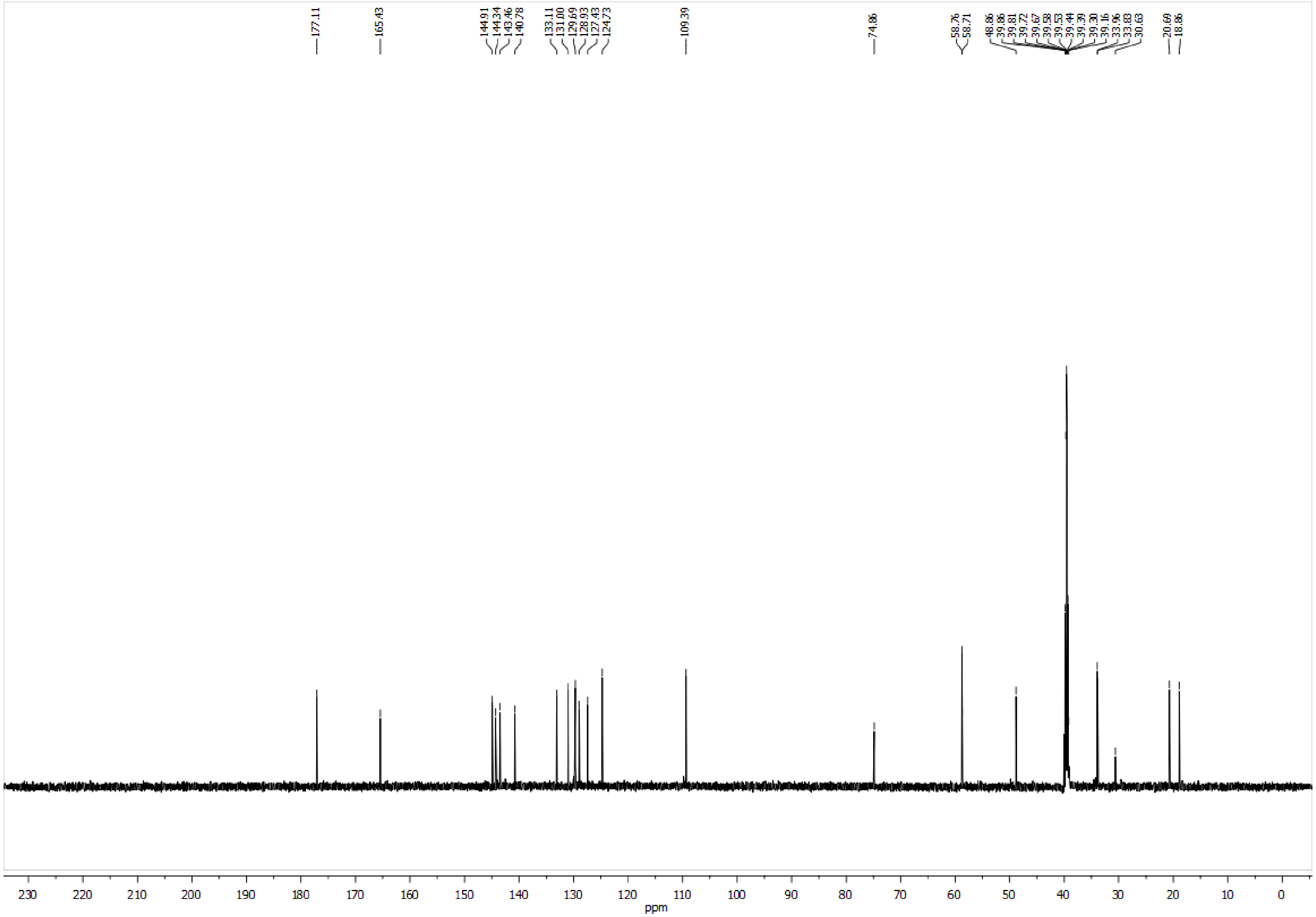

Figure S24. ^{13}C NMR spectra of the compound **13b** (DMSO-d_6)

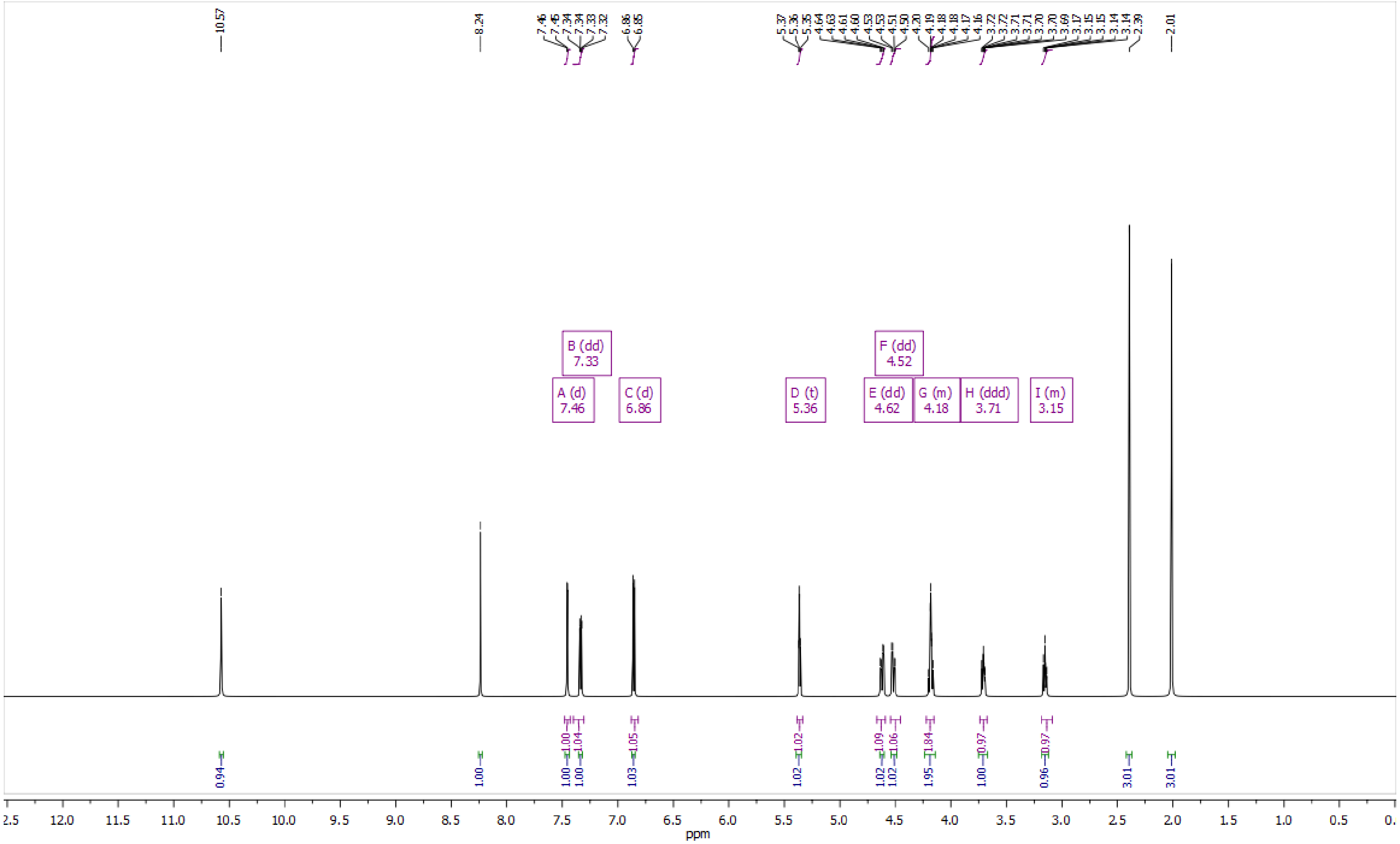

^{1}H NMR (600 MHz, DMSO-d_6) δ 7.46 (d, J = 2.2 Hz, 1H), 7.33 (dd, J = 8.3, 2.2 Hz, 1H), 6.86 (d, J = 8.3 Hz, 1H), 5.36 (t, J = 5.4 Hz, 1H), 4.62 (dd, J = 13.4, 5.4 Hz, 1H), 4.52 (dd, J = 13.4, 5.4 Hz, 1H), 4.22 – 4.15 (m, 2H), 3.71 (ddd, J = 8.4, 6.3, 1.9 Hz, 1H), 3.18 – 3.09 (m, 1H).

Figure S25. ^{1}H NMR spectra of the compound **13c** (DMSO-d_6)

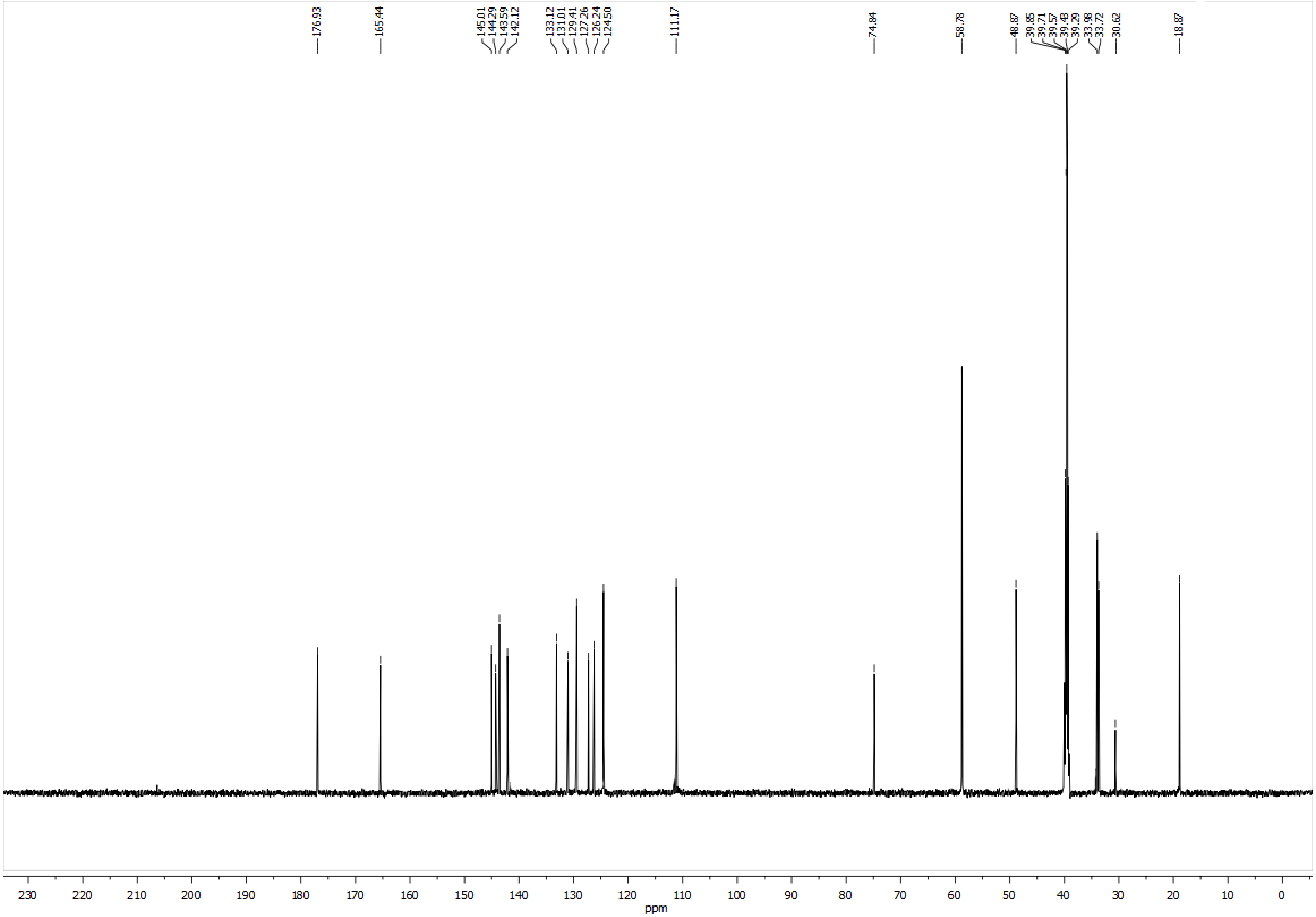

Figure S26. ^{13}C NMR spectra of the compound **13c** (DMSO-d_6)

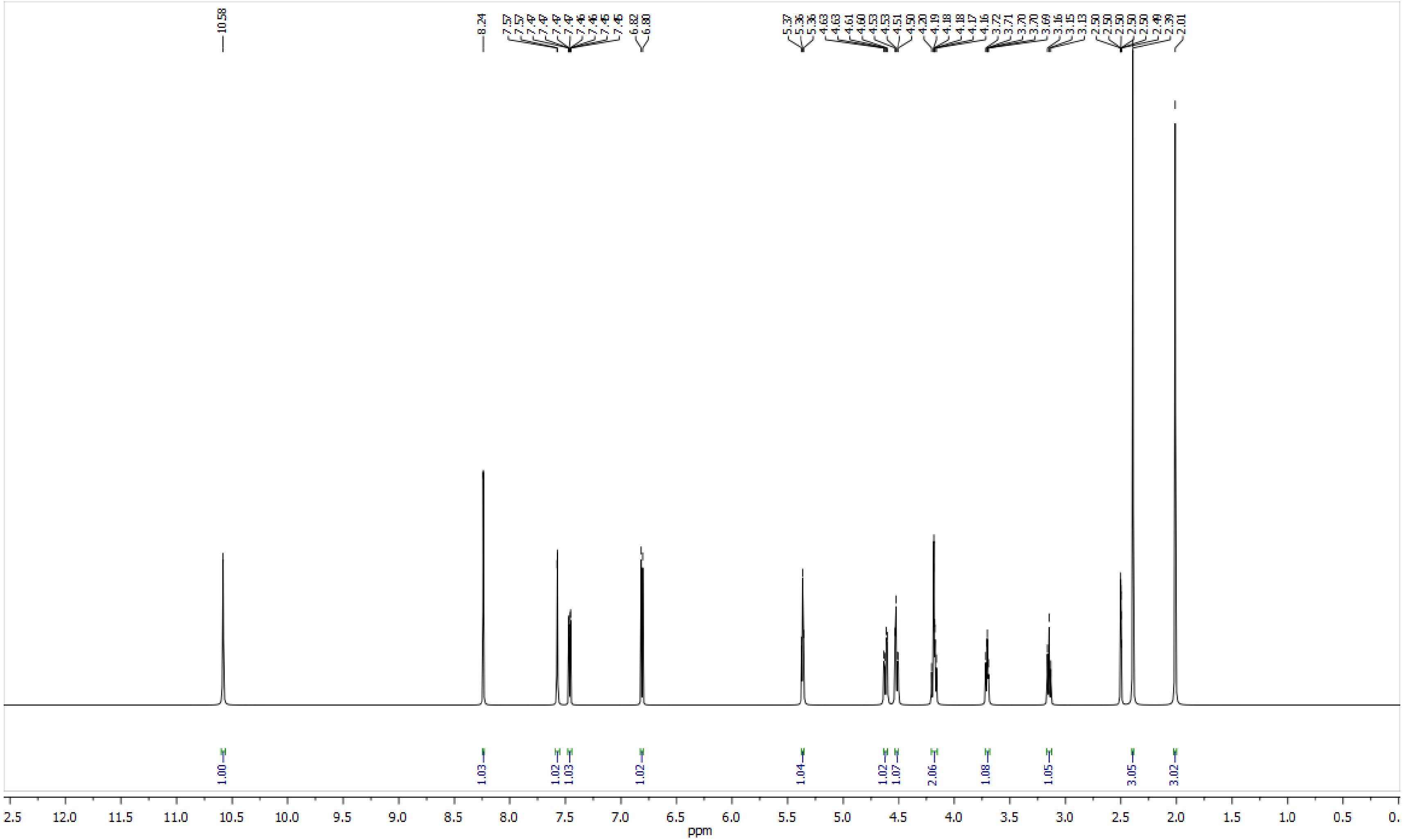

^{1}H NMR (600 MHz, DMSO-d_6) δ 7.57 (d, J = 2.1 Hz, 1H), 7.46 (ddd, J = 8.4, 2.2, 0.9 Hz, 1H), 6.81 (d, J = 8.2 Hz, 1H), 5.36 (t, J = 5.4 Hz, 1H), 4.62 (dd, J = 13.4, 5.3 Hz, 1H), 4.52 (dd, J = 13.6, 5.3 Hz, 1H), 4.23 – 4.16 (m, 2H), 3.73 – 3.69 (m, 1H), 3.15 (t, J = 8.8 Hz, 1H).

Figure S27. ^{1}H NMR spectra of the compound **13d** (DMSO-d_6)

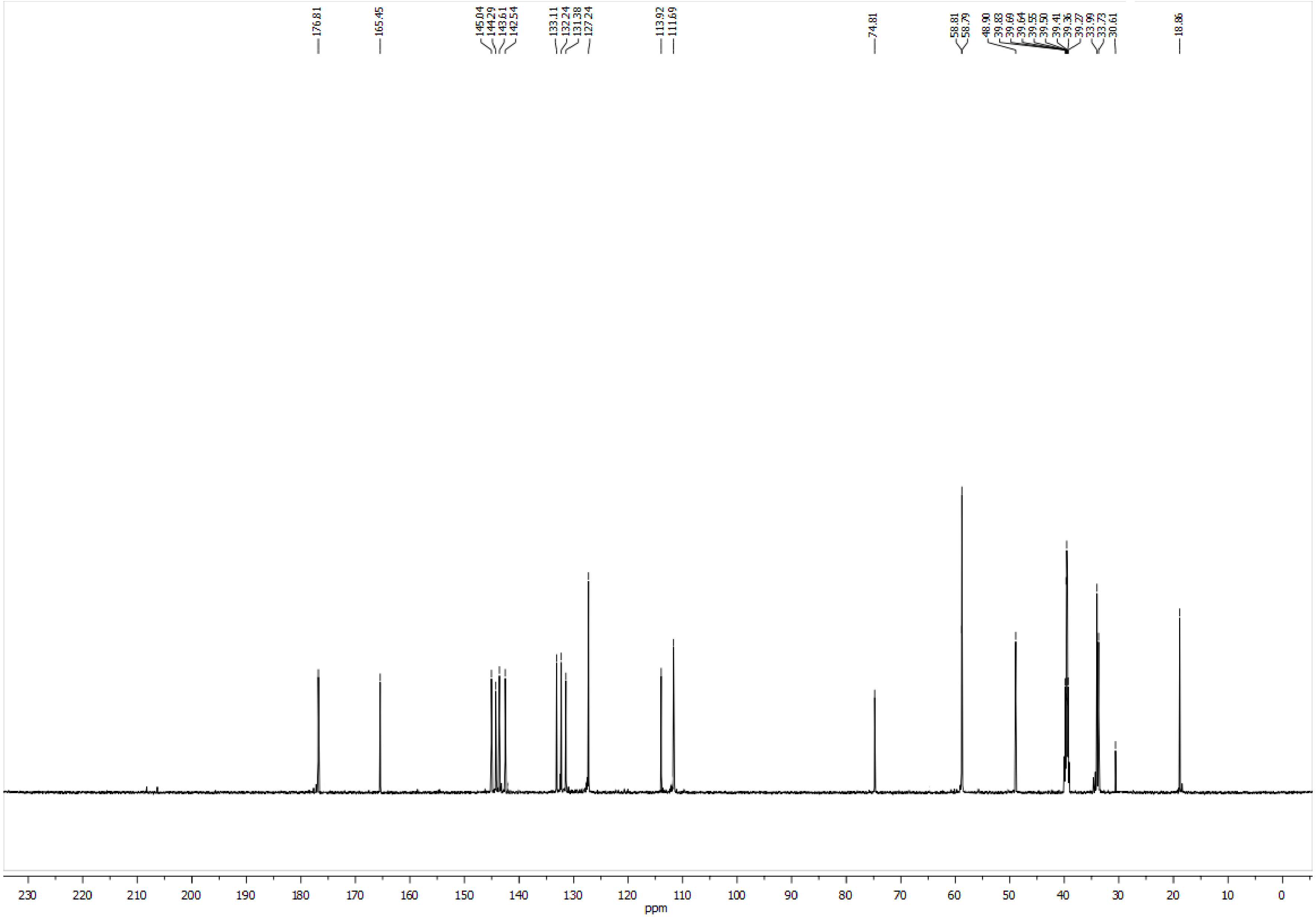

Figure S28.^{13}C NMR spectra of the compound **13d** (DMSO-d_6)

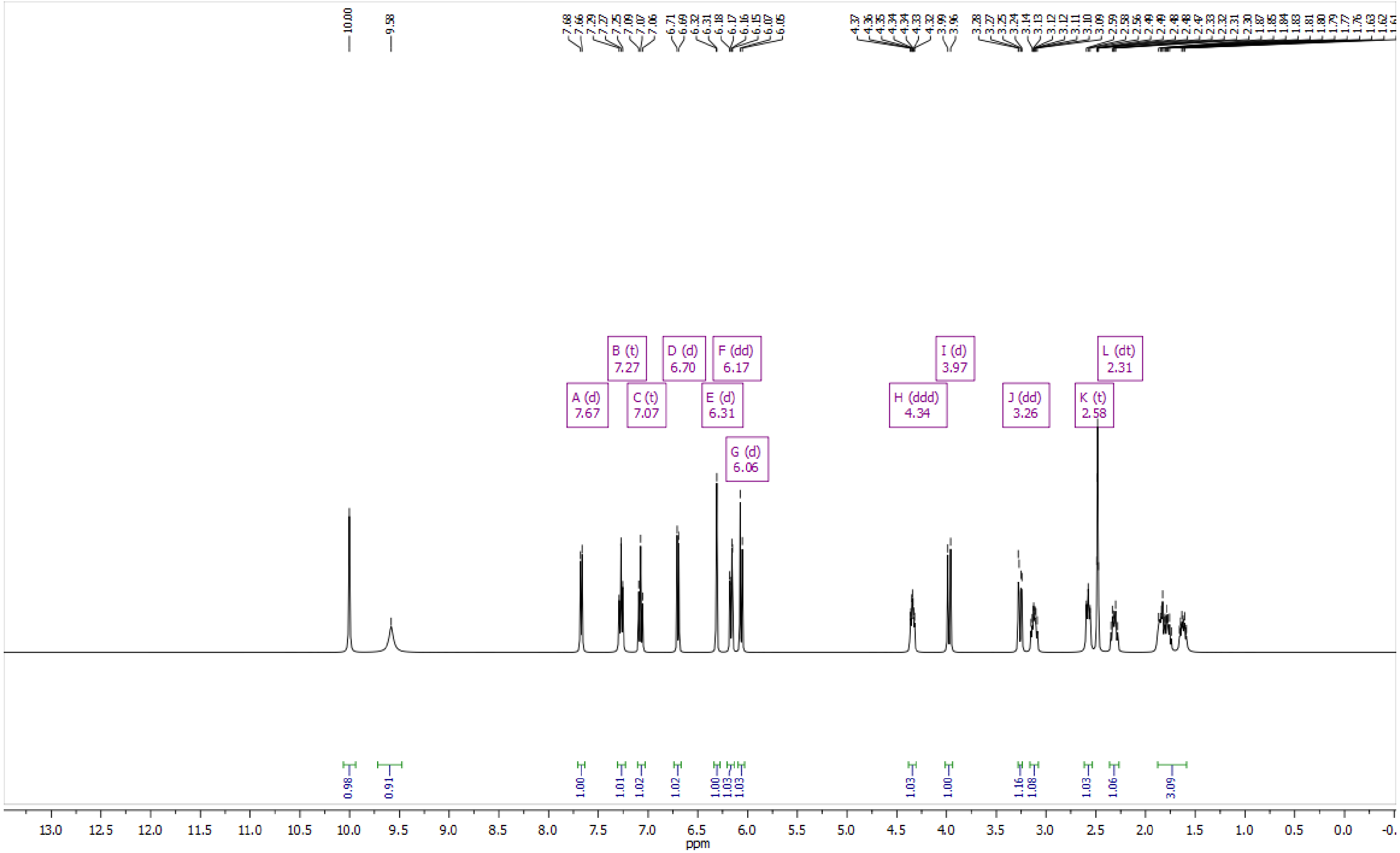

^{1}H NMR (400 MHz, DMSO-d_6) δ 7.67 (d, J = 7.5 Hz, 1H), 7.27 (t, J = 7.6 Hz, 1H), 7.07 (t, J = 7.5 Hz, 1H), 6.70 (d, J = 7.7 Hz, 1H), 6.31 (d, J = 2.4 Hz, 1H), 6.17 (dd, J = 8.4, 2.4 Hz, 1H), 6.06 (d, J = 8.4 Hz, 1H), 4.34 (ddd, J = 8.1, 6.4, 3.2 Hz, 1H), 3.97 (d, J = 11.3 Hz, 1H), 3.26 (dd, J = 11.2, 3.3 Hz, 1H), 2.58 (t, J = 7.1 Hz, 1H), 2.31 (dt, J = 12.9, 7.7 Hz, 1H).

Figure S29. ^{1}H NMR spectra of the compound **17a** (DMSO-d_6)

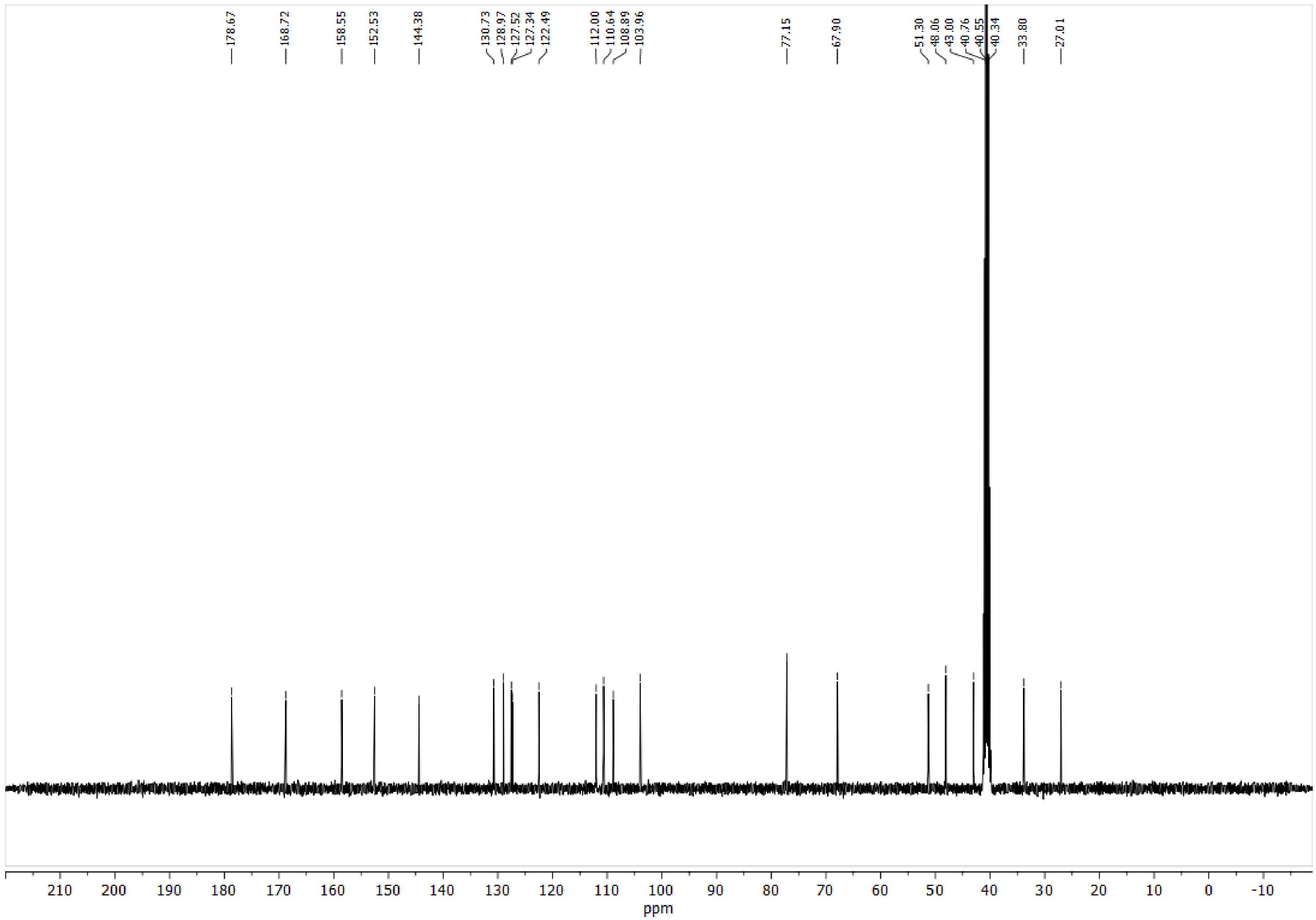

Figure S30. ^{13}C NMR spectra of the compound **17a** (DMSO-d_6)

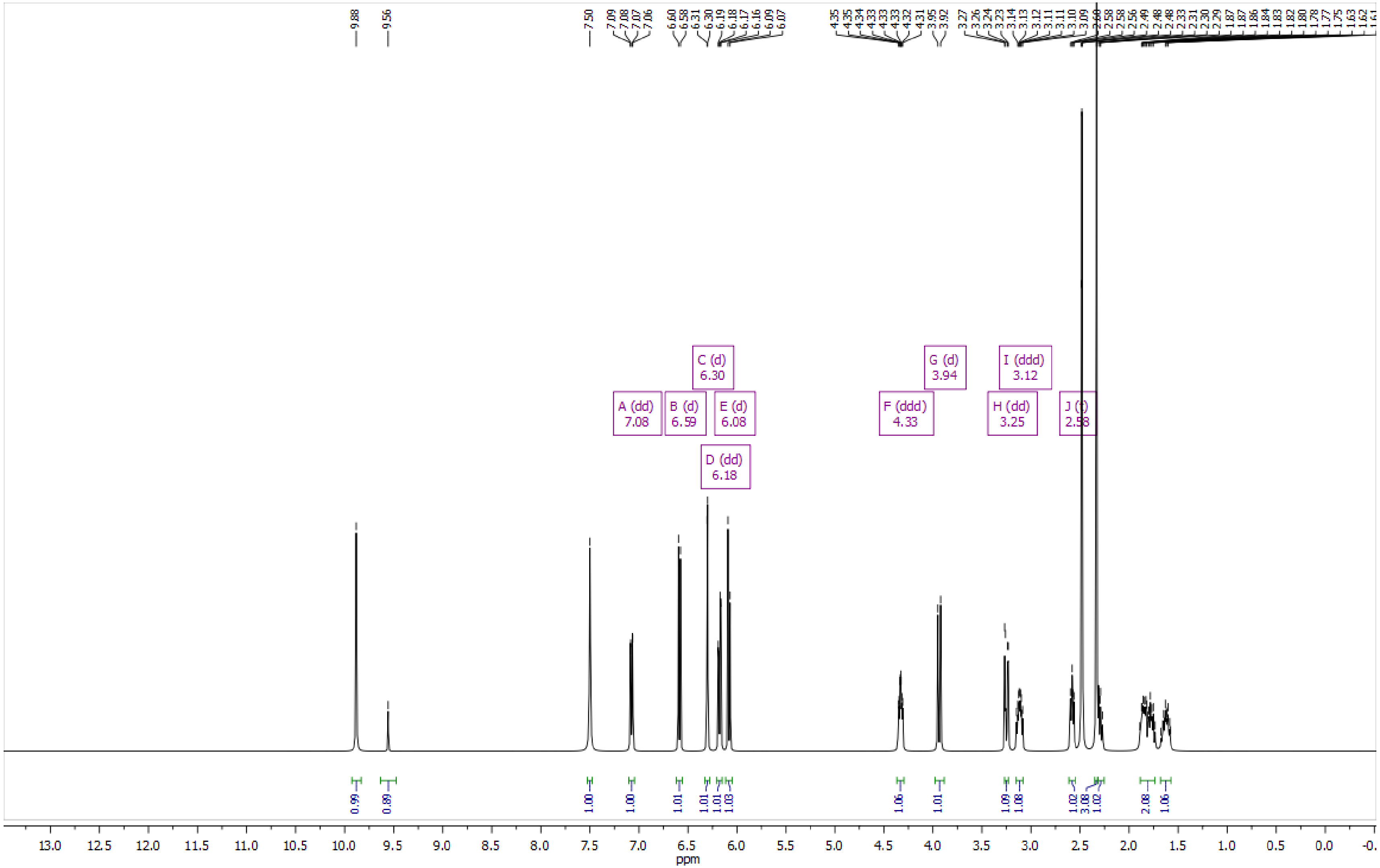

^{1}H NMR (400 MHz, DMSO-d_6) δ 7.08 (dd, J = 8.0, 1.6 Hz, 1H), 6.59 (d, J = 7.9 Hz, 1H), 6.30 (d, J = 2.4 Hz, 1H), 6.18 (dd, J = 8.4, 2.4 Hz, 1H), 6.08 (d, J = 8.4 Hz, 1H), 4.33 (ddd, J = 7.8, 6.4, 3.2 Hz, 1H), 3.94 (d, J = 11.3 Hz, 1H), 3.25 (dd, J = 11.2, 3.3 Hz, 1H), 3.12 (ddd, J = 10.7, 8.5, 5.1 Hz, 1H), 2.58 (t, J = 7.2 Hz, 1H).

Figure S31. ^{1}H NMR spectra of the compound **17b** (DMSO-d_6)

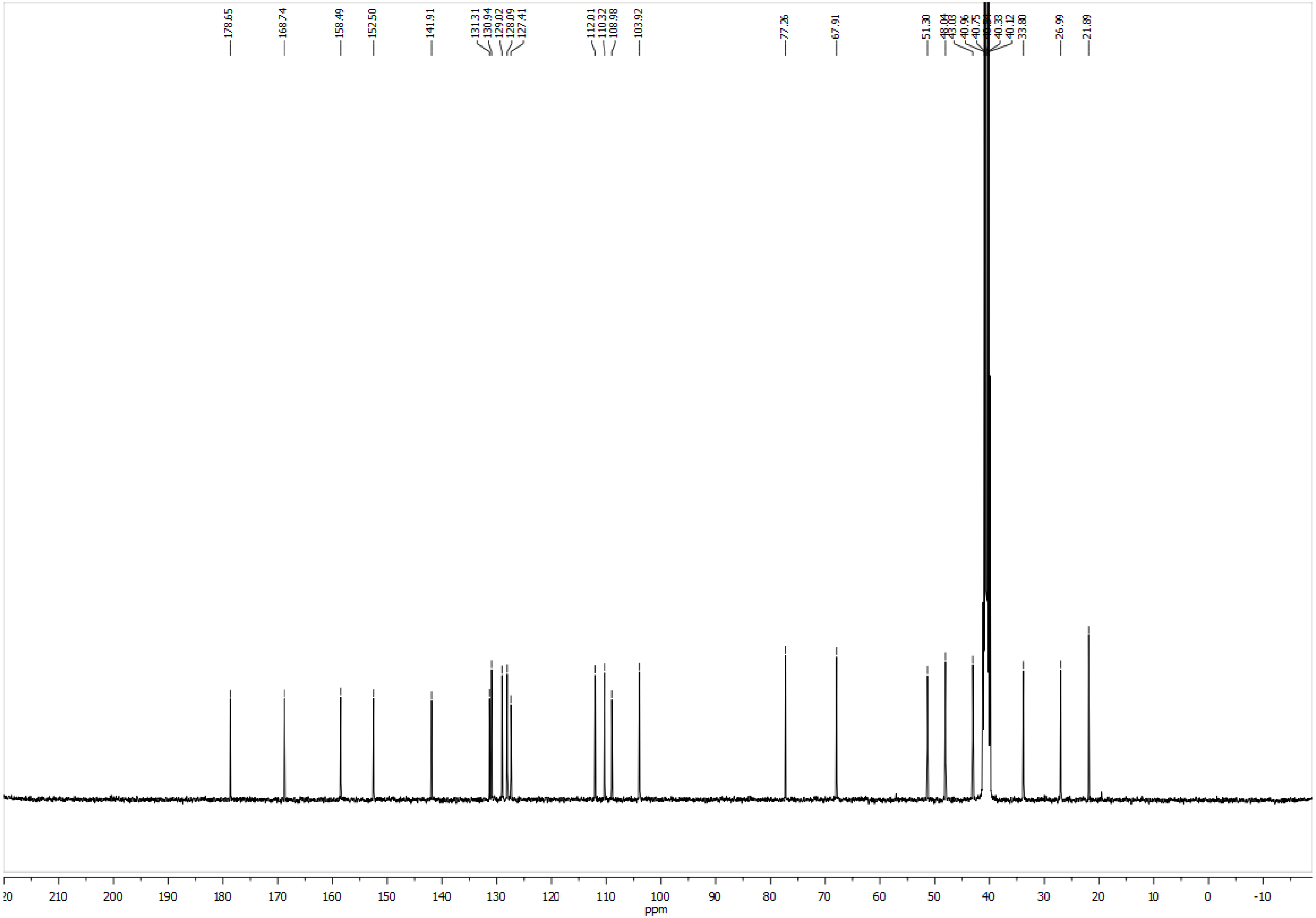

Figure S32.^{13}C NMR spectra of the compound **17b** (DMSO-d_6)

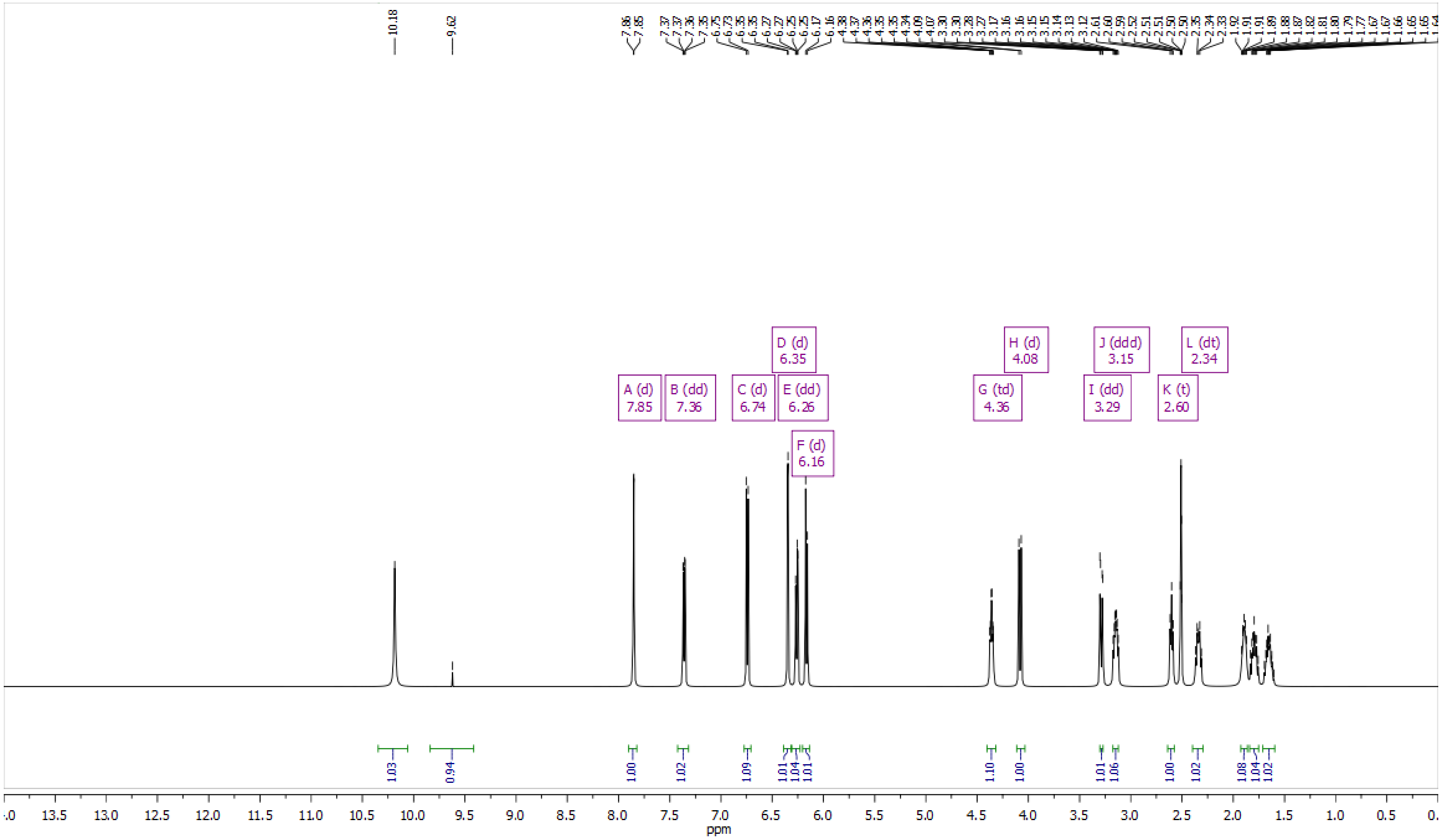

^{1}H NMR (500 MHz, DMSO-d_6) δ 7.85 (d, J = 2.2 Hz, 1H), 7.36 (dd, J = 8.3, 2.2 Hz, 1H), 6.74 (d, J = 8.3 Hz, 1H), 6.35 (d, J = 2.4 Hz, 1H), 6.26 (dd, J = 8.3, 2.5 Hz, 1H), 6.16 (d, J = 8.4 Hz, 1H), 4.36 (td, J = 7.3, 3.2 Hz, 1H), 4.08 (d, J = 11.3 Hz, 1H), 3.29 (dd, J = 11.2, 3.3 Hz, 1H), 3.15 (ddd, J = 10.7, 8.2, 5.1 Hz, 1H), 2.60 (t, J = 7.2 Hz, 1H), 2.34 (dt, J = 14.3, 7.6 Hz, 1H).

Figure S33. ^{1}H NMR spectra of the compound **17c** (DMSO-d_6)

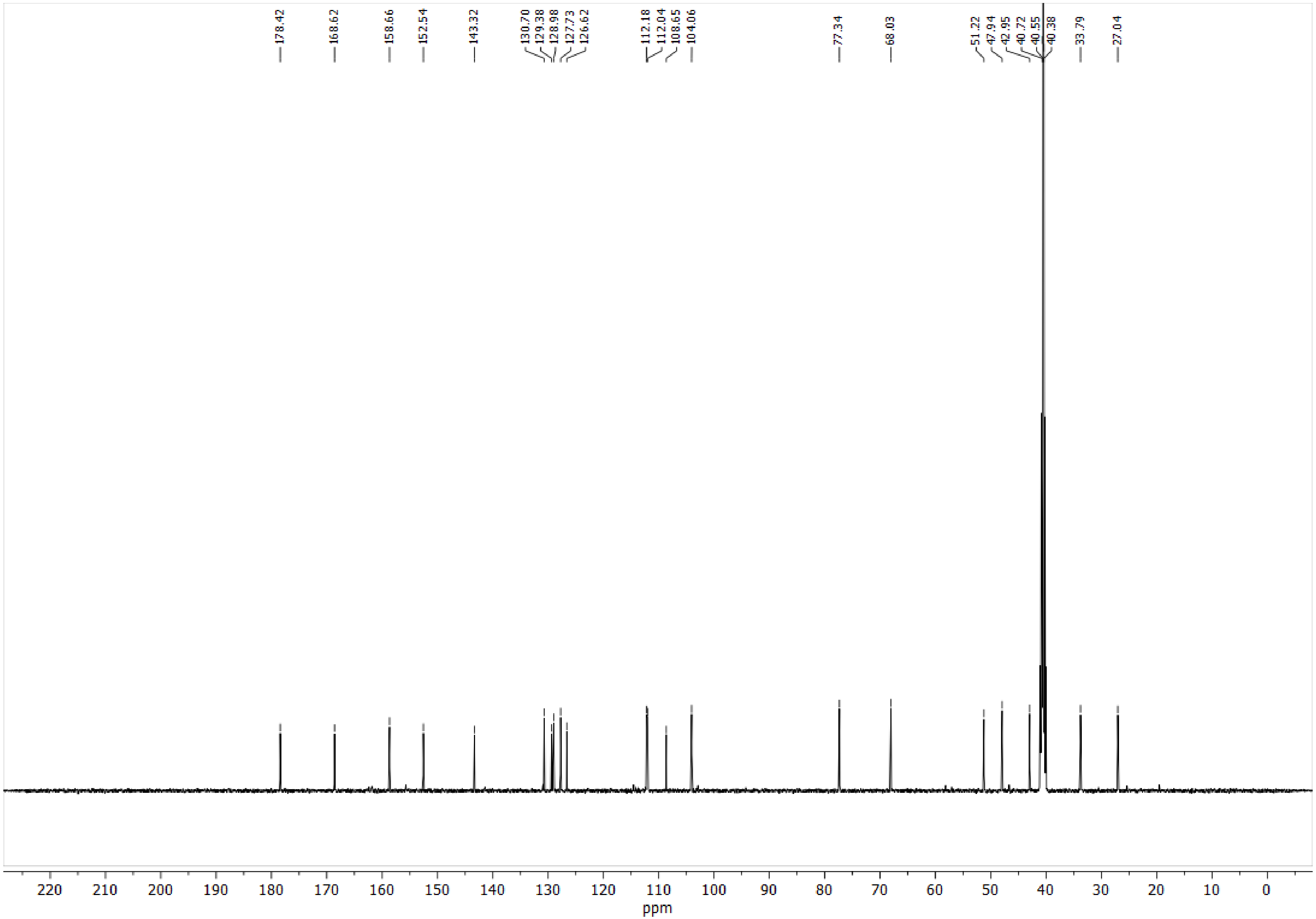

Figure S34. ^{13}C NMR spectra of the compound **17c** (DMSO-d_6)

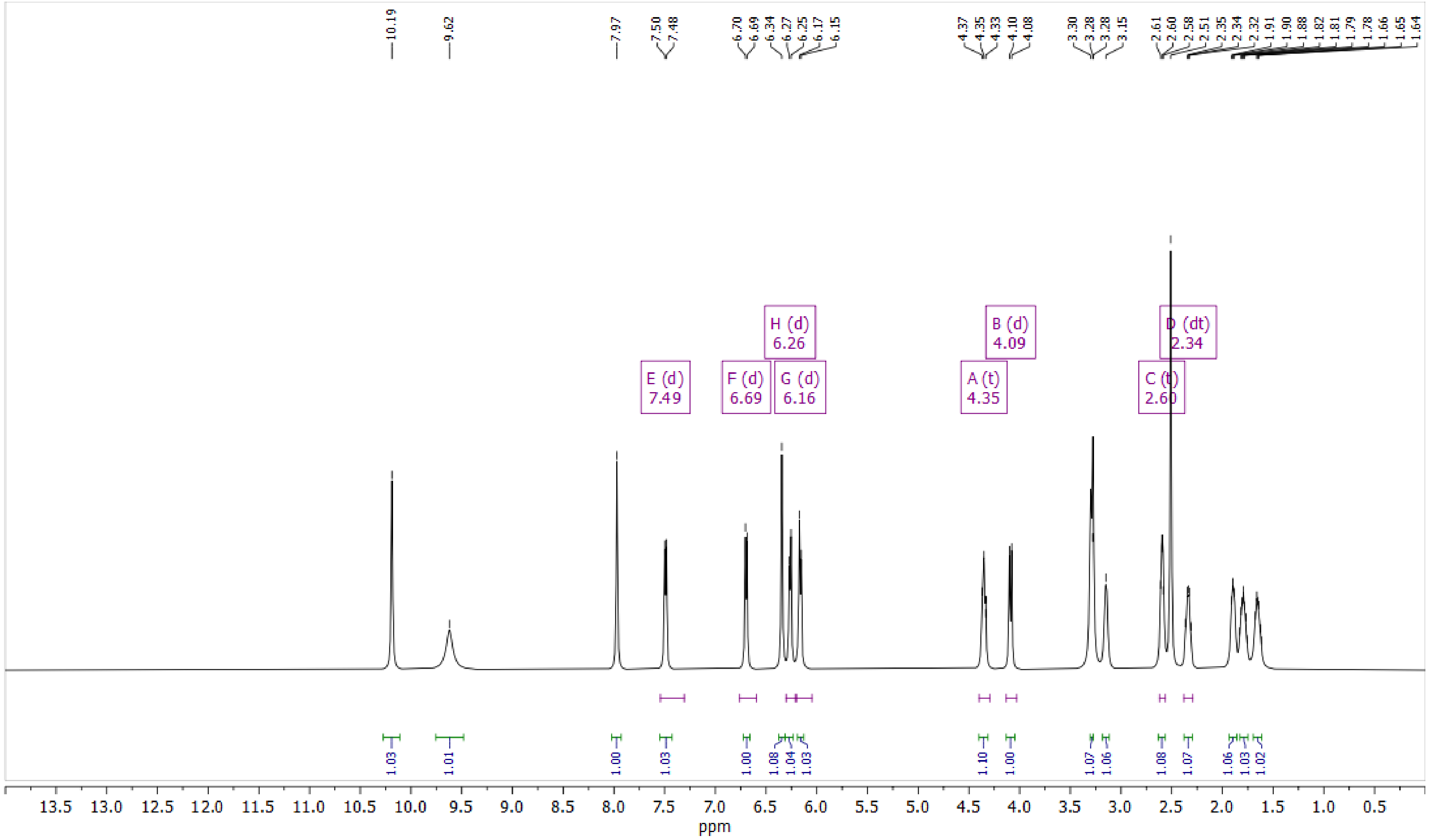

^{1}H NMR (500 MHz, DMSO-d_6) δ 7.49 (d, J = 8.3 Hz, 1H), 6.69 (d, J = 8.2 Hz, 1H), 6.26 (d, J = 8.3 Hz, 1H), 6.16 (d, J = 8.4 Hz, 1H), 4.35 (t, J = 8.5 Hz, 1H), 4.09 (d, J = 11.1 Hz, 1H), 2.60 (t, J = 6.6 Hz, 1H), 2.34 (dt, J = 13.9, 8.0 Hz, 1H).

Figure S35. ^{1}H NMR spectra of the compound **17d** (DMSO-d_6)

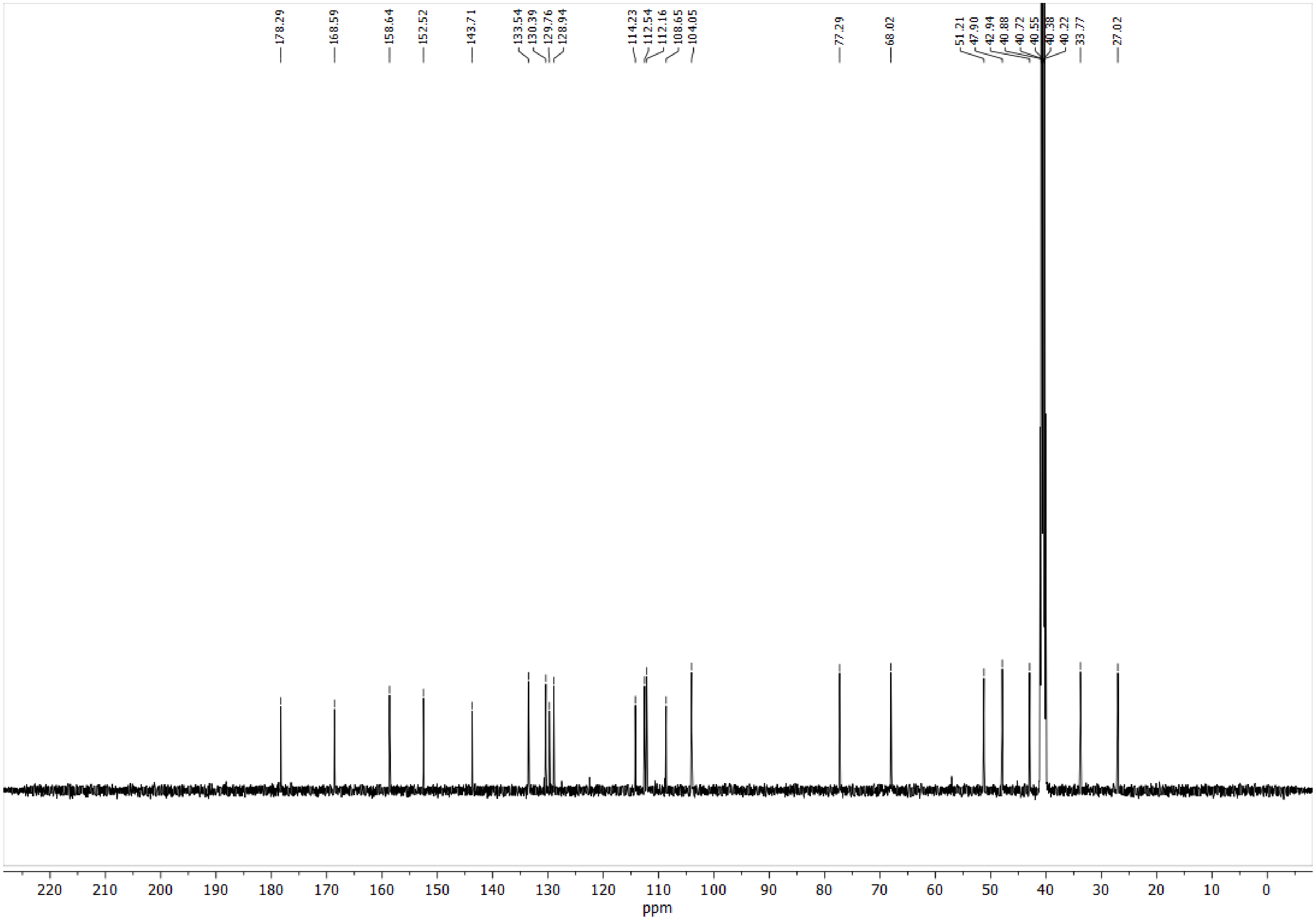

Figure S36. ^{13}C NMR spectra of the compound **17d** (DMSO-d_6)

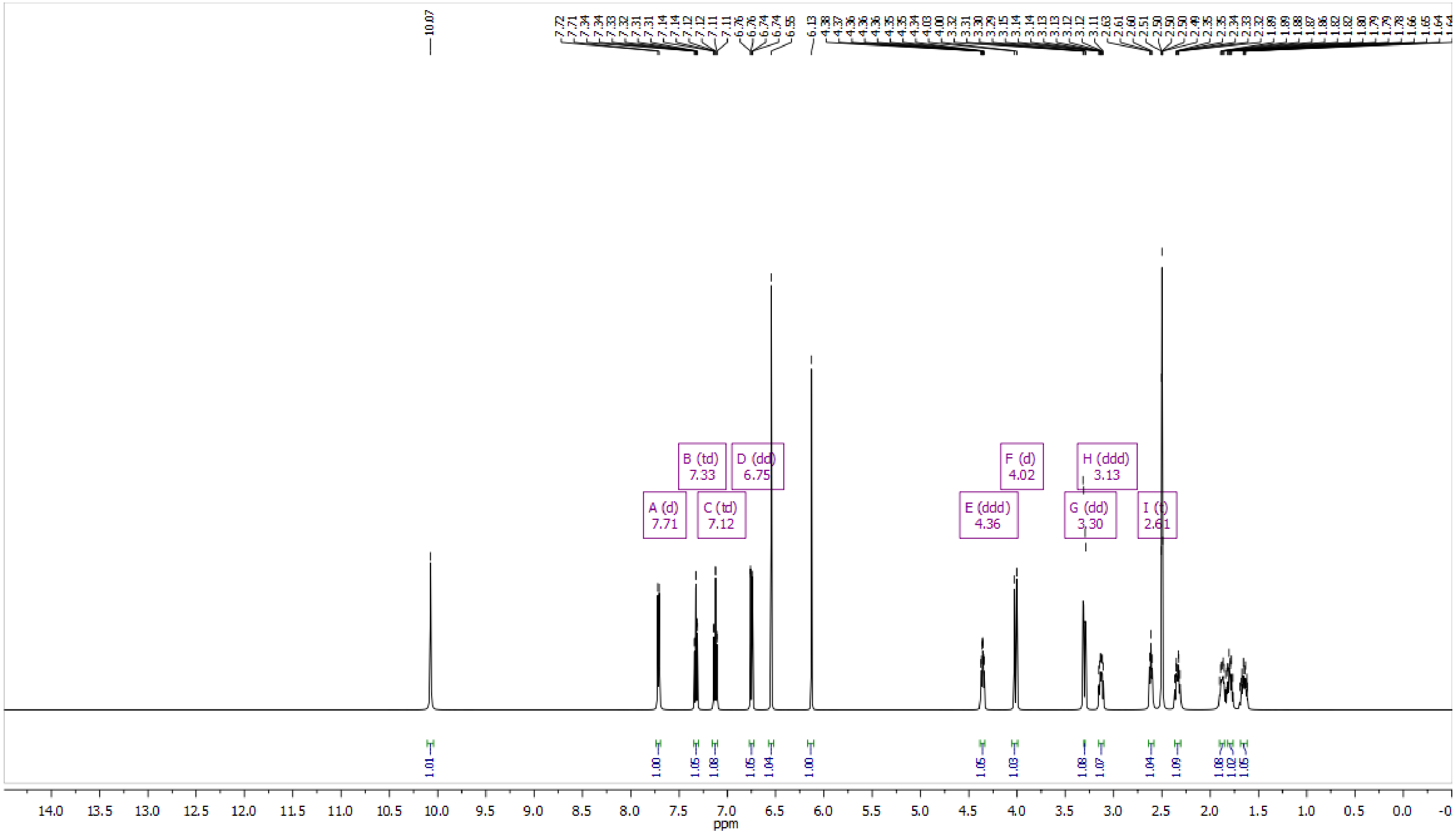

^{1}H NMR (500 MHz, DMSO-d_6) δ 7.71 (d, J = 7.4 Hz, 1H), 7.33 (td, J = 7.7, 1.2 Hz, 1H), 7.12 (td, J = 7.6, 1.1 Hz, 1H), 6.75 (dd, J = 7.8, 1.1 Hz, 1H), 4.36 (ddd, J = 8.0, 6.5, 3.3 Hz, 1H), 4.02 (d, J = 11.3 Hz, 1H), 3.30 (dd, J = 11.2, 3.3 Hz, 1H), 3.13 (ddd, J = 10.7, 8.4, 5.1 Hz, 1H), 2.61 (t, J = 7.3 Hz, 1H).

Figure S37. ^{1}H NMR spectra of the compound **18** (DMSO-d_6)

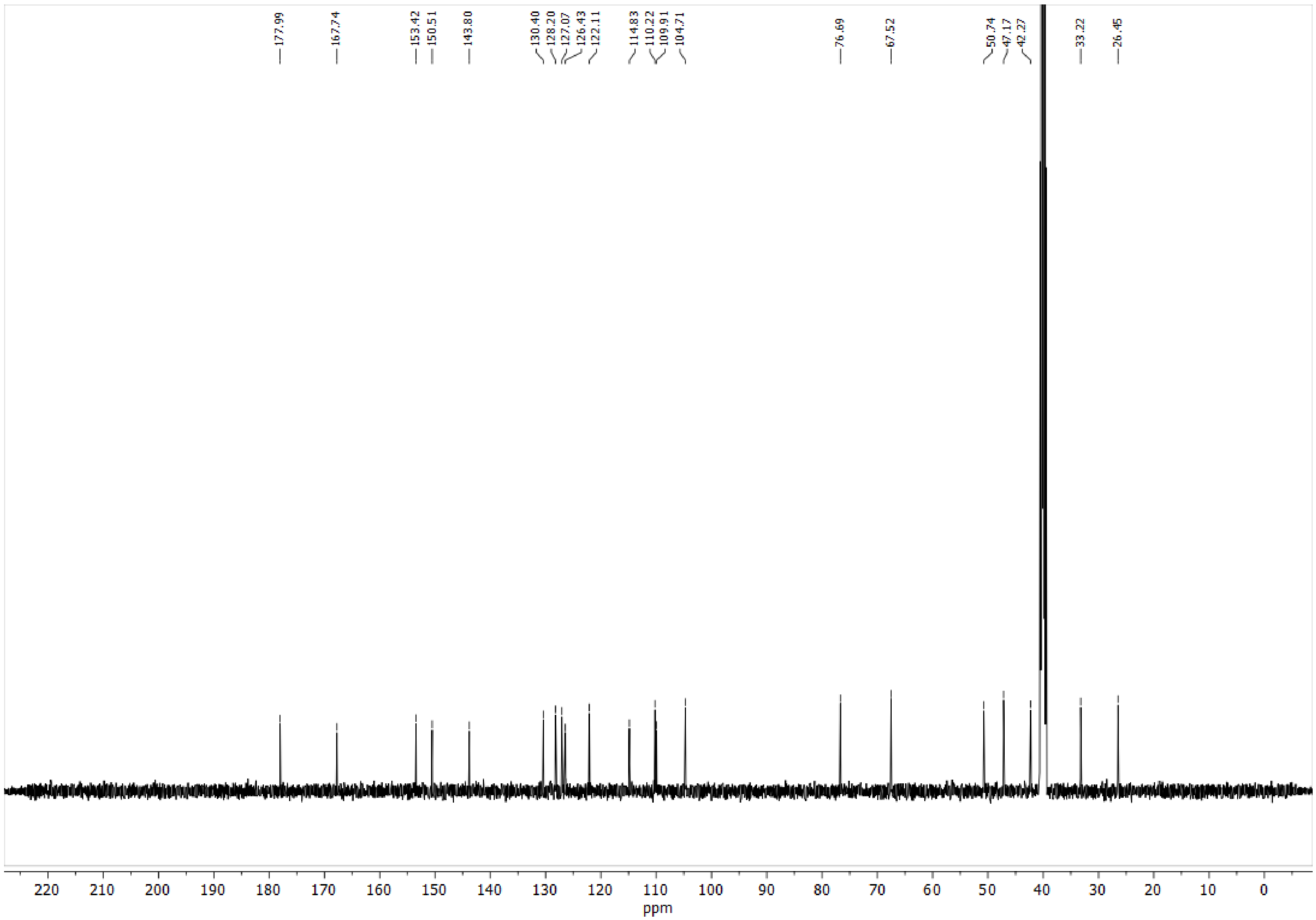

Figure S38. ^{13}C NMR spectra of the compound **18** (DMSO-d_6)

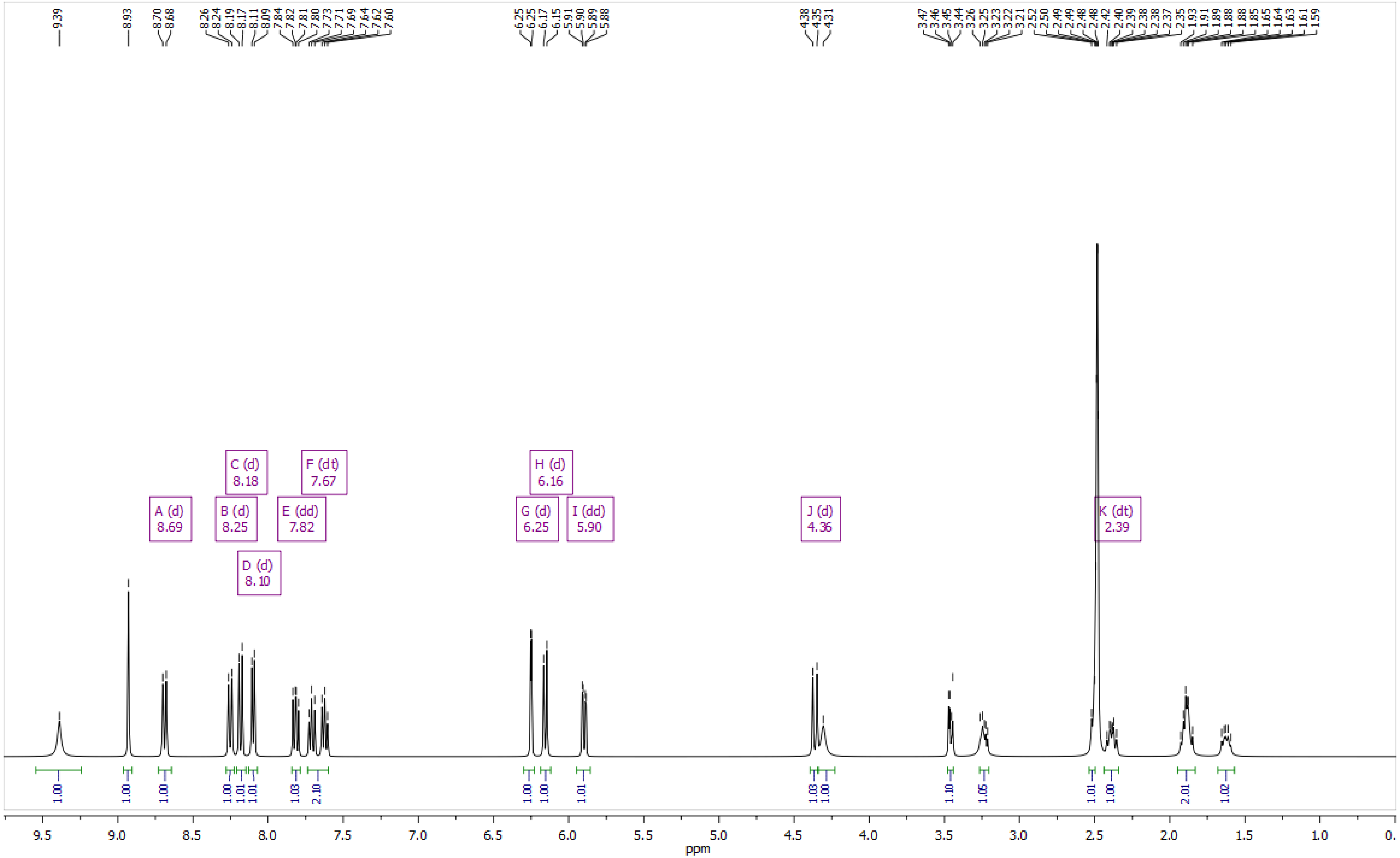

^{1}H NMR (400 MHz, DMSO-d_6) δ 8.69 (d, *J* = 8.5 Hz, 1H), 8.25 (d, *J* = 8.5 Hz, 1H), 8.18 (d, *J* = 8.7 Hz, 1H), 8.10 (d, *J* = 6.7 Hz, 1H), 7.82 (dd, *J* = 8.7, 6.7 Hz, 1H), 7.67 (dt, *J* = 34.4, 7.2 Hz, 2H), 6.25 (d, *J* = 2.4 Hz, 1H), 6.16 (d, *J* = 8.4 Hz, 1H), 5.90 (dd, *J* = 8.4, 2.4 Hz, 1H), 4.36 (d, *J* = 11.4 Hz, 1H), 2.39 (dt, *J* = 11.4, 7.6 Hz, 1H).

Figure S39. ^{1}H NMR spectra of the compound **19** (DMSO-d_6)

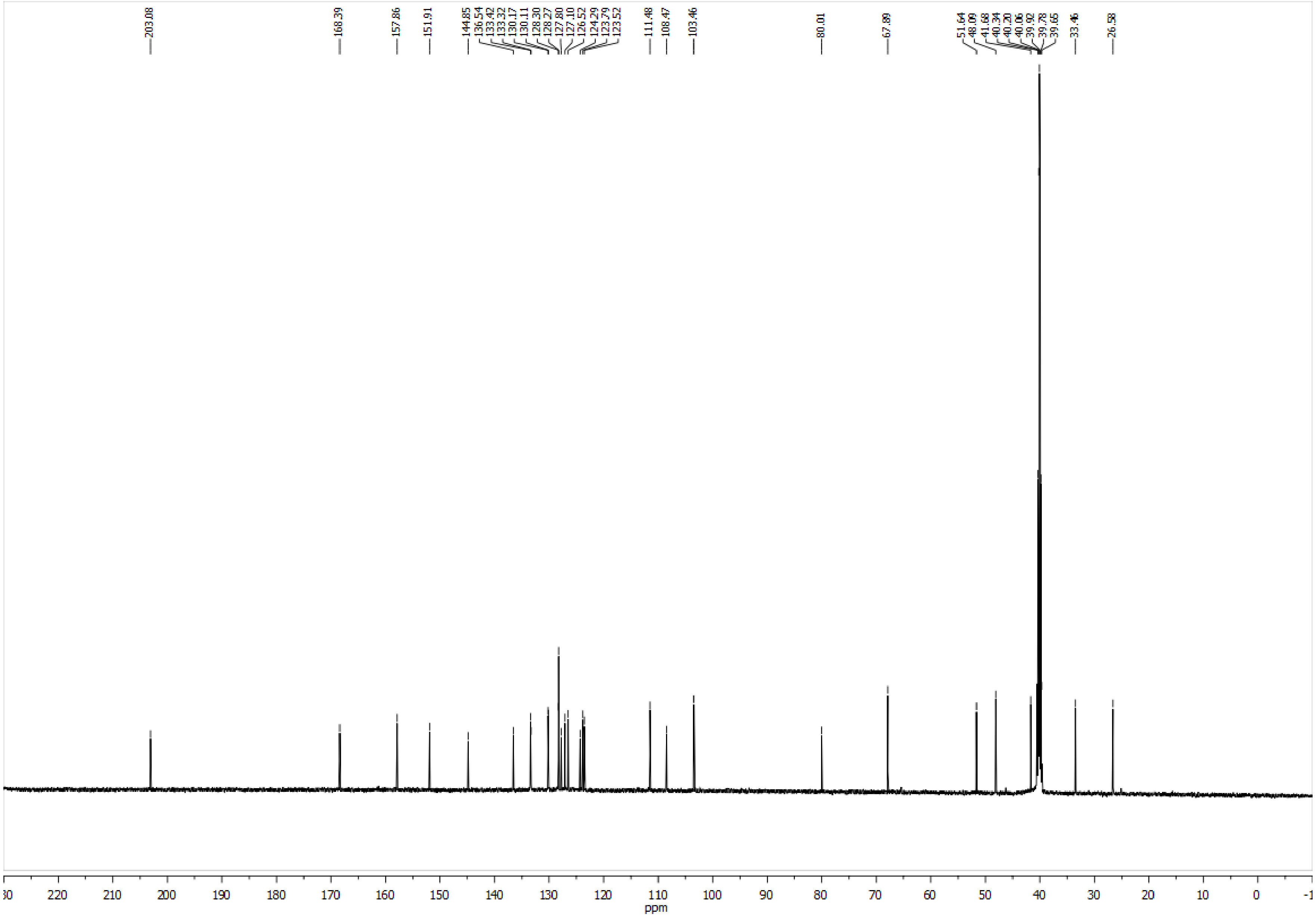

Figure S40.^{13}C NMR spectra of the compound **19** (DMSO-d_6)

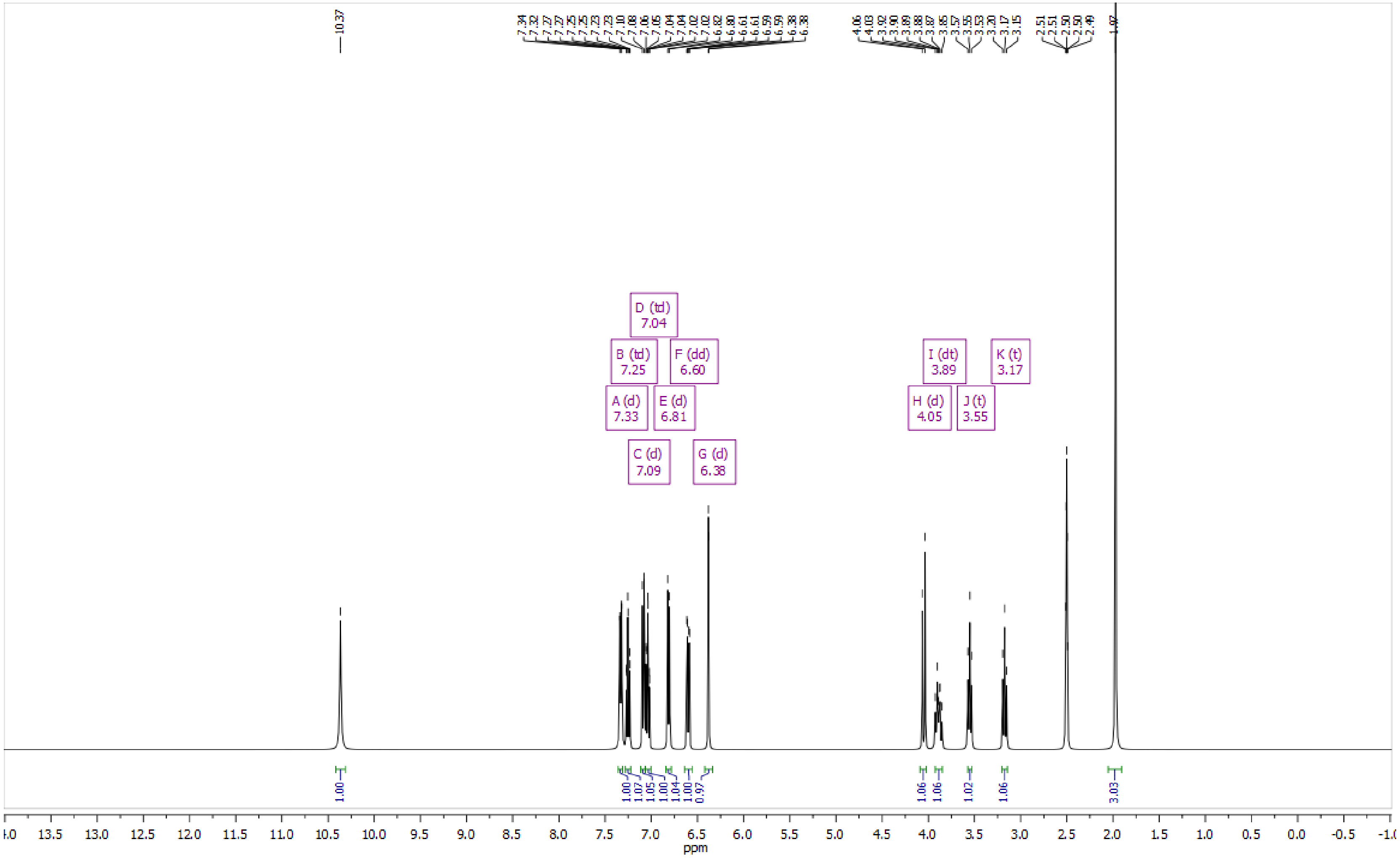

^{1}H NMR (400 MHz, DMSO-d_6) δ 7.33 (d, J = 7.3 Hz, 1H), 7.25 (td, J = 7.7, 1.3 Hz, 1H), 7.09 (d, J = 8.4 Hz, 1H), 7.04 (td, J = 7.5, 1.0 Hz, 1H), 6.81 (d, J = 7.7 Hz, 1H), 6.60 (dd, J = 8.3, 2.4 Hz, 1H), 6.38 (d, J = 2.4 Hz, 1H), 4.05 (d, J = 12.3 Hz, 1H), 3.89 (dt, J = 12.4, 8.4 Hz, 1H), 3.55 (t, J = 8.1 Hz, 1H), 3.17 (t, J = 8.8 Hz, 1H).

Figure S41. ^{1}H NMR spectra of the compound **20a** (DMSO-d_6)

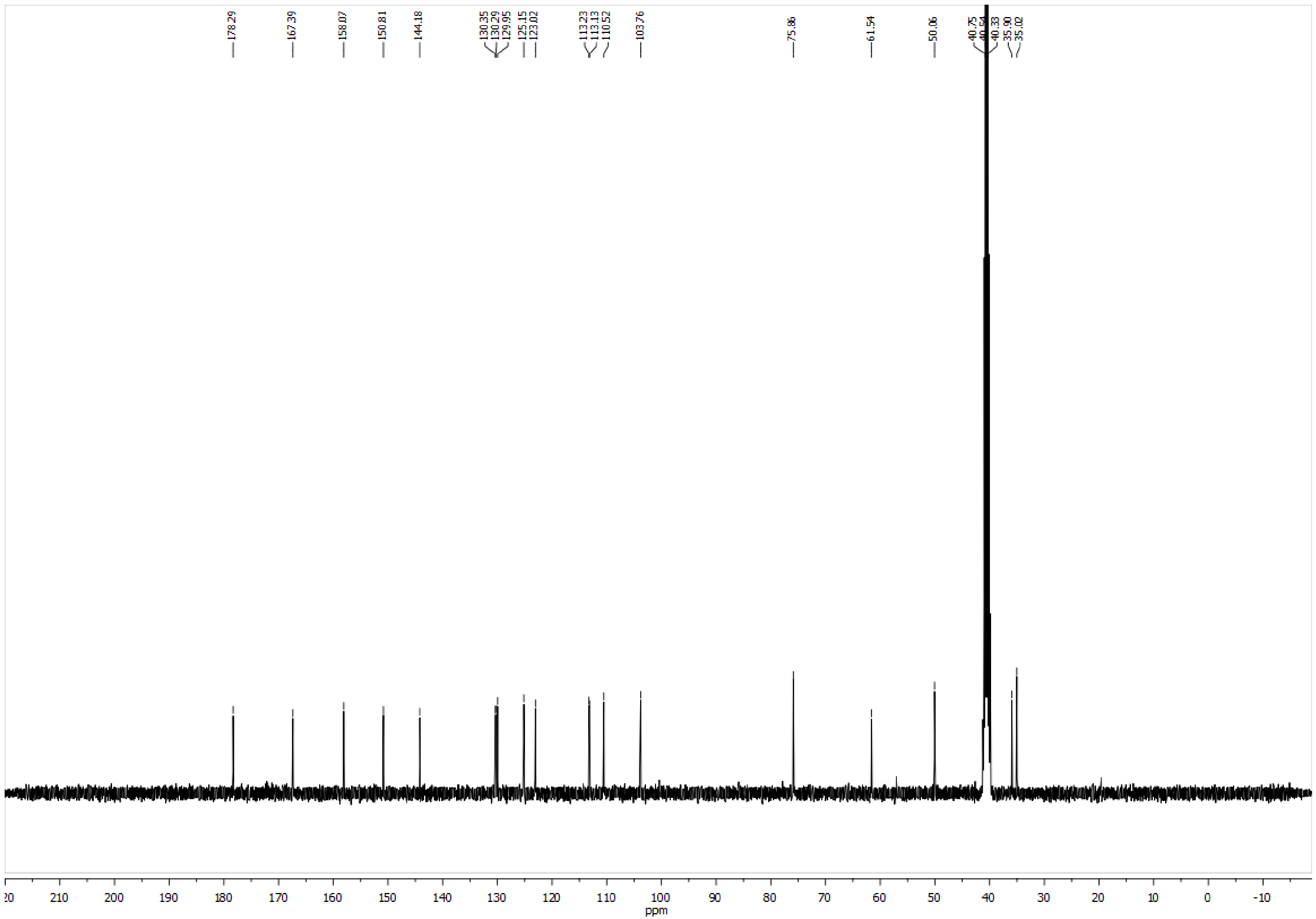

Figure S42. ^{13}C NMR spectra of the compound **20a** (DMSO-d_6)

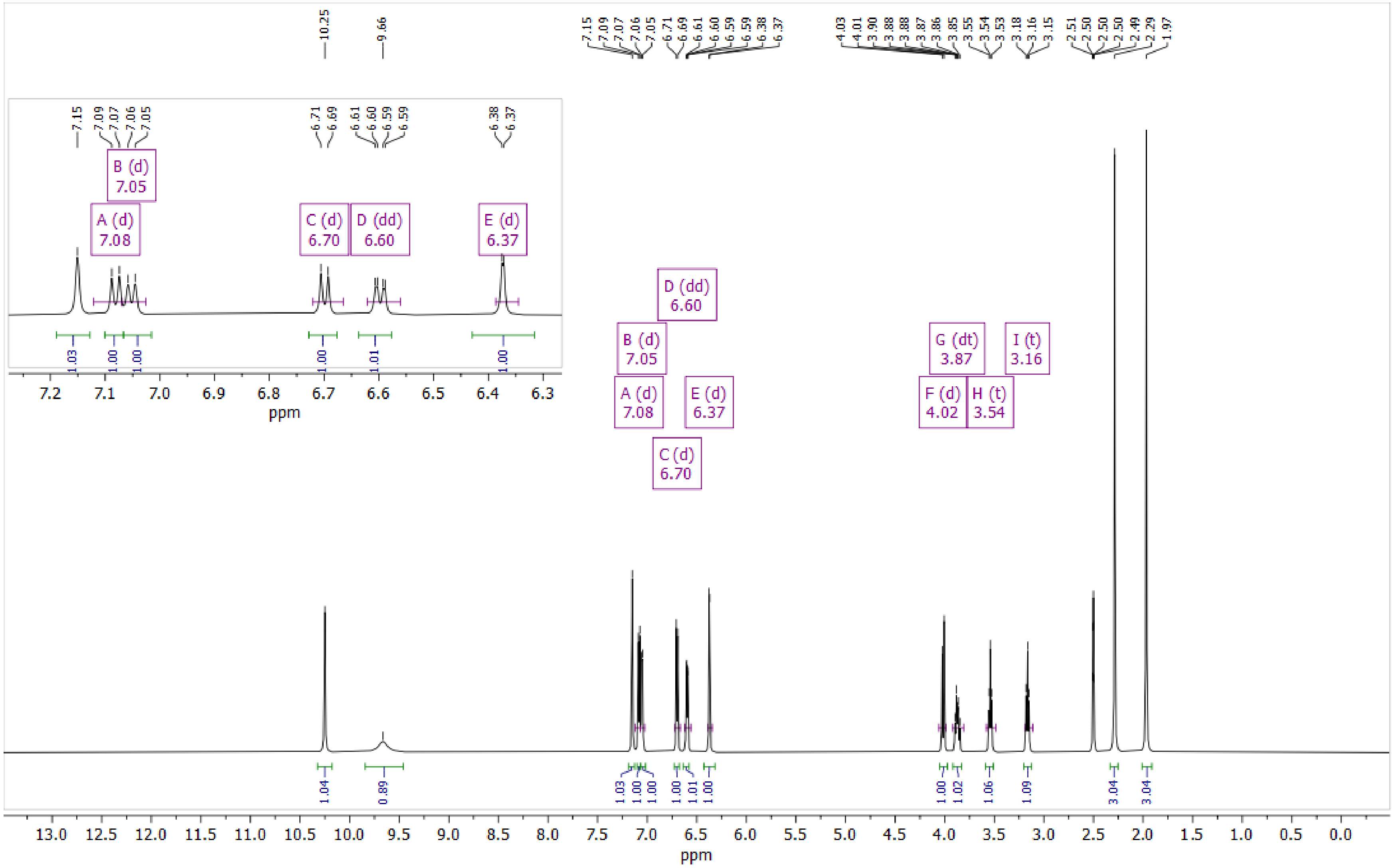

^{1}H NMR (600 MHz, DMSO-d_6) δ 7.08 (d, J = 8.4 Hz, 1H), 7.05 (d, J = 7.8 Hz, 1H), 6.70 (d, J = 7.8 Hz, 1H), 6.60 (dd, J = 8.3, 2.4 Hz, 1H), 6.37 (d, J = 2.4 Hz, 1H), 4.02 (d, J = 12.3 Hz, 1H), 3.87 (dt, J = 12.4, 8.5 Hz, 1H), 3.54 (t, J = 8.1 Hz, 1H), 3.16 (t, J = 8.8 Hz, 1H).

Figure S43.^{1}H NMR spectra of the compound **20b** (DMSO-d_6)

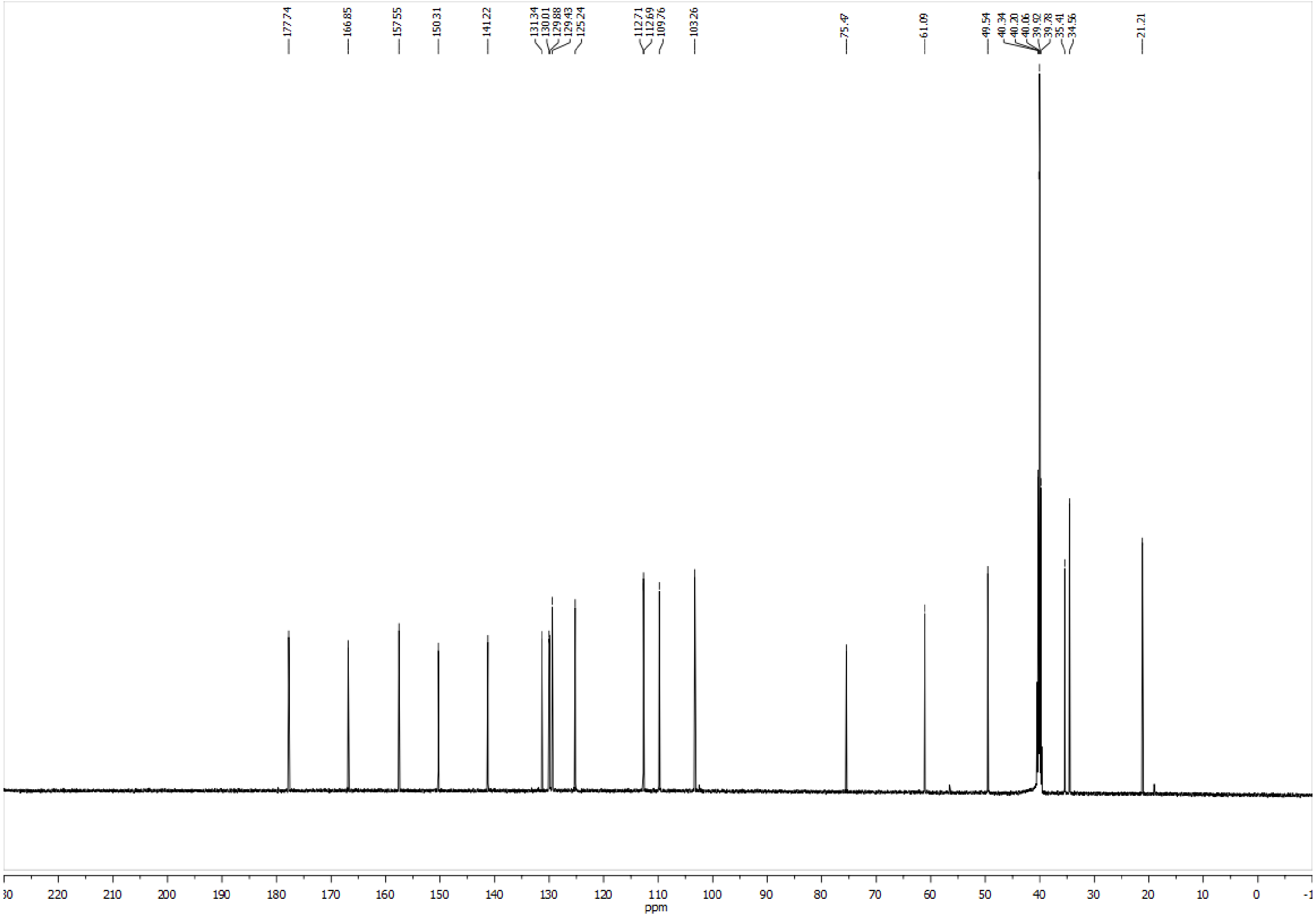

Figure S44.^{13}C NMR spectra of the compound **20b** (DMSO-d_6)

3'-(5-(hydroxymethyl)-8-methyl-2-oxo-2*H*-pyrano[2,3-*c*]pyridin-3-yl)spiro[indene-2,2'-piperidine]-1,3-dione 6a

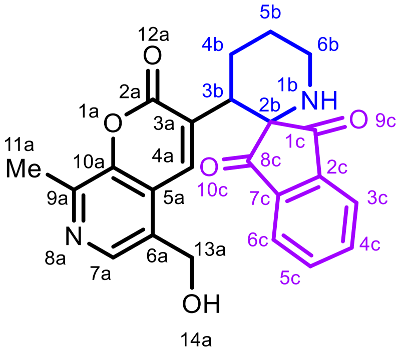

^{1}H NMR (500 MHz, DMSO-d_6): δ 1.674 and 1.834 (m, 2H, H5b), 1.838 and 2.365 (m, 2H, H4b), 2.385 (s, 3H, H11a), 2.911 and 3.329 (m, 2H, H6b), 3.414 (dd, *J* = 12.6 Hz, *J* = 3.2 Hz, 1H, H3b), 4.508 and 4.550 (dd, *J* = 12.54 Hz, *J* = 5.2 Hz, 2H, H13a), 5.380 (tr, *J* = 5.2 Hz, 1H, H14a), 7.780 (s, 1H, H4a), 7.781 (m, 1H, H6c), 7.787 (m, 1H, H4c), 7.809 (m, 1H, H3c), 7.810 (m, 1H, H5c), 8.179 (s, 1H, H7a).

^{13}C{^{1}H} NMR (DMSO-d_6, 126 MHz) δ 200.9 (C8c), 199.0 (C1c), 158.7 (C2a), 145.6 (C9a), 145.6 (C10a), 142.3 (C7a), 140.7 (C2c), 139.6 (C7c), 136.6 (C4c), 136.2 (C5c), 135.3 (C4a), 132.5 (C3a), 130.1 (C5a), 123.2 (C3c), 122.7 (C6c), 121.3 (C6a), 65.8 (C2b), 57.9 (C13a), 40.5 (C6b), 40.2 (C3b), 25.7 (C4b), 23.3 (C5b), 18.1 (C11a).

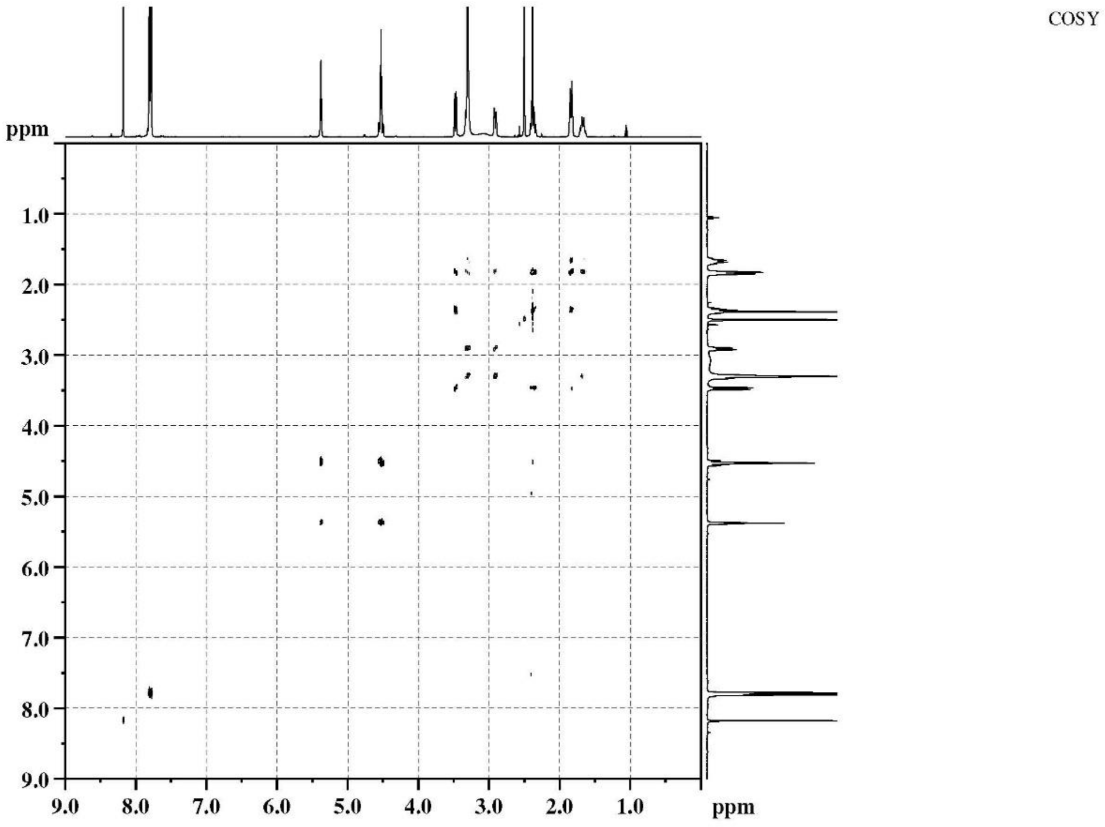

Figure S45. ¹H- ¹H COSY correlations

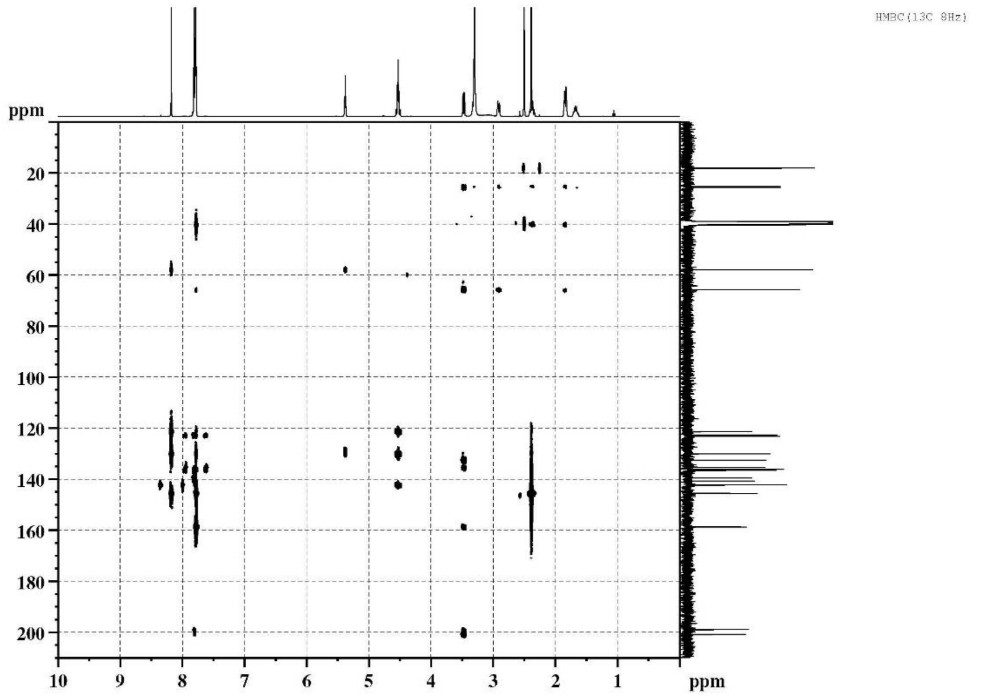

Figure S46. ^{1}H- ^{13}C HMBC correlations

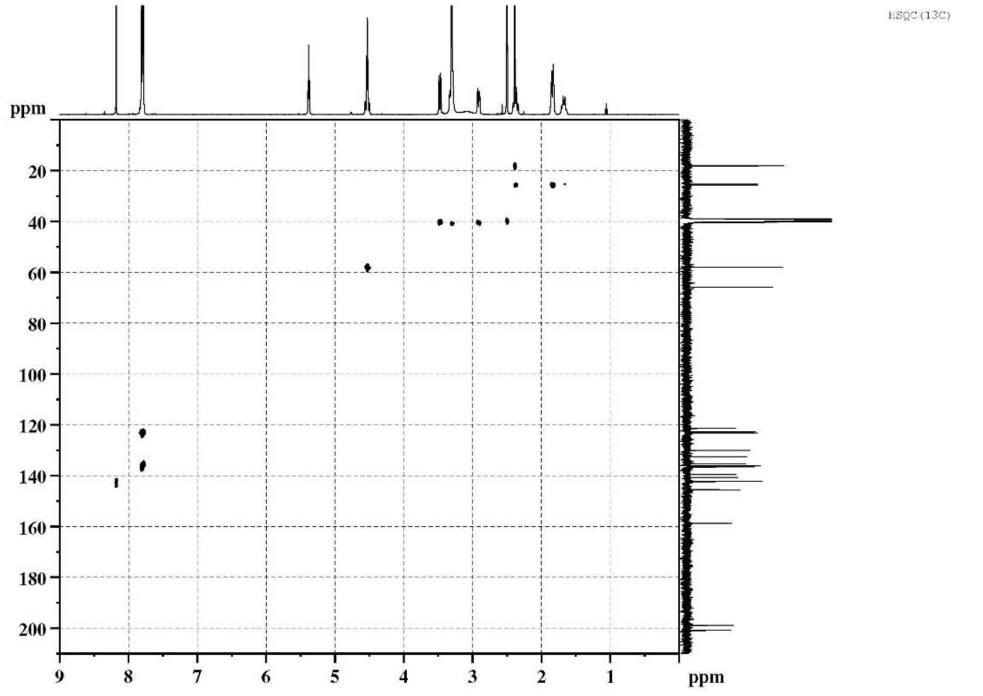

Figure S47. ^{1}H- ^{13}C HSQC correlations

5-(hydroxymethyl)-8-methyl-3-(1-oxo-1*H*-spiro[aceanthrylene-2,2'-piperidin]-3'-yl)-2*H*-pyrano[2,3-*c*]pyridin-2-one 8

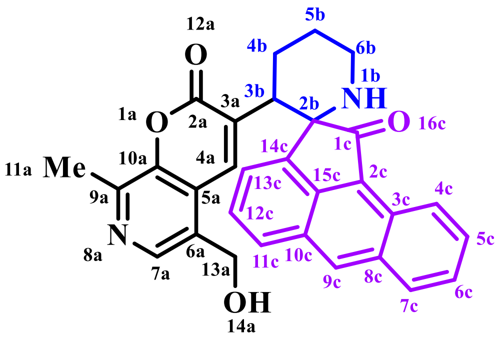

^{1}H NMR (500 MHz, DMSO-d_6): δ 1.865 and 1.992 (m, 2H, H5b), 1.996 and 2.432 (m, 2H, H4b), 2.141 (s, 3H, H11a), 3.123 and 3.440 (m, 2H, H6b), 3.740 (dd, *J* = 13.0 Hz, *J* = 3.0 Hz, 1H, H3b), 4.060 (d, *J* = 5.2 Hz, 2H, H13a), 5.150 (tr, *J* = 5.2 Hz, 1H, H14a), 7.122 (s, 1H, H4a), 7.636 (trd, *J* = 8.6 Hz, *J* = 1.0 Hz, 1H, H6c), 7.743 (dd, *J* = 8.7 Hz, *J* = 6.8 Hz, 1H, H11c), 7.794 (trd, *J* = 8.6 Hz, *J* = 1.0 Hz, 1H, H5c), 7.965 (s, 1H, H7a), 8.047 (d, *J* = 8.7 Hz, 1H, H10c), 8.188 (dd, *J* = 8.6 Hz, *J* = 1.0 Hz, 1H, H7c), 8.230 (d, *J* = 6.8 Hz, 1H, H12c), 8.794 (s, 1H, H9c), 8.896 (dd, *J* = 8.6 Hz, *J* = 1.0 Hz, 1H, H4c).

^{13}C{^{1}H} NMR (DMSO-d_6, 126 MHz) δ 202.3 (C1c), 158.7 (C2a), 145.1 (C9a), 145.1 (C10a), 142.7 (C15c), 141.7 (C7a), 139.1 (C14c), 133.1 (C9c), 133.0 (C4a), 132.9 (C3a), 132.6 (C8c), 129.6 (C5c), 129.8 (C7c), 129.5 (C5a), 127.7 (C10c), 127.6 (C12c), 127.1 (C3c), 126.5 (C6c), 125.1 (C11c), 123.9 (C2c), 123.6 (C4c), 123.0 (C13c), 120.8 (C6a), 69.2 (C2b), 57.4 (C13a), 41.8 (C6b), 41.5 (C3b), 26.8 (C4b), 26.2 (C5b), 17.9 (C11a).

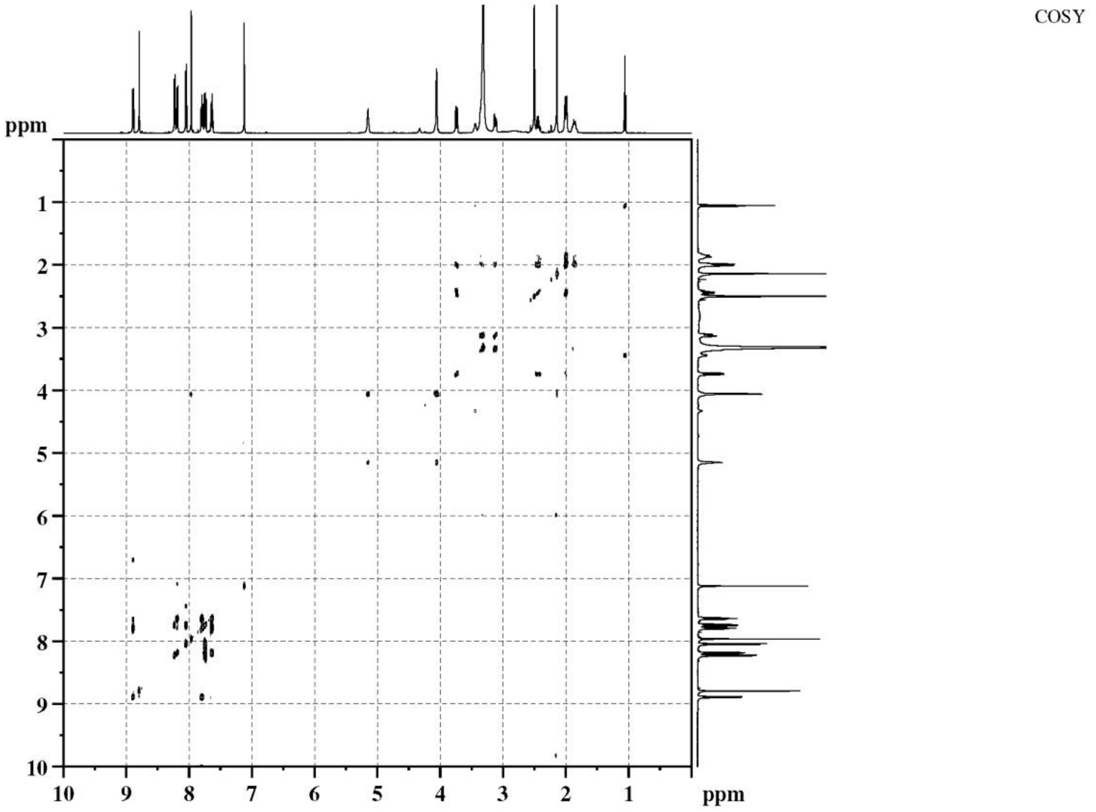

Figure S48. ^{1}H- ^{1}H COSY correlations

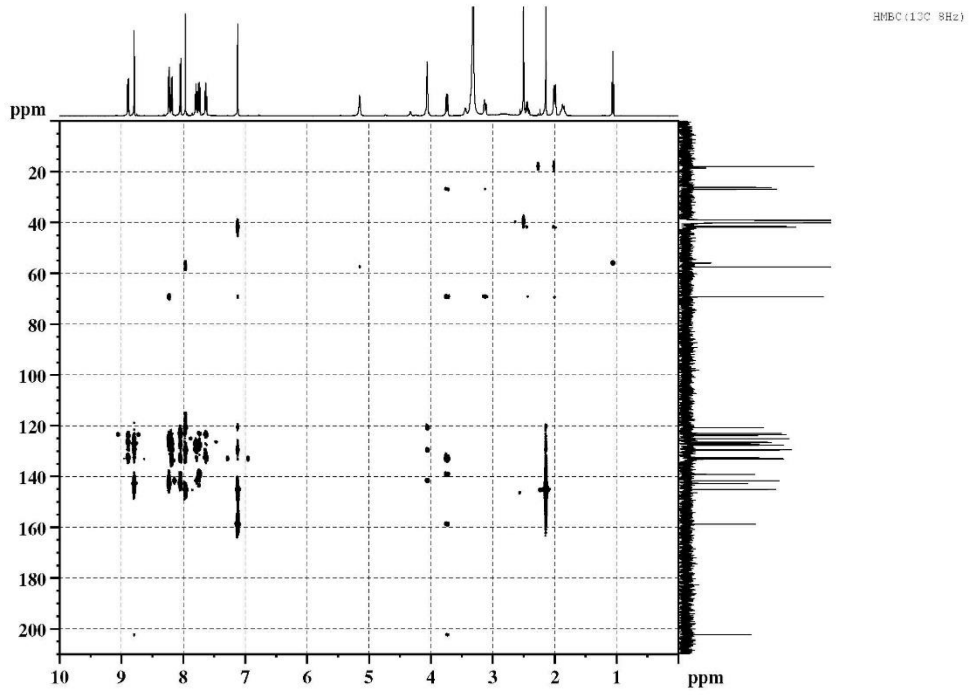

Figure S49. ^{1}H- ^{13}C HMBC correlations

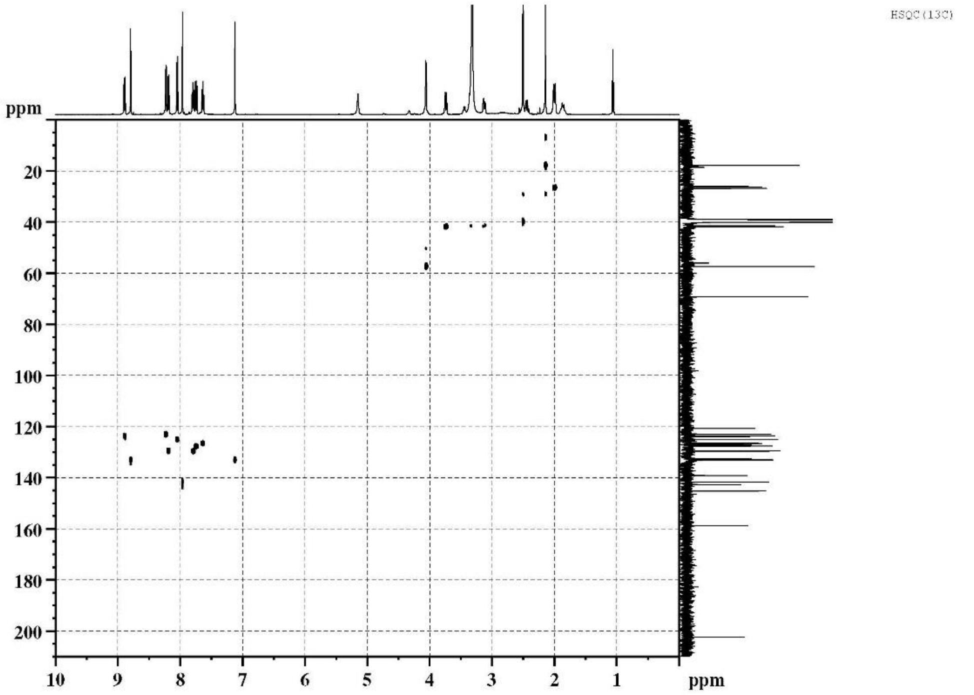

Figure S50. ^{1}H- ^{13}C HSQC correlations

9'-(hydroxymethyl)-2',6'-dimethyl-1',2',3a',9b'-tetrahydro-4'*H*-spiro[indoline-3,3'-pyrrolo[3',4':4,5]pyrano[2,3-*c*]pyridine]-2,4'-dione 13a

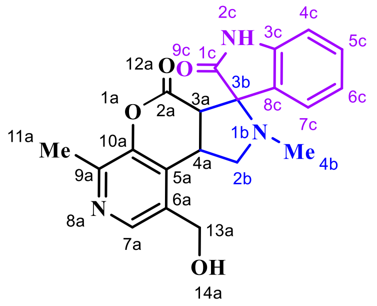

^{1}H NMR (500 MHz, DMSO-d_6): δ 1.991 (s, 3H, H4b), 2.389 (s, 3H, H11a), 3.166 (dd, J = 9.0 Hz, J = 8.4 Hz, 1H, H2b trans- with respect to H4a) and 3.694 (dd, J = 8.4 Hz, J = 8.2 Hz, 1H, H2b cis- with respect to H4a), 4.088 (d, J = 12.2 Hz, 1H, H3a), 4.149 (m, 1H, H4a), 4.518 and 4.625 (dd, J = 13.4 Hz, J = 5.4 Hz, 2H, H13a), 5.357 (tr, J = 5.4 Hz, 2H, H14a), 6.652 (d, J = 7.6 Hz, 1H, H4c), 7.059 (tr, J = 7.6 Hz, 1H, H6c), 7.272 (trd, J = 7.6 Hz, J = 1.4 Hz, 1H, H5c), 7.354 (dd, J = 7.6 Hz, J = 1.4 Hz, 1H, H7c), 8.232 (s, 1H, H7a), 10.409 (s, 1H, H2c or (NH)).

^{13}C{^{1}H} NMR (DMSO-d_6, 126 MHz) δ 177.2 (C1c), 165.4 (C2a), 144.9 (C9a), 144.3 (C10a), 143.4 (C7a), 143.2 (C3c), 133.1 (C6a), 129.4 (C5c), 128.9 (C8c), 127.4 (C5a), 124.1 (C7c), 122.1 (C6c), 109.6 (C4c), 74.7 (C3b), 58.7 (C2b), 58.6 (C13a), 48.8 (C3a), 33.9 (C4b), 33.8 (C4a), 18.9 (C11a).

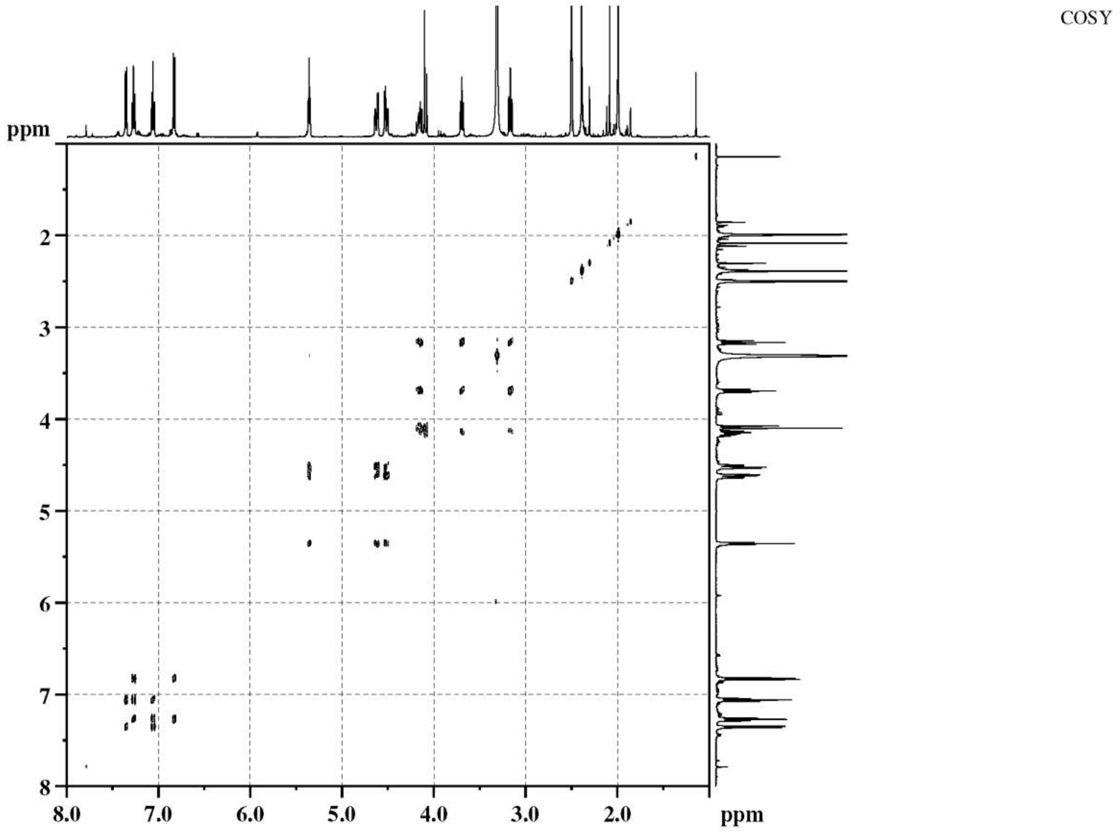

Figure S51. ^{1}H- ^{1}H COSY correlations

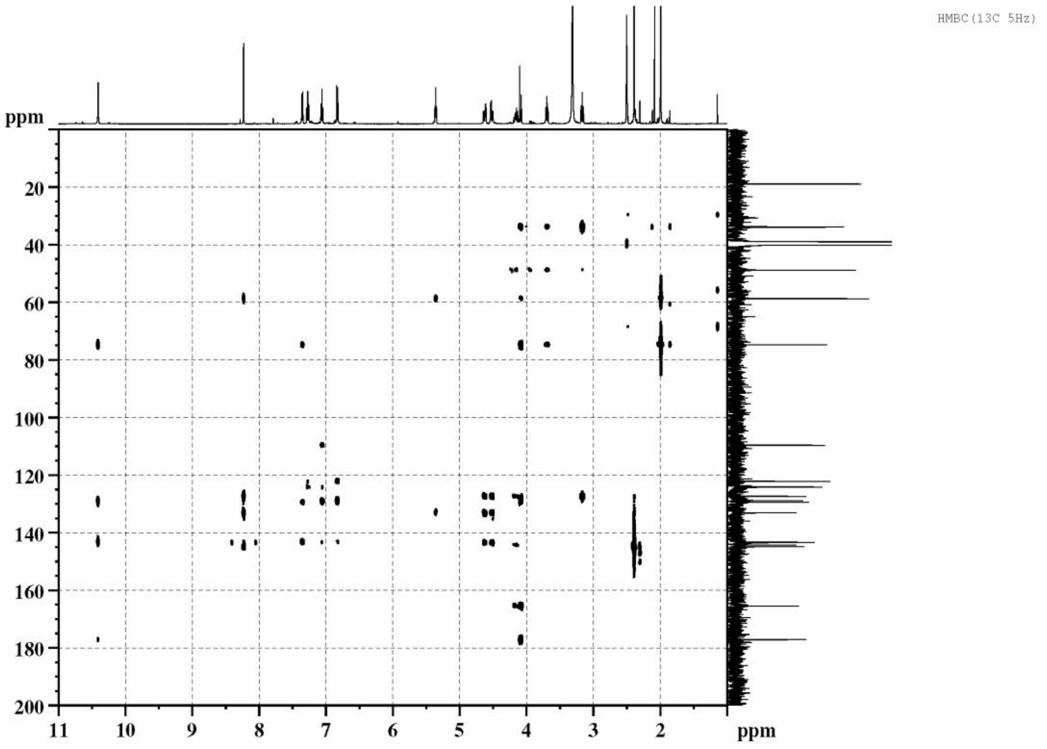

Figure S52. ^{1}H- ^{13}C HMBC correlations

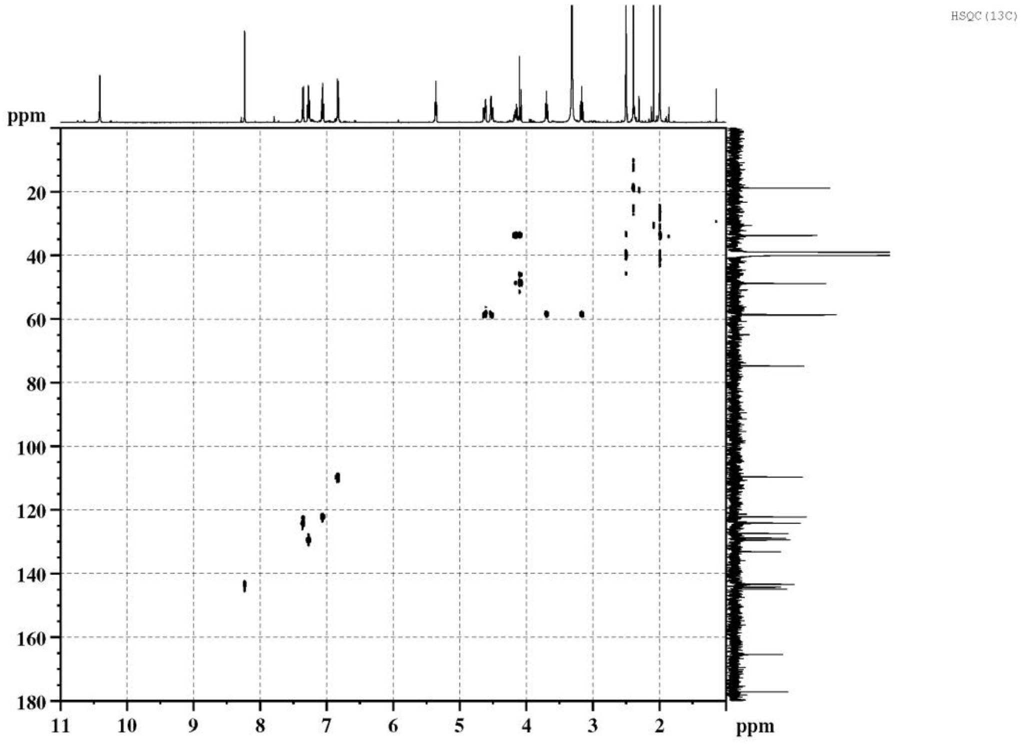

Figure S53. ^{1}H- ^{13}C HSQC correlations

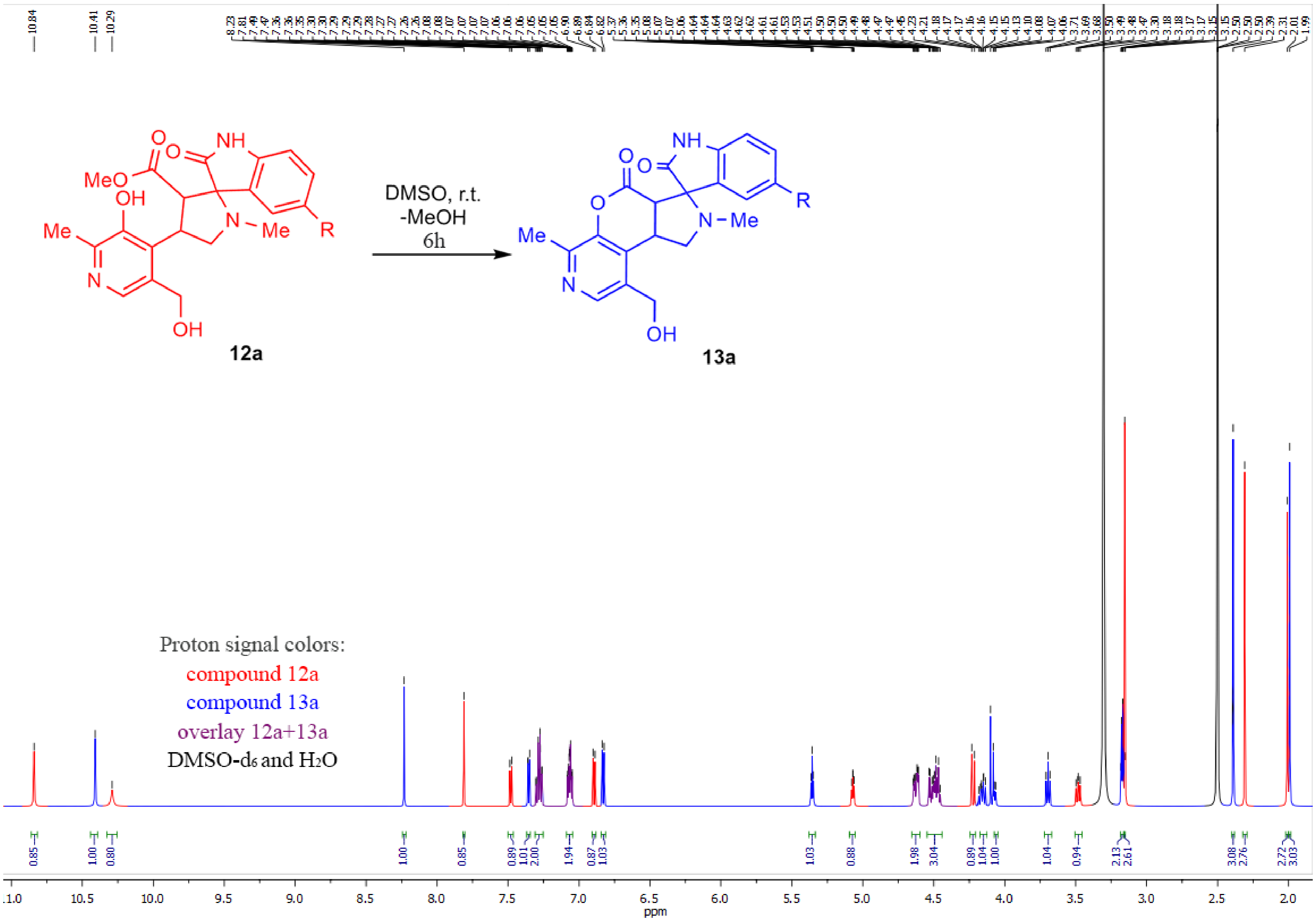

Figure S54. ^{1}H NMR spectra of the compound **12a** kept in a DMSO-d_6 solution for 6 hours at room temperature.

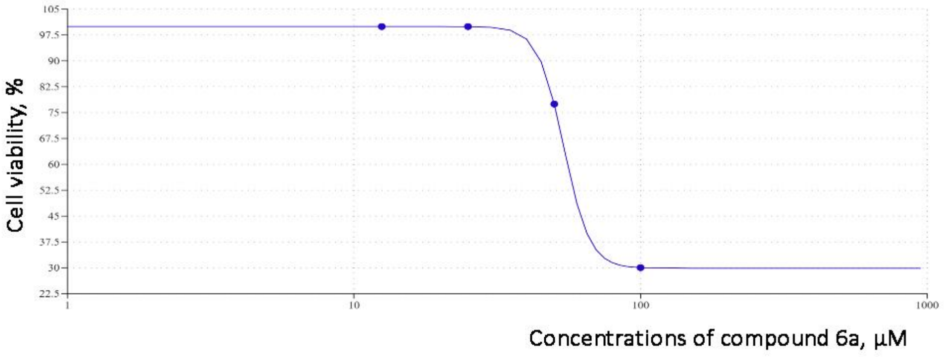

Figure S55. IC_{50} Chart of the compound **6a** for Wi-38 cell line

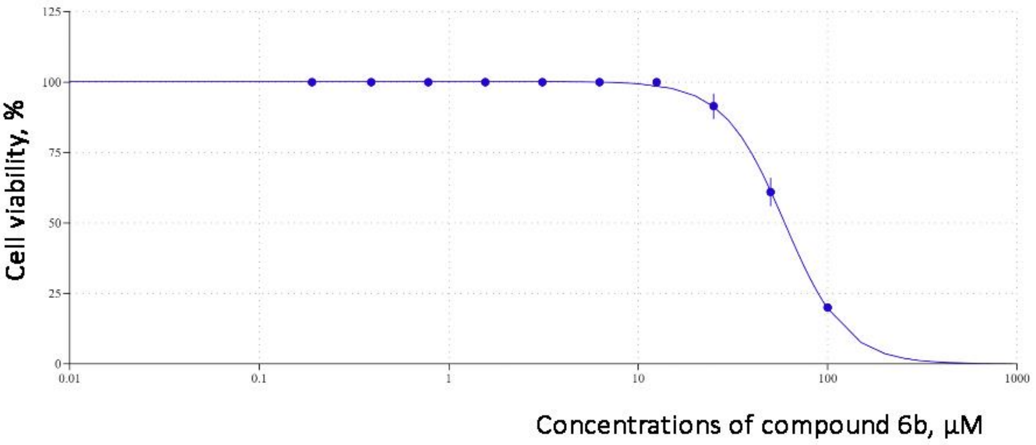

Figure S56. IC_{50} Chart of the compound **6b** for HuTu80 cell line

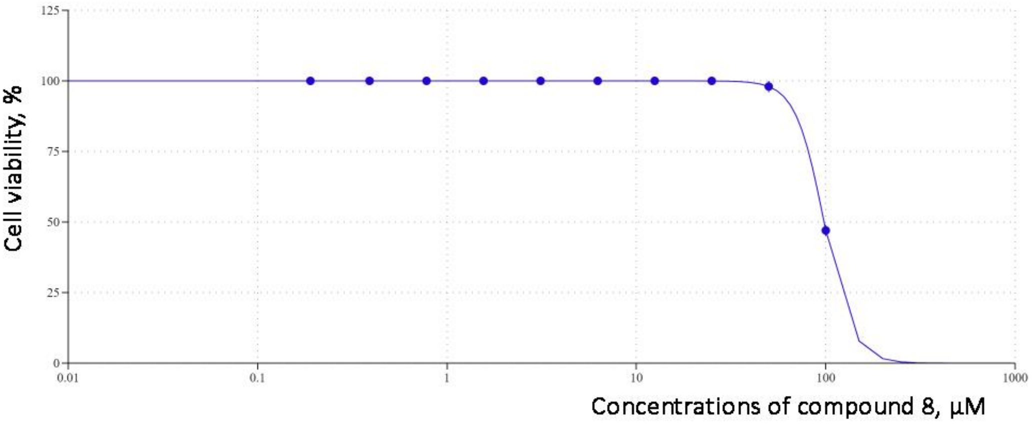

Figure S57. IC_{50} Chart of the compound **8** for Wi-38 cell line

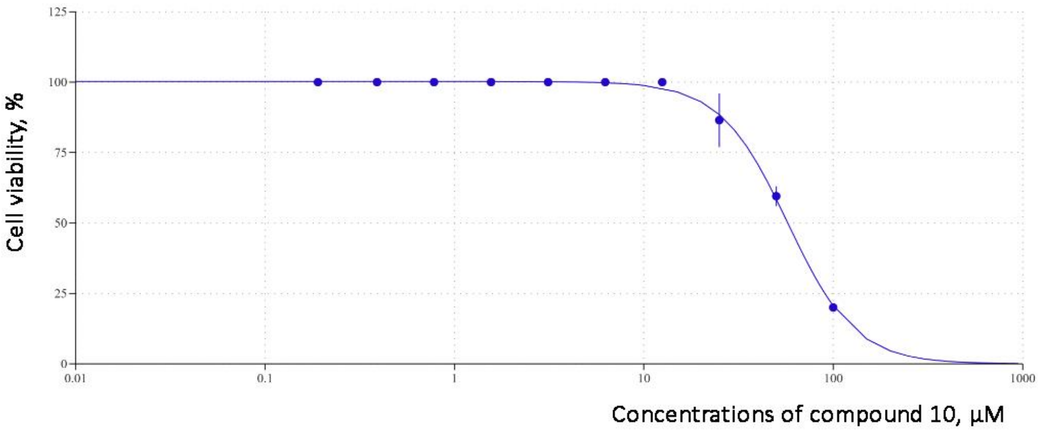

Figure S58. IC_{50} Chart of the compound **10** for M-HeLa cell line

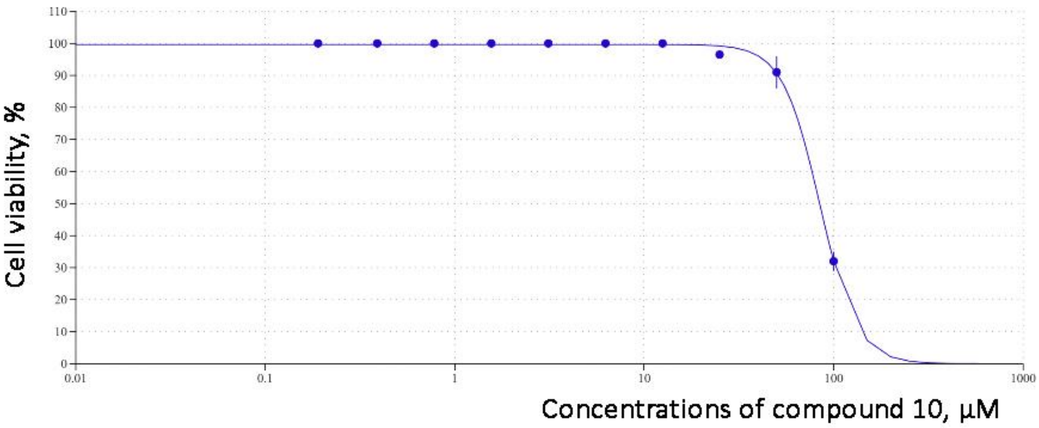

Figure S59. IC_{50} Chart of the compound **10** for A549 cell line

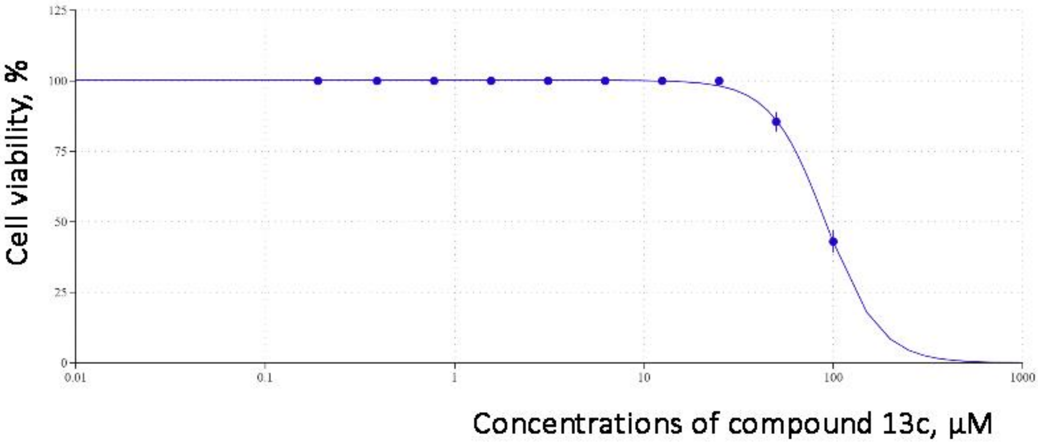

Figure S60. IC_{50} Chart of the compound **13c** for A549 cell line

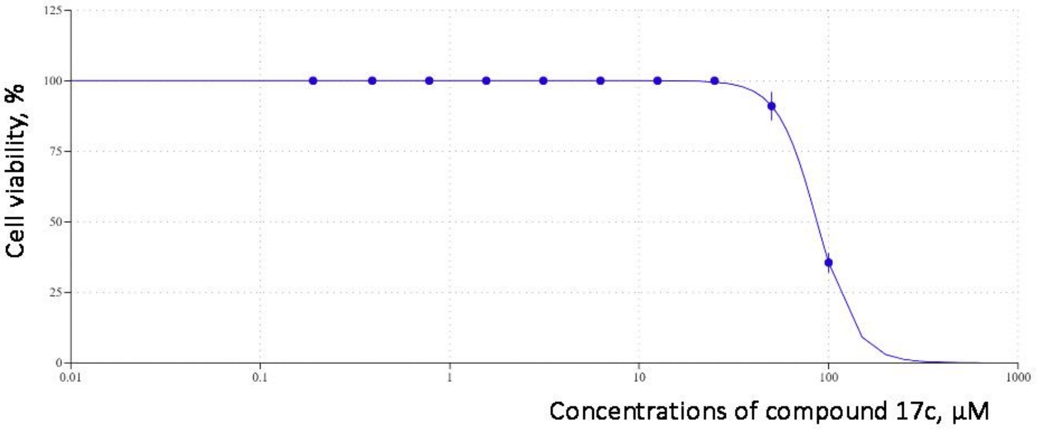

Figure S61. IC_{50} Chart of the compound **17c** for M-HeLa cell line

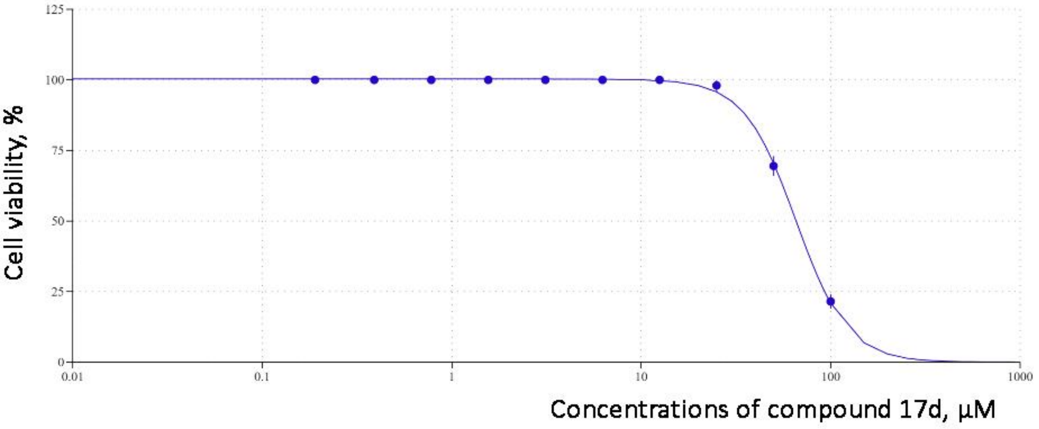

Figure S62. IC_{50} Chart of the compound **17d** for M-HeLa cell line

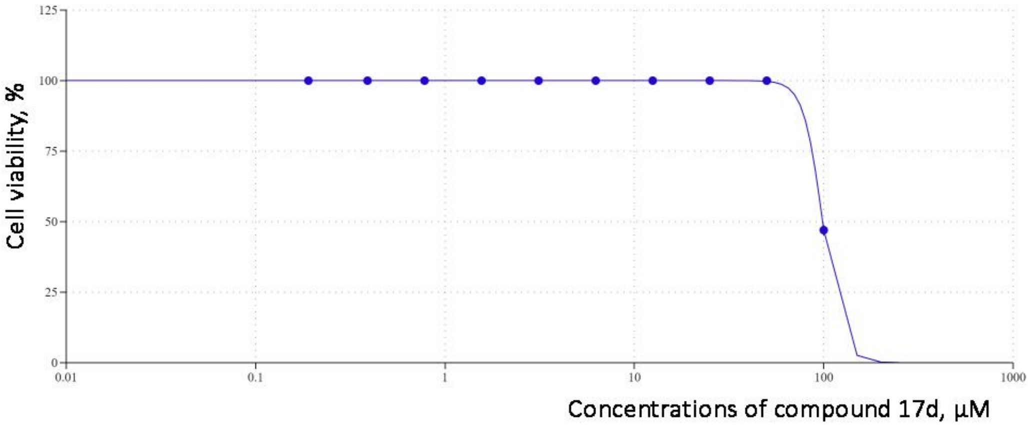

Figure S63. IC_{50} Chart of the compound **17d** for HuTu80 cell line

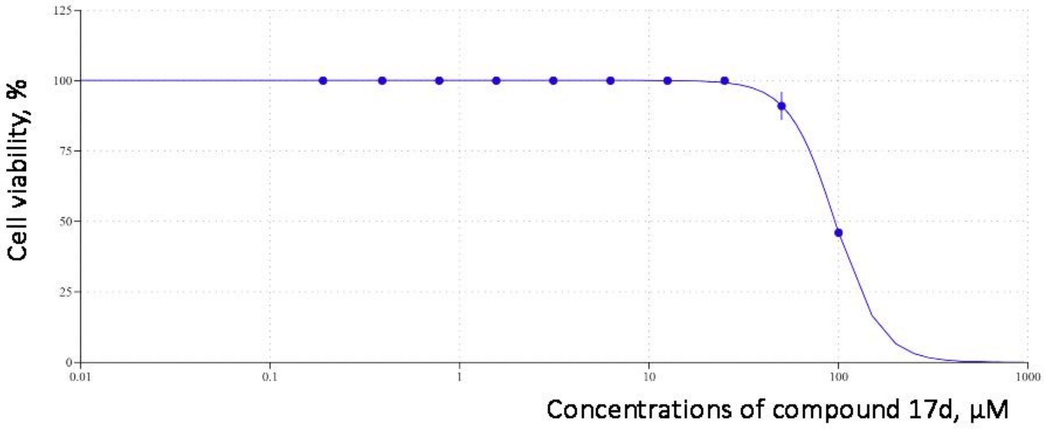

Figure S64. IC_{50} Chart of the compound **17d** for Wi-38 cell line

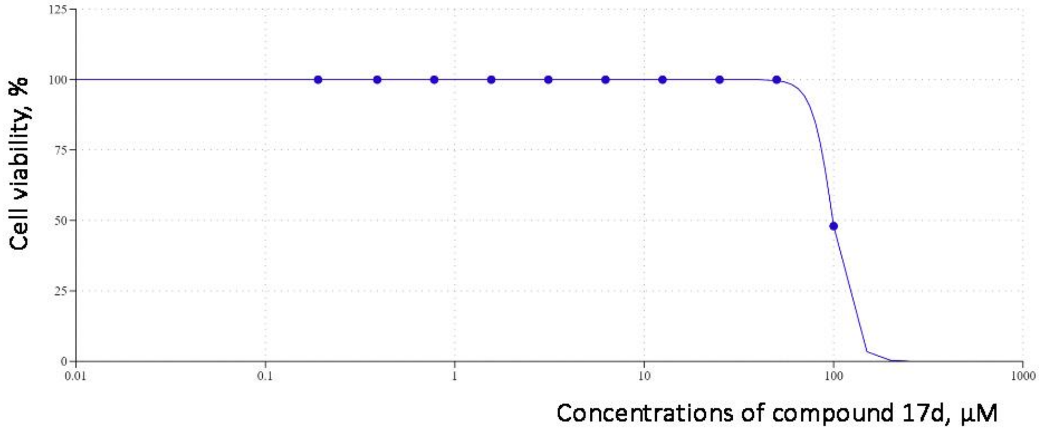

Figure S65. IC_{50} Chart of the compound **17d** for A 549 cell line

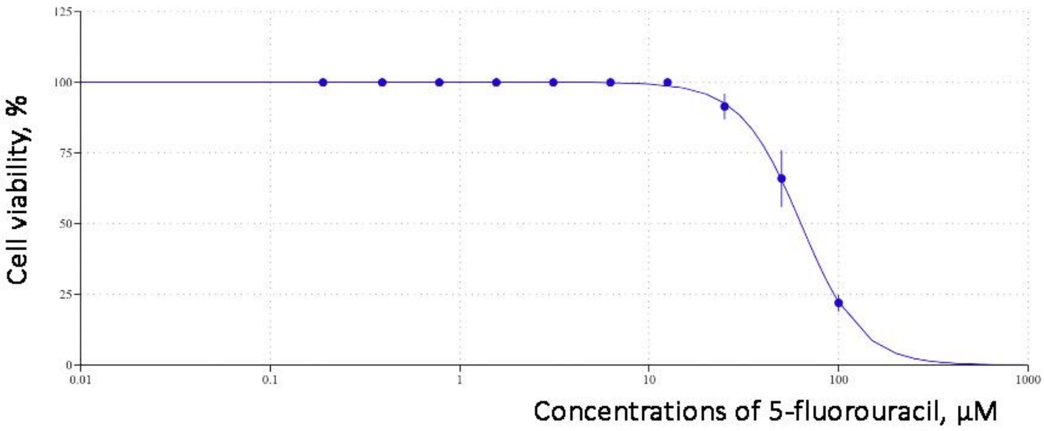

Figure S66. IC_{50} Chart of the compound **5-fluorouracil** for M-HeLa cell line

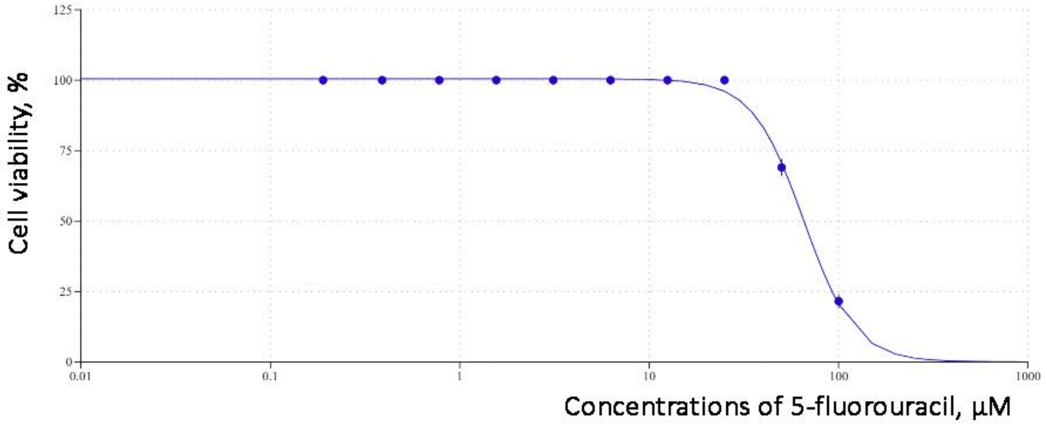

Figure S67. IC_{50} Chart of the compound **5-fluorouracil** for HuTu80 cell line

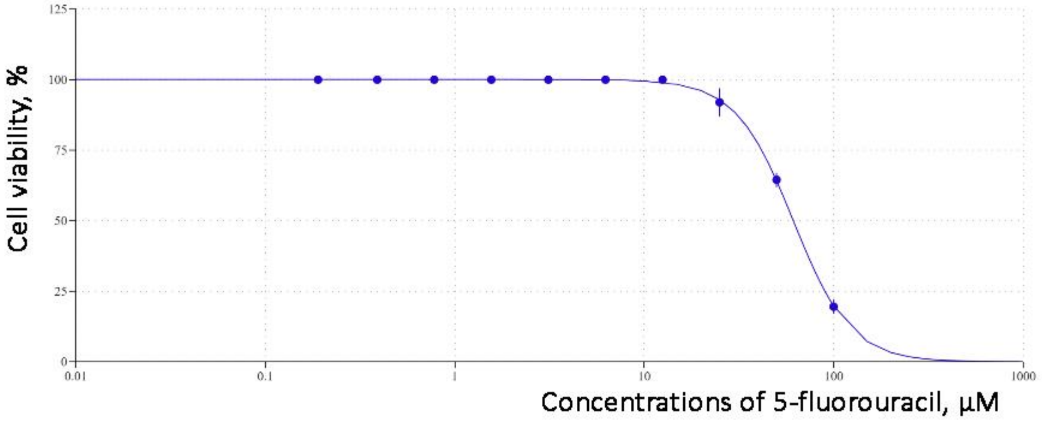

Figure S68. IC_{50} Chart of the compound **5-fluorouracil** for Wi-38 cell line

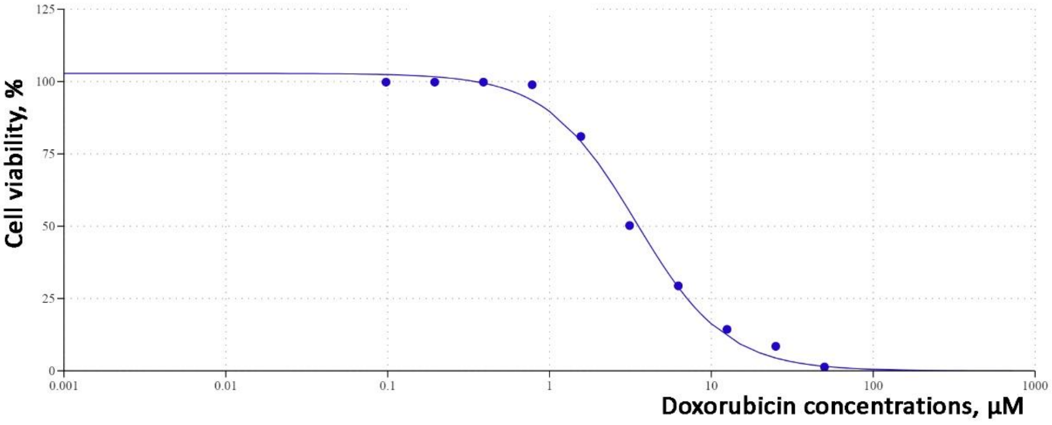

Figure S69. IC_{50} Chart of the compound **Doxorubicin** for M-HeLa cell line

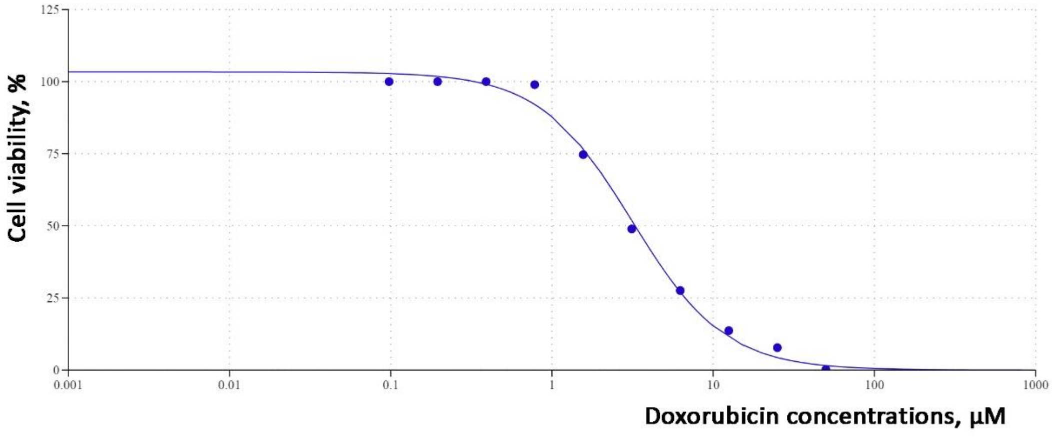

Figure S70. IC_{50} Chart of the compound **Doxorubicin** for HuTu80 cell line

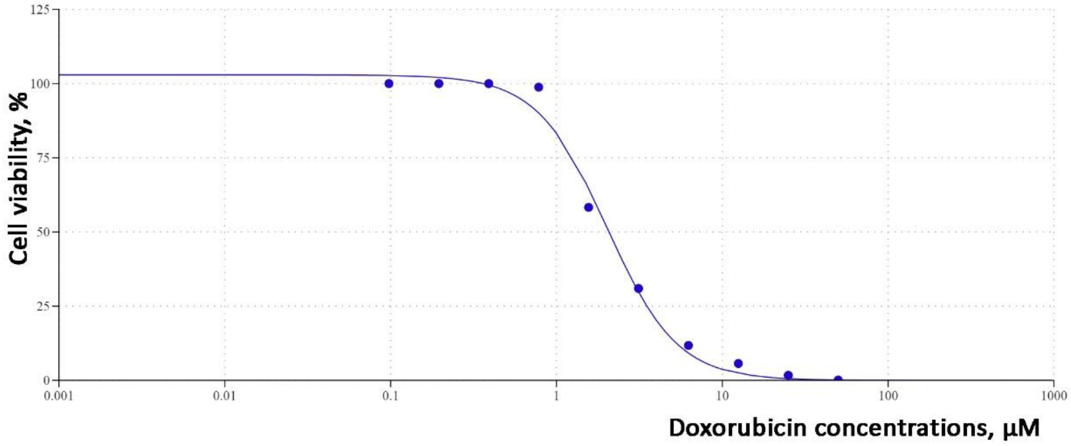

Figure S71. IC_{50} Chart of the compound **Doxorubicin** for A 549 cell line

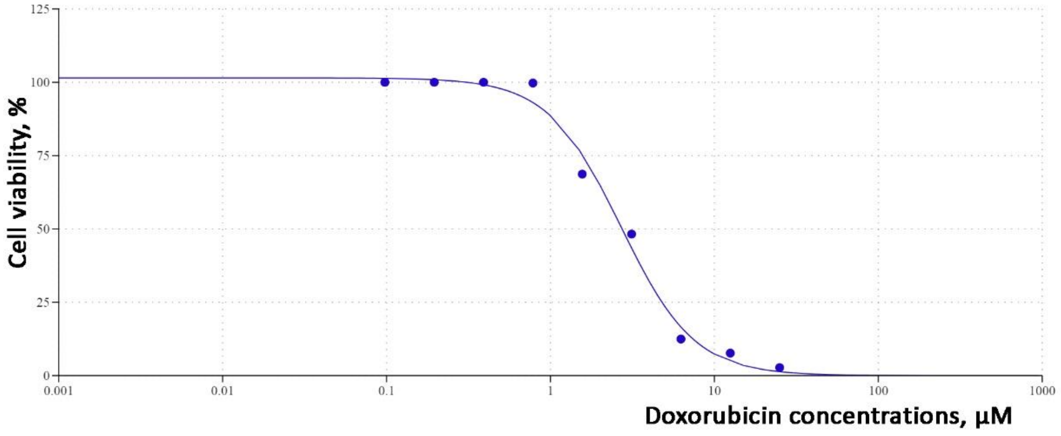

Figure S72. IC_{50} Chart of the compound **Doxorubicin** for Wi-38 cell line

Table S4. Data of quantitative 1D ¹H NMR spectroscopy (qNMR) experiments.

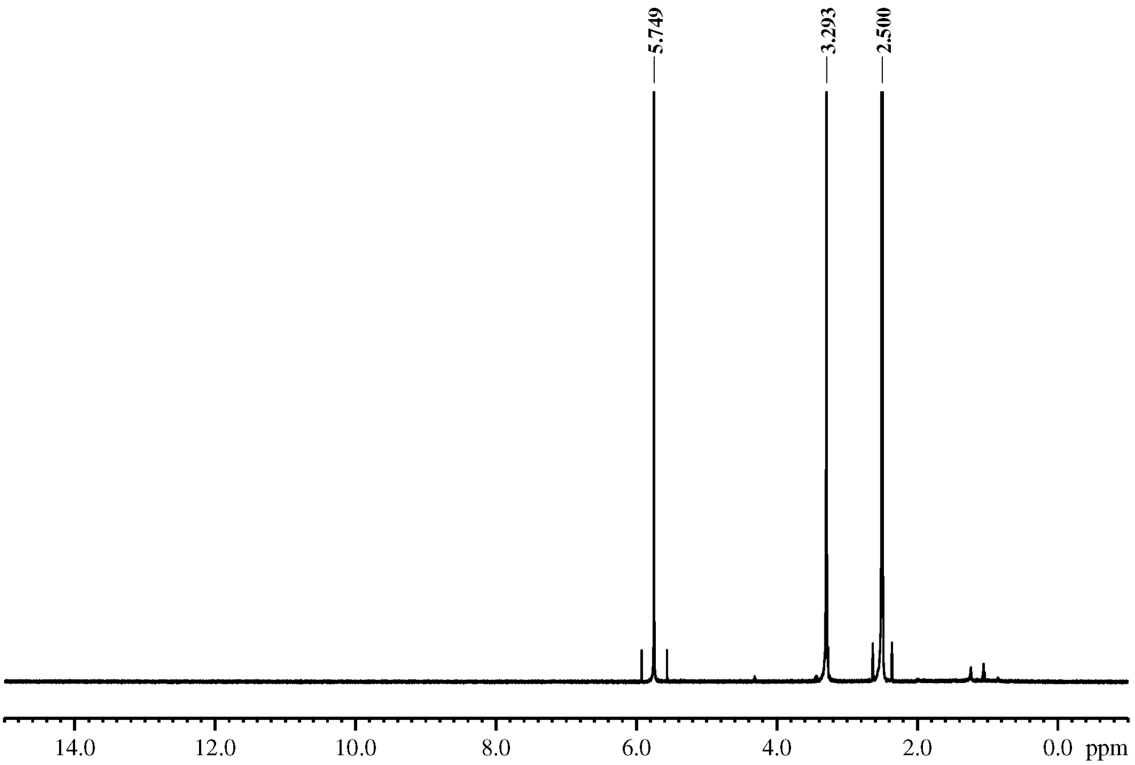

^{1}H NMR spectrum of the solvent (DMSO-d_6) with the addition of dichloromethane (extra pure) as an internal calibrant

Quantitative 1D ^{1}H NMR spectroscopy (qNMR) experiments were performed according to the procedure described in Pauli, G. F.; Chen, S.-N.; Simmler, C.; Lankin, D. C.; Gödecke, T.; Jaki, B. U.; J. Friesen, B.; McAlpine, J. B.; Napolitano, J. G. Importance of Purity Evaluation and the Potential of Quantitative 1H NMR as a Purity Assay. *J. Med. Chem.* **2014**, *57*, 9220-9231. DOI: 10.1021/jm500734a

$$P\,[\%] = \frac{n_{IC} \cdot Int_t \cdot MW_t \cdot m_{IC}}{n_t \cdot Int_{IC} \cdot MW_{IC} \cdot m_s} \cdot P_{IC}$$

Where: m_{IC} = weight (mass) of the internal calibrant (IC)
m_S = weight (mass) of the sample
Int_{IC} = area (integral) of the IC resonance signal being used for quantification
Int_t = area (integral) of the target analyte (t) resonance signal being used for quantification
n_{IC} = number of protons that give rise to Int_{IC}
n_t = number of protons of the target analyte that give rise to Int_t
MW_{IC} = molecular weight of the internal calibrant
MW_t = molecular weight of the target analyte
P_{IC} = purity of the internal calibrant, as percent value

Controlled parameters	
Pulse angle	90^0
RT Relaxation delay (D1)	60s
Acquisition time (AQ)	4s
Spectral window (SW)	30 ppm
Transmitter offset	7.5 ppm
Number of scans	64
Temperature	303 K

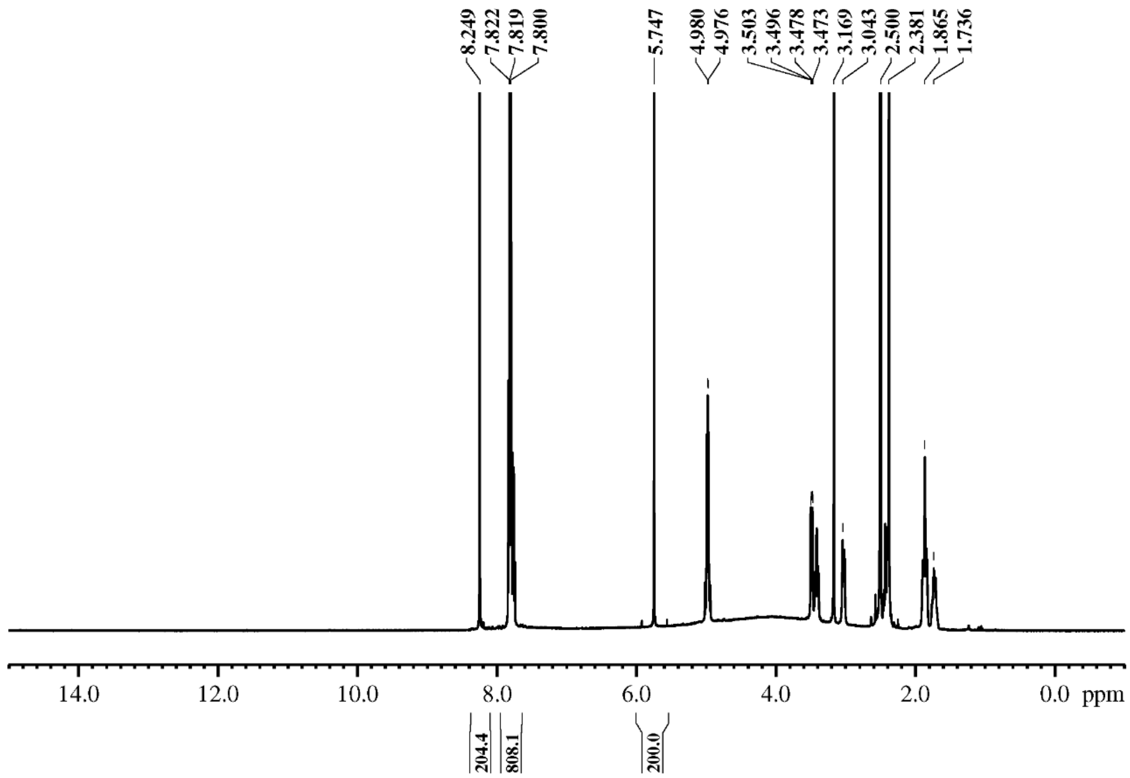

^{1}H NMR spectrum of compound **6b** in DMSO-d_6 solution with the addition of dichloromethane (extra pure) as an internal calibrant

m_s = 4.8 mg, m_{IC} = 0.4 mg, P_{IC} = 99.8%
Int_t = 204.4, n_t = 1
Int_{IC} = 200, n_{IC} = 2
MW_t = 484.40 g/mol, MW_{IC} = 84.93 g/mol

$$P = \frac{2 * 204.4 * 484.40 * 0.4}{1 * 200 * 84.93 * 4.8} * 99.8 = 97.0\%$$

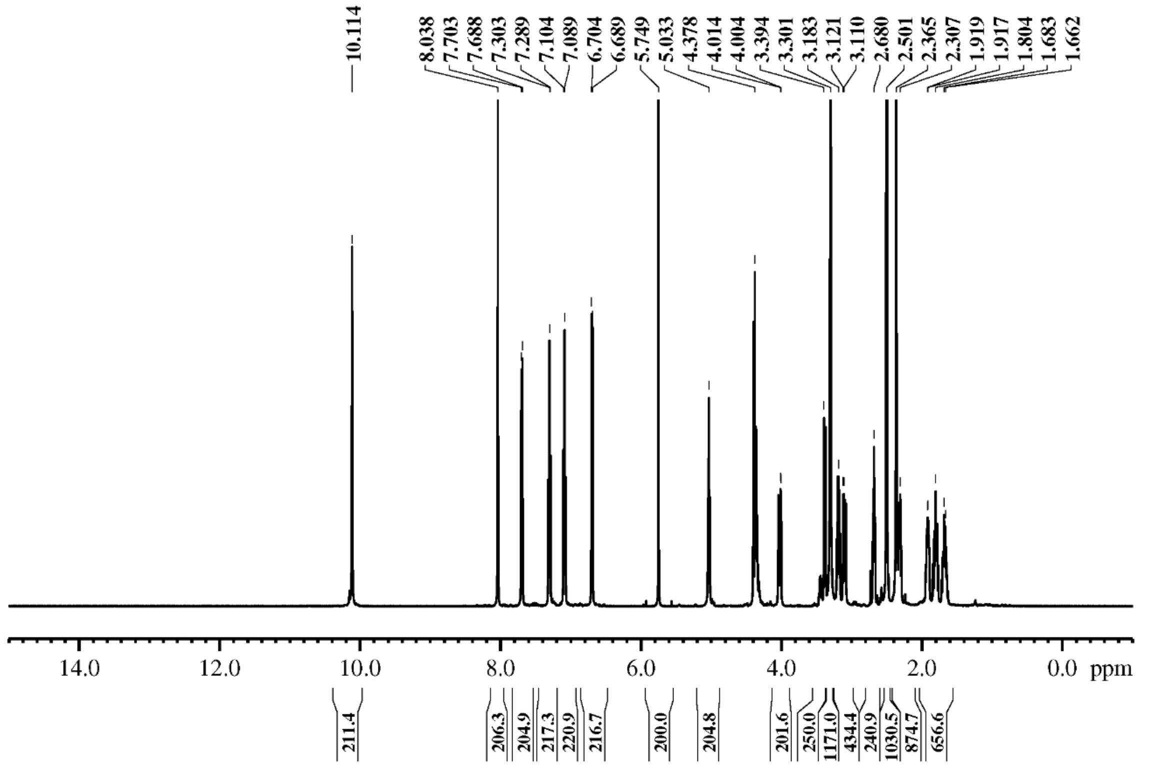

^{1}H NMR spectrum of compound **10** in DMSO-d_6 solution with the addition of dichloromethane (extra pure) as an internal calibrant

m_s = 3.9 mg, m_{IC} = 0.4 mg, P_{IC} = 99.8%

Int_t = 206.3, n_t = 1

Int_{IC} = 200, n_{IC} = 2

MW_t = 391.43 g/mol, MW_{IC} = 84.93 g/mol

$$P = \frac{2 * 206.3 * 391.43 * 0.4}{1 * 200 * 84.93 * 3.9} * 99.8 = 97.3\%$$

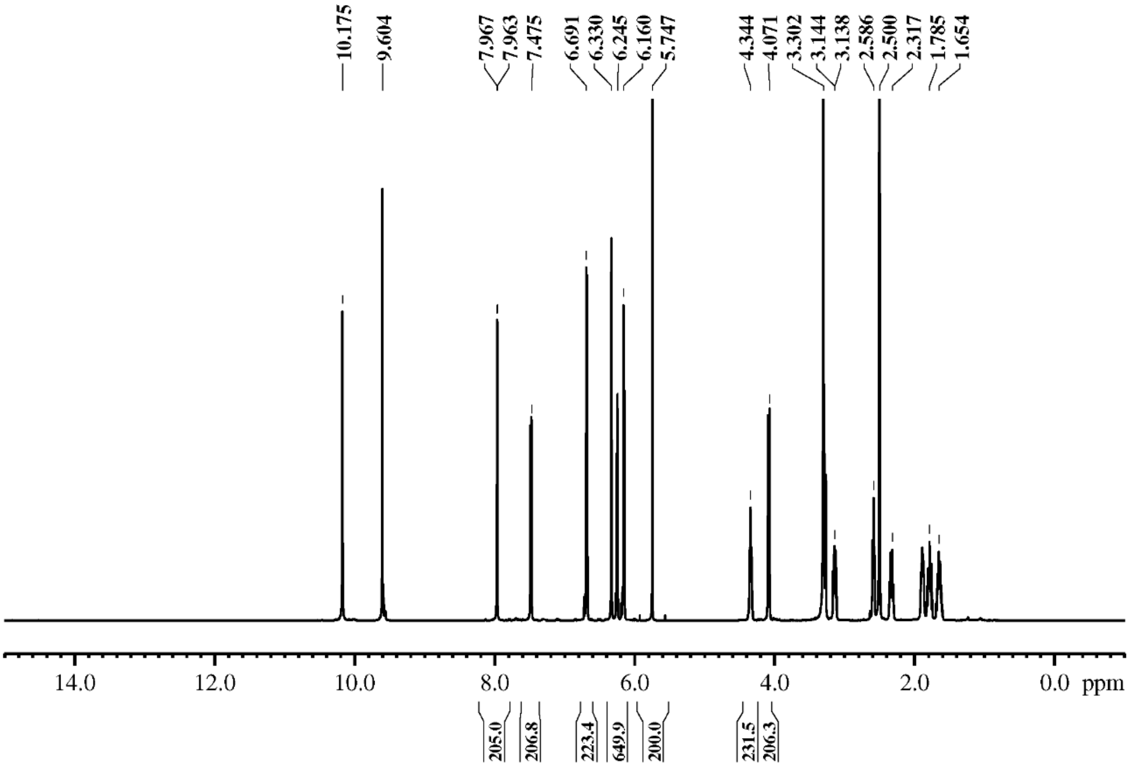

^{1}H NMR spectrum of compound **17d** in DMSO-d_6 solution with the addition of dichloromethane (extra pure) as an internal calibrant

m_s = 4.4 mg, m_{IC} = 0.4 mg, P_{IC} = 99.8%
Int_t = 206.8, n_t = 1
Int_{IC} = 200, n_{IC} = 2
MW_t = 441.28 g/mol, MW_{IC} = 84.93 g/mol

$$P = \frac{2 * 206.8 * 441.28 * 0.4}{1 * 200 * 84.93 * 4.4} * 99.8 = 97.5\%$$

checkCIF/PLATON report

Structure factors have been supplied for datablock(s) kibard22_2_0m_a

THIS REPORT IS FOR GUIDANCE ONLY. IF USED AS PART OF A REVIEW PROCEDURE FOR PUBLICATION, IT SHOULD NOT REPLACE THE EXPERTISE OF AN EXPERIENCED CRYSTALLOGRAPHIC REFEREE.

No syntax errors found.   CIF dictionary   Interpreting this report

Datablock: kibard22_2_0m_a

Bond precision:	C-C = 0.0021 A		Wavelength=0.71073
Cell:	a=12.7434(10)	b=13.0155(10)	c=22.6919(18)
	alpha=90	beta=94.697(3)	gamma=90
Temperature:	110 K		

	Calculated	Reported
Volume	3751.1(5)	3751.1(5)
Space group	P 21/n	P 1 21/n 1
Hall group	-P 2yn	-P 2yn
Moiety formula	C23 H20 N2 O5	C23 H20 N2 O5
Sum formula	C23 H20 N2 O5	C23 H20 N2 O5
Mr	404.41	404.41
Dx,g cm-3	1.432	1.432
Z	8	8
Mu (mm-1)	0.102	0.102
F000	1696.0	1696.0
F000'	1696.86	
h,k,lmax	15,15,27	15,15,27
Nref	6836	6806
Tmin,Tmax	0.985,0.988	0.684,0.707
Tmin'	0.985	

Correction method= # Reported T Limits: Tmin=0.684 Tmax=0.707
AbsCorr = MULTI-SCAN

Data completeness= 0.996 Theta(max)= 25.303

R(reflections)= 0.0417(5047) wR2(reflections)= 0.1146(6806)

S = 1.059 Npar= 553

The following ALERTS were generated. Each ALERT has the format
test-name_ALERT_alert-type_alert-level.
Click on the hyperlinks for more details of the test.

Alert level C

```
PLAT230_ALERT_2_C Hirshfeld Test Diff for    O11A     --C2A      .        5.7 s.u.
PLAT420_ALERT_2_C D-H Bond Without Acceptor  N14B     --H14B     .     Please Check
PLAT911_ALERT_3_C Missing FCF Refl Between Thmin & STh/L=    0.600          8 Report
               12  4  1, -15  0  5, -12  0  8,   0  6 15,  11  2 16,  -6  0 18,
                0  0 20,   0  0 22,
```

Alert level G

```
PLAT007_ALERT_5_G Number of Unrefined Donor-H Atoms .............          2 Report
              H28A    H28B
PLAT793_ALERT_4_G Model has Chirality at C12A        (Centro SpGr)          R Verify
PLAT793_ALERT_4_G Model has Chirality at C12B        (Centro SpGr)          S Verify
PLAT909_ALERT_3_G Percentage of I>2sig(I) Data at Theta(Max) Still        54% Note
PLAT910_ALERT_3_G Missing FCF Reflection(s) Below Theta(Min)[Deg]=       1.90 Note
               -1  0  1,   0  1  1,   0  0  2,
PLAT912_ALERT_4_G Missing # of FCF Reflections Above STh/L=  0.600         19 Note
PLAT933_ALERT_2_G Number of HKL-OMIT Records in Embedded .res File         11 Note
                0  0  2,  -1  0  1,   0  6 15,   0  0 22,  -6  0 18,   0  1  1,
                0  0 20, -12  0  8,  12  4  1, -15  0  5,  11  2 16,
PLAT967_ALERT_5_G Note: Two-Theta Cutoff Value in Embedded .res ..       60.0 Degree
PLAT969_ALERT_5_G The 'Henn et al.' R-Factor-gap value ..........      2.121 Note
              Predicted wR2: Based on SigI**2  5.41 or SHELX Weight 10.83 Note
PLAT978_ALERT_2_G Number C-C Bonds with Positive Residual Density.          7 Info
```

---

0 **ALERT level A** = Most likely a serious problem - resolve or explain
0 **ALERT level B** = A potentially serious problem, consider carefully
3 **ALERT level C** = Check. Ensure it is not caused by an omission or oversight
10 **ALERT level G** = General information/check it is not something unexpected

0 ALERT type 1 CIF construction/syntax error, inconsistent or missing data
4 ALERT type 2 Indicator that the structure model may be wrong or deficient
3 ALERT type 3 Indicator that the structure quality may be low
3 ALERT type 4 Improvement, methodology, query or suggestion
3 ALERT type 5 Informative message, check

It is advisable to attempt to resolve as many as possible of the alerts in all categories. Often the minor alerts point to easily fixed oversights, errors and omissions in your CIF or refinement strategy, so attention to these fine details can be worthwhile. It is up to the individual to critically assess their own results and, if necessary, seek expert advice.

PLATON version of 23/04/2026; check.def file version of 30/03/2026

---

duplicate check

No duplication found

Datablock kibard22_2_0m_a - ellipsoid plot

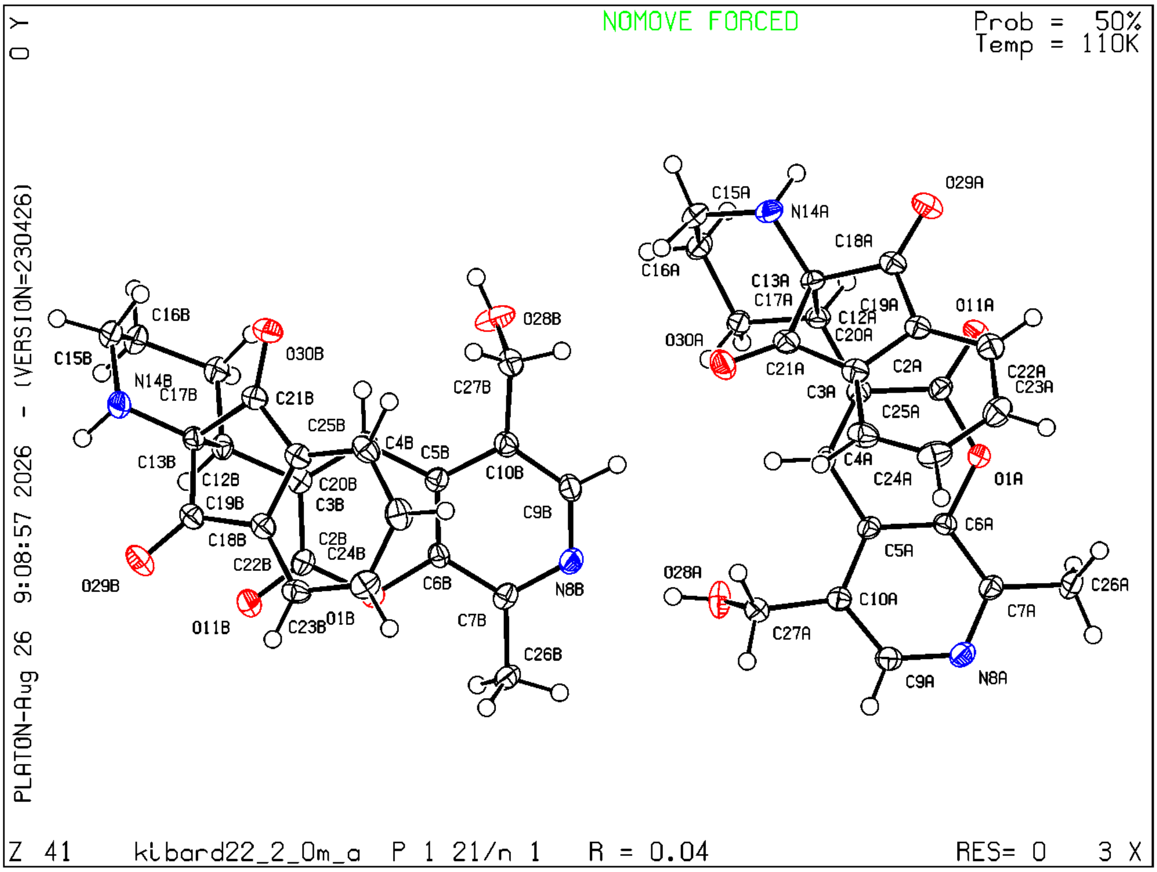

checkCIF/PLATON report

Structure factors have been supplied for datablock(s) trif_a_7_a

THIS REPORT IS FOR GUIDANCE ONLY. IF USED AS PART OF A REVIEW PROCEDURE FOR PUBLICATION, IT SHOULD NOT REPLACE THE EXPERTISE OF AN EXPERIENCED CRYSTALLOGRAPHIC REFEREE.

No syntax errors found. CIF dictionary Interpreting this report

Datablock: trif_a_7_a

Bond precision: C-C = 0.0014 A Wavelength=0.71073

Cell:	a=9.4629(9)	b=15.8129(16)	c=12.6006(12)
	alpha=90	beta=108.043(3)	gamma=90
Temperature:	150 K		

	Calculated	Reported
Volume	1792.8(3)	1792.8(3)
Space group	P 21/n	P 1 21/n 1
Hall group	-P 2yn	-P 2yn
Moiety formula	C22 H21 N3 O4	C22 H21 N3 O4
Sum formula	C22 H21 N3 O4	C22 H21 N3 O4
Mr	391.42	391.42
Dx,g cm-3	1.450	1.450
Z	4	4
Mu (mm-1)	0.101	0.101
F000	824.0	824.0
F000'	824.38	
h,k,lmax	15,25,20	15,25,20
Nref	7667	7662
Tmin,Tmax	0.974,0.984	0.715,0.747
Tmin'	0.960	

Correction method= # Reported T Limits: Tmin=0.715 Tmax=0.747
AbsCorr = MULTI-SCAN

```
Data completeness= 0.999            Theta(max)= 34.616

                                                           wR2(reflections)=
R(reflections)= 0.0533( 6273)                              0.1475( 7662)

S = 1.070                   Npar= 277
```

The following ALERTS were generated. Each ALERT has the format
test-name_ALERT_alert-type_alert-level.
Click on the hyperlinks for more details of the test.

Alert level C

```
DIFMX02_ALERT_1_C  The maximum difference density is > 0.1*ZMAX*0.75
            The relevant atom site should be identified.
PLAT097_ALERT_2_C Large Reported Max.  (Positive) Residual Density       0.79 eA-3
PLAT220_ALERT_2_C NonSolvent   Resd 1  C   Ueq(max)/Ueq(min) Range        3.1 Ratio
PLAT241_ALERT_2_C High  MainResAtom  Ueq as Compared to Neighbours        C20 Check
```

Alert level G

```
PLAT007_ALERT_5_G Number of Unrefined Donor-H Atoms .............          1 Report
              H22
PLAT230_ALERT_2_G Hirshfeld Test Diff for    N16      --C18      .        6.9 s.u.
PLAT301_ALERT_3_G Main Residue  Disorder .............(Resd    1)          3% Note
PLAT410_ALERT_2_G Short Intra H...H Contact  H18B     ..H28      .       2.03 Ang.
                                                     x,y,z  =      1_555 Check
PLAT793_ALERT_4_G Model has Chirality at C7          (Centro SpGr)          R Verify
PLAT793_ALERT_4_G Model has Chirality at C8          (Centro SpGr)          S Verify
PLAT793_ALERT_4_G Model has Chirality at C15         (Centro SpGr)          R Verify
PLAT793_ALERT_4_G Model has Chirality at C17         (Centro SpGr)          R Verify
PLAT910_ALERT_3_G Missing FCF Reflection(s) Below Theta(Min)[Deg]=       2.37 Note
                0  1  1,
PLAT912_ALERT_4_G Missing # of FCF Reflections Above STh/L=  0.600          3 Note
PLAT969_ALERT_5_G The 'Henn et al.' R-Factor-gap value ..........       4.807 Note
              Predicted wR2: Based on SigI**2  3.07 or SHELX Weight 13.78 Note
PLAT978_ALERT_2_G Number C-C Bonds with Positive Residual Density.         16 Info
```

---

0 **ALERT level A** = Most likely a serious problem - resolve or explain
0 **ALERT level B** = A potentially serious problem, consider carefully
4 **ALERT level C** = Check. Ensure it is not caused by an omission or oversight
12 **ALERT level G** = General information/check it is not something unexpected

1 ALERT type 1 CIF construction/syntax error, inconsistent or missing data
6 ALERT type 2 Indicator that the structure model may be wrong or deficient
2 ALERT type 3 Indicator that the structure quality may be low
5 ALERT type 4 Improvement, methodology, query or suggestion
2 ALERT type 5 Informative message, check

It is advisable to attempt to resolve as many as possible of the alerts in all categories. Often the minor alerts point to easily fixed oversights, errors and omissions in your CIF or refinement strategy, so attention to these fine details can be worthwhile. It is up to the individual to critically assess their own results and, if necessary, seek expert advice.

PLATON version of 23/04/2026; check.def file version of 30/03/2026

---

duplicate check

No duplication found

Datablock trif_a_7_a - ellipsoid plot

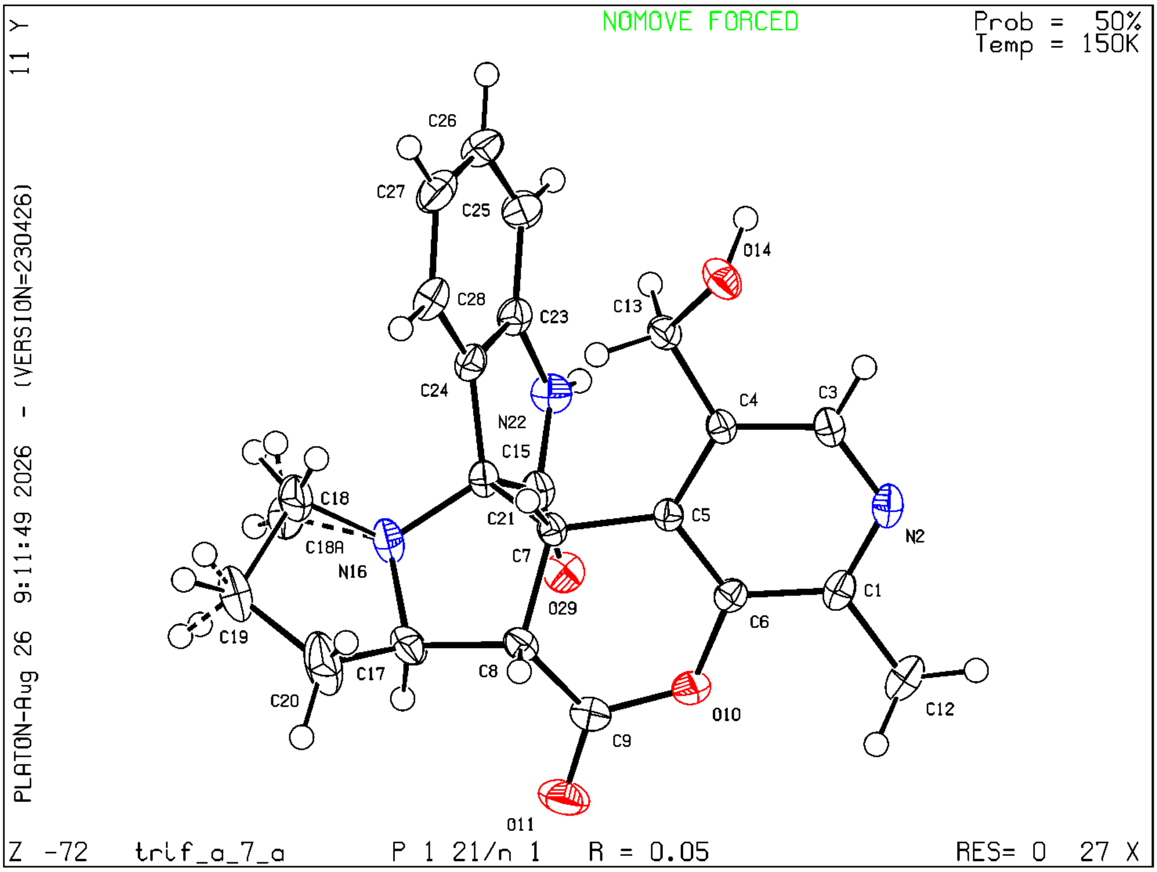

checkCIF/PLATON report

Structure factors have been supplied for datablock(s) trif_a_8_a

THIS REPORT IS FOR GUIDANCE ONLY. IF USED AS PART OF A REVIEW PROCEDURE FOR PUBLICATION, IT SHOULD NOT REPLACE THE EXPERTISE OF AN EXPERIENCED CRYSTALLOGRAPHIC REFEREE.

No syntax errors found. CIF dictionary Interpreting this report

Datablock: trif_a_8_a

Bond precision: C-C = 0.0041 A Wavelength=0.71073

Cell:	a=14.1911(15)	b=13.6496(15)	c=12.3614(13)
	alpha=90	beta=100.268(4)	gamma=90
Temperature:	150 K		

	Calculated	Reported
Volume	2356.1(4)	2356.1(4)
Space group	P 21/c	P 1 21/c 1
Hall group	-P 2ybc	-P 2ybc
Moiety formula	C21 H23 N3 O5 [+ solvent]	C21 H23 N3 O5
Sum formula	C21 H23 N3 O5 [+ solvent]	C21 H23 N3 O5
Mr	397.42	397.42
Dx,g cm-3	1.120	1.120
Z	4	4
Mu (mm-1)	0.081	0.081
F000	840.0	840.0
F000'	840.41	
h,k,lmax	20,19,17	20,19,17
Nref	7217	7205
Tmin,Tmax	0.992,0.996	0.660,0.746
Tmin'	0.990	

Correction method= # Reported T Limits: Tmin=0.660 Tmax=0.746
AbsCorr = MULTI-SCAN

Data completeness= 0.998   Theta(max)= 30.546

R(reflections)= 0.0916(4269)   wR2(reflections)= 0.2545(7205)

S = 1.029   Npar= 277

The following ALERTS were generated. Each ALERT has the format
test-name_ALERT_alert-type_alert-level.
Click on the hyperlinks for more details of the test.

Alert level C

```
RINTA01_ALERT_3_C  The value of Rint is greater than 0.12
            Rint given   0.142
PLAT020_ALERT_3_C The Reported Value of Rint is Greater Than  0.12      0.142 Report
PLAT222_ALERT_3_C NonSolvent Resd 1  H   Uiso(max)/Uiso(min) Range        5.2 Ratio
PLAT340_ALERT_3_C Low Bond Precision on  C-C Bonds ..............    0.00405 Ang.
PLAT352_ALERT_3_C Short  N-H (X0.87,N1.01A)  N7       - H7       .       0.68 Ang.
PLAT355_ALERT_3_C Long   O-H (X0.82,N0.98A)  O29      - H29      .       1.04 Ang.
PLAT906_ALERT_3_C Large K Value in the Analysis of Variance ......      3.069 Check
PLAT906_ALERT_3_C Large K Value in the Analysis of Variance ......      2.616 Check
PLAT911_ALERT_3_C Missing FCF Refl Between Thmin & STh/L=    0.600          3 Report
                1  1  0,   2  0  0,   0  1  1,
```

Alert level G

```
PLAT072_ALERT_2_G SHELXL First  Parameter in WGHT  Unusually Large       0.12 Report
PLAT605_ALERT_4_G Largest Solvent Accessible VOID in the Structure        580 A**3
PLAT793_ALERT_4_G Model has Chirality at C3          (Centro SpGr)          S Verify
PLAT793_ALERT_4_G Model has Chirality at C4          (Centro SpGr)          S Verify
PLAT793_ALERT_4_G Model has Chirality at C5          (Centro SpGr)          S Verify
PLAT868_ALERT_4_G ALERTS Due to the Use of _smtbx_masks Suppressed          ! Info
PLAT912_ALERT_4_G Missing # of FCF Reflections Above STh/L=  0.600          9 Note
PLAT969_ALERT_5_G The 'Henn et al.' R-Factor-gap value ..........      2.818 Note
              Predicted wR2: Based on SigI**2  9.03 or SHELX Weight 24.74 Note
PLAT978_ALERT_2_G Number C-C Bonds with Positive Residual Density.          5 Info
```

---

0 **ALERT level A** = Most likely a serious problem - resolve or explain
0 **ALERT level B** = A potentially serious problem, consider carefully
9 **ALERT level C** = Check. Ensure it is not caused by an omission or oversight
9 **ALERT level G** = General information/check it is not something unexpected

0 ALERT type 1 CIF construction/syntax error, inconsistent or missing data
2 ALERT type 2 Indicator that the structure model may be wrong or deficient
9 ALERT type 3 Indicator that the structure quality may be low
6 ALERT type 4 Improvement, methodology, query or suggestion
1 ALERT type 5 Informative message, check

It is advisable to attempt to resolve as many as possible of the alerts in all categories. Often the minor alerts point to easily fixed oversights, errors and omissions in your CIF or refinement strategy, so attention to these fine details can be worthwhile. It is up to the individual to critically assess their own results and, if necessary, seek expert advice.

PLATON version of 23/04/2026; check.def file version of 30/03/2026

---

duplicate check

No duplication found

Datablock trif_a_8_a - ellipsoid plot

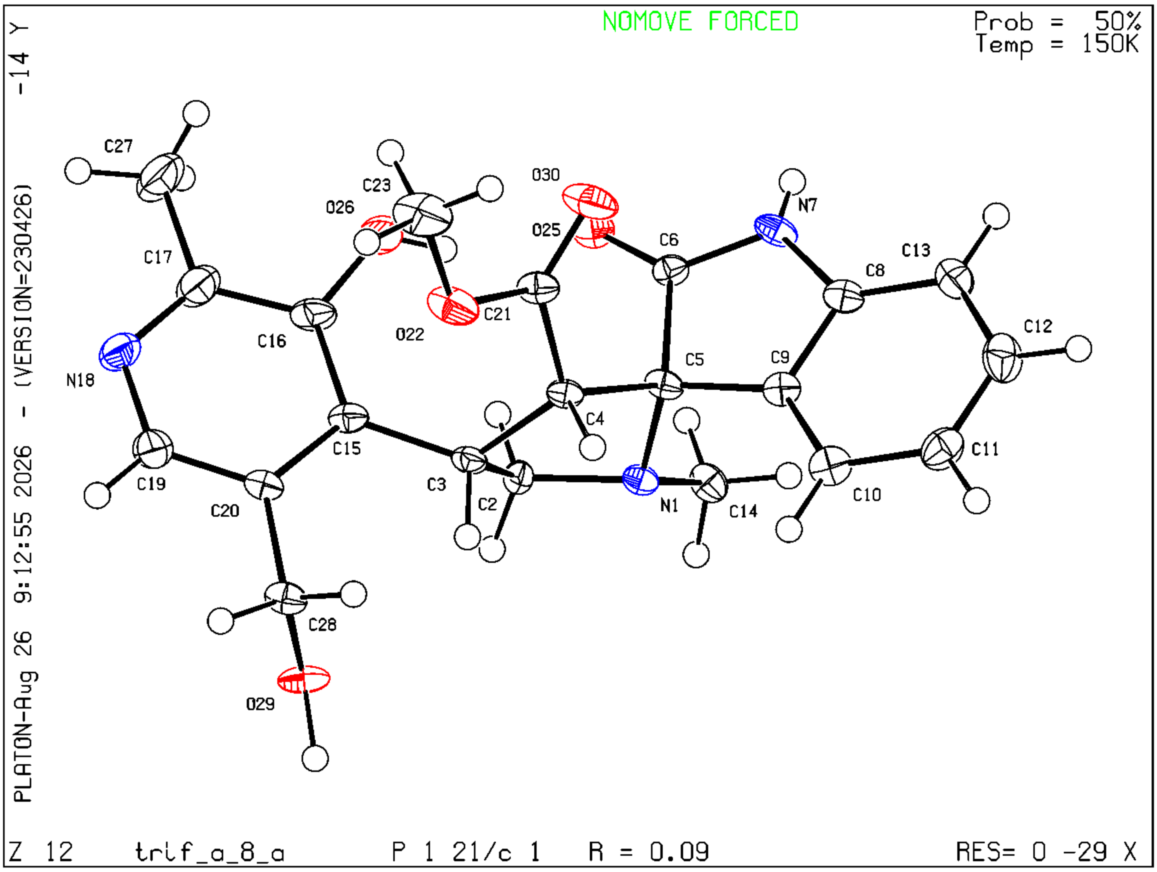

checkCIF/PLATON report

Structure factors have been supplied for datablock(s) trif_a2_0m_a

THIS REPORT IS FOR GUIDANCE ONLY. IF USED AS PART OF A REVIEW PROCEDURE FOR PUBLICATION, IT SHOULD NOT REPLACE THE EXPERTISE OF AN EXPERIENCED CRYSTALLOGRAPHIC REFEREE.

No syntax errors found. CIF dictionary Interpreting this report

Datablock: trif_a2_0m_a

Bond precision:	C-C = 0.0054 A		Wavelength=0.71073
Cell:	a=10.3836(12)	b=29.164(4)	c=10.4383(11)
	alpha=90	beta=114.315(4)	gamma=90
Temperature:	105 K		

	Calculated	Reported
Volume	2880.6(6)	2880.6(6)
Space group	P 21/n	P 1 21/n 1
Hall group	-P 2yn	-P 2yn
Moiety formula	C21 H18 N2 O4, 2(C2 H6 O S) [+ solvent]	2(C2 H6 O S), C21 H18 N2 O4
Sum formula	C25 H30 N2 O6 S2 [+ solvent]	C25 H30 N2 O6 S2
Mr	518.63	518.63
Dx,g cm-3	1.196	1.196
Z	4	4
Mu (mm-1)	0.223	0.223
F000	1096.0	1096.0
F000’	1097.49	
h,k,lmax	14,41,14	14,41,14
Nref	8821	8712
Tmin,Tmax	0.928,0.947	0.559,0.746
Tmin’	0.928	

Correction method= # Reported T Limits: Tmin=0.559 Tmax=0.746
AbsCorr = MULTI-SCAN

Data completeness= 0.988 Theta(max)= 30.563

R(reflections)= 0.1072(7836) wR2(reflections)= 0.3324(8712)

S = 1.060 Npar= 351

The following ALERTS were generated. Each ALERT has the format
test-name_ALERT_alert-type_alert-level.
Click on the hyperlinks for more details of the test.

Alert level C

```
DIFMX02_ALERT_1_C  The maximum difference density is > 0.1*ZMAX*0.75
            The relevant atom site should be identified.
PLAT082_ALERT_2_C High R1 Value ................................        0.11 Report
PLAT084_ALERT_3_C High wR2 Value (i.e. > 0.25) ..................       0.33 Report
PLAT097_ALERT_2_C Large Reported Max.  (Positive) Residual Density       1.24 eA-3
PLAT250_ALERT_2_C Large U3/U1 Ratio for <U(i,j)> Tensor(Resd    2)        3.0 Note
PLAT340_ALERT_3_C Low Bond Precision on  C-C Bonds ..............    0.00541 Ang.
PLAT906_ALERT_3_C Large K Value in the Analysis of Variance ......     6.297 Check
PLAT911_ALERT_3_C Missing FCF Refl Between Thmin & STh/L=    0.600       50 Report
                9  1  0,   8  3  1,  10 11  1,  10  2  2,   6  5  3,   6  7  3,
                8  1  4,   5  2  4,   7  2  4,   7  6  4,   4  7  4,   4  8  4,
                6 19  4,   3  6  5,   2  8  5,   7  8  5,  -2  4  6,   4  4  6,
                5 12  6,   0  1  7,   1  1  7,   3  1  7,   0  3  7,   1  3  7,
                3  3  7,   1  5  7,   5  7  7,   5  9  7,  -1 10  7,   4 11  7,
                   (   20 More NOT listed: see .ckf listing file)
PLAT918_ALERT_3_C Reflection(s) with I(obs) much Smaller I(calc) .          4 Check
                0  1  1,   2  1  1,   0  1  3,  -2  3  3,
PLAT975_ALERT_2_C Check Calcd Resid. Dens.  0.86Ang From O35     .       0.90 eA-3
PLAT975_ALERT_2_C Check Calcd Resid. Dens.  1.00Ang From O25     .       0.75 eA-3
PLAT977_ALERT_2_C Check Negative Difference Density on H33A      .      -0.33 eA-3
```

Alert level G

```
PLAT007_ALERT_5_G Number of Unrefined Donor-H Atoms .............          2 Report
             H15     H24
PLAT042_ALERT_1_G Calc. and Reported MoietyFormula Strings  Differ     Please Check
             Calc: C21 H18 N2 O4, 2(C2 H6 O S)
             Rep.: 2(C2 H6 O S), C21 H18 N2 O4
PLAT072_ALERT_2_G SHELXL First  Parameter in WGHT  Unusually Large       0.16 Report
PLAT083_ALERT_2_G SHELXL Second Parameter in WGHT  Unusually Large      19.18 Why ?
PLAT302_ALERT_4_G Anion/Solvent/Minor-Residue Disorder (Resd    2)        75% Note
PLAT605_ALERT_4_G Largest Solvent Accessible VOID in the Structure        257 A**3
PLAT793_ALERT_4_G Model has Chirality at C3          (Centro SpGr)          S Verify
PLAT793_ALERT_4_G Model has Chirality at C4          (Centro SpGr)          R Verify
PLAT793_ALERT_4_G Model has Chirality at C6          (Centro SpGr)          R Verify
PLAT793_ALERT_4_G Model has Chirality at C7          (Centro SpGr)          S Verify
PLAT868_ALERT_4_G ALERTS Due to the Use of _smtbx_masks Suppressed          ! Info
PLAT910_ALERT_3_G Missing FCF Reflection(s) Below Theta(Min)[Deg]=       2.25 Note
```

```
                0  2  0,
PLAT912_ALERT_4_G Missing # of FCF Reflections Above STh/L=  0.600         58 Note
PLAT933_ALERT_2_G Number of HKL-OMIT Records in Embedded .res File          4 Note
                0  3  7,  -3  1  8,  -3  3  8,   4  4  6,
PLAT969_ALERT_5_G The 'Henn et al.' R-Factor-gap value ..........      9.877 Note
              Predicted wR2: Based on SigI**2  3.37 or SHELX Weight 31.35 Note
PLAT978_ALERT_2_G Number C-C Bonds with Positive Residual Density.          1 Info
```

```
   0 ALERT level A = Most likely a serious problem - resolve or explain
   0 ALERT level B = A potentially serious problem, consider carefully
  12 ALERT level C = Check. Ensure it is not caused by an omission or oversight
  16 ALERT level G = General information/check it is not something unexpected

   2 ALERT type 1 CIF construction/syntax error, inconsistent or missing data
  10 ALERT type 2 Indicator that the structure model may be wrong or deficient
   6 ALERT type 3 Indicator that the structure quality may be low
   8 ALERT type 4 Improvement, methodology, query or suggestion
   2 ALERT type 5 Informative message, check
```

It is advisable to attempt to resolve as many as possible of the alerts in all categories. Often the minor alerts point to easily fixed oversights, errors and omissions in your CIF or refinement strategy, so attention to these fine details can be worthwhile. It is up to the individual to critically assess their own results and, if necessary, seek expert advice.

PLATON version of 20/06/2026; check.def file version of 19/06/2026

Datablock trif_a2_0m_a - ellipsoid plot

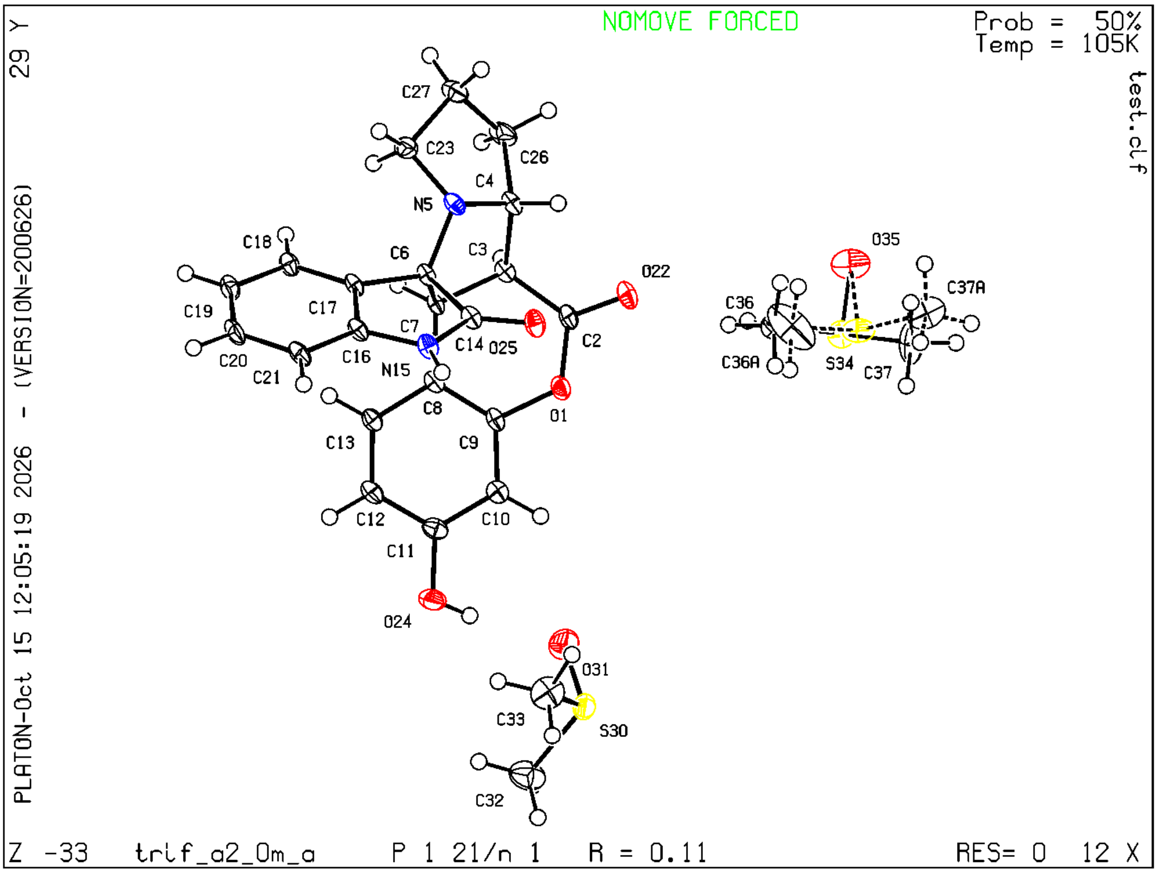

checkCIF/PLATON report

Structure factors have been supplied for datablock(s) klk1_0m_a

THIS REPORT IS FOR GUIDANCE ONLY. IF USED AS PART OF A REVIEW PROCEDURE FOR PUBLICATION, IT SHOULD NOT REPLACE THE EXPERTISE OF AN EXPERIENCED CRYSTALLOGRAPHIC REFEREE.

No syntax errors found. CIF dictionary Interpreting this report

Datablock: klk1_0m_a

Bond precision: C-C = 0.0031 A Wavelength=0.71073

Cell:	a=15.8032(15)	b=6.2603(6)	c=16.5254(16)
	alpha=90	beta=96.234(3)	gamma=90
Temperature:	293 K		

	Calculated	Reported
Volume	1625.2(3)	1625.2(3)
Space group	P 21/n	P 1 21/n 1
Hall group	-P 2yn	-P 2yn
Moiety formula	C19 H16 N2 O4, H2 O	C19 H16 N2 O4, H2 O
Sum formula	C19 H18 N2 O5	C19 H18 N2 O5
Mr	354.35	354.35
Dx,g cm-3	1.448	1.448
Z	4	4
Mu (mm-1)	0.106	0.106
F000	744.0	744.0
F000’	744.39	
h,k,lmax	20,8,21	20,8,21
Nref	3923	3922
Tmin,Tmax	0.993,0.997	0.644,0.746
Tmin’	0.963	

Correction method= # Reported T Limits: Tmin=0.644 Tmax=0.746
AbsCorr = MULTI-SCAN

Data completeness= 1.000 Theta(max)= 27.994

R(reflections)= 0.0569(2356) wR2(reflections)= 0.1317(3922)

S = 1.023 Npar= 246

The following ALERTS were generated. Each ALERT has the format
test-name_ALERT_alert-type_alert-level.
Click on the hyperlinks for more details of the test.

Alert level C

```
RINTA01_ALERT_3_C  The value of Rint is greater than 0.12
            Rint given   0.180
PLAT906_ALERT_3_C Large K Value in the Analysis of Variance ......      5.274 Check
PLAT906_ALERT_3_C Large K Value in the Analysis of Variance ......      2.279 Check
```

Alert level G

```
PLAT007_ALERT_5_G Number of Unrefined Donor-H Atoms .............          2 Report
              H15     H24
PLAT020_ALERT_3_G The Reported Value of Rint is Greater Than  0.12      0.180 Report
PLAT199_ALERT_1_G Reported _cell_measurement_temperature ..... (K)        293 Check
PLAT200_ALERT_1_G Reported   _diffrn_ambient_temperature ..... (K)        293 Check
PLAT793_ALERT_4_G Model has Chirality at C3          (Centro SpGr)          R Verify
PLAT793_ALERT_4_G Model has Chirality at C4          (Centro SpGr)          R Verify
PLAT793_ALERT_4_G Model has Chirality at C7          (Centro SpGr)          R Verify
PLAT883_ALERT_1_G Absent Datum for _atom_sites_solution_primary ..     Please Do !
PLAT910_ALERT_3_G Missing FCF Reflection(s) Below Theta(Min)[Deg]=       1.89 Note
               -1  0  1,
PLAT933_ALERT_2_G Number of HKL-OMIT Records in Embedded .res File          1 Note
               -1  0  1,
PLAT967_ALERT_5_G Note: Two-Theta Cutoff Value in Embedded .res ..       56.0 Degree
PLAT969_ALERT_5_G The 'Henn et al.' R-Factor-gap value ..........      1.480 Note
              Predicted wR2: Based on SigI**2  8.90 or SHELX Weight 12.87 Note
PLAT978_ALERT_2_G Number C-C Bonds with Positive Residual Density.          5 Info
```

---

0 **ALERT level A** = Most likely a serious problem - resolve or explain
0 **ALERT level B** = A potentially serious problem, consider carefully
3 **ALERT level C** = Check. Ensure it is not caused by an omission or oversight
13 **ALERT level G** = General information/check it is not something unexpected

3 ALERT type 1 CIF construction/syntax error, inconsistent or missing data
2 ALERT type 2 Indicator that the structure model may be wrong or deficient
5 ALERT type 3 Indicator that the structure quality may be low
3 ALERT type 4 Improvement, methodology, query or suggestion
3 ALERT type 5 Informative message, check

It is advisable to attempt to resolve as many as possible of the alerts in all categories. Often the minor alerts point to easily fixed oversights, errors and omissions in your CIF or refinement strategy, so attention to these fine details can be worthwhile. It is up to the individual to critically assess their own results and, if necessary, seek expert advice.

PLATON version of 23/04/2026; check.def file version of 30/03/2026

---

duplicate check

No duplication found

Datablock klk1_0m_a - ellipsoid plot

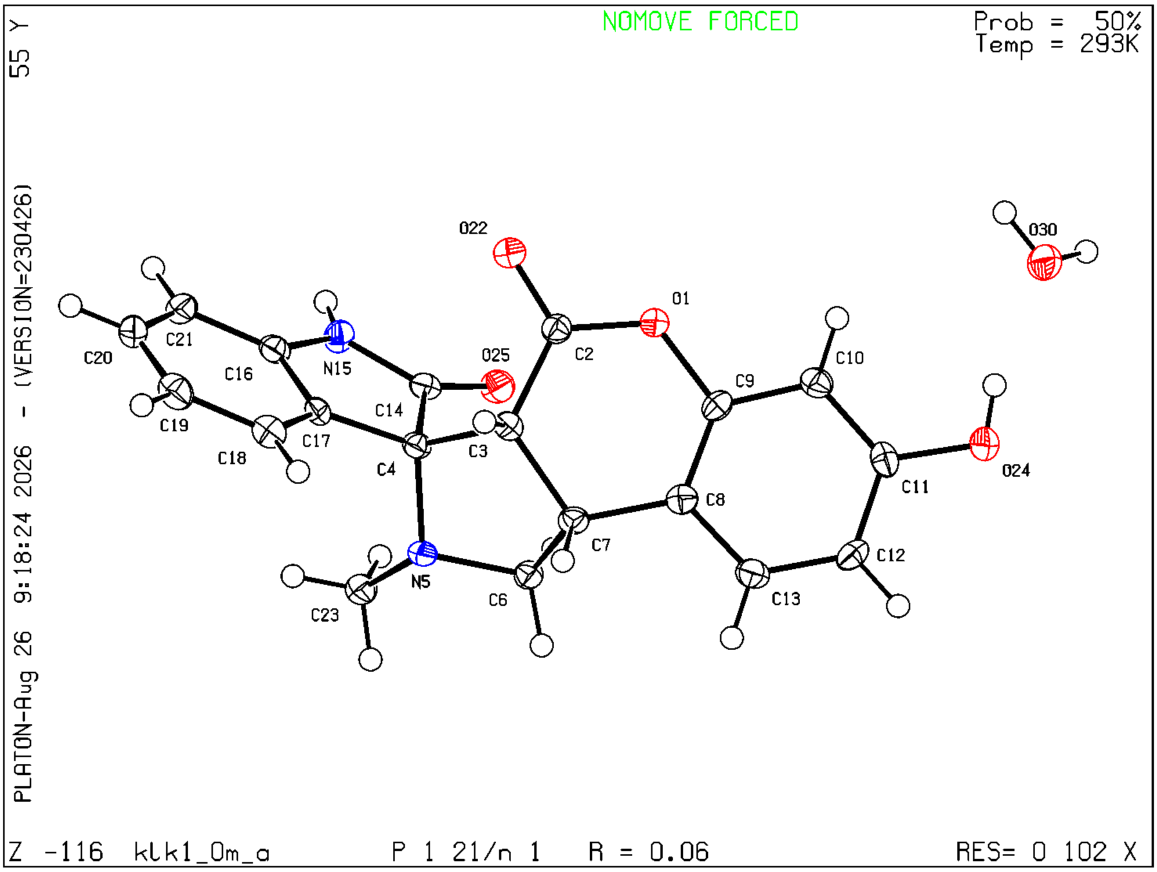

Supplement: Supplementary file 1 [file ijms-27-09600-s001.zip › ijms-4543378-Supplementary_Materials.pdf]
